# Multi-Subunit SARS-CoV-2 Vaccine Design Using Evolutionarily Conserved T- and B- Cell Epitopes

**DOI:** 10.3390/vaccines9070702

**Published:** 2021-06-26

**Authors:** Burkitkan Akbay, Syed Hani Abidi, Mahmoud A. A. Ibrahim, Zhussipbek Mukhatayev, Syed Ali

**Affiliations:** 1 Department of Biomedical Sciences, Nazarbayev School of Medicine, Nazarbayev University, Nur-Sultan 010000, Kazakhstan; burkitkan.akbay@nu.edu.kz (B.A.); zhussipbek.mukhatayev@nu.edu.kz (Z.M.); 2 Department of Biological and Biomedical Sciences, Aga Khan University, Karachi 74800, Pakistan; 3 Computational Chemistry Laboratory, Chemistry Department, Faculty of Science, Minia University, Minia 61519, Egypt; m.ibrahim@compchem.net

**Keywords:** human coronaviruses, MERS, SARS-CoV-2, SARS-CoV, epitope, vaccine

## Abstract

The SARS-CoV-2 pandemic has created a public health crisis worldwide. Although vaccines against the virus are efficiently being rolled out, they are proving to be ineffective against certain emerging SARS-CoV-2 variants. The high degree of sequence similarity between SARS-CoV-2 and other human coronaviruses (HCoV) presents the opportunity for designing vaccines that may offer protection against SARS-CoV-2 and its emerging variants, with cross-protection against other HCoVs. In this study, we performed bioinformatics analyses to identify T and B cell epitopes originating from spike, membrane, nucleocapsid, and envelope protein sequences found to be evolutionarily conserved among seven major HCoVs. Evolutionary conservation of these epitopes indicates that they may have critical roles in viral fitness and are, therefore, unlikely to mutate during viral replication thus making such epitopes attractive candidates for a vaccine. Our designed vaccine construct comprises of twelve T and six B cell epitopes that are conserved among HCoVs. The vaccine is predicted to be soluble in water, stable, have a relatively long half-life, and exhibit low allergenicity and toxicity. Our docking results showed that the vaccine forms stable complex with toll-like receptor 4, while the immune simulations predicted that the vaccine may elicit strong IgG, IgM, and cytotoxic T cell responses. Therefore, from multiple perspectives, our multi-subunit vaccine design shows the potential to elicit a strong immune-protective response against SARS-CoV-2 and its emerging variants while carrying minimal risk for causing adverse effects.

## 1. Introduction

The novel severe acute respiratory syndrome coronavirus 2 (SARS-CoV-2), has caused a worldwide pandemic that continues to negatively impact the global economy, social dynamics, and health care systems [1]. SARS-CoV-2 has been identified as the seventh coronavirus that can infect humans [2]. Although coronaviruses have infected the human population repeatedly over the past century [3], only a few have caused severe large-scale outbreaks. Prior to SARS-CoV-2, the SARS-CoV outbreak occurred in southern China in November 2002, followed by the Middle East respiratory syndrome (MERS-CoV) outbreak in Saudi Arabia and South Korea, respectively, in 2012 and 2015. SARS-CoV and MERS-CoV have an estimated mortality rate of ~9.6% and 35%, respectively [4,5]. Moreover, four other human coronaviruses (HCoVs) associated with mild upper respiratory symptoms, have previously been identified, i.e., HCoV-NL63, HCoV-229E, HCoV-OC43, and HCoV-HKU1 [6,7,8].

The genomes of SARS-CoV and MERS-CoV are, respectively, 79% and 50% similar to SARS-CoV-2 [9]. Out of these HCoVs, SARS-CoV and SARS-CoV-2 not only share a phylogenetic relationship, but also exhibit a high level of similarity in the cell entry mechanism and the use of human cell receptors [10,11]. 

The urgency for having an effective vaccine against SARS-CoV-2 has given rise to fervent efforts toward vaccine design worldwide [12,13]. Over 200 candidate vaccines have been developed, and several of them are currently authorized for use in different parts of the world and have shown varying efficacy [14]. Two mRNA-based vaccines, BNT162b2 (Pfizer–BioNTech, New York, NY, USA and Mainz, Rhineland-Palatinate, Germany), mRNA-1273 (Moderna, Cambridge, MA, USA), showed up to 95% efficacy, the adenovirus vector-based Ad26.CoV2.S (Johnson & Johnson, New Brunswick, NJ, USA) presented 72% efficacy in the USA but 57% in South Africa, while the chimpanzee adenovirus vector-based ChAdOx (AZD1222) (AstraZeneca, Cambridge, UK) presented 70% efficacy. Similarly, heterologous recombinant adenovirus vector-base Gam-COVID-Vac (Sputnik V, Moscow, Russia) demonstrated 91% efficacy in the preliminary phase 3 trials, while the protein-based vaccine NVX-CoV237 (Novavax, Gaithersburg, MD, USA) was 89% efficient in the U.K. but 49% in South Africa [14,15,16,17,18]. Two inactivated virus vaccines, CoronoVac (Sinovac, Beijing, China) and BBIBP-CorV (Sinopharm, China National Pharmaceutical Group, Beijing, China), passed through phase 3 clinical trials and were reported to show from 50% to 91% efficacy. The data, however, were not published at the time of writing of this manuscript. In all of the vaccines that have been designed so far, the antigenic target used was the spike protein, which is the most antigenic, highly variable, and prone to mutations [19,20]. Due to a high substitution/mutation rate and rapid transmissions, new variants of SARS-CoV-2 have been continuously emerging globally, posing threats to circumvent the efficacy of current vaccines, possibly leading to a novel-SARS-CoV-2-variant-assciated surge in transmissions [15]. In fact, there have already been reports of the current vaccines losing effectiveness against the emerging SARS-CoV-2 variants [21,22]. Therefore, vaccines that can stay effective against the emerging strains while simultaneously exhibiting an acceptable safety profile are the need of the hour.

One possible way to design such vaccines is to include epitopes, aside from spike, from SARS-CoV-2 that are evolutionary conserved. Structural proteins of coronaviruses, including spike (S), envelope (E), membrane (M), and nucleocapsid (N), have been shown to elicit greater immunogenicity compared to those against the non-structural proteins [23]. A vaccine bearing a design that is based on conserved epitopes of structural proteins is likely to remain effective against emerging variants since mutations in conserved regions, as they incur a fitness cost to the virus, are not likely to occur. Corroborating that argument are several studies showing that individuals with previous exposure to other HCoVs exhibit high protection against SARS-CoV-2 [24], indicating that due to their functional significance, certain antigenic regions have remained conserved in all HCoVs. 

In this study, using in silico analyses, we designed a multi-subunit chimeric SARS-CoV-2 vaccine comprising of CD8+ T and linear B cell epitopes originating from the regions of envelope (E), membrane (M), nucleocapsid (N), and spike (S) proteins found to be conserved among several human coronaviruses. Evolutionary conservation of these motifs implies their critical roles in viral fitness, indicating that they are unlikely to become mutated during viral replication, making them attractive candidates for a SARS-CoV-2 subunit vaccine that promises to provide coverage against multiple HCoVs, including SARS-CoV-2 and its emerging variants.

## 2. Materials and Methods 

### 2.1. Nucleotide Sequence Retrieval and Phylogenetic Analysis

In the first step, we used E, N, M, and S reference sequences for all seven HCoVs, available at the NCBI Genbank database, namely, SARS-CoV-2 (NC_045512), SARS-CoV (NC_004718), MERS-CoV (NC_019843), HCoV-NL63 (NC_005831), HCoV-229E (NC_002645), HCoV-HKU1 (NC_006577), and HCoV-OC43 (NC_006213) to retrieve nucleotide sequences for each HCoV exhibiting >90% sequence similarity to the reference sequences. The retrieved sequences were aligned using the online MAFFT alignment tool (https://mafft.cbrc.jp/alignment/server/ (accessed on 23 May 2020)). In order to analyze the evolutionary relationship between SARS-CoV-2 with other HCoVs (SARS-CoV, MERS-CoV, HCoV-NL63, HCoV-229E, HCoV-OC43, and HKU-1), nucleotide alignment was used to construct a maximum-likelihood (ML) phylogenetic tree using IQ-TREE v1.6.12 [25]. The ML tree was constructed using the general time reversible (GTR) model of nucleotide substitution and gamma correction for among site rate variation. The ultrafast bootstrap method was used with 1000 replicates to infer support branching in the tree topology. The Newick file for the ML tree was visualized using FigTree v1.4.4.

### 2.2. Protein Sequence Retrieval and Vaccine Design Workflow 

In the next step, E, N, M, and S reference sequences for all seven HCoVs (described above) were used in BLAST search (pBLAST), with filters of 90–100% percent identity and 100% query coverage, to retrieve E, N, M, and S protein sequences from HCoVs that are over 90% conserved. The total number of protein sequences from each HCoV used in our study are shown in Table 1.

**Figure 1:**
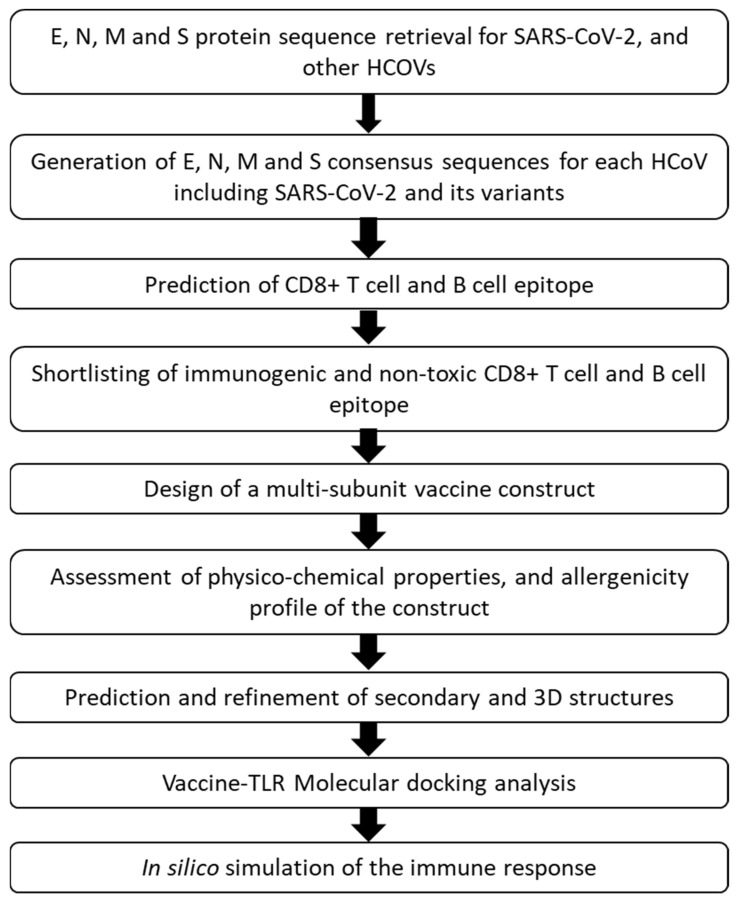
Vaccine design workflow.

Subsequently, the E, N, M, and S protein sequences for each HCoV were separately aligned using an online MAFFT alignment tool [26], and each alignment was used to generate protein specific consensus sequences for each HCoV with the Consensus Maker tool (https://www.hiv.lanl.gov/content/sequence/CONSENSUS/SimpCon.html (accessed on 25 May 2020)). In the next step, protein-specific consensus sequences from the HCoVs were aligned again, and the conserved regions within the E, N, M, and S proteins from the seven HCoVs were identified using ESPript 3.x [27] and Jalview 2.11.1.3 [28] software. Additionally, to take into account the recently emerging mutations among SARS-CoV-2 variants, approximately 5000 whole genome sequences of three SARS-CoV-2 variants:B.1.1.7 (VOC-202012/01) identified in the U.K., 501Y.V2 (B.1.351) identified in the South Africa, and 501Y.V3 (P.1) identified in Brazil were retrieved from GSAID (https://www.gisaid.org/ (accessed on 5 April 2021), and aligned with MAFFT to generate consensus sequences for each of the variants using BioEdit [29]. Nucleotide consensus sequences were then translated into amino acid sequences using the Expasy tool (https://web.expasy.org/translate/ (accessed on 5 April 2021)). The resulting amino acid sequences were trimmed with reference to E, N, M, and S reference protein sequences in Aliview [30]. The protein-specific consensus sequences for each HCoV, including the SARS-CoV-2 variants, were used to predict B and T cell epitopes and to design a multi-subunit vaccine based on the selected epitopes as described below (Figure 1). 

### 2.3. In Silico CD8+ T Cell and B Cell Epitope Mapping, and Epitope Conservation Analysis 

Using E, N, M, and S protein consensus sequences, HCoV-derived cytotoxic T cell epitopes were predicted using the web-based CTLPred tool [31] and a combined support vector machine and artificial neural network approach. Human leukocyte antigen (HLA) restriction was identified using the nHLAPred tool for each predicted T cell epitope [32]. The prediction of linear B cell epitopes was performed using the web-based ABCpred tool [33]. Epitopes located in the conserved regions between the 7 HCoVs or 3 closely related viruses, namely, SARS-CoV-2, SARS-CoV, and MERS-CoV, were selected. Predicted CD8+ T and B cell epitopes were compared to the experimentally validated SARS-CoV epitopes available in IEDB (http://www.iedb.org/ (accessed on 10 April 2021)) database [34] and that had been reported by other studies.

### 2.4. Selection of Immunogenic and Non-Toxic Epitopes for Multi-Subunit Vaccine Design

The predicted epitopes were shortlisted after screening for immunogenicity and toxicity. VaxiJen v2.0 [35] was used to evaluate immunogenicity (a score >0.4 (default value) was considered as antigenic), while ToxinPred [36] was used to evaluate the toxicity of the epitopes. Epitopes predicted to be immunogenic and non-toxic were selected to design the multi-subunit vaccine. 

### 2.5. Design of a Multi-Subunit Vaccine 

As indicated earlier, the conserved epitopes predicted to be immunogenic and non-toxic were used to design a multi-subunit vaccine (Supplementary Figure S1). For the vaccine design, the CD8+ T cell epitopes were linked using an AAY linker, and linear B cell epitopes were linked by an GPGPG linker [37]. At the C-terminal of the vaccine, a six-histidine tail was added while at the N-terminus, β-defensin amino acid sequence (UniProt ID: Q5U7J2) was added to the first epitope via an EAAAK linker. β-defensin acts as an adjuvant and mediates recruitment of naïve T cells and immature dendritic cells at the site of infection/immune activity [38]. 

### 2.6. Evaluation of Physico-Chemical Properties, and Allergenicity Profile of the Multi-Subunit Vaccine

Physico-chemical properties, such as half-life, instability index, aliphatic index, and grand average of hydrophobicity of the vaccine were determined using the ProtParam server (http://web.expasy.org/protparam (accessed on April 12 2021)), while the basic property of allergenicity of the multi-subunit vaccine was assessed using AllerTOP v. 2.0 server [39]. The immunogenicity/antigenicity of the multi-subunit vaccine was evaluated using the VaxiJen v2.0 server [35]. 

### 2.7. Prediction and Refinement of Secondary and 3D Structures 

The secondary structure of the designed vaccine was predicted using the PSIPRED server [40], while the 3D structure was predicted using both the I-TASSER [41] and the Phyre2 server (using an intensive modelling approach) [42]. The best model was selected and refined/optimized using the Galaxyrefine server [43], while PROSA [44] and Ramachandran plot analyses were performed to analyze the quality of the refined structure. Solubility of the protein (vaccine) was determined using the Protein-Sol server [45]. 

### 2.8. Molecular Docking Analysis

Since Toll-like receptor 4 (TLR4) is capable of recognizing various exogenous and endogenous ligands such as viral protein, lipopolysaccharide, heat shock proteins, β-defensin, etc., which leads to dendritic cell maturation, and up-regulation of costimulatory molecules resulting in type 1 polarized adaptive immune response [46,47], we performed molecular docking between TLR4 and our vaccine-construct (containing β-defensin as an adjuvant). For molecular docking calculations, the crystal structure of the human TLR4 deposited in the Protein Data Bank with the PDB accession code of 4G8A [48] was retrieved and utilized as a template. For the preparation of the TLR4 crystal structure, all crystallographic water molecules and heteroatoms were removed. Moreover, mutated residues were reversed and refined. The protonation state of the TLR4 was then examined, and hydrogen atoms were consequently added [49]. Binding modes of the developed vaccine with the human TLR4 were then predicted using the PatchDock server [50]. All predicted docked complexes (exactly 1000 solutions) were then refined and rescored using the FireDock server [51,52]. The interactions between the vaccine and the TLR4 were resolved using the LigPlot + software [53].

### 2.9. In Silico Simulation of the Immune Response

The humoral and cellular immune response against the multi-subunit vaccine construct was simulated using the C-ImmSim tool [54]. For simulations, the following parameters were used: three injections at 4-week intervals; simulation steps = 1000; time to simulation = default; injection volume = vaccine only and no LPS. Additionally, we used NetCTL [55] and NetChop [56,57] to predict proteasomal sites (using default a value of 0.5) and the possible proteolytic release of the epitopes from the vaccine construct. 

## 3. Results

### 3.1. Phylogenetic and Sequence Similarity among the Human Coronaviruses 

To evaluate the genetic similarity among the seven human coronaviruses, we constructed ML phylogenetic trees that revealed alpha-HCoVs, NL63 and 229E, clustered distantly from Beta-HCoVs, OC43 and HKU1. Importantly, SARS-CoV-2, SARS-CoV, and MERS-CoV clustered together, indicating a close phylogenetic relatedness between these viruses (Supplementary Figure S2). 

**Figure 2:**
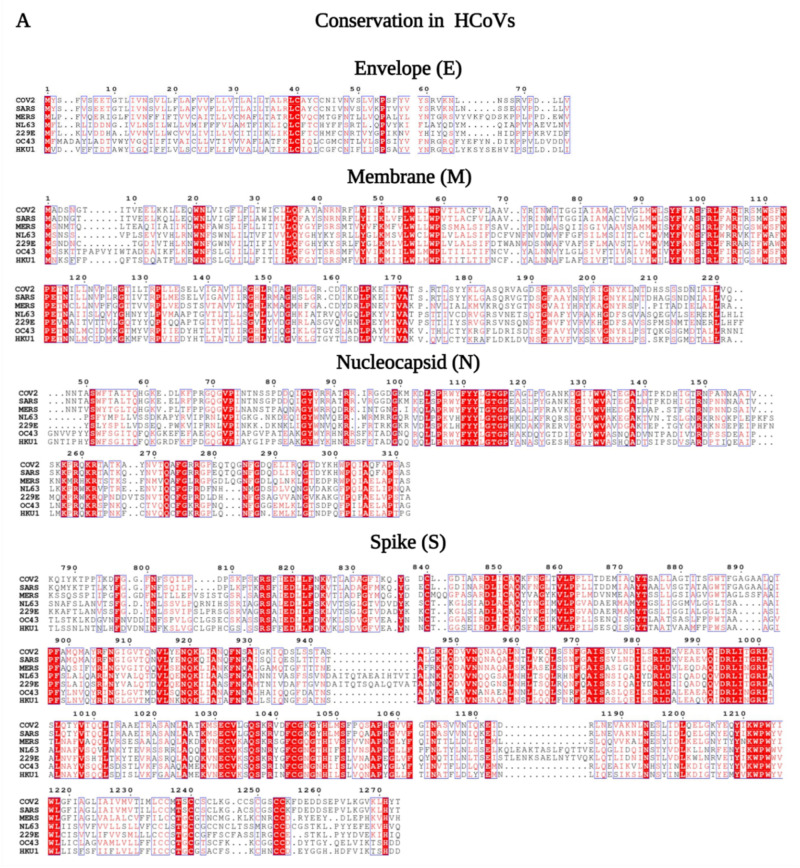

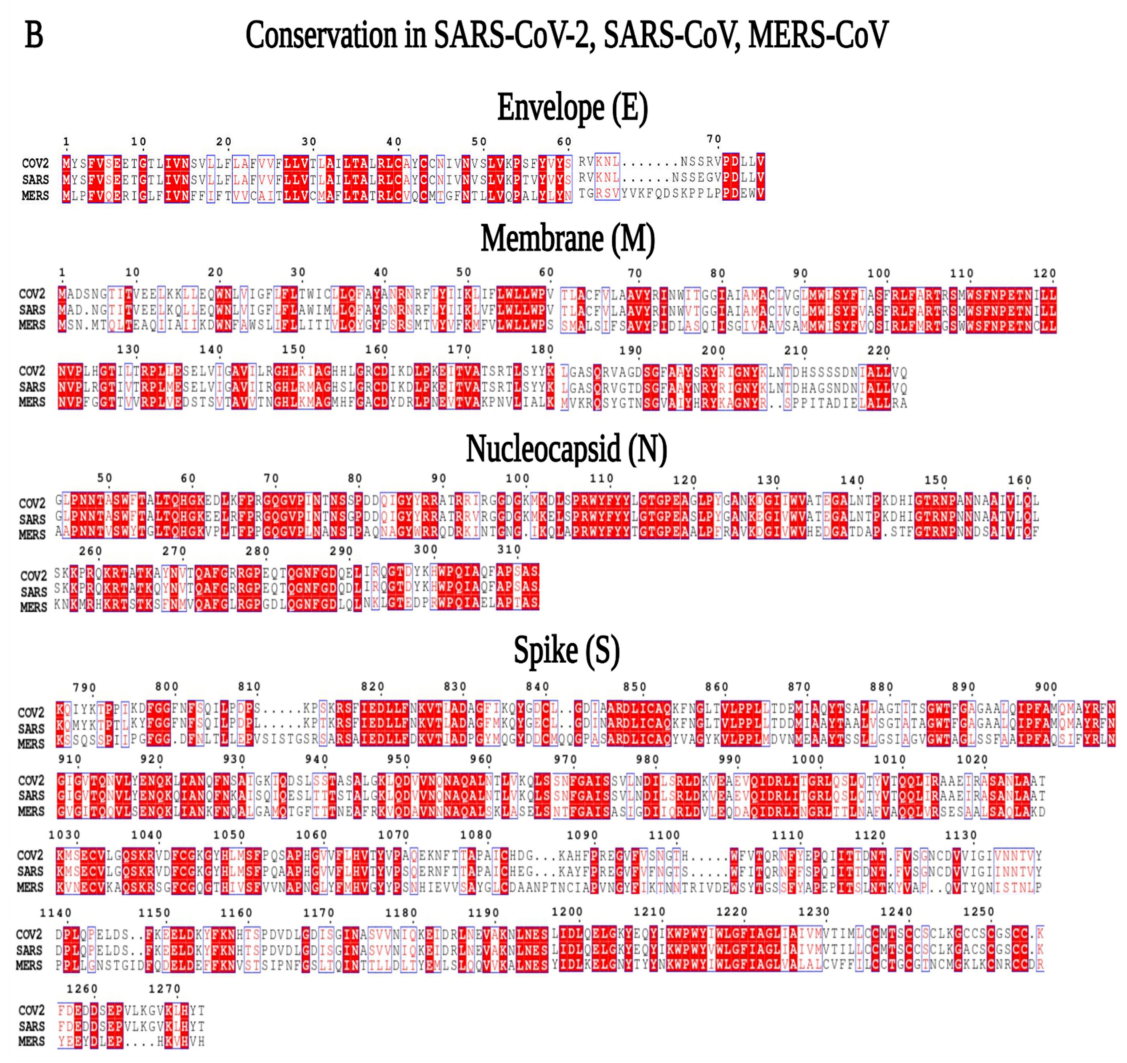
Conservation of envelope (E), membrane (M), nucleocapsid (N), and spike (S) protein sequences in seven HCoVs and in SARS-CoV-2, SARS-CoV, and MERS-CoV. Conservation of E, M, N, and S proteins in (**A**) seven HCoVs and (**B**) SARS-CoV-2 (COV2), SARS-CoV (SARS), and MERS-CoV (MERS). Only the regions showing conservancy are presented for N and S proteins (full length sequences are shown in Supplementary Figures). Images are shown from ESPript results. Conserved regions are colored in red.

In the next step, we identified conserved regions in the consensus sequences of the E, N, M, and S proteins from seven HCoVs (Figure 2A and Supplementary Figure S3) and three phylogenetically closely related HCoVs-SARS-CoV-2, SARS-CoV, and MERS-CoV (Figure 2B and Supplementary Figure S4) using ESPript 3.x and Jalview 2.11.1.3 software [28]. The results revealed several regions within N, M, and S proteins exhibiting more than 40% homology among the seven HCoVs, while no conserved regions were found for the E protein (Supplementary Figure S3), corroborating the findings of the phylogenetic analysis. Additionally, while the conservation was analyzed for SARS-CoV-2, SARS-CoV, and MERS-CoV, the results showed greater sequence conservation for E, N, M, and S compared to those in the seven hCoVs (Supplementary Figures S3 and S4). 

In the next steps, focusing on these conserved regions, we predicted CD8+ T and B cell epitopes for each SARS-CoV-2 protein. 

### 3.2. CD8+ T Cell Epitopes Originating in Conserved Regions of E, N, M, and S Proteins

CD8+ T cell epitopes were analyzed for E, N, M, and S proteins, focusing on the conserved regions of the seven HCoVs protein sequences.

No CD8+ T cell epitopes were identified in the conserved regions of E protein among all seven HCoVs (Supplementary Figure S3 and Table 2). However, when the analysis was carried out using E sequences from only SARS-CoV-2, SARS-CoV and MERS-CoV, two conserved CD8+ T cell epitopes were identified, each exhibiting ~44% and ~77% conservation (Table 3 and Supplementary Figure S4). These epitopes were found to be restricted by HLA- C*0401 HLA-A*0205, HLA-A*0301, HLA-A2, HLA-A3, and HLA-B*5301 (Table 3). Similarly, analysis of the M protein sequences showed the presence of one CD8+ T cell epitope with ~55% conservation among all seven HCoVs (Table 2), which was found to be restricted by HLA-A24, HLA-A*2402, and HLA-Cw*0401 (Table 2). Conservation of this epitope between SARS-CoV-2, SARS-CoV, and MERS-CoV was found to be 66% (Table 2 and Supplementary Figure S4).

Further analysis of SARS-CoV-2, SARS-CoV, and MERS-CoV epitopes revealed three conserved CD8+ T cell epitopes exhibiting ~44%, ~66%, and ~88% conservation between the three viruses (Table 3 and Supplementary Figure S4). These epitopes were found to be restricted by HLA-C*0401 and then by HLA -A*0203, -A*24, -B*2703, and -B*35 (Table 3). 

Analysis of the N protein sequences showed one CD8+ T cell epitope with 44% conservation among all HCoVs (Table 2 and Supplementary Figure S3). This epitope was found to be restricted by HLA-Cw*0401, HLA-A*0202, HLA-A*0203, HLA-A*0205, HLA-A*0301, HLA-A*0401, HLA-B*35, and HLA-B*3501 (Table 2). Again, conservation of this epitope was increased to ~77% when the analysis was focused only on SARS-CoV-2, SARS-CoV, and MERS-CoV (Table 3 and Supplementary Figure S4). Seven additional conserved CD8+ T cell epitopes were identified for the N protein with ~77%, ~66%, and ~55% conservation (Table 3 and Supplementary Figure S4). Most of the conserved epitopes were found to be restricted by HLA- C*0401 and then by HLA-A*0201, HLA-A*2402, HLA-A*A2, HLA-A*6801, HLA-B*3501, HLA-B*51, and HLA-B*5301 (Table 3). 

Unlike E, N, and M proteins, two conserved CD8+ T cell epitopes were found for the S protein among all the HCoVs, with ~44% and ~66% conservation, respectively (Table 2 and Supplementary Figure S3). These epitopes were found to be mainly restricted by HLA- C*0401, HLA-A*0206, and HLA-A*3301 (Table 2). Conservation of these two epitopes increased to ~66% and ~77% in the SARS-CoV-2, SARS-CoV, and MERS-CoV sequences (Table 3 and Supplementary Figure S4). Further analysis of epitopes in the S sequences from these three HCoVs revealed 12 conserved CD8+ T cell epitopes, with ~44%, ~55%, ~66%, ~77%, and ~88% conservation (Table 3 and Supplementary Figure S4). Most of the conserved epitopes were found to be restricted by HLA- C*0401, HLA-A1, HLA-A2, HLA-A*0206, HLA-A24, HLA-A*2402, HLA-A*3301, HLA-A*6801, HLA-B*3501, HLA-B8, HLA-B27, HLA-B*2706, and HLA-B51 (Table 3). 

It is important to note that all CD8+ T epitopes predicted in the E, N, M, and S sequences were found to be 100% conserved in the latest deposited 5000 SARS-CoV-2 sequences as well as in the three variants of SARS-CoV-2 (Supplementary Figure S5). 

### 3.3. Predicted Linear B Cell Epitopes Found to Be Conserved in E, N, M, and S Protein Sequences of HCoVs

E, N, M, and S consensus protein sequences of seven HCoVs were used to predict linear B cell epitopes of varying length (between 10 to 20 amino acids). Even though the protein sequences showed several regions exhibiting more than 40% homology among the seven HCoVs, no B cell epitopes were found in the conserved regions. Analysis of SARS-CoV-2, SARS-CoV, and MERS-CoV, however, revealed conserved B cell epitopes among these three viruses. Two B cell epitopes, with ~42% and ~50% conservation, were predicted for the E protein, while only one epitope, with ~86% conservation, was predicted for the M protein (Table 4, Supplementary Figure S4). Similarly, for the N proteins, four epitopes were found to be conserved in the N protein with ~50%, ~56%, and ~69%, while five epitopes were found to be conserved in the S protein, showing ~50%, ~56%, and ~63% conservation (Table 4, Supplementary Figure S4). 

It is again important to note that all the predicted B cell epitopes for E, N, M, and S were found to be 100% conserved in the latest deposited 5000 SARS-CoV-2 sequences as well as in the three variants (B.1.1.7, B.1.351, and P.1) of SARS-CoV-2 (Table 5, Supplementary Figure S5).

### 3.4. Selection of Immunogenic Non-Toxic CD8+ T and Linear B Cell Epitopes for Design of Multi-Subunit Vaccine 

In the next step, the immunogenicity and toxicity profile of all the predicted epitopes found to be conserved in the E, N, M, and S proteins of SARS-CoV-2 and other HCoVs was evaluated. Among the CD8+ T cell epitopes, one out of two epitopes for E, two out of four epitopes for M, three out of eight epitopes for N, and six out of fourteen epitopes for S were predicted to be immunogenic and non-toxic, with the highest immunogenic score (1.5) for the peptides of S, followed by M, N, and E (Table 5). From B cell epitopes, two out of two epitopes for E, three out of four epitopes for N, and one of five epitopes for the S protein were predicted to be immunogenic and non-toxic, with the highest immunogenic score (1.8) for the peptides of N, followed by S and E (Table 5). B cell epitope for M, however, was predicted to be non-immunogenic. Therefore, the B cell epitope for M was not selected for further analysis. A total of 18 (12 CD8+ T cell and 6 linear B cell) immunogenic, non-toxic, and conserved epitopes were subsequently used to construct a multi-subunit vaccine. 

### 3.5. Design and Assessment of the Multi-Subunit Vaccine

Physico-chemical assessment of the vaccine construct revealed the estimated half-life of the vaccine to be 30 h in mammalian reticulocytes (in vitro), >20 h in yeast (in vivo), and >10 h in *Escherichia coli* (in vivo). The instability index was predicted to be 33.06, classifying the vaccine as stable. The aliphatic index and grand average of hydropathicity (GRAVY) of the vaccine was found to be −0.250, making the construct hydrophilic and capacitating it to interact better with the neighboring water molecules [37]. The molecular weight of the construct was found to be 34,336.60 daltons. The solubility index of the vaccine construct, based on Protein-Sol calculation, was found to be 0.34, indicating an acceptable solubility profile for the vaccine. Allergenicity and immunogenicity prediction revealed the construct to be a probable antigen (VaxiJen score 0.56) and non-allergen. 

### 3.6. Secondary and Tertiary Structure of the Multiepitope Vaccine:

**Figure 3:**
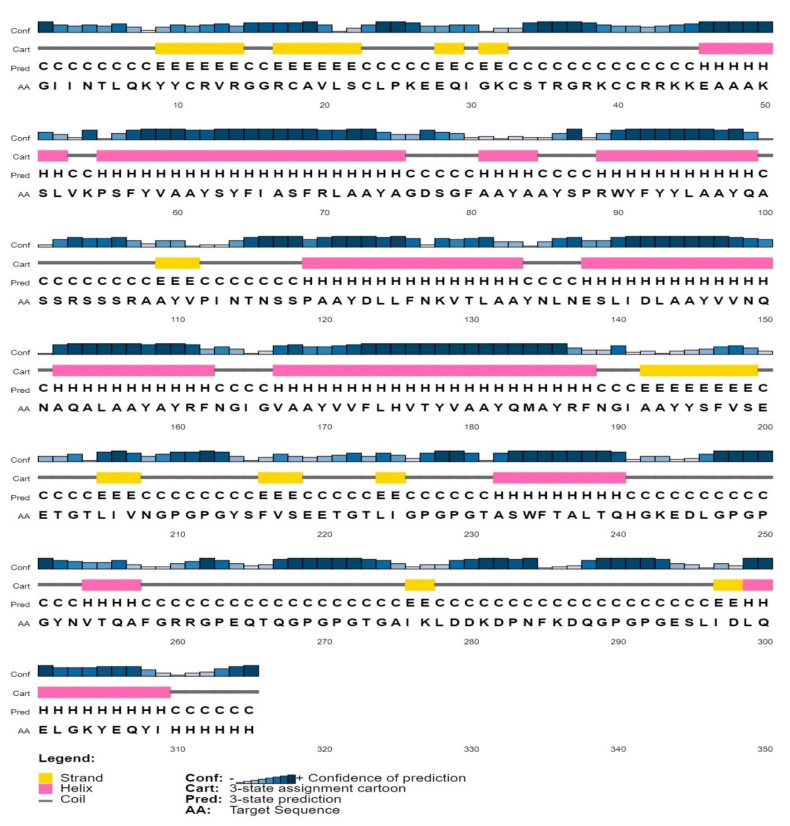
Secondary structure prediction of the vaccine construct. The color-coding of different regions is explained in the legend within the figure. For secondary structure prediction, C, H, and E symbols indicate the coil, helix, and strand regions, respectively.

**Figure 4:**
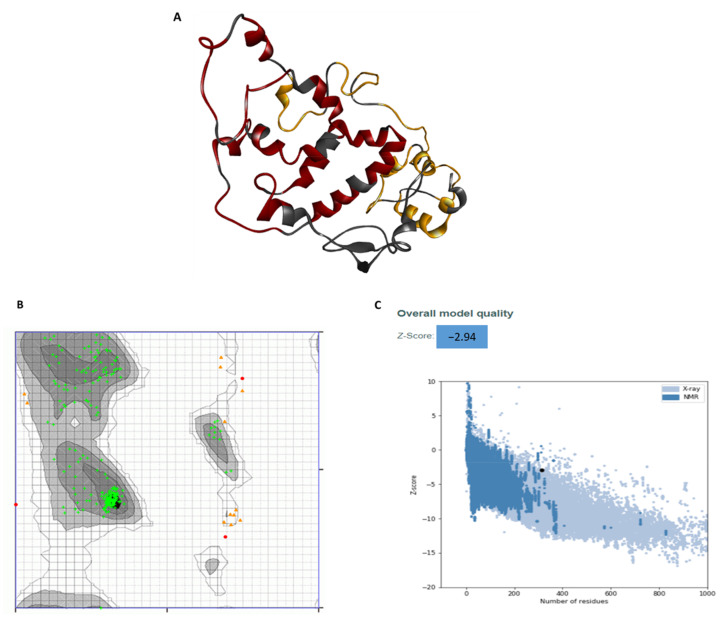
Predicted 3D structure of the vaccine and its quality assessment. (**A**) Predicted 3D structure of the vaccine (black) showing T cell epitopes in red, while B cell epitopes in orange, (**B**) Ramachandran plot for the 3D structure of the vaccine showing 97.8% of the residues in the favorable region, (**C**) ProSA z-score for the 3D structure of the vaccine, determined using NMR spectroscopy (dark blue) and X-ray crystallography (light blue).

Secondary structure assessment of the vaccine construct showed that the construct had 11 helices and 11 strands (Figure 3). The 3D structure of the vaccine was constructed using both the Phyre2 and I-TASSER tools. The structure constructed using I-TASSER had an overall low-quality score after refinement, and on a Ramachandran plot, only 88% residues were in the favorable region (data not shown). To the contrary, the 3D structure predicted by the Phyre2 tool after refinement (Figure 4A) exhibited a high confidence score, ranging from 84–96% for most regions, and 66–74% for some regions. The Ramachandran analysis of the structure showed that the predicted structure had 94.3% of the residues in a highly preferred region, 4.5% in a preferred region, and only 1.1% residues in a non-preferred/disallowed region (Figure 4B). The PROSA score of the structure predicted by Phyre2 was −2.94, which lies in the acceptable range (Figure 4C). Overall, the assessment of the different protein quality parameters indicates the structure predicted by Phyre2 to be of high quality.

### 3.7. Molecular Docking Analysis of the Vaccine–TLR4 Complex

**Figure 5:**
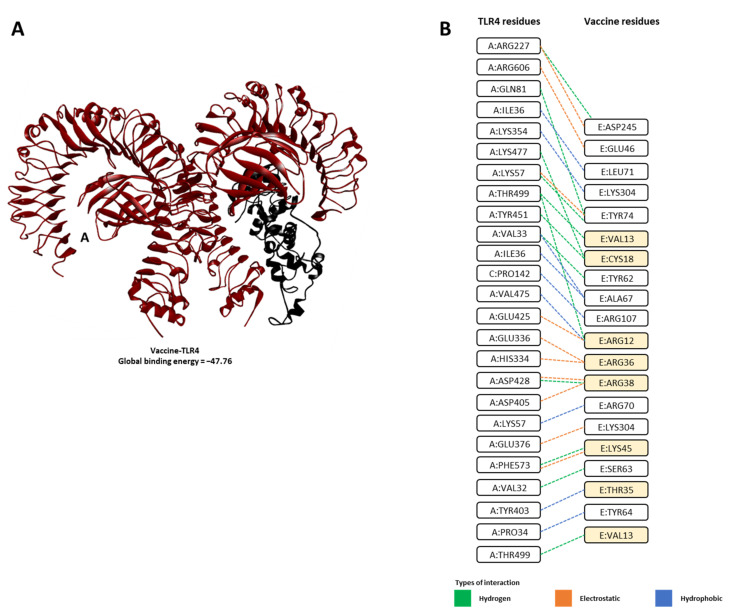
Molecular docking analysis between vaccine construct and TLR4. (**A**) Docked complex of the designed vaccine with TLR4, (**B**) amino acid interaction between vaccine–TLR4 (vaccine construct: chain E; TLR4: chain A; C: Lymphocyte antigen 96), resolved using LigPlot+ (DIMPLOT). Residues that are part of β-defensin found to interact with TLR4 are highlighted in orange.

To reveal the binding modes and energies of the developed vaccine with human TLR4, the molecular docking technique was utilized. All possible vaccine–TLR4 solutions (exactly 1000 solutions) were first predicted using the PatchDock server [50]. The predicted vaccine–TLR4 complex solutions were then refined, and the global binding energies were rescored using the FireDock server [51,52]. The 3D representation of the predicted vaccine–TLR4 complex structure is depicted in Figure 5, and the estimated global binding energy and energy contributions for the top-ranked vaccine–TLR4 complex are listed in Table 6. Interactions between the vaccine–TLR4 complex were resolved using LigPlot+ software. 

The global binding energy estimates suggest that our vaccine construct formed a stable complex with TLR4, with global binding energy of −47.76 kcal/mol(Table 6 and Figure 5A). Assessment of the energy contributions revealed that attractive van der Waals (vdW) was the dominant force in vaccine–TLR4 binding energy, with a value of −36.33 kcal/mol (Table 6). Analysis of the protein–protein interaction suggested that the construct formed multiple interactions with TLR4, especially with several residues of β-defensin (adjuvant used in our construct, and a known agonist of TLR4 [46,47] were also found to interact with TLR4 (Figure 5B).

### 3.8. In Silico Simulation of the Immune Response against the Vaccine Construct

**Figure 6:**
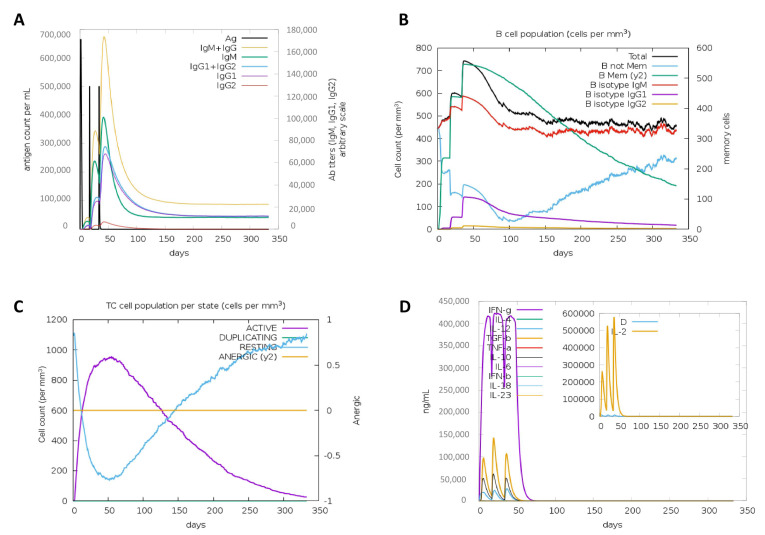
Simulated immune response after administration of the vaccine construct: The graphs show stimulation of (**A**) IgG, IgM, and total antibody response, (**B**) Effector and memory B cell response, (**C**) Active and anergic CD8+ T cell response, and (**D**) IFN-gamma and other cytokine responses after vaccine administration; (**D**) (insert): plot shows levels of danger signal (positive or negative signals immune systems generate in response to ‘danger’ such as pathogens (Ramadan et al., 2017)) together with leukocyte growth factor IL-2 over days.

We performed in silico immune simulations to examine the potential of our vaccine construct to elicit potent and durable B (including antibody response) and T cell responses. Results from proteasomal processing showed that the proteasomal sites in the vaccine are strategically located to release all 9-mer T cell epitopes and variable-length B cell epitopes (Supplementary Figure S2). Results from the immune simulation showed that the vaccine construct was predicted to elicit significant titers of IgG and IgM (Figure 6A), significant effector and memory B-cell response (Figure 6B) as well as cytotoxic T cell and interferon-gamma response (Figure 6C). Overall, the vaccine construct was predicted to be stable, non-toxic and highly immunogenic, and capable of mounting a sustained protective immune response. 

## 4. Discussion

Using immunoinformatic tools, we have designed, constructed, evaluated, and assessed the performance of a non-allergenic and antigenic chimeric multi-epitope vaccine that combines T and B cell epitopes from conserved regions of the E, M, N, and S proteins of seven human coronaviruses. The vaccine shows potential to elicit a strong response against SARS-CoV-2 while also providing immune protection against emerging SARS-CoV-2 variants and other HCoVs.

All of the currently available vaccines target the SARS-CoV-2 S protein, which is the most antigenic, highly variable, and prone to mutations [19,20]. Due to the high substitution/mutation rate and rapid transmissions, new variants of SARS-CoV-2 have been continuously emerging globally, posing threats to circumvent the efficacy of current vaccines, possibly leading to a novel-SARS-CoV-2-variant-assciated surge in transmissions [15]. In fact, there have already been reports of the current vaccines losing effectiveness against the emerging SARS-CoV-2 variants [21,22]. Variants of high concern, B.1.1.7 (VOC-202012/01) identified in the U.K.; 501Y.V2 (B.1.351) identified in the South Africa; and P.1 (B.1.1.28.1) identified in Brazil, all have more than 20 mutations in the S protein, raising the concern that both natural infection-derived immunity as well as vaccine-derived immunity may fail to confer protection against a SARS-CoV-2 variant (re)infection [15,21]. This hypothesis has been partly supported by a recent study showing that the 501Y.V2 (B.1.351) variant presents a complete immune escape from natural immunity [67]. Nonetheless, efforts to design new vaccines or to improve the existing vaccines that can confer immunity against SARS-CoV-2 and its variants are underway. In this regard, numerous in silico studies have also been conducted to design multi-epitope vaccine candidates against SARS-CoV-2. However, most of these studies focused on epitopes from SARS-CoV-2 S protein as the antigenic targets [37,68,69,70,71], while others included the N and M proteins [72,73].

The primary structure of the protein can provide information regarding mutations and the conservation of residues, while the secondary structure, depicting the spatial orientation, such as β-folds and α-helices, can give insights into how the protein will fold into its final form. Finally, the tertiary structure depicts the three-dimensional orientation of the protein in space [74]. The nature of the amino acids and protein folding can affect the physio-chemical properties of the protein, such as solubility, stability and interaction with other molecules, etc. [74]. The physico-chemical analyses revealed the molecular weight of our vaccine, 34.34 kDa, indicating that it is an ideal vaccine candidate, as proteins with a molecular weight less than 100 kDa are considered to be efficient vaccine candidates [70]. The instability index of our vaccine construct was shown to be 33.06, indicating that our construct is stable, as proteins with an index greater than 40 are generally considered unstable [38]. The vaccine construct was also found to be thermo-stable, with an aliphatic index of 72.00. A high aliphatic index is an indication of a protein to be thermo-stable over a wide temperature range [75]. The GRAVY index was predicted to be −0.250, reflecting high solubility of the vaccine and its effective interaction with water molecules [76]. The half-life of the vaccine was found to be 30 h in mammalian reticulocytes (in vitro), >20 h in yeast (in vivo), and >10 h in *Escherichia coli* (in vivo). Vaccine candidates with similar a half-life have been found to induce strong humoral and cellular responses in mice [77]. Finally, a refined and validated vaccine model showed that the designed vaccine is of high quality, with >97% of residues in the favored region [78].

In our study, we analyzed all the structural proteins, E, N, M, and S, not only in SARS-CoV-2 and its known variants, but also from six other human coronaviruses, and selected highly conserved epitopes with the aim of designing an effective multi-subunit vaccine that can be effective against SARS-CoV-2 and its variants and may also confer cross-immunoprotection against other HCoVs. Yazdani et al. [79] conducted a similar study using the E, N, M, and S proteins, however, this work investigated the conservation of peptides for only SARS-CoV-2 and SARS-CoV. In addition, the final epitopes selected in their study presented a lower antigenicity score (~0.7), while the epitopes we used in our design exhibited a high immunogenicity score of 1.5 for CD8+ T epitopes residing in S protein, and of 1.8 for linear B cell epitopes residing in the N protein. As our multi-subunit vaccine contains peptides from multiple proteins which are 100% conserved in the B.1.1.7, 501Y.V2, and P.1 variants SARS-CoV-2, mutations that potentially reduce immunogenicity are not likely to occur. The existence of cross-immune reactivity has been documented in several studies [80,81,82,83,84]. It has been reported that the antigenic domains of the S and N proteins are highly cross-reactive across coronaviruses [80,82,83], and it has been shown that T cell immunity previously induced by circulating human alpha- and beta-HCoVs in young adults is protective against severe clinical outcomes [81].

An ideal vaccine candidate, on the one hand, should be immunogenic/antigenic, and should be able to elicit targeted humoral and cellular immune responses, while, on the other hand, should also be non-toxic and non-allergenic [85], hence providing protection against adverse effects. Vaccines using a complete agent (virus, for example) or large proteins carry an increased chance of allergenic response due to the unnecessary antigenic load introduced in the vaccine design [86]. This drawback is avoided in our construct, as we used short immunogenic peptides that were predicted to be non-allergenic and non-toxic. Moreover, the predicted conserved T and B cell epitopes used for our multi-subunit vaccine can elicit cellular and humoral immune responses simultaneously, further increasing the chance of triggering an efficient overall immune response. Additionally, our vaccine was constructed with β-defensin as an adjuvant at the N-terminal, which allows for a long-lasting immune response. β-defensin has been shown to be an efficient adjuvant capable of enhancing the immunogenicity of candidate vaccines against viruses, including MERS-CoV [14,87].

In the immune simulation analyses, these properties were predicted for our vaccine construct as well, showing that the vaccine may be able to elicit a sustained IgG, IgM, B- and cytotoxic T-response, and interferon-gamma response for well over 300 days. The activation/release of interferon-gamma is a hallmark of antiviral activity by T cells, as this cytokine is capable of killing the virus-infected cells [88]. Similarly, the analysis of the immunological response in individuals enrolled in phase 1/2 trial of the ChAdOx1 nCoV-19 vaccine showed that a single dose of the vaccine in adults was able to induce a potent immune response, primarily composed of neutralization antibodies of IgG1 and IgG3 subclasses, TH-1 response with interferon-γ, and tumor necrosis factor-α cytokine secretion by CD4+ T cells as well as mono- and poly-functional and cytotoxic CD8+ T cells up to 8 weeks after vaccination [89]. Similar results were observed for the Pfizer/BioNTech and Moderna vaccines, where robust T cell response against spike protein of SARS-CoV-2 was observed in vaccinated individuals, which was more profound than the response observed in convalescent patients [90]. Moreover, the CD8+ T cell epitopes in our vaccine design were linked with the AYY linker that provides binding sites for the transporter associated with antigen processing (TAP) to facilitate epitope presentation [91], while B cell epitopes were linked with the glycine rich GPGPG linker, which gives the vaccine structural flexibility, prevents junctional epitope formation, and helps immune processing and epitope presentation [91].

Lastly, in molecular docking studies, using TLR4 showed that our vaccine construct, and especially several of β-defensin residues, a known TLR4 agonist [46,47] used as an adjuvant in our vaccine, can form stable complexes with the TLR4. TLR-4 is capable of recognizing various exogenous and endogenous ligands such as viral protein, lipopolysaccharide, heat shock proteins, β-defensin, etc., which leads to dendritic cell maturation and up-regulation of costimulatory molecules, resulting in a type 1 polarized adaptive immune response [46,47]. Recently, Zhao et al. showed that SARS-CoV-2 is able to activate TLR4 to activate an anti-bacterial like immune response [92]. Similarly, other in silico studies have shown that the ability of a vaccine construct to form stable complexes with these TLRs may lead to the induction of antiviral immunity [37,93].

## 5. Conclusions

Multi-epitope vaccines designed using immune-informatics have been widely studied against viruses as well as against cancer [94,95,96,97]. Some of these vaccines have shown promising results in vivo, while some entered clinical trials [98,99,100,101,102]. Analyzing a vaccine construct in silico before proceeding to in vitro evaluations and clinical trials is an efficient way to determine the vaccine’s potential with confidence, economizing on time, effort, and money. Here we present in silico analyses of a multi-subunit vaccine design that, from multiple perspectives, shows potential to elicit a strong immune-protective response against SARS-CoV-2 and its emerging variants, while carrying minimal risk for causing adverse effects.

## Figures and Tables

**Table 1:** Number of protein sequences for envelope, membrane, nucleocapsid, and spike proteins from HCoVs. The table shows the number of sequences used in our study that gave 100% query coverage and >90% conservation between HCoVs.

HCoVs	Protein Sequences			
	Envelope	Membrane	Nucleocapsid	Spike
HCoV-NL63	2	11	36	51
HCoV-229E	5	6	51	71
HCoV-OC43	6	15	106	183
HCoV-HKU1	3	8	28	26
MERS-Cov	18	35	87	250
SARS-CoV	13	23	29	80
SARS-CoV-2	56	176	3082	1139

**Table 2:** Predicted CD8+ T cell epitopes found to be conserved in the seven HCoVs. Conserved amino acids are shown in bold letters. HLA restriction for each epitope is also given.

Conserved CD8 + T Cell Epitopes in HCoVs															
Membrane															
**№**	**SARS-COV-2**	**HLA-Resctriciton**	**SARS-COV**	**HLA-Resctriciton**	**MERS**	**HLA-Resctriciton**	**NL63**	**HLA-Resctriciton**	**229E**	**HLA-Resctriciton**	**OC43**	**HLA-Resctriciton**	**HKU1**	**HLA-Resctriciton**	**Conservation (%)**
1	S**YF**IA**S**F**RL**	HLA-A24,HLA-Cw*0401	S**YF**VA**S**F**RL**	HLA-A24, HLA-A*2402,HLA-Cw*0401	S**YF**VQ**S**I**RL**	HLA-Cw*0401	M**YF**VN**S**F**RL**	HLA-A24,HLA-Cw*0401	M**YF**AN**S**F**RL**	HLA-A24,HLA-Cw*0401	V**YF**VN**S**I**RL**	HLA-A24,HLA-A*2402,HLA-Cw*0401	**YF**VN**S**I**RL**F	HLA-Cw*0401	55
**Nucleocapsid**															
1	S**P**RWY**FYY**L	HLA-Cw*0401	S**P**RWY**FYY**L	HLA-Cw*0401	A**P**RWY**FYY**T	HLA-A*0202,HLA-A*0203,HLA-A*0205,HLA-A*0301,HLA-Cw*0401	P**P**KVH**FYY**L	HLA-A*0301,HLA-Cw*0401	S**P**KLH**FYY**L	HLA-B*3501,HLA-Cw*0401,HLA-B35	L**P**RWY**FYY**L	HLA-A*0301,HLA-Cw*0401,HLA-B35	L**P**RWY**FYY**L	HLA-A*0301,HLA-Cw*0401,HLA-B35	44
**Spike**															
1	**Y**I**KWPW**YI**W**	HLA-A*0206,HLA-Cw*0401	**Y**I**KWPW**YV**W**	HLA-A*0206,HLA-Cw*0401	**Y**N**KWPW**YI**W**	HLA-A*0206,HLA-B*3501,HLA-B*5102,HLA-B*5103,HLA-B*5401,HLA-B7,HLA-Cw*0401,HLA-B35	**Y**I**KWPWW**V**W**	HLA-A2,HLA-A*0206,HLA-Cw*0401	**Y**I**KWPW**WV**W**	HLA-A2,HLA-A*0206,HLA-Cw*0401	**Y**V**KWPW**YV**W**	HLA-A*0206,HLA-B*51, HLA-Cw*0401	**Y**V**KWPW**YV**W**	HLA-A*0206,HLA-B*51,HLA-Cw*0401	66
2	QS**AP**H**G**VV**F**	HLA-Cw*0401,HLA-A*3301	QA**AP**H**G**VV**F**	HLA-Cw*0401,	VN**AP**N**G**LY**F**	HLA-B*5401,HLA-B*51,HLA-B*0702,HLA-Cw*0401	NS**AP**D**G**LL**F**	HLA-Cw*0401	NA**AP**E**G**LV**F**	HLA-Cw*0401	QN**AP**Y**G**LY**F**	HLA-B*5401,HLA-Cw*0401,HLA-A*3301	QN**AP**Y**G**LL**F**	HLA-B*5401,HLA-Cw*0401	44

**Table 3:** Predicted CD8+ T cell epitopes found to be conserved SARS-CoV-2, SARS-CoV, and MERS-CoV. Conserved amino acids are shown in bold letters. HLA restriction for each epitope is also given.

Conserved CD8 + T Cell Epitopes in SARS-CoV-2, SARS-CoV, and MERS-CoV							
Envelope							
№	SARS-CoV-2	HLA-resctriciton	SARS-CoV	HLA-resctriciton	MERS-CoV	HLA-resctriciton	Conservation (%)
1	**A**I**LTA**L**RLC**	HLA-Cw*0401	**A**I**LTA**L**RLC**	HLA-Cw*0401	**A**F**LTA**T**RLC**	HLA-Cw*0401	77
2	S**LV**K**P**SF**Y**V	HLA-Cw*0401	S**LV**K**P**TV**Y**V	HLA-A*0205,HLA-A*0301,HLA-B*5301,HLA-Cw*0401	L**LV**Q**P**AL**Y**L	HLA-A2,HLA-A3,HLA-A*0301,HLA-Cw*0401	44
**Membrane**							
1	**SFNPETN**I**L**	HLA-A*0203,HLA-Cw*0401,	**SFNPETN**I**L**	HLA-A*0203,HLA-Cw*0401,	**SFNPETN**C**L**	HLA-Cw*0401	88
2	**SYF**IA**S**F**RL**	HLA-A24,HLA-Cw*0401	**SYF**VA**S**F**RL**	HLA-A24,HLA-A*2402,HLA-Cw*0401	**SYF**VQ**S**I**RL**	HLA-Cw*0401	66
3	KD**LP**K**E**I**TV**	HLA-Cw*0401	KD**LP**K**E**I**TV**	HLA-Cw*0401	DR**LP**N**E**V**TV**	HLA-B*51,HLA-Cw*0401	66
4	AGD**SG**F**A**A**Y**	HLA-A1,HLA-Cw*0401,HLA-B35,HLA-B*2703	GTD**SG**F**A**A**Y**	HLA-A1,HLA-A3,HLA-Cw*0401,HLA-B35,HLA-B*2703	GTN**SG**V**A**I**Y**	HLA-A3,HLA-Cw*0401,HLA-B35,HLA-B44	44
**Nucleocapsid**							
1	S**PRWYFYY**L	HLA-Cw*0401	S**PRWYFYY**L	HLA-Cw*0401	A**PRWYFYY**T	HLA-A*0202,HLA-A*0203,HLA-A*0205,HLA-A*0301,HLA-Cw*0401	77
2	**ASAF**F**GMS**R	HLA-Cw*0401,HLA-A*6801	**ASAF**F**GMS**R	HLA-Cw*0401,HLA-A*6801	**ASAF**M**GMS**Q	HLA-A*1101,HLA-A3,HLA-A31,HLA-Cw*0401,HLA-A*3301,HLA-A*6801	77
3	**A**N**K**D**GI**I**WV**	HLA-B*3501,HLA-B*5101,HLA-B*5301,HLA-B*5401,HLA-B*51,HLA-B*0702,HLA-Cw*0401,HLA-B35	**A**N**K**E**GI**V**WV**	HLA-B*3501,HLA-B*5101, HLA-B*5301,HLA-B*5401, HLA-B*51,HLA-B*0702,HLA-Cw*0401,HLA-B35	**A**V**K**D**GI**V**WV**	HLA-B*5301,HLA-B*51,HLA-B8,HLA-Cw*0401	66
4	**Q**A**SSR**S**SS**R	HLA-Cw*0401	**Q**A**SSR**S**SS**R	HLA-Cw*0401	**Q**S**SSR**A**SS**V	HLA-B*5301,HLA-Cw*0401	66
5	**L**L**LD**R**LN**Q**L**	HLA-A2,HLA-A*0201,HLA-A*0206,HLA-Cw*0401	**L**L**LD**R**LN**Q**L**	HLA-A2,HLA-A*0201,HLA-A*0206,HLA-Cw*0401	**L**Y**LD**L**LN**R**L**	HLA-A2,HLA-A24,HLA-Cw*0401	66
6	**VP**I**N**T**NS**S**P**	HLA-Cw*0401	**VP**I**N**T**NS**G**P**		**VP**L**N**A**NS**T**P**	HLA-Cw*0401	66
7	**GY**Y**RR**AT**R**R	HLA-Cw*0401	**GY**Y**RR**AT**R**R	HLA-Cw*0401	**GY**W**RR**QD**R**K	HLA-A24,HLA-A*2402,HLA-B*2705,HLA-Cw*0401,HLA-B*2902	55
8	**NP**A**N**NA**A**I**V**	HLA-A*2402,HLA-B*5301,HLA-Cw*0401	**NP**N**N**NA**A**T**V**	HLA-B*5301,HLA-Cw*0401	**NP**N**N**DS**A**I**V**	HLA-Cw*0401	55
**Spike**							
1	**ARDLICAQ**K	HLA-A1,HLA-Cw*0401	**ARDLICAQ**K	HLA-A1,HLA-Cw*0401	**ARDLICAQ**Y	HLA-A1,HLA-Cw*0401,HLA-B*2703	88
2	**DLLF**N**KVT**L	HLA-A2,HLA-A*0206,HLA-Cw*0401	**DLLF**N**KVT**L	HLA-A2,HLA-A*0206,HLA-Cw*0401	**DLLF**D**KVT**I	HLA-A3,HLA-Cw*0401	77
3	N**LNES**L**IDL**	HLA-Cw*0401	N**LNES**L**IDL**	HLA-Cw*0401	A**LNES**Y**IDL**	HLA-A*0203,HLA-Cw*0401	77
4	**Y**I**KWPWY**I**W**	HLA-A*0206,HLA-Cw*0401	**Y**I**KWPWY**V**W**	HLA-A*0206, HLA-Cw*0401	**Y**N**KWPWY**I**W**	HLA-A*0206,HLA-B*3501,HLA-B*5102,HLA-B*5103,HLA-B*5401, HLA-B7, HLA-Cw*0401,HLA-B35	77
5	**GFIAGL**I**A**I	HLA-B8,HLA-Cw*0401	**GFIAGL**I**A**I	HLA-B8,HLA-Cw*0401	**GFIAGL**V**A**L	HLA-B8,HLA-Cw*0401,HLA-B35	77
6	Q**Y**I**KWPWY**I	HLA-A*1101,HLA-A11,HLA-A24,HLA-A3,HLA-B8,HLA-Cw*0401,	Q**Y**I**KWPWY**V	HLA-A24, HLA-A*2402,HLA-B*5301,HLA-B8,HLA-Cw*0401	Y**Y**NK**WPWY**I	HLA-A*1101,HLA-A11,HLA-B8,HLA-Cw*0401,	66
7	**NQK**L**IAN**Q**F**	HLA-B27,HLA-Cw*0401,HLA-B*2703	**NQK**Q**IAN**Q**F**	HLA-B14,HLA-B27,HLA-Cw*0401	**NQK**L**IAN**K**F**	HLA-B27,HLA-Cw*0401,	66
8	V**VN**Q**NAQAL**	HLA-B*3501,HLA-B*51,HLA-B*0702,HLA-Cw*0401,HLA-B35	V**VN**Q**NAQAL**	HLA-B*3501,HLA-B*51,HLA-B*0702,HLA-Cw*0401,HLA-B35	A**VN**N**NAQAL**	HLA-B*51,HLA-Cw*0401,HLA-B35	66
9	A**YR**F**NG**I**G**V	HLA-A*2402,HLA-Cw*0401,HLA-B*2706,HLA-A*3301	A**YR**F**NG**I**G**V	HLA-A*2402,HLA-Cw*0401,HLA-B*2706,HLA-A*3301	F**YR**L**NG**V**G**I	HLA-A*1101,HLA-A11,HLA-Cw*0401,HLA-A*3301	55
10	D**FCG**K**G**Y**H**L	HLA-Cw*0401	D**FCG**K**G**YHL	HLA-Cw*0401	G**FCG**Q**G**T**H**I	HLA-A*1101,HLA-A11,HLA-B8,HLA-Cw*0401,	55
11	**APHGVVFLH**	HLA-A*2402,HLA-A*0301,HLA-Cw*0401	**APHGVVFLH**	HLA-A*2402,HLA-A*0301,HLA-Cw*0401,	**APNGLYFMH**	HLA-B*5102,HLA-B*5103,HLA-Cw*0401	55
12	QS**AP**H**G**VV**F**	HLA-Cw*0401,HLA-A*3301	QA**AP**H**G**VV**F**	HLA-Cw*0401,	VN**AP**N**G**LY**F**	HLA-B*5401,HLA-B*51,HLA-B*0702,HLA-Cw*0401	44
13	VV**F**L**HV**T**Y**V	HLA-B*51,HLA-Cw*0401	VV**F**LHVT**Y**V	HLA-B*51,HLA-Cw*0401	LY**F**M**HV**G**Y**Y	HLA-A1,HLA-A*2402,HLA-A3,HLA-B*51,HLA-Cw*0401,HLA-A*3301	44
14	QMA**YR**F**NG**I	HLA-Cw*0401,HLA-A*3301,HLA-A*6801	QMA**YR**F**NG**I	HLA-Cw*0401,HLA-A*3301,HLA-A*6801	SIF**YR**L**NG**V	HLA-B*51,HLA-Cw*0401	44

**Table 4:** Predicted B cell epitopes found to be conserved in E, N, M, and S protein sequences of SARS-CoV-2, SARS-CoV, and MERS-CoV. Conserved amino acids are in bold letters.

Conserved B cell epitopes in SARS-CoV-2, SARS-CoV, and MERS-CoV				
Envelope				
**№**	**SARS-CoV-2**	**SARS-CoV**	**MERS-CoV**	**Conservation (%)**
1	YS**FV**S**E**ET**G**TL**IVN**	YS**FV**S**E**ET**G**TL**IVN**	LP**FV**Q**E**RI**G**LF**IVN**	50
2	YS**FV**S**E**ET**G**TL**I**	YS**FV**S**E**ET**G**TL**I**	LP**FV**Q**E**RI**G**LF**I**	42
**Membrane**				
1	**S**M**WSFNPETN**I**LLN**	**S**M**WSFNPETN**I**LLN**	**S**W**WSFNPETN**C**LLN**	86
**Nucleocapsid**				
1	**T**A**SW**F**T**A**LTQHGK**ED**L**	**T**A**SW**F**T**A**LTQHGK**EE**L**	**T**V**SW**Y**T**G**LTQHGK**VP**L**	69
2	Y**N**VT**QAFG**R**RGP**EQT**Q**	Y**N**VT**QAFG**R**RGP**EQT**Q**	F**N**MV**QAFG**L**RGP**GDL**Q**	56
3	DQVI**LL**NKH**IDAYKTF**	DNVI**LL**NKH**IDAYKTF**	KWLE**LL**EQN**IDAYKTF**	56
4	T**GAIKLD**D**K**D**P**NFKDQ	H**GAIKLD**D**K**D**P**QFKDN	S**GAIKLD**P**K**N**P**NYNKW	50
**Spike**				
1	NE**V**A**K**N**LNES**L**IDL**Q**E**	NE**V**A**K**N**LNES**L**IDL**Q**E**	QQ**V**V**K**A**LNES**Y**IDL**K**E**	63
2	**ES**L**IDL**Q**ELG**K**Y**EQ**Y**I	**ES**L**IDL**Q**ELG**K**Y**EQ**Y**I	**ES**Y**IDL**K**ELG**N**Y**TY**Y**N	63
3	**CV**LG**QS**K**R**VD**FCG**K**G**Y	**CV**LG**QS**K**R**VD**FCG**K**G**Y	**CV**KA**QS**K**R**SG**FCG**Q**G**T	56
4	R**DL**I**C**A**Q**KFN**G**LT**VLP**	R**DL**I**C**A**Q**KFN**G**LT**VLP**	R**DL**I**C**A**Q**YVA**G**YK**VLP**	50
5	EAEV**Q**I**DRLI**T**GRL**QS	EAEV**Q**I**DRLI**T**GRL**QS	EQDA**Q**I**DRLI**N**GRL**TT	50

**Table 5:** List of immunogenic and non-toxic T and B cell epitopes from E, N, M, and S proteins. The table shows sequences of the shortlisted epitopes, their immunogenicity and toxicity score, and conservation (including level of conservation) between seven HCoVs and the three closely related SARS-CoV-2, SARS-CoV, and MERS-CoV as well as in three variants of SARS-CoV-2. The last column shows whether these epitopes have previously been experimentally validated or not.

SARS-CoV-2 Antigenic Non-Toxic CD8+ T and B Cell Epitopes							
T Cell Epitopes							
Envelope							
**No**	**Peptide**	**Immunogenecity (Vaxijen)**	**Toxicity (ToxinPred)**	**Conservation in 7 HCoVs (%)**	**Conservation in SARS-CoV-2, SARS-CoV, MERS-CoV (%)**	**Conservation in B.1.1.7, B.1.351, P.1 (%)**	**Studies Experimentally Validating the Epitopes**
1	S**LV**K**P**SF**Y**V	0.414	Non-toxin	-	44	100	[58,59]
**Membrane**							
1	S**YF**IA**S**F**RL**	0.4821	Non-toxin	55	66	100	[59,60,61]
2	AGD**SG**F**A**A**Y**	0.9095	Non-toxin	-	44	100	[61,62]
**Nucleocapsid**							
1	S**P**RWY**FYY**L	0.734	Non-toxin	44	77	100	[62,63]
2	**QASSRSSSR**	0.8294	Non-toxin	-	66	100	[59]
3	**VPINTNSSP**	0.4439	Non-toxin	-	66	88	[62]
**Spike**							
1	**DLLFNKVTL**	0.68	Non-toxin	-	77	100	[62,64,65]
2	N**LNES**L**IDL**	0.6827	Non-toxin	-	77	100	[58,59,64]
3	V**VN**Q**NAQAL**	0.4749	Non-toxin	-	66	100	[64]
4	A**YR**F**NG**I**G**V	1.2995	Non-toxin	-	55	100	[62,64,65]
5	VV**F**L**HV**T**Y**V	1.5122	Non-toxin	-	44	100	[62]
6	QMA**YR**F**NG**I	0.6803	Non-toxin	-	44	100	[62,65]
**B cell epitopes**							
**Envelope**							
**№**	**Peptide**	**Immunogenecity (Vaxijen)**	**Toxicity (ToxinPred)**	**Conservation in 7 HCoVs (%)**	**Conservation in SARS-CoV-2, SARS-CoV, MERS-CoV (%)**	**Conservation in B.1.1.7, B.1.351, P.1 (%)**	**Experimentally Validated Epitope**
1	YS**FV**S**E**ET**G**TL**IVN**	0.4532	Non-Toxin	-	50	100	[66]
2	YS**FV**S**E**ET**G**TL**I**	0.5014	Non-Toxin	-	42	100	[66]
**Nucleocapsid**							
1	**T**A**SW**F**T**A**LTQHGK**ED**L**	0.4149	Non-Toxin	-	69	100	-
2	Y**N**VT**QAFG**R**RGP**EQT**Q**	0.4899	Non-Toxin	-	56	100	-
3	T**GAIKLD**D**K**D**P**NFKDQ	1.8438	Non-Toxin	-	50	100	-
**Spike**							
1	**ES**L**IDL**Q**ELG**K**Y**EQ**Y**I	0.6105	Non-Toxin	-	63	100	-

**Table 6:** Docking results of the developed vaccine with human toll-like receptor 4 (TLR4). The table shows global binding energy as well as different forces contributing to the global energy.

Complex	Global Binding Energy	Energy Contributions			
		Attractive Van Der Waals (vdW)	Repulsive Van Der Waals (vdW)	Atomic Contact Energy (ACE)	Hydrogen Bond (HB)
Vaccine-TLR4	−47.76	−36.33	16.90	15.29	−0.42

## Data Availability

All datasets presented in this study are included in the article/Supplementary Materials.

## Supplementary Materials

The following are available online at https://www.mdpi.com/article/10.3390/vaccines9070702/s1, Figure S1: Multisubunit vaccine construct, Figure S2: Phylogenetic analysis between human coronaviruses, Figure S3: Conservation of envelope, membrane, nucleocapsid and spike protein sequences in seven HCoVs, Figure S4: Conservation of envelope, membrane, nucleocapsid, and spike protein sequences in SARS-CoV-2, SARS-CoV and MERS-CoV, Figure S5: Conservation of envelope, membrane, nucleocapsid, and spike protein sequence in 5000 SARS-CoV-2 sequences and in B.1.1.7, B.1.351, and P.1 variants of SARS-CoV-2, Table S1: Consensus sequences used to predict the epitopes for vaccine design, Table S2: List of all epitopes predicted with CTLpred.
