# Supplementary material for: Multi-Subunit SARS-CoV-2 Vaccine Design Using Evolutionarily Conserved T- and B- Cell Epitopes
Source: Vaccines (Basel). 2021 Jun 26;9(7):702. doi: 10.3390/vaccines9070702 (PMC8310312; doi:10.3390/vaccines9070702)
Supplement: Supplementary file 1 [file vaccines-09-00702-s001.zip › vaccines-1231099-supplementary.pdf]

A

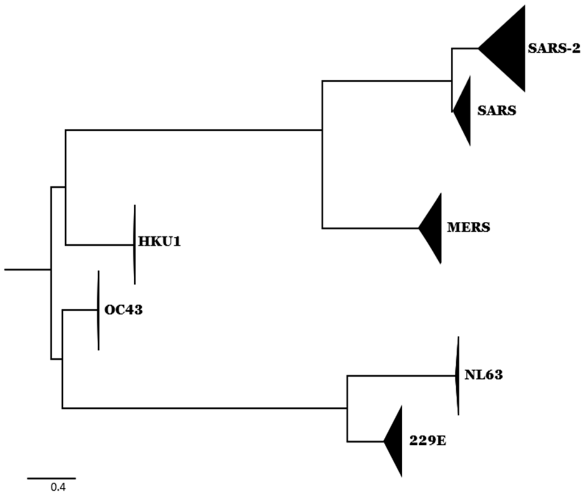

B

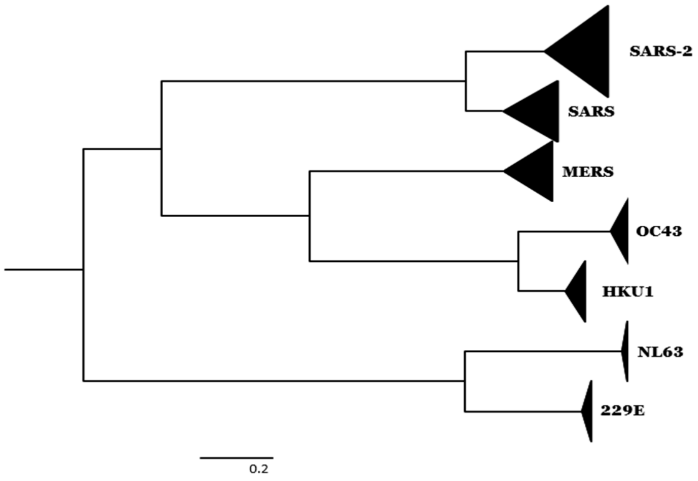

Supplementary Figure S1. Phylogenetic analysis between human coronaviruses: ML tree was constructed using A. envelope and B. membrane nucleotide sequences from human coronaviruses.

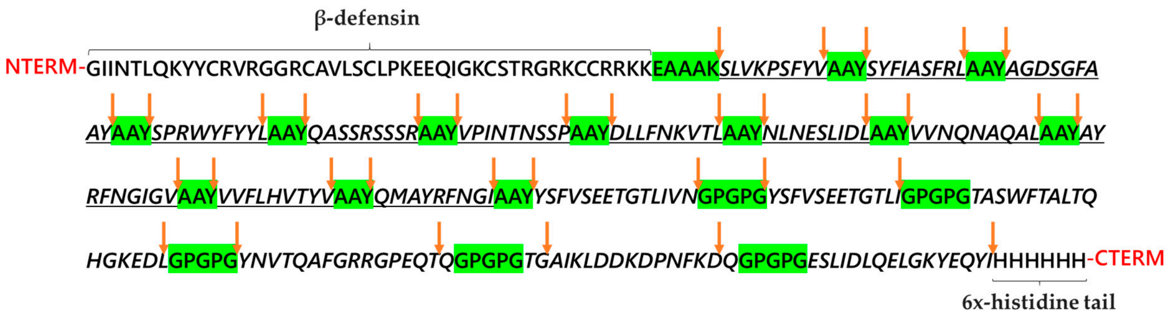

Supplementary Figure S2. Multisubunit vaccine construct: Figure shows different components of our vaccine construct, namely N-terminus (NTERM), β-defensin amino acid sequence, followed by EAAK, AAY and GPGPG linkers, epitopes and C-terminus (CTERM) 6x Histidine sequence. In between linkers, CD8 T-cell (Italics and underlined) and B-cells (italics) epitopes are shown. The arrows indicate proteasomal sites within the construct that may give rise to mature epitopes.

A.

Conservation of Envelope Protein Sequence in HCoVs

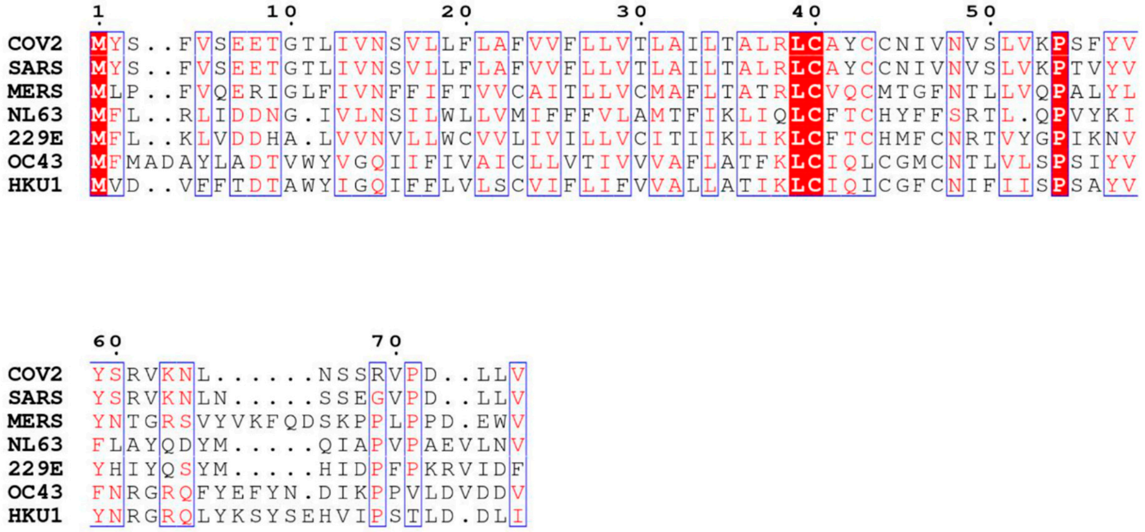

B. Conservation of Membrane Protein Sequence in HCoVs

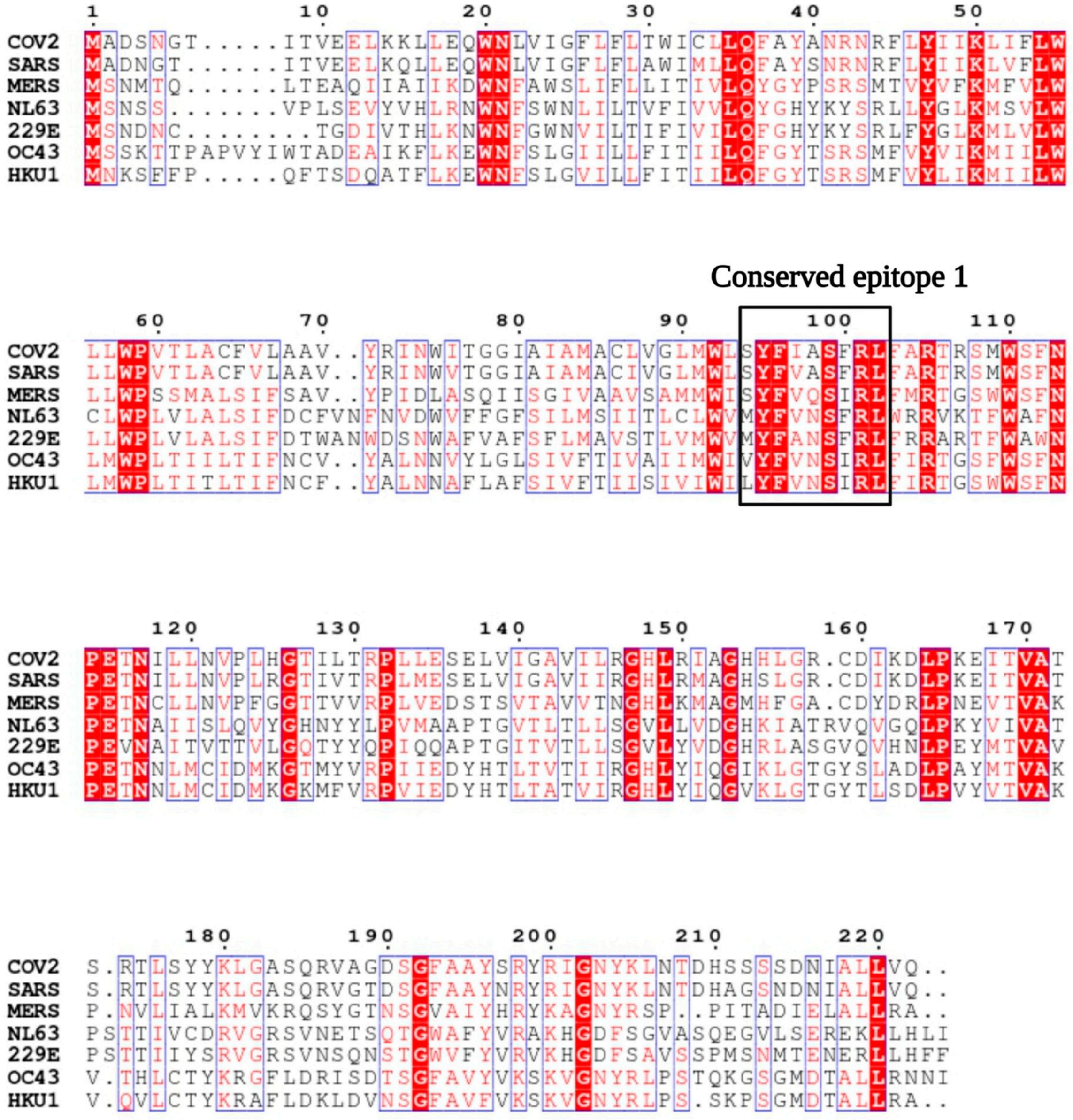

C. Conservation of Nucleocapsid Protein Sequence in HCoVs

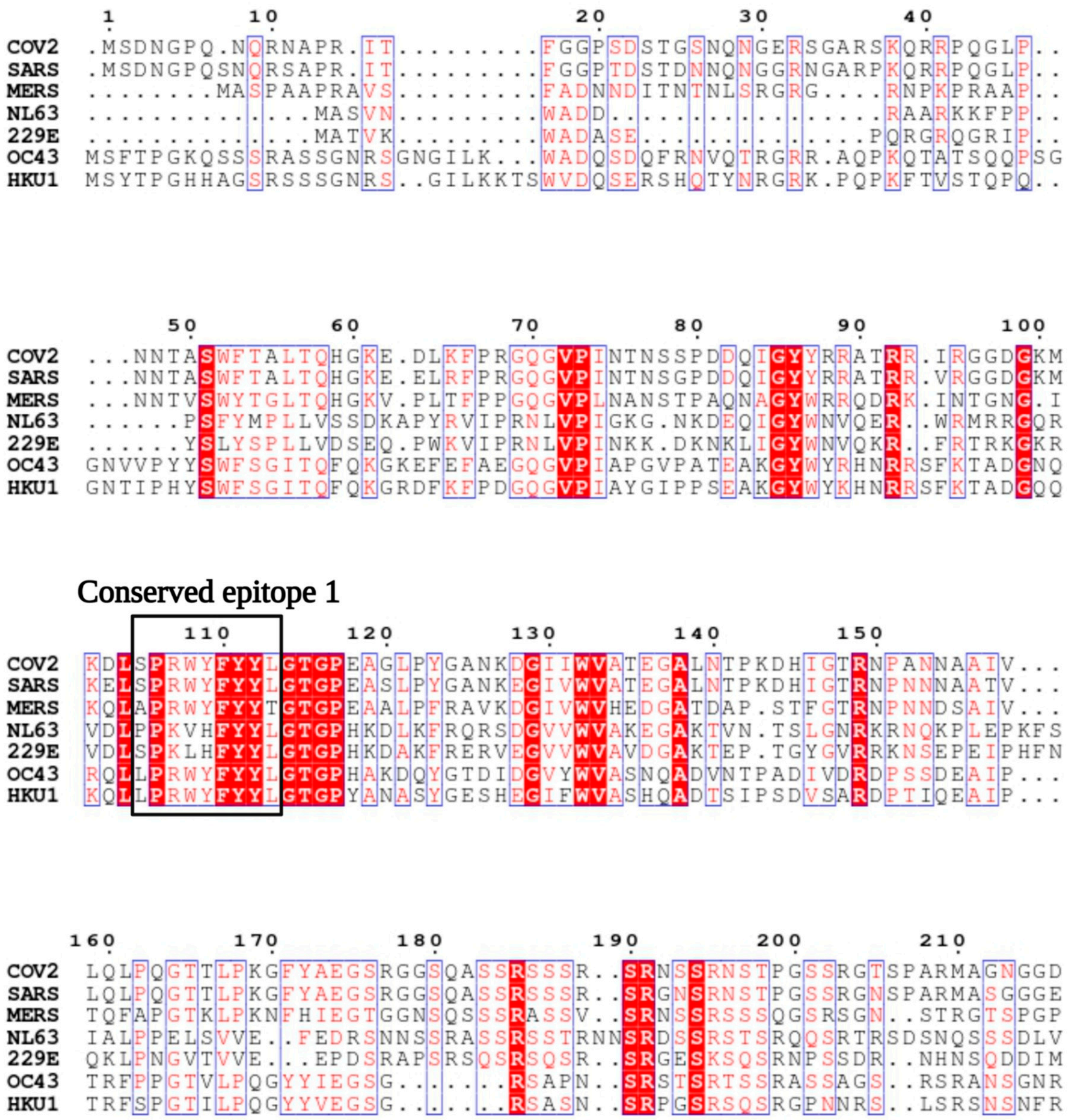

```
         220                 230       240                          250
COV2  AALALLL.........LDRLNQLESKMSGKGQQQQG.............QTVTKKSAAEA
SARS  TALALLL.........LDRLNQLESKVSGKGQQQQG.............QTVTKKSAAEA
MERS  SGIGAVGGDLLY....LDLLNRLQALESGKVKQSQP.............KVITKKDAAAA
NL63  AAVTLALKNLGFD...NQSKSPSSSGTSTPKKPNKP.............LSQPRADKPSQ
229E  KAVAAALKSLGFDKPQEKDKKSAKTGTPKPSRNQSPASSQSAAKILARSQSSETKEQKHE
OC43  TPTSGVTPDMADQ...IASLVLAKLGKDATKPQQVT.............KHTAKEVRQKI
HKU1  HSDSIVKPDMADE...IASLVLAKLGKD.SKPQQVT.............KQNAKEIRHKI

          260         270       280       290       300       310
COV2  SKKPRQKRTATKA..YNVTQAFGRRGPEQTQGNFGDQELIRQGTDYKHWPQIAQFAPSAS
SARS  SKKPRQKRTATKQ..YNVTQAFGRRGPEQTQGNFGDQDLIRQGTDYKHWPQIAQFAPSAS
MERS  KNKMRHKRTSTKS..FNMVQAFGLRGPGDLQGNFGDLQLNKLGTEDPRWPQIAELAPTAS
NL63  LKKPRWKRVPTRE..ENVIQCFGPRDFNH...NMGDSDLVQNGVDAKGFPQLAELIPNQA
229E  MQKPRWKRQPNDDVTSNVTQCFGPRDLDH...NFGSAGVVANGVKAKGYPQFAELVPSTA
OC43  LNKPRQKRSPNKQ..CTVQQCFGKRGPNQ...NFGGGEMLKLGTSDPQFPILAELAPTAG
HKU1  LMKPRQKRTPNKF..CNVQQCFGKRGPLQ...NFGN.EMLKLGTNDPQFPILAELAPTPG

           320                  330       340       350       360
COV2  AFFGMSRIGMEVTPS...........GTWLTYTGAIKLDDKDPNFKDQVILLNKHIDAYK
SARS  AFFGMSRIGMEVTPS...........GTWLTYHGAIKLDDKDPQFKDNVILLNKHIDAYK
MERS  AFMGMSQFKLTHQNN.....DDHGNPVYFLRYSGAIKLDPKNPNYNKWLELLEQNIDAYK
NL63  ALFFDSEVSTDEVGD...........NVQITYTYKMLVAKDNKNLPKFIEQIS.......
229E  AMLFDSHIVSKESGN...........TVVLTFTTRVTVPKDHPHLGKFLEELN.......
OC43  AFFFGSRLELAKVQNLSGNPDEPQKDVYELRYNGAIRFDSTLSGFETIMKVLSENLNAYQ
HKU1  AFFFGSKLELFKRDS...DADSPSKDTFELRYSGSIRFDSTLPGFETIMKVLKENLDAYV

                        370                                380
COV2  .........TFPPTDFSKQLQQS........................MSSADSTQA....
SARS  .........TFPPTEPKKDKKKKTDEAQPLPQRQKKQPTVTLLPAADMDDFSRQLQNSMS
MERS  .........TFPKKEKKQKAPKE..ESTDQMSEPPKEQRVQG....SITQRTRTRPSVQP
NL63  .........AFTKPSSIKEMQSQSSHVVQNTVLNASIPESKP.....LADDDSAIIEIVN
229E  .........AFTREMQQQPLLNPSALEFNPSQTSPATVE..P.....VRDEVSIETDIID
OC43  QQDGMM...NMSPKPQRQRGHKNGQGENDNISVAVPKSRVQQNKSIELTAEDISLLKKMD
HKU1  NSNQNTVSGSLSPKPQRKRGVKQSPESFDSLNLSADTQHISN....DFTPEDHSLLATLD

COV2  ..........
SARS  GASADSTQA.
MERS  GPMIDVNTD.
NL63  EVLH......
229E  EVN.......
OC43  EPYTEDTSEI
HKU1  DPYVEDSVA.
```

D. Conservation of Spike Protein Sequence in HCoVs

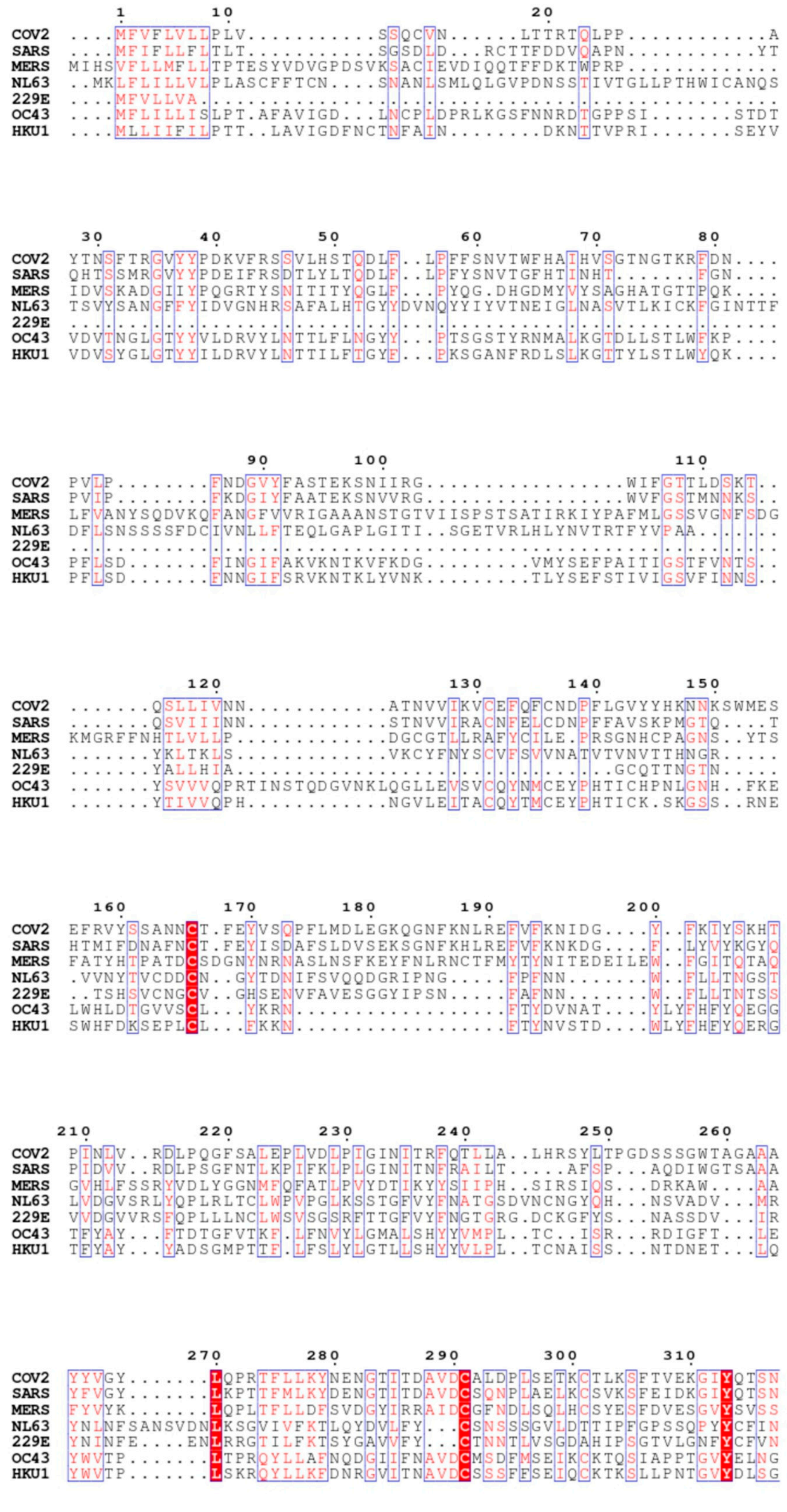

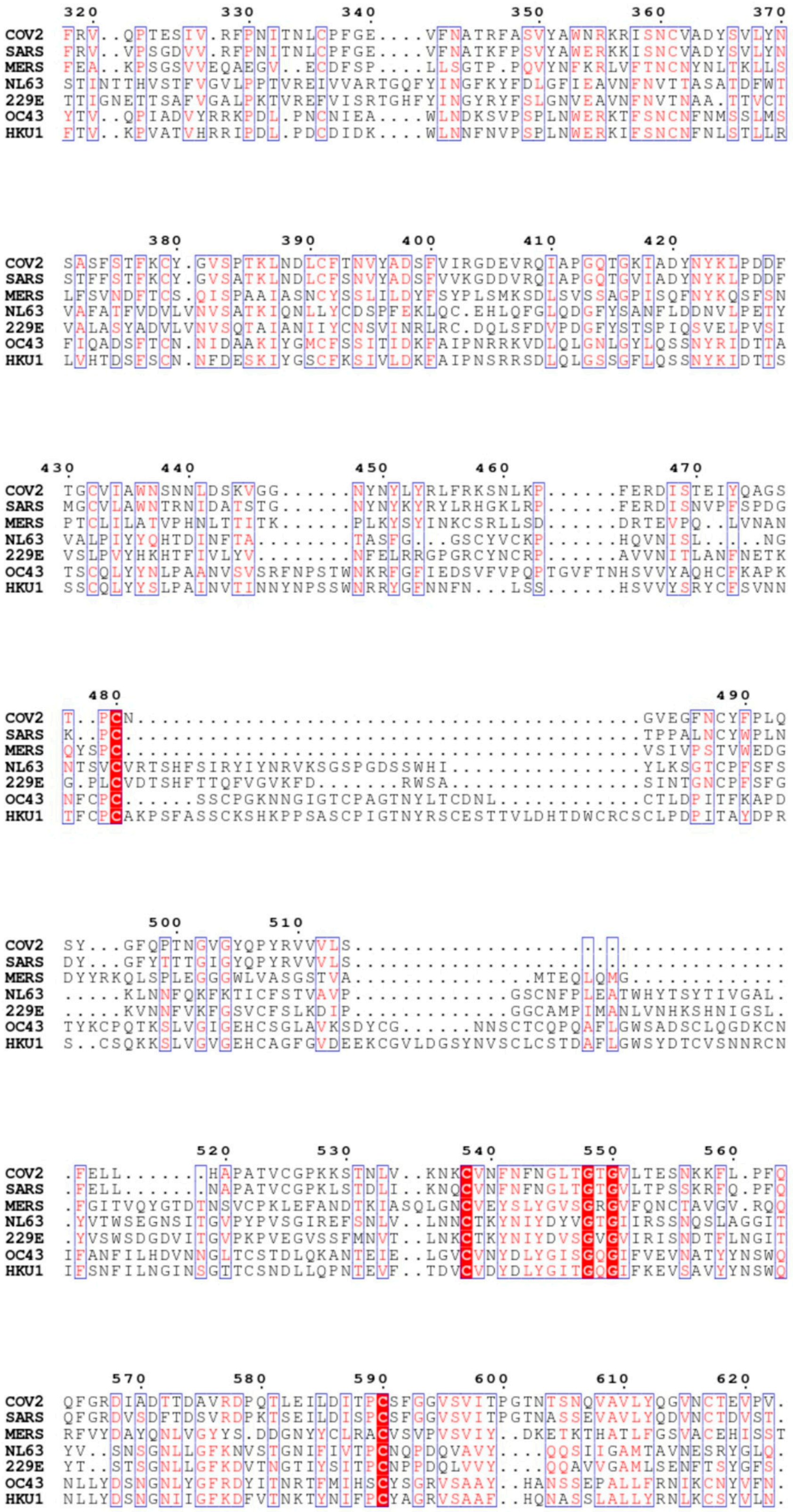
320 330 340 350 360 370
COV2 FRV..QPTESIV.RFPNITNLCPFGE....VFNATRFASVYAWNRKRISNCVADYSVLYN
SARS FRV..VPSGDVV.RFPNITNLCPFGE....VFNATKFPSVYAWERKKISNCVADYSVLYN
MERS FEA..KPSGSVVEQAEGV..ECDFSP....LLSGTP.PQVYNFKRLVFTNCNYNLTKLLS
NL63 STINTTHVSTFVGVLPPTVREIVVARTGQFYINGFKYFDLGFIEAVNFNVTTASATDFWT
229E TTIGNETTSAFVGALPKTVREFVISRTGHFYINGYRYFSLGNVEAVNFNVTNAA.TTVCT
OC43 YTV..QPIADVYRRKPDL.PNCNIEA....WLNDKSVPSPLNWERKTFSNCNFNMSSLMS
HKU1 FTV..KPVATVHRRIPDL.PDCDIDK....WLNNFNVPSPLNWERKIFSNCNFNLSTLLR
380 390 400 410 420
COV2 SASFSTFKCY.GVSPTKLNDLCFTNVYADSFVIRGDEVRQIAPGQTGKIADYNYKLPDDF
SARS STFFSTFKCY.GVSATKLNDLCFSNVYADSFVVKGDDVRQIAPGQTGVIADYNYKLPDDF
MERS LFSVNDFTCS.QISPAAIASNCYSSLILDYFSYPLSMKSDLSVSSAGPISQFNYKQSFSN
NL63 VAFATFVDVLVNVSATKIQNLLYCDSPFEKLQC.EHLQFGLQDGFYSANFLDDNVLPETY
229E VALASYADVLVNVSQTAIANIIYCNSVINRLRC.DQLSFDVPDGFYSTSPIQSVELPVSI
OC43 FIQADSFTCN.NIDAAKIYGMCFSSITIDKFAIPNRRKVDLQLGNLGYLQSSNYRIDTTA
HKU1 LVHTDSFSCN.NFDESKIYGSCFKSIVLDKFAIPNSRRSDLQLGSSGFLQSSNYKIDTTS
430 440 450 460 470
COV2 TGCVIAWNSNNLDSKVGG......NYNYLYRLFRKSNLKP......FERDISTEIYQAGS
SARS MGCVLAWNTRNIDATSTG......NYNYKYRYLRHGKLRP......FERDISNVPFSPDG
MERS PTCLILATVPHNLTTITK......PLKYSYINKCSRLLSD......DRTEVPQ..LVNAN
NL63 VALPIYYQHTDINFTA........TASFG...GSCYVCKP......HQVNISL.....NG
229E VSLPVYHKHTFIVLYV........NFELRRGPGRCYNCRP......AVVNITLANFNETK
OC43 TSCQLYYNLPAANVSVSRFNPSTWNKRFGFIEDSVFVPQPTGVFTNHSVVYAQHCFKAPK
HKU1 SSCQLYYSLPAINVTINNYNPSSWNRRYGFNNFN...LSS......HSVVYSRYCFSVNN
480 490
COV2 T..PCN..GVEGFNCYFPLQ
SARS K..PC...TPPALNCYWPLN
MERS QYSPC...VSIVPSTVWEDG
NL63 NTSVCVRTSHFSIRYIYNRVKSGSPGDSSWHI................YLKSGTCPFSFS
229E G.PLCVDTSHFTTQFVGVKFD.......RWSA................SINTGNCPFSFG
OC43 NFCPC......SSCPGKNNGIGTCPAGTNYLTCDNL............CTLDPITFKAPD
HKU1 TFCPCAKPSFASSCKSHKPPSASCPIGTNYRSCESTTVLDHTDWCRCSCLPDPITAYDPR
500 510
COV2 SY...GFQPTNGVGYQPYRVVVLS....................................
SARS DY...GFYTTTGIGYQPYRVVVLS....................................
MERS DYYRKQLSPLEGGGWLVASGSTVA...............MTEQLQMG.............
NL63 .....KLNNFQKFKTICFSTVAVP.............GSCNFPLEATWHYTSYTIVGAL.
229E .....KVNNFVKFGSVCFSLKDIP.............GGCAMPIMANLVNHKSHNIGSL.
OC43 TYKCPQTKSLVGIGEHCSGLAVKSDYCG......NNSCTCQPQAFLGWSADSCLQGDKCN
HKU1 S..CSQKKSLVGVGEHCAGFGVDEEKCGVLDGSYNVSCLCSTDAFLGWSYDTCVSNNRCN
520 530 540 550 560
COV2 .FELL.......HAPATVCGPKKSTNLV..KNKCVNFNFNGLTGTGVLTESNKKFL.PFQ
SARS .FELL.......NAPATVCGPKLSTDLI..KNQCVNFNFNGLTGTGVLTPSSKRFQ.PFQ
MERS .FGITVQYGTDTNSVCPKLEFANDTKIASQLGNCVEYSLYGVSGRGVFQNCTAVGV.RQQ
NL63 .YVTWSEGNSITGVPYPVSGIREFSNLV..LNNCTKYNIYDYVGTGIIRSSNQSLAGGIT
229E .YVSWSDGDVITGVPKPVEGVSSFMNVT..LNKCTKYNIYDVSGVGVIRISNDTFLNGIT
OC43 IFANFILHDVNNGLTCSTDLQKANTEIE..LGVCVNYDLYGISGQGIFVEVNATYYNSWQ
HKU1 IFSNFILNGINSGTTCSNDLLQPNTEVF..TDVCVDYDLYGITGQGIFKEVSAVYYNSWQ
570 580 590 600 610 620
COV2 QFGRDIADTTDAVRDPQTLEILDITPCSFGGVSVITPGTNTSNQVAVLYQGVNCTEVPV.
SARS QFGRDVSDFTDSVRDPKTSEILDISPCSFGGVSVITPGTNASSEVAVLYQDVNCTDVST.
MERS RFVYDAYQNLVGYYS.DDGNYYCLRACVSVPVSVIY..DKETKTHATLFGSVACEHISST
NL63 YV..SNSGNLLGFKNVSTGNIFIVTPCNQPDQVAVY....QQSIIGAMTAVNESRYGLQ.
229E YT..STSGNLLGFKDVTNGTIYSITPCNPPDQLVVY....QQAVVGAMLSENFTSYGFS.
OC43 NLLYDSNGNLYGFRDYITNRTFMIHSCYSGRVSAAY..HANSSEPALLFRNIKCNYVFN.
HKU1 NLLYDSNGNIIGFKDFVTNKTYNIFPCYAGRVSAAF..HQNASSLALLYRNLKCSYVLN.

```
              630       640       650       660       670
COV2  .AIHADQLTPTWRVYSTGSNVFQTRAGCLIGAEHVNNS..YECDIPIGAGICASYQTQT.
SARS  .AIHADQLTPAWRIYSTGNNVFQTQAGCLIGAEHVDTS..YECDIPIGAGICASYHTVS.
MERS  MSQYSRSTRSMLKRRDSTYGPLQTPVGCVLGLVNSSLF.VEDCKLPLGQSLCALPDTPST
NL63  ...NLLQLPNFYYVSNGG.......NNCTTAVMTYSNF...........GICADGSLIP.
229E  ...NVVEMPKFFYASNGT.......YNCTDAVLTYSSF...........GVCADGSIIA.
OC43  .NSLTRQLQPINYSFDSY.......LGCVVNAYNSTAISVQTCDLTVGSGYCVDY.....
HKU1  ..NISLATQPYFDSY..........LGCVFNADNLTDYSVSSCALRMGSGFCVDYNSPSS
```

```
      680       690       700       710                720
COV2  NSPRRARSVASQSIIAYTMSLGAENSVAYSNNS.........IAIPTNFTISVTTEILPV
SARS  ....LLRSTSQKSIVAYTMSLGADSSIAYSNNT.........IAIPTNFSISITTEVMPV
MERS  LTPRSVR.SVPGEMRLASIAFNHPIQVDQLNSS......YFKLSIPTNFSFGVTQEYIQT
NL63  VRPRNSSDNGISAIITAN........................LSIPSNWTTSVQVEYLQI
229E  VQPRNVSYDSVSAIVTAN........................LSIPSNWTTSVQVEYLQI
OC43  SKNRRSRRAITTGYRFTNF...EPFTVNSVNDSLEPVGGLYEIQIPSEFTIGNMEEFIQT
HKU1  SSSRRKRRSISASYRFVTF...EPFNVSFVNDSIESVGGLYEIKIPTNFTIVGQEEFIQT
```

```
     730       740       750       760       770           780
COV2  SMTKTSVDCTMYICGDSTECSNLLLQYGSFCTQLNRALTGIAVEQDKN....TQEVFAQV
SARS  SMAKTSVDCNMYICGDSTECANLLLQYGSFCTQLNRALSGIAAEQDRN....TREVFAQV
MERS  TIQKVTVDCKQYVCNGFQKCEQLLREYGQFCSKINQALHGANLRQDDS....VRNLFASV
NL63  TSTPIVVDCATYVCNGNPRCKNLLKQYTSACKTIEDALRLSAHLETND....VSSMLTFD
229E  TSTPIVVDCSTYVCNGNVRCVELLKQYTSACKTIEDALRNSAMLESAD....VSEMLTFD
OC43  SSPKVTIDCAAFVCGDYAACKLQLVEYGSFCDNINAILTEVNELLDTTQLQVANSLMNGV
HKU1  NSPKVTIDCSLFVCSNYAACHDLLSEYGTFCDNINSILDEVNGLLDTTQLHVADTLMQGV
```

```
         790         800           810       820       830
COV2  KQIYKTPPIKDFG.G.FNFSQILP....DPSKPSKRSFIEDLLFNKVTLADAGFIKQ.YG
SARS  KQMYKTPTLKYFG.G.FNFSQILP....DPLKPTKRSFIEDLLFNKVTLADAGFMKQ.YG
MERS  KSSQSSPIIPGFG.GDFNLTLLEPVSISTGSR.SARSAIEDLLFDKVTIADPGYMQG.YD
NL63  SNAFSLANVTSFG.D.YNLSSVLPQRNIHSSRIAGRSALEDLLFSKVVTSGLGTVDVDYK
229E  KKAFTLANVSSFG.D.YNLSSVIPSLPRSGSRVAGRSAIEDILFSKLVTSGLGTVDADYK
OC43  TLSTKLKDGVNFNVDDINFSPVLGCLGSECSKASSRSAIEDLLFDKVKLSDVGFVEA.YN
HKU1  TLSSNLNTNLHFDVDNINFKSLVGCLGPHCGS.SSRSFFEDLLFDKVKLSDVGFVEA.YN
```

```
      840         850       860       870       880       890
COV2  DCL..GDIAARDLICAQKFNGLTVLPPLLTDEMIAQYTSALLAGTITSGWTFGAGAALQI
SARS  ECL..GDINARDLICAQKFNGLTVLPPLLTDDMIAAYTAALVSGTATAGWTFGAGAALQI
MERS  DCMQQGPASARDLICAQYVAGYKVLPPLMDVNMEAAYTSSLLGSIAGVGWTAGLSSFAAI
NL63  SCT..KGLSIADLACAQYYNGIMVLPGVADAERMAMYTGSLIGGMVLGGLTSA....AAI
229E  KCT..KGLSIADLACAQYYNGIMVLPGVADAERMAMYTGSLIGGIALGGLTSA....ASI
OC43  NCT..GGAEIRDLICVQSYKGIKVLPPLLSENQISGYTLAATSASLFPPWTAA....AGV
HKU1  NCT..GGSEIRDLLCVQSFNGIKVLPPILSESQISGYTTAATVAAMFPPWSAA....AGI
```

```
        900       910       920       930       940
COV2  PFAMQMAYRFNGIGVTQNVLYENQKLIANQFNSAIGKIQDSLSSTAS.............
SARS  PFAMQMAYRFNGIGVTQNVLYENQKQIANQFNKAISQIQESLTTTST.............
MERS  PFAQSIFYRLNGVGITQQVLSENQKLIANKFNQALGAMQTGFTTTNE.............
NL63  PFSLALQARLNYVALQTDVLQENQKILAASFNKAINNIVASFSSVNDAITQTAEAIHTVT
229E  PFSLAIQSRLNYVALQTDVLQENQKILAASFNKAMTNIVDAFTGVNDAITQTSQALQTVA
OC43  PFYLNVQYRINGLGVTMDVLSQNQKLIANAFNNALHAIQQGFDATNS.............
HKU1  PFSLNVQYRINGLGVTMDVLNKNQKLIATAFNNALLSIQNGFSATNS.............
```

```
             950       960       970       980       990       1000
COV2  .ALGKLQDVVNQNAQALNTLVKQLSSNFGAISSVLNDILSRLDKVEAEVQIDRLITGRLQ
SARS  .ALGKLQDVVNQNAQALNTLVKQLSSNFGAISSVLNDILSRLDKVEAEVQIDRLITGRLQ
MERS  .AFRKVQDAVNNNAQALSKLASELSNTFGAISASIGDIIQRLDVLEQDAQIDRLINGRLT
NL63  IALNKIQDVVNQQGSALNHLTSQLRHNFQAISNSIQAIYDRLDSIQADQQVDRLITGRLA
229E  TALNKIQDVVNQQGNSLNHLTSQLRQNFQAISSSIQAIYDRLDIIQADQQVDRLITGRLA
OC43  .ALVKIQAVVNANAEALNNLLQQLSNRFGAISASLQEILSRLDALEAEAQIDRLINGRLT
HKU1  .ALAKIQSVVNSNAQALNSLLQQLFNKFGAISSSLQEILSRLDALEAQVQIDRLINGRLT
```

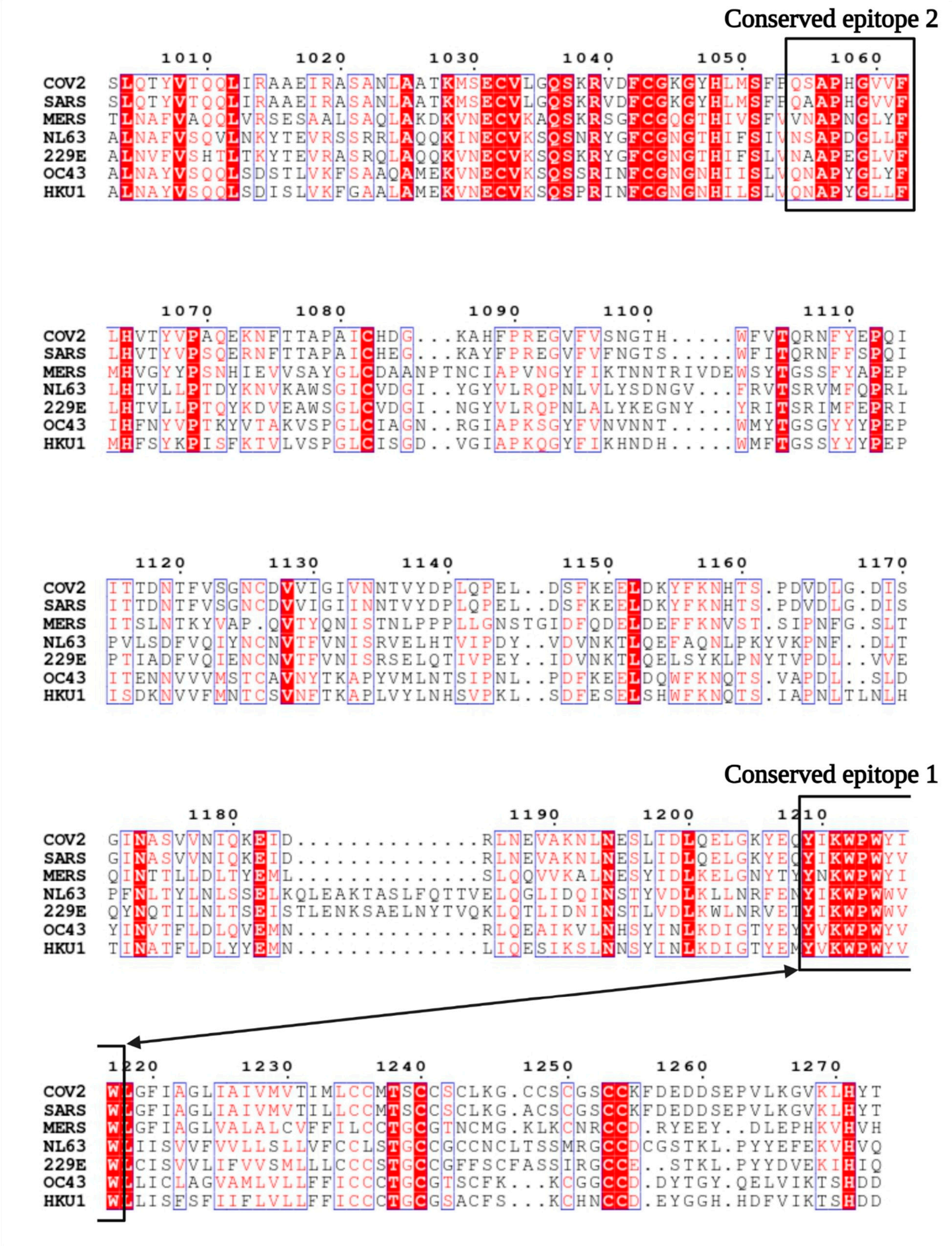
Conserved epitope 2
1010 1020 1030 1040 1050 1060
COV2 SLQTYVTQQLIRAAEIRASANLAATKMSECVLGQSKRVDFCGKGYHLMSFPQSAPHGVVF
SARS SLQTYVTQQLIRAAEIRASANLAATKMSECVLGQSKRVDFCGKGYHLMSFPQAAPHGVVF
MERS TLNAFVAQQLVRSESAALSAQLAKDKVNECVKAQSKRSGFCGQGTHIVSFVVNAPNGLYF
NL63 ALNAFVSQVLNKYTEVRSSRRLAQQKINECVKSQSNRYGFCGNGTHIFSIVNSAPDGLLF
229E ALNVFVSHTLTKYTEVRASRQLAQQKVNECVKSQSKRYGFCGNGTHIFSLVNAAPEGLVF
OC43 ALNAYVSQQLSDSTLVKFSAAQAMEKVNECVKSQSSRINFCGNGNHIISLVQNAPYGLYF
HKU1 ALNAYVSQQLSDISLVKFGAALAMEKVNECVKSQSPRINFCGNGNHILSLVQNAPYGLLF
1070 1080 1090 1100 1110
COV2 LHVTYVPAQEKNFTTAPAICHDG...KAHFPREGVFVSNGTH.....WFVTQRNFYEPQI
SARS LHVTYVPSQERNFTTAPAICHEG...KAYFPREGVFVFNGTS.....WFITQRNFFSPQI
MERS MHVGYYPSNHIEVVSAYGLCDAANPTNCIAPVNGYFIKTNNTRIVDEWSYTGSSFYAPEP
NL63 LHTVLLPTDYKNVKAWSGICVDGI..YGYVLRQPNLVLYSDNGV...FRVTSRVMFQPRL
229E LHTVLLPTQYKDVEAWSGLCVDGI..NGYVLRQPNLALYKEGNY...YRITSRIMFEPRI
OC43 IHFNYVPTKYVTAKVSPGLCIAGN..RGIAPKSGYFVNVNNT.....WMYTGSGYYYPEP
HKU1 MHFSYKPISFKTVLVSPGLCISGD..VGIAPKQGYFIKHNDH.....WMFTGSSYYYPEP
1120 1130 1140 1150 1160 1170
COV2 ITTDNTFVSGNCDVVIGIVNNTVYDPLQPEL..DSFKEELDKYFKNHTS.PDVDLG.DIS
SARS ITTDNTFVSGNCDVVIGIINNTVYDPLQPEL..DSFKEELDKYFKNHTS.PDVDLG.DIS
MERS ITSLNTKYVAP.QVTYQNISTNLPPPLLGNSTGIDFQDELDEFFKNVST.SIPNFG.SLT
NL63 PVLSDFVQIYNCNVTFVNISRVELHTVIPDY..VDVNKTLQEFAQNLPKYVKPNF..DLT
229E PTIADFVQIENCNVTFVNISRSELQTIVPEY..IDVNKTLQELSYKLPNYTVPDL..VVE
OC43 ITENNVVVMSTCAVNYTKAPYVMLNTSIPNL..PDFKEELDQWFKNQTS.VAPDL..SLD
HKU1 ISDKNVVFMNTCSVNFTKAPLVYLNHSVPKL..SDFESELSHWFKNQTS.IAPNLTLNLH
Conserved epitope 1
1180 1190 1200 1210
COV2 GINASVVNIQKEID..............RLNEVAKNLNESLIDLQELGKYEQYIKWPWYI
SARS GINASVVNIQKEID..............RLNEVAKNLNESLIDLQELGKYEQYIKWPWYV
MERS QINTTLLDLTYEML..............SLQQVVKALNESYIDLKELGNYTYYNKWPWYI
NL63 PFNLTYLNLSSELKQLEAKTASLFQTTVELQGLIDQINSTYVDLKLLNRFENYIKWPWWV
229E QYNQTILNLTSEISTLENKSAELNYTVQKLQTLIDNINSTLVDLKWLNRVETYIKWPWWV
OC43 YINVTFLDLQVEMN..............RLQEAIKVLNHSYINLKDIGTYEYYVKWPWYV
HKU1 TINATFLDLYYEMN..............LIQESIKSLNNSYINLKDIGTYEMYVKWPWYV
1220 1230 1240 1250 1260 1270
COV2 WLGFIAGLIAIVMVTIMLCCMTSCCSCLKG.CCSCGSCCKFDEDDSEPVLKGVKLHYT
SARS WLGFIAGLIAIVMVTILLCCMTSCCSCLKG.ACSCGSCCKFDEDDSEPVLKGVKLHYT
MERS WLGFIAGLVALALCVFFILCCTGCGTNCMG.KLKCNRCCD.RYEEY..DLEPHKVHVH
NL63 WLIISVVFVVLLSLLVFCCLSTGCCGCCNCLTSSMRGCCDCGSTKL.PYYEFEKVHVQ
229E WLCISVVLIFVVSMLLLCCCSTGCCGFFSCFASSIRGCCE..STKL.PYYDVEKIHIQ
OC43 WLLICLAGVAMLVLLFFICCCTGCGTSCFK...KCGGCCD.DYTGY.QELVIKTSHDD
HKU1 WLLISFSFIIFLVLLFFICCCTGCGSACFS...KCHNCCD.EYGGH.HDFVIKTSHDD

Supplementary figure S3. Conservation of envelope, membrane, nucleocapsid and spike protein sequences in seven HCoVs. Consensus sequences of each protein were aligned and conserved regions were identified using ESPript 3.x. Conserved amino acids between HCoVs are colored in red. A. For envelope protein, no conserved epitopes were found for envelope protein among seven HCoVs. B. For membrane protein, 1 CD8+ T cell epitope (Conserved epitope 1) was found to be conserved among seven HCoVs with ~55% conservancy, which is shown in the black square. C. For nucleocapsid protein, 1 CD8+ T cell epitope (Conserved epitope 1) was found to be conserved among seven HCoVs with ~44% conservancy, which is shown in the black square. D. For spike protein, 2 CD8+ T cell epitopes (Conserved epitope 1 and Conserved epitope 2) were found to be conserved among seven HCoVs, which are shown in the black squares. Epitope 1 is ~66% conserved, while epitope 2 is ~44% conserved. Epitopes are numbered with order of the highest conservancy.

A. Conservation of Envelope Protein Sequence in SARS-CoV-2, SARS-CoV, MERS-CoV

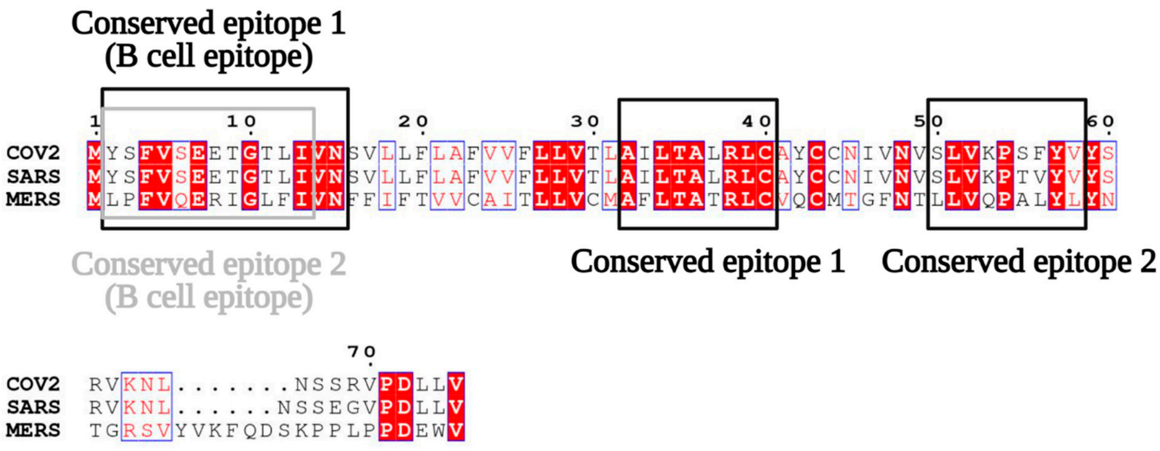

B. Conservation of Membrane Protein Sequence in SARS-COV-2, SARS-COV, MERS

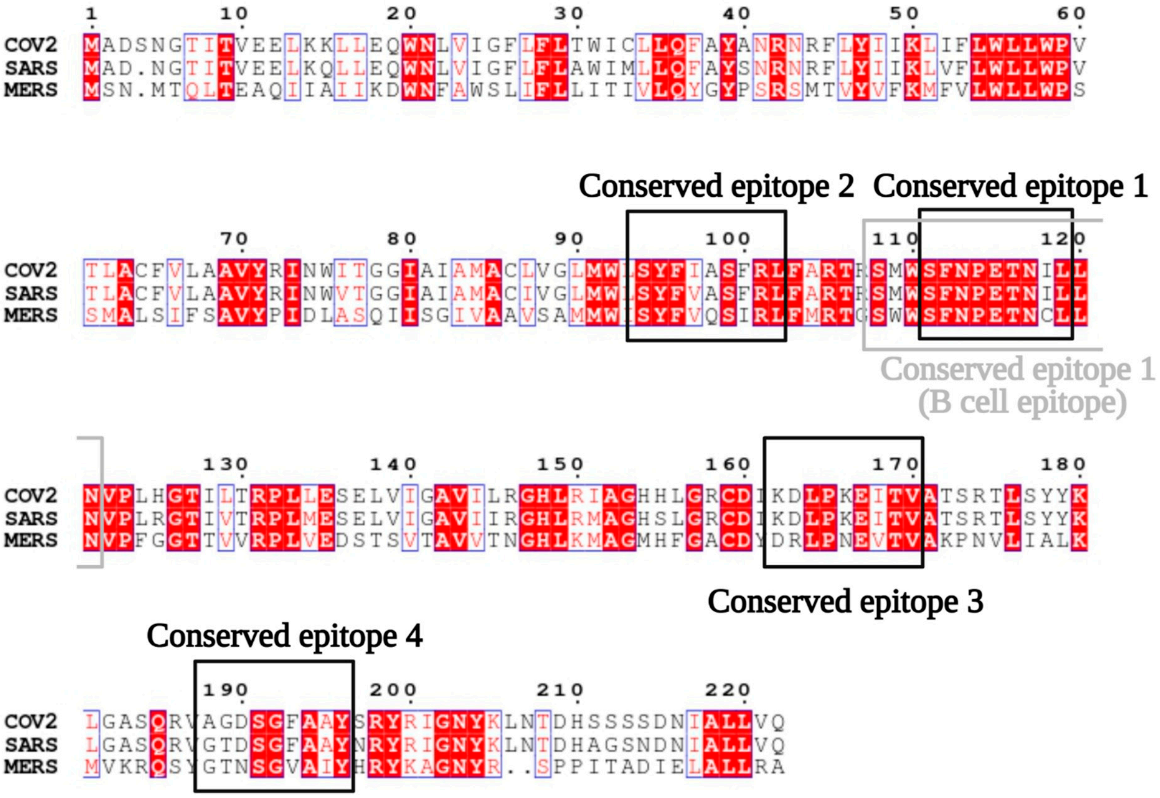

C. Conservation of Nucleocapsid Protein Sequence in SARS-CoV-2, SARS-CoV, MERS-CoV

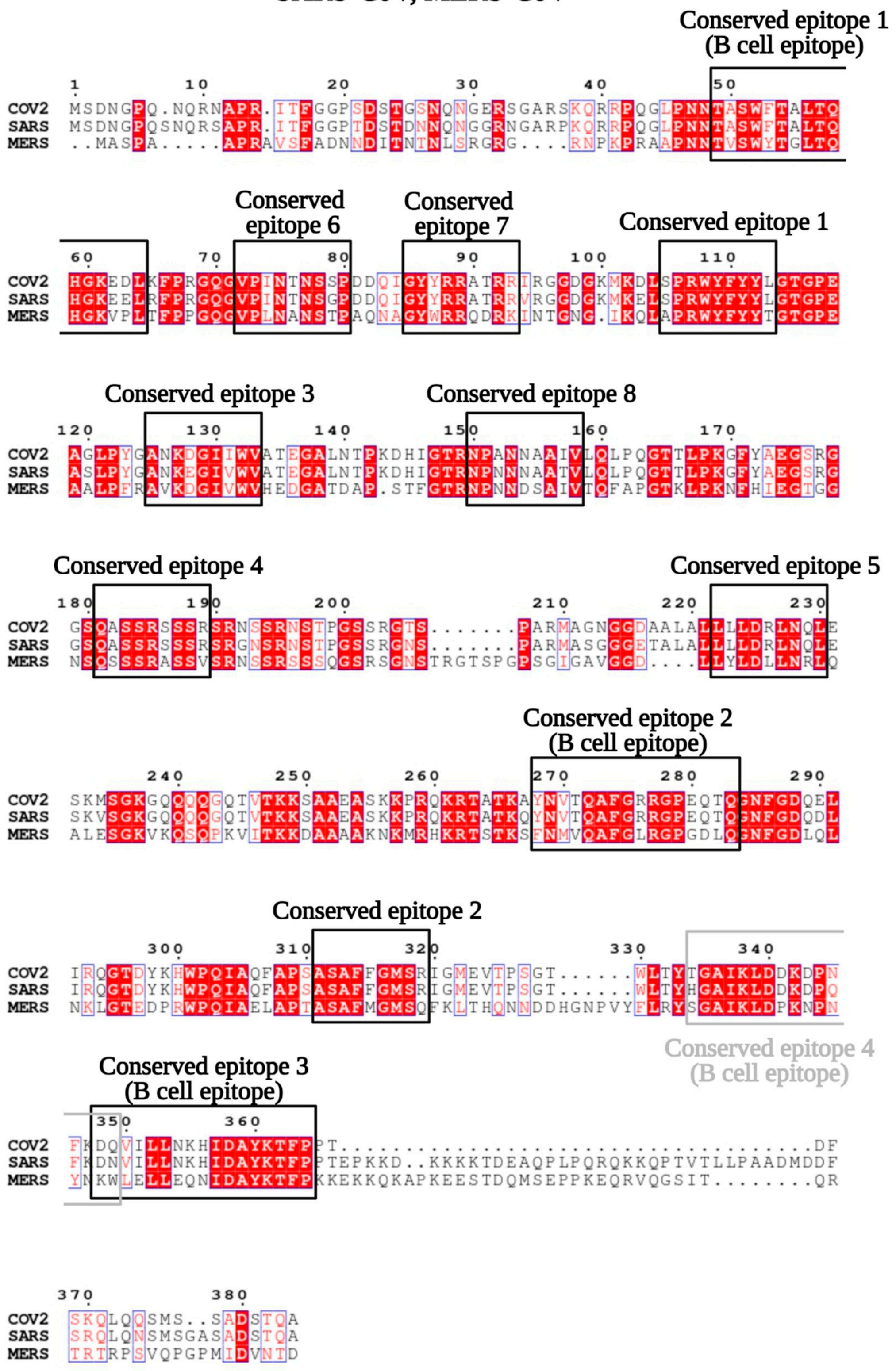

D. Conservation of Spike Protein Sequence in SARS-COV-2, SARS-COV, MERS

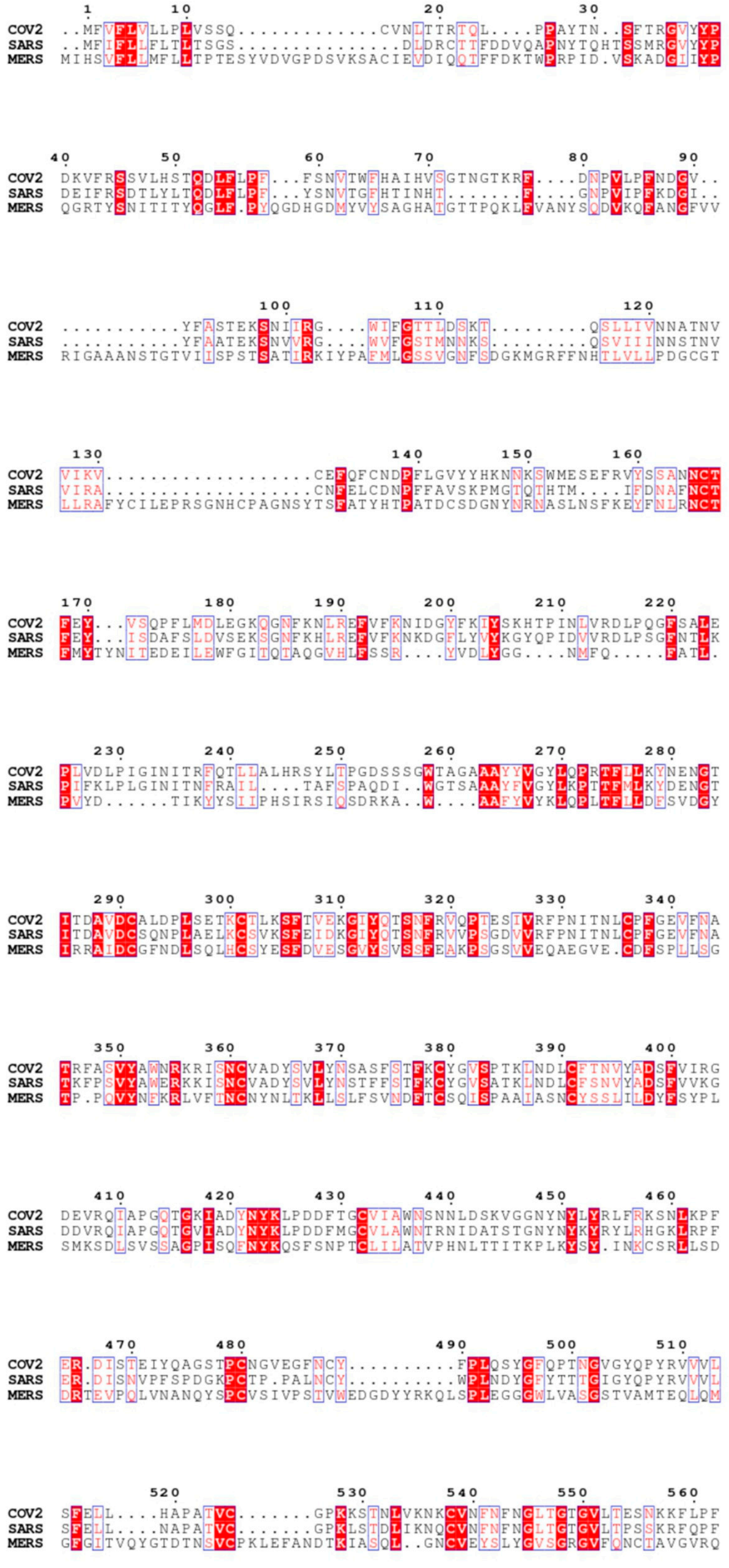

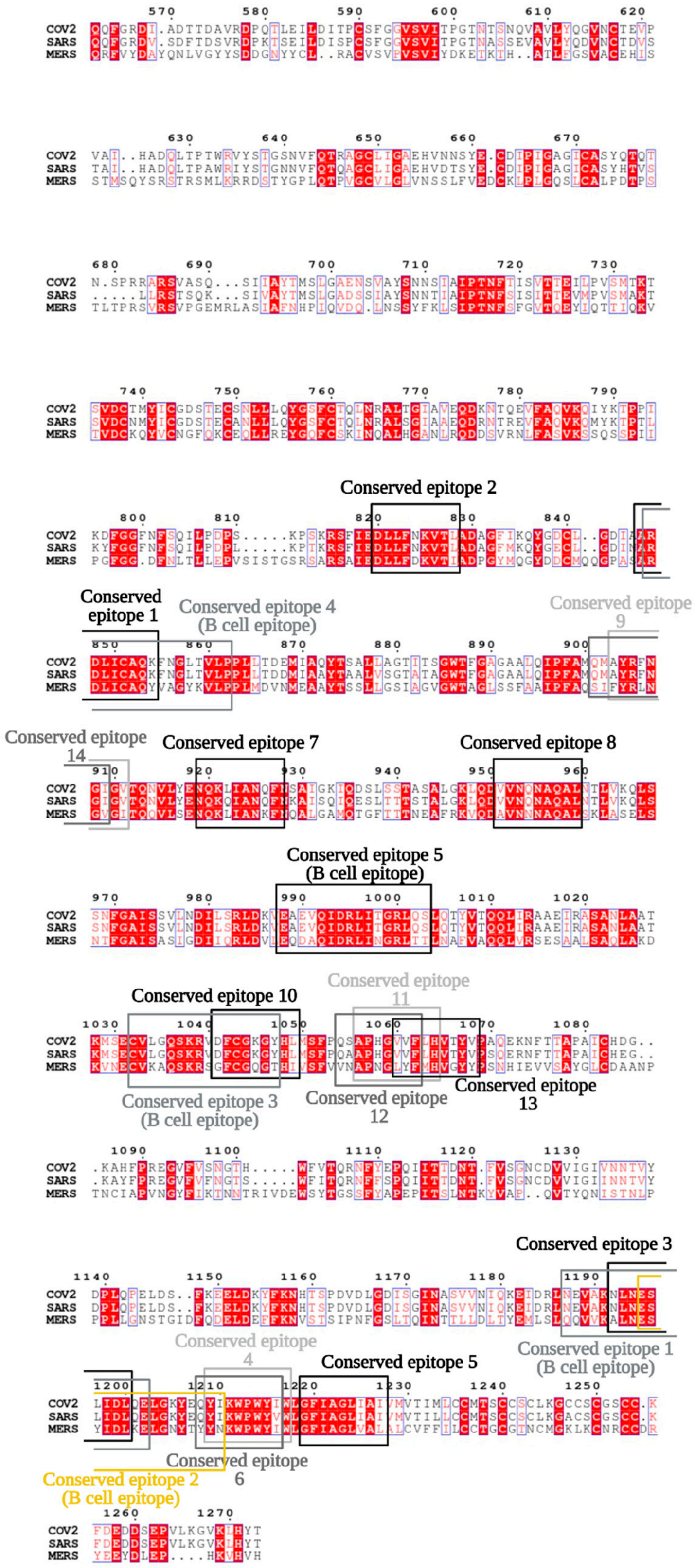
COV2
SARS
MERS
Conserved epitope 2
Conserved epitope 1
Conserved epitope 4 (B cell epitope)
Conserved epitope 9
Conserved epitope 14
Conserved epitope 7
Conserved epitope 8
Conserved epitope 5 (B cell epitope)
Conserved epitope 10
Conserved epitope 11
Conserved epitope 3 (B cell epitope)
Conserved epitope 12
Conserved epitope 13
Conserved epitope 3
Conserved epitope 4
Conserved epitope 5
Conserved epitope 1 (B cell epitope)
Conserved epitope 6
Conserved epitope 2 (B cell epitope)

Supplementary figure S4. Conservation of envelope, membrane, nucleocapsid, and spike protein sequences in SARS-CoV-2, SARS-CoV and MERS-CoV. Consensus sequences of each protein were aligned and conserved regions were identified using ESPript 3.x. Conserved amino acids are colored in red. A. For envelope protein, two CD8+ T cell epitopes 1 and 2, and two B cell epitopes 1 and 2, were found to be conserved among SARS-CoV-2, SARS-CoV and MERS-CoV, which are shown in black and grey squares. Two CD8+ T cell epitopes, 1 and 2, were ~77% and ~44% conserved, respectively, while two B cell epitopes 1 and 2 were, respectively, ~50% and ~42% conserved. B. For membrane protein, four CD8+ T cell epitopes 1, 2, 3 and 4, and one B cell epitope, were found to be conserved among SARS-CoV-2, SARS-CoV and MERS-CoV, which are shown in black and grey squares. Four CD8+ T cell epitopes, 1, 2, 3, and 4 were, respectively, ~88%, ~66%, ~66% and ~44% conserved, while one B cell epitope was ~86% conserved. C. For nucleocapsid protein, eight CD8+ T cell epitopes 1, 2, 3, 4, 5, 6, 7, and 8, and four B cell epitopes 1, 2, 3, and 4, were found to be conserved among SARS-CoV-2, SARS-CoV and MERS-CoV, which are shown in black and grey squares. Eight CD8+ T cell epitopes 1, 2, 3, 4, 5, 6, 7, and 8 – were, respectively, ~77%, ~77%, ~66%, ~66%, ~66%, ~66%, ~55% and ~55% conserved, while four B cell epitope 1, 2, 3, and 4, were, respectively, ~69%, ~56%, ~56% and ~50% conserved. D. For spike protein, 14 CD8+ T cell epitopes, 1, 2, 3, 4, 5, 6, 7, 8, 9, 10, 11, 12, 13, and 14, and five B cell epitopes, 1, 2, 3, 4, and 5, were found to be conserved among SARS-CoV-2, SARS-CoV and MERS-CoV, which are shown in, respectively, black, grey, and orange squares. Fourteen CD8+ T cell epitopes - 1, 2, 3, 4, 5, 6, 7, 8, 9, 10, 11, 12, 13, and 14 – were ~88%, ~77%, ~77%, ~77%, ~77%, ~66%, ~66%, ~66%, ~55%, ~55%, ~55%, ~44%, ~44%, and ~44% conserved, respectively, while five B cell epitope - Conserved epitopes 1, 2, 3, 4, and 5 - are ~63%, ~63%, ~56%, ~50%, and ~50% conserved. Epitopes are numbered in order of the highest conservancy.

A.

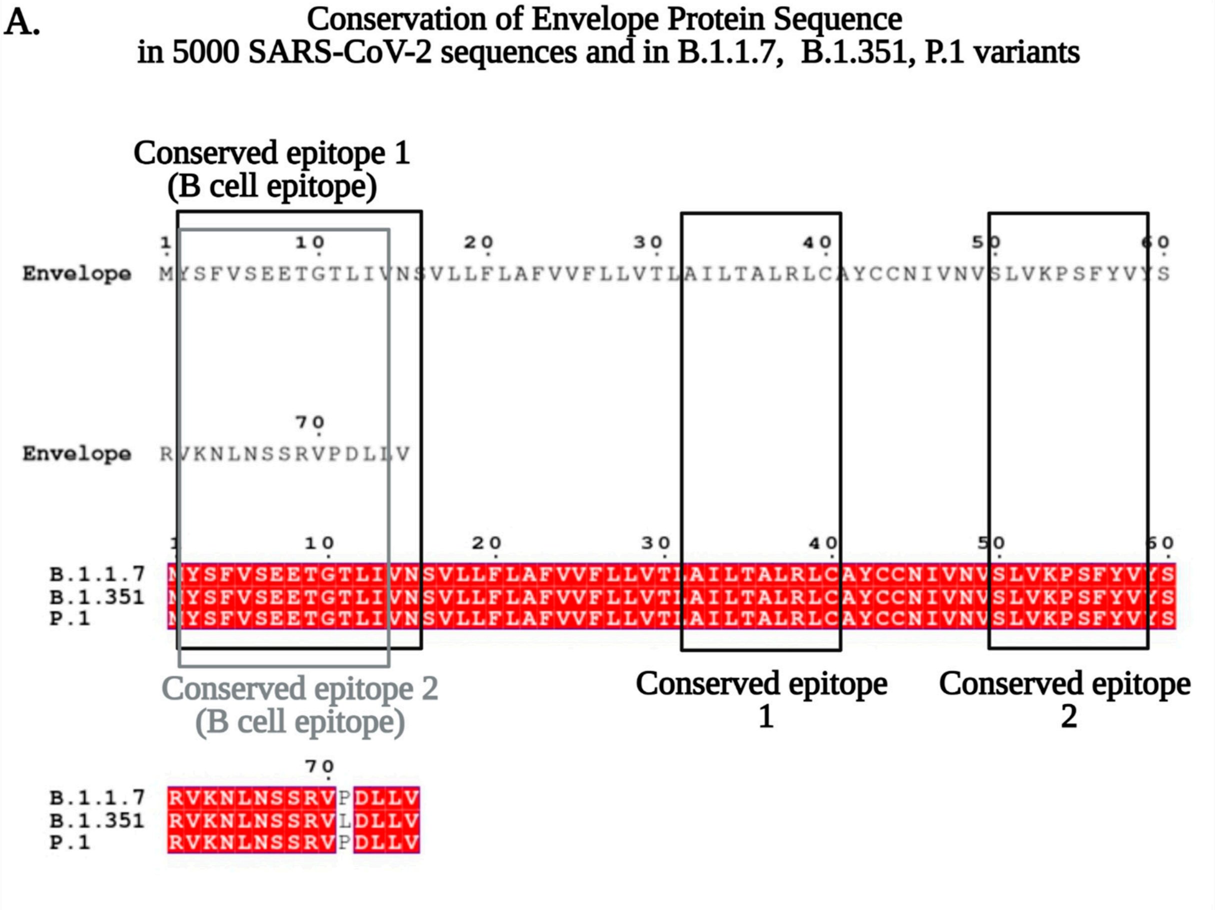
Conservation of Envelope Protein Sequence
in 5000 SARS-CoV-2 sequences and in B.1.1.7, B.1.351, P.1 variants
Conserved epitope 1
(B cell epitope)
Envelope MYSFVSEETGTLIVNSVLLFLAFVVFLLVTLAILTALRLCAYCCNIVNVSLVKPSFYVYS
Envelope RVKNLNSSRVPDLLV
B.1.1.7
B.1.351
P.1
Conserved epitope 2
(B cell epitope)
Conserved epitope
1
Conserved epitope
2
B.1.1.7 RVKNLNSSRVPDLLV
B.1.351 RVKNLNSSRVLDLLV
P.1 RVKNLNSSRVPDLLV

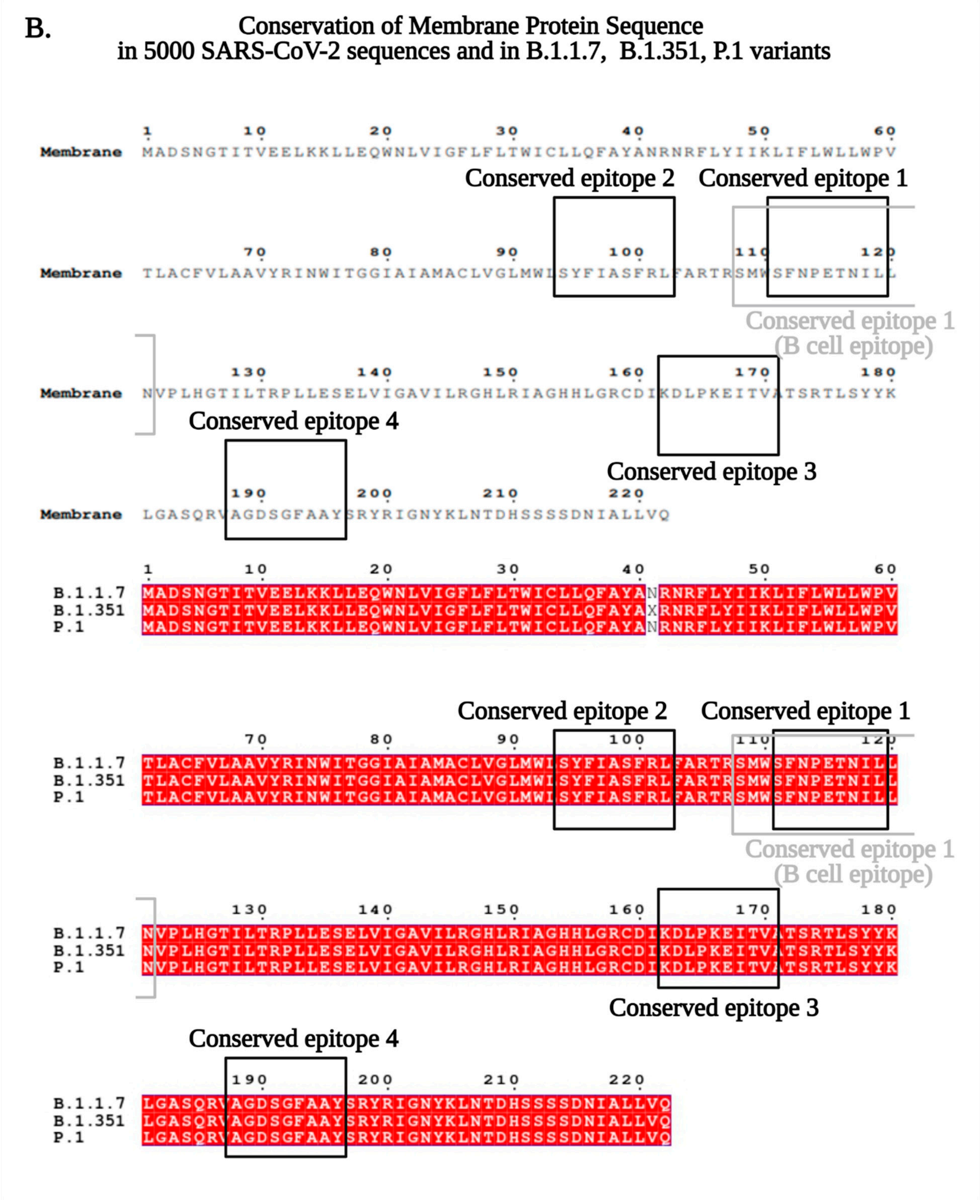
B.
Conservation of Membrane Protein Sequence
in 5000 SARS-CoV-2 sequences and in B.1.1.7, B.1.351, P.1 variants
Membrane MADSNGTITVEELKKLLEQWNLVIGFLFLTWICLLQFAYANRNRFLYIIKLIFLWLLWPV
Membrane TLACFVLAAVYRINWITGGIAIAMACLVGLMWLSYFIASFRLFARTRSMWSFNPETNILL
Membrane NVPLHGTILTRPLLESELVIGAVILRGHLRIAGHHLGRCDIKDLPKEITVATSRTLSYYK
Membrane LGASQRVAGDSGFAAYSRYRIGNYKLNTDHSSSSDNIALLVQ
Conserved epitope 1
Conserved epitope 2
Conserved epitope 3
Conserved epitope 4
Conserved epitope 1 (B cell epitope)
B.1.1.7 MADSNGTITVEELKKLLEQWNLVIGFLFLTWICLLQFAYANRNRFLYIIKLIFLWLLWPV
B.1.351 MADSNGTITVEELKKLLEQWNLVIGFLFLTWICLLQFAYAXRNRFLYIIKLIFLWLLWPV
P.1 MADSNGTITVEELKKLLEQWNLVIGFLFLTWICLLQFAYANRNRFLYIIKLIFLWLLWPV
B.1.1.7 TLACFVLAAVYRINWITGGIAIAMACLVGLMWLSYFIASFRLFARTRSMWSFNPETNILL
B.1.351 TLACFVLAAVYRINWITGGIAIAMACLVGLMWLSYFIASFRLFARTRSMWSFNPETNILL
P.1 TLACFVLAAVYRINWITGGIAIAMACLVGLMWLSYFIASFRLFARTRSMWSFNPETNILL
B.1.1.7 NVPLHGTILTRPLLESELVIGAVILRGHLRIAGHHLGRCDIKDLPKEITVATSRTLSYYK
B.1.351 NVPLHGTILTRPLLESELVIGAVILRGHLRIAGHHLGRCDIKDLPKEITVATSRTLSYYK
P.1 NVPLHGTILTRPLLESELVIGAVILRGHLRIAGHHLGRCDIKDLPKEITVATSRTLSYYK
B.1.1.7 LGASQRVAGDSGFAAYSRYRIGNYKLNTDHSSSSDNIALLVQ
B.1.351 LGASQRVAGDSGFAAYSRYRIGNYKLNTDHSSSSDNIALLVQ
P.1 LGASQRVAGDSGFAAYSRYRIGNYKLNTDHSSSSDNIALLVQ

C.

Conservation of Nucleocapsid Protein Sequence
in 5000 SARS-CoV-2 sequences and in B.1.1.7, B.1.351, P.1 variants

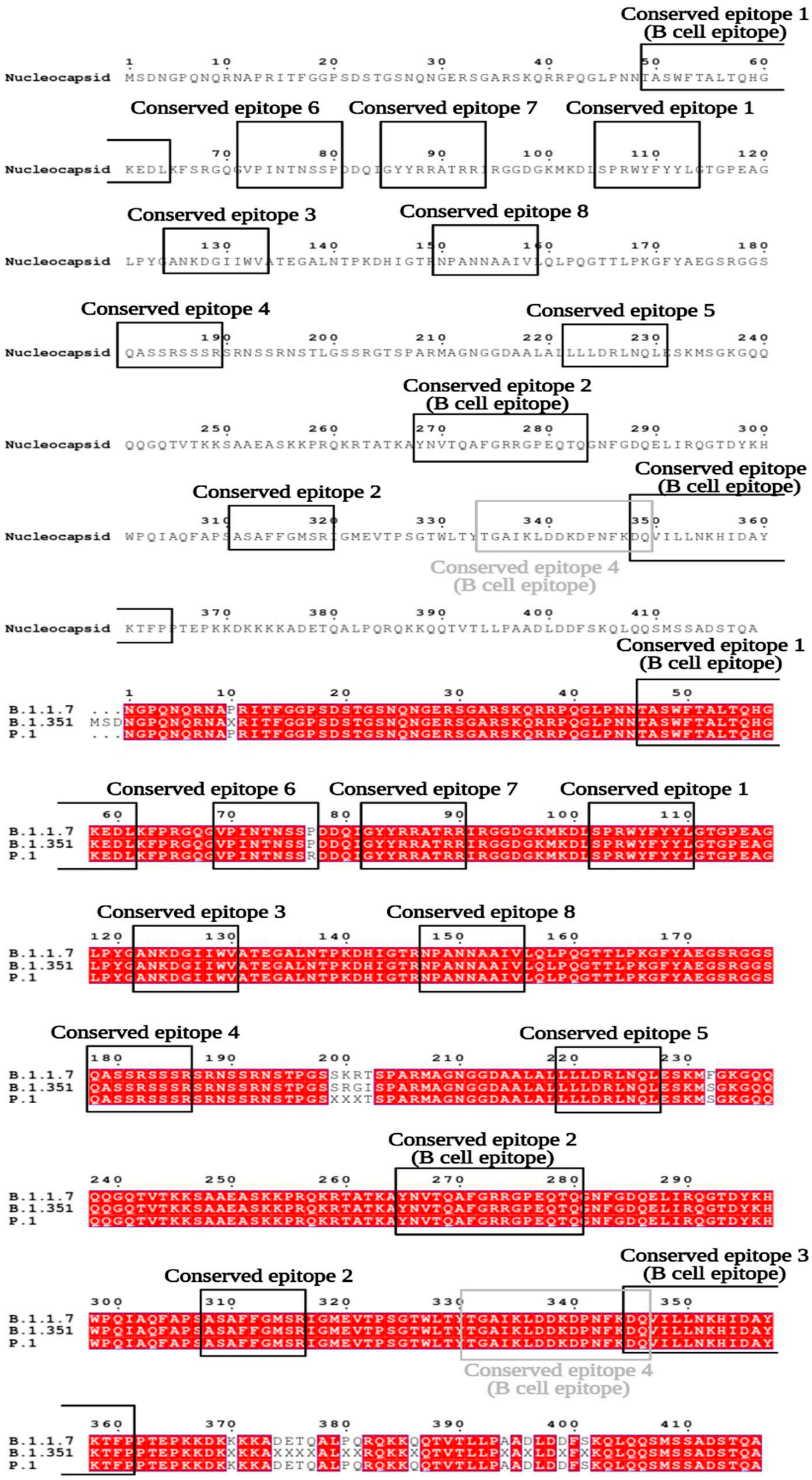

D.

Conservation of Spike Protein Sequence
in 5000 SARS-CoV-2 sequences and in B.1.1.7, B.1.351, P.1 variants

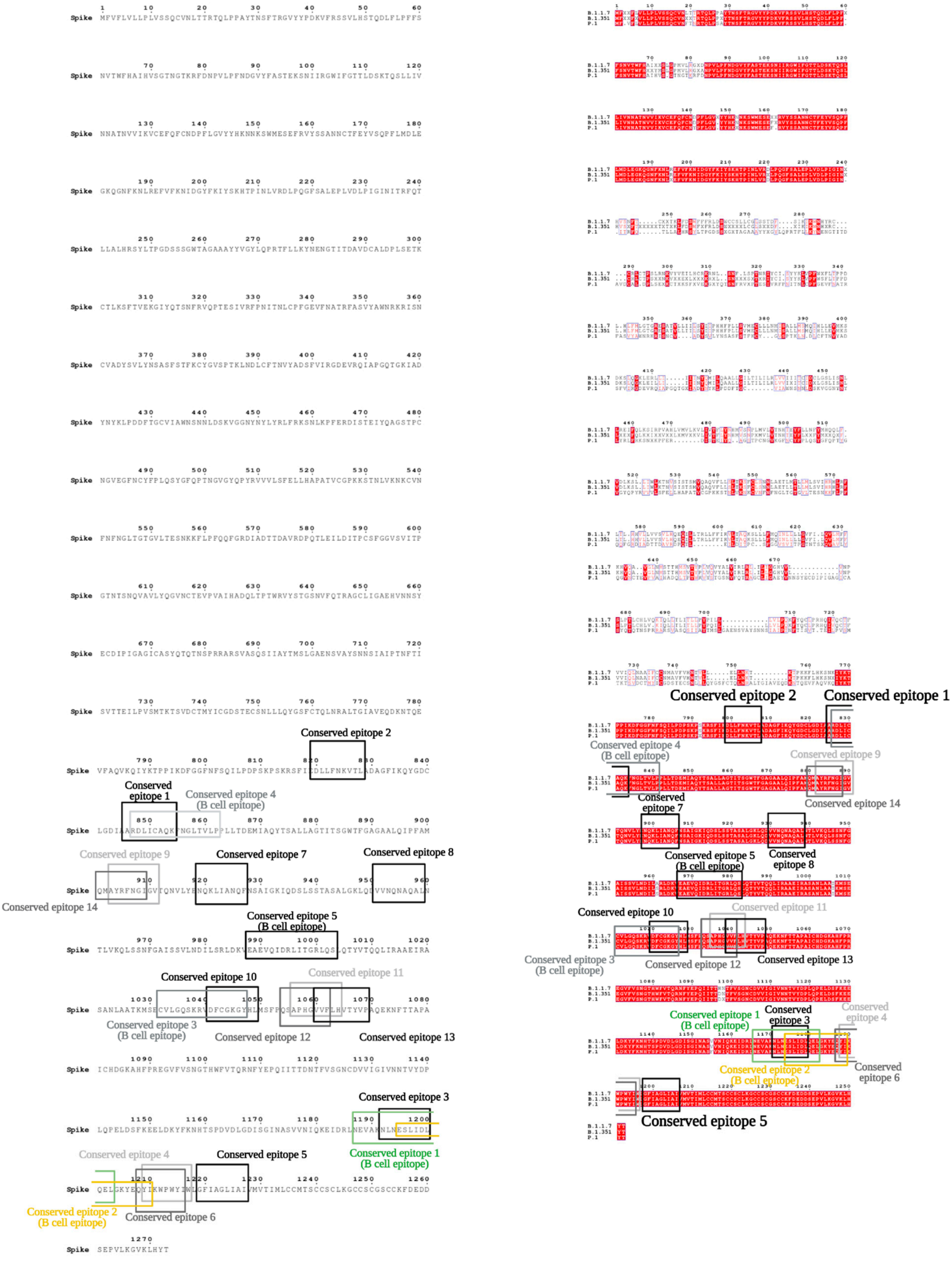

Supplementary figure S5. Conservation of envelope, membrane, nucleocapsid, and spike protein sequence in 5000 SARS-CoV-2 sequences and in B.1.1.7, B.1.351, and P.1 variants of SARS-CoV-2. Consensus sequences of envelope protein were aligned and conserved regions were identified using ESPript 3.x. CD8+ T and B cell epitopes of SARS-CoV-2 predicted to show conserved amino acids in HCoVs were also found to be 100% conserved in different variants of SARS-CoV-2.

Supplementary Table S1. Consensus sequences used to predict the epitopes for vaccine design. Viral protein sequences for Envelop, Membrane, Nucleocapsid and Spike proteins were retrieved using National Center for Biotechnology Information (NCBI) reference sequences for all seven HCoVs: SARS-CoV-2 (NC_045512), SARS-CoV (NC_004718), MERS-CoV (NC_019843), HCoV-NL63 (NC_005831), HCoV-229E (NC_002645), HCoV-HKU1 (NC_006577), and HCoV-OC43 (NC_006213). The protein sequences were then aligned using online MAFFT version 7 (https://mafft.cbrc.jp/alignment/server/) and consensus sequences were generated with Consensus Maker tool (https://www.hiv.lanl.gov/content/sequence/CONSENSUS/SimpCon.html)

**Name of the virus**	**Consensus sequence**
**Envelope**	
SARS-CoV-2	MYSFVSEETGTLIVNSVLLFLAFVVFLLVTLAILTALRLCAYCCNIVNVSLVK PSFYVYSRVKNLNSSRVPDLLV
SARS-CoV	MYSFVSEETGTLIVNSVLLFLAFVVFLLVTLAILTALRLCAYCCNIVNVSLVK PTVYVYSRVKNLNSSEGVPDLLV
MERS-CoV	MLPFVQERIGLFIVNFFIFTVVCAITLLVCMAFLTATRLCVQCMTGFNTLLVQ PALYLYNTGRSVYVKFQDSKPPLPPDEWV
HCoV-NL63	MFLRLIDDNGIVLNSILWLLVMIFFFVLAMTFIKLIQLCFTCHYFFSRTLQPVY KIFLAYQDYMQIAPVPAEVLNV
HCoV-229E	MFLKLVDDHALVVNVLLWCVVLIVILLVCITIIKLIKLCFTCHMFCNRTVYGP IKNVYHIYQSYMHIDPFPKRVIDF

HCoV-OC43	MFMADAYLADTVWYVGQIIFIVAICLLVTIVVVAFLATFKLCIQLCGMCNTL<br>VLSPSIYVFNRGRQFYEFYNDIKPPVLDVDDV
HCoV-HKU1	MVDVFFTDTAWYIGQIFFLVLSCVIFLIFVVALLATIKLCIQICGFCNIFIISPSA<br>YVVYNRGRQLYKSYSEHVIPSTLDDLI
**Membrane**	
SARS-CoV-2	MADSNGTITVEELKKLLEQWNLVIGFLFLTWICLLQFAYANRNRFLYIIKLIF<br>LWLLWPVTLACFVLAAVYRINWITGGIAIAMACLVGLMWLSYFIASFRLFAR<br>TRSMWSFNPETNILLNVPLHGTILTRPLLESELVIGAVILRGHLRIAGHHLGR-<br>CDIKDLPKEITVATSRTLSYYKLGASQRVAGDSGFAAYSRYRIGNYKLNTDH<br>SSSSDNIALLVQ
SARS-CoV	MADNGTITVEELKQLLEQWNLVIGFLFLAWIMLLQFAYSNRNRFLYIIKLVF<br>LWLLWPVTLACFVLAAVYRINWVTGGIAIAMACIVGLMWLSYFVASFRLFA<br>RTRSMWSFNPETNILLNVPLRGTIVTRPLMESELVIGAVIIRGHLRMAGHSLG<br>RCDIKDLPKEITVATSRTLSYYKLGASQRVGTDSGFAAYNRYRIGNYKLNTD<br>HAGSNDNIALLVQ
MERS-CoV	MSNMTQLTEAQIIAIIKDWNFAWSLIFLLITIVLQYGYPSRSMTVYVFKMFVL<br>WLLWPSSMALSIFSAVYPIDLASQIISGIVAAVSAMMWISYFVQSIRLFMRTG<br>SWWSFNPETNCLLNVPFGGTTVVRPLVEDSTSVTAVVTNGHLKMAGMHFG<br>ACDYDRLPNEVTVAKPNVLIALKMVKRQSYGTNSGVAIYHRYKAGNYRSPP<br>ITADIELALLRA
HCoV-NL63	MSNSSVPLSEVYVHLRNWNFSWNLILTVFIVVLQYGHYKYSRLLYGLKMSV<br>LWCLWPLVLALSIFDCFVNFNVDWVFFGFSILMSIITLCLWVMYFVNSFRLW<br>RRVKTFWAFNPETNAIISLQVYGHNYYLPVMAAPTGVTLTLLSGVLLVDGH<br>KIATRVQVGQLPKYVIVATPSTTIVCDRVGRSVNETSQTGWAFYVRAKHGD<br>FSGVASQEGVLSEREKLLHLI

HCoV-229E	MSNDNCTGDIVTHLKNWNFGWNVILTIFIVILQFGHYKYSRLFYGLKMLVL WLLWPLVLALSIFDTWANWDSNWAFVAFSFLMAVSTLVMWVMYFANSFR LFRRARTFWAWNPEVNAITVTTVLGQTYYQPIQQAPTGITVTLLSGVLYVDG HRLASGVQVHNLPEYMTVAVPSTTIIYSRVGRSVNSQNSTGWVFYVRVKHG DFSAVSSPMSNMTENERLLHFF
HCoV-OC43	MSSKTTPAPVYIWTADEAIKFLKEWNFSLGIILLFITIILQFGYTSRSMFVYVIK MIILWLMWPLTIILTIFNCVYALNNVYLGLSIVFTIVAIIMWIVYFVNSIRLFIR TGSFWSFNPETNNLMCIDMKGTMYVRPIIEDYHTLTVTIIRGHLYIQGIKLGT GYSLADLPAYMTVAKVTHLCTYKRGFLDRISDTSGFAVYVKSKVGNYRLPS TQKGSGMDTALLRNNI
HCoV-HKU1	MNKSFFPQFTSDQATFLKEWNFSLGVILLFITIILQFGYTSRSMFVYLIKMIIL WLMWPLTITLTIFNCFYALNNAFLAFSIVFTIISIVIWILYFVNSIRLFIRTGSW WSFNPETNNLMCIDMKGKMFVRPVIEDYHTLTATVIRGHLYIQGVKLGTGY TLSDLPVYVTVAKVQVLCTYKRAFLDKLDVNSGFAVFVKSKVGNYRLPSSK PSGMDTALLRA
**Nucleocapsid**	
SARS-CoV-2	MSDNGPQNQRNAPRITFGGPSDSTGSNQNGERSGARSKQRRPQGLPNNTAS WFTALTQHGKE- DLKFPRGQGVPINTNSSPDDQIGYYRRATRRIRGGDGKMKDLSPRWYFYYL GTGPEAGLPYGANKDGIIWVATEGALNTPKDHIGTRNPANNAAIVLQLPQGT TLPKGFYAEGSRGGSQASSRSSSRSRNSSRNSTPGSSRGTSPARMAGNGGDA ALALLLLDRLNQLESKMSGKGQQQQGQTVTKKSAAEASKKPRQKRTATKA- YNVTQAFGRRGPEQTQGNFGDQELIRQGTDYKHWPQIAQFAPSASAFFGMS RIGMEVTPSGTWLTYTGAIKLDDKDPNFKDQVILLNKHIDAYKTFPPTDFSK QLQQSMSSADSTQA
SARS-CoV	MSDNGPQSNQRSAPRITFGGPTDSTDNNQNGGRNGARPKQRRPQGLPNNTA SWFTALTQHGKEELRFPRGQGVPINTNSGPDDQIGYYRRATRRVRGGDGKM KELSPRWYFYYLGTGPEASLPYGANKEGIVWVATEGALNTPKDHIGTRNPN NNAATVLQLPQGTTLPKGFYAEGSRGGSQASSRSSSRSRGNSRNSTPGSSRG NSPARMASGGGETALALLLLDRLNQLESKVSGKGQQQQGQTVTKKSAAEA

	SKKPRQKRTATKQYNVTQAFGRRGPEQTQGNFGDQDLIRQGTDYKHWPQI<br>AQFAPSASAFFGMSRIGMEVTPSGTWLTYHGAIKLDDKDPQFKDNVILLNKH<br>IDAYKTFPPTEPKKDKKKKTDEAQPLPQRQKKQPTVTLLPAADMDDFSRQL<br>QNSMSGASADSTQA
MERS-CoV	MASPAAPRAVSFADNNDITNTNLSRGRGRNPKPRAAPNNTVSWYTGLTQHG<br>KVPLTFPPGQGVPLNANSTPAQNAGYWRRQDRKINTGNGIKQLAPRWYFYY<br>TGTGPEAALPFRAVKDGIVWVHEDGATDAPSTFGTRNPNNDSAIVTQFAPGT<br>KLPKNFHIEGTGGNSQSSSRASSVSRNSSRSSSQGSRSGNSTRGTSPGPSGIGA<br>VGGDLLYLDLLNRLQALESGKVKQSQPKVITKKDAAAAKNKMRHKRTSTK<br>SFNMVQAFGLRGPGDLQGNFGDLQLNKLGTEDPRWPQIAELAPTASAFMG<br>MSQFKLTHQNNDDHGNPVYFLRYSGAIKLDPKNPNYNKWLELLEQNIDAY<br>KTFPKKEKKQKAPKEESTDQMSEPPKEQRVQGSITQRTRTRPSVQPGPMIDV<br>NTD
HCoV-NL63	MASVNWADDRAARKKFPPPSFYMPLLVSSDKAPYRVIPRNLVPIGKGNKDE<br>QIGYWNVQERWRMRRGQRVDLPPKVHFYYLGTGPHKDLKFRQRSDGVVW<br>VAKEGAKTVNTSLGNRKRNQKPLEPKFSIALPPELSVVEFEDRSNNSSRASSR<br>SSTRNNSRDSSRSTSRQQSRTRSDSNQSSSDLVAAVTLALKNLGFDNQSKSPS<br>SSGTSTPKKPNKPLSQPRADKPSQLKKPRWKRVPTREENVIQCFGPRDFNHN<br>MGDSDLVQNGVDAKGFPQLAELIPNQAALFFDSEVSTDEVGDNVQITYTYK<br>MLVAKDNKNLPKFIEQISAFTKPSSIKEMQSQSSHVVQNTVLNASIPESKPLA<br>DDDSAIIEIVNEVLH
HCoV-229E	MATVKWADASEPQRGRQGRIPYSLYSPLLVDSEQ-PWKVIPRNLVPINKK-<br>DKNKLIGYWNVQKRFRTRKGKRVDLSPKLHFYYLGTGPHKDAKFRERVEG<br>VVWVAVDGAKTEPTGYGVRRKNSEPEIPHFNQKLPNGVTVVEEPDSRAPSR<br>SQSRSQSRSRGESKSQSRNPSSDRNHNSQDDIMKAVAAALKSLGFDKPQEKD<br>KKSAKTGTPKPSRNQSPASSQSAAKILARSQSSETKEQKHEMQKPRWKRQP<br>NDDVTSNVTQCFGPRDLDHNFGSAGVVANGVKAKGYPQFAELVPSTAAML<br>FDSHIVSKESGNTVVLTFTTRVTVPKDHPHLGKFLEELNAFTREMQQQPLLN<br>PSALEFNPSQTSPATVEPVRDEVSIETDIIDEVN
HCoV-OC43	MSFTPGKQSSSRASSGNRSGNGILKWADQSDQFRNVQTRGRRAQPKQTATS<br>QQPSGGNVVPYYSWFSGITQFQKGKEFEFAEGQGVPIAPGVPATEAKGYWY<br>RHNRRSFKTADGNQRQLLPRWYFYYLGTGPHAKDQYGTDIDGVYWVASN<br>QADVNTPADIVDRDPSSDEAIPTRFPPGTVLPQGYYIEGSGRSAPNSRSTSRTS<br>SRASSAGSRSRANSGNRTPTSGVTPDMADQIASLVLAKLGKDATKPQQVTK<br>HTAKEVRQKILNKPRQKRSPNKQCTVQQCFGKRGPNQNFGGGEMLKLGTS

	DPQFPILAELAPTAGAFFFGSRLELAKVQNLSGNPDEPQKDVYELRYNGAIRF DSTLSGFETIMKVLSENLNAYQQQDGMMNMSPKPQRQRGHKNGQGENDNI SVAVPKSRVQQNKSIELTAEDISLLKKMDEPYTEDTSEI
HCoV-HKU1	MSYTPGHHAGSRSSSGNRSGILKKTSWVDQSERSHQTYNRGRKPQPKFTVST QPQGNTIPHYSWFSGITQFQKGRDFKFPDGQGVPIAYGIPPSEAKGYWYKHN RRSFKTADGQQKQLLPRWYFYYLGTGPYANASYGESHEGIFWVASHQADTS IPSDVSARDPTIQEAIPTRFSPGTILPQGYYVEGSGRSASNSRPGSRSQSRGPNN RSLSRSNSNFRHSDSIVKPDMADEIASLVLAKLGKDSKPQQVTKQNAKEIRH KILMKPRQKRTPNKFCNVQQCFGKRGPLQNFGNEMLKLGTNDPQFPILAEL APTPGAFFFGSKLELFKRDSDADSPSKDTFELRYSGSIRFDSTLPGFETIMKVL KENLDAYVNSNQNTVSGSLSPKPQRKRGVKQSPESFDSLNLSADTQHISNDF TPEDHSLLATLDDPYVEDSVA
**Spike**	
SARS-CoV-2	MFVFLVLLPLVSSQCVNLTTRTQLPPAYTNSFTRGVYYPDKVFRSSVLHSTQ DLFLPFFSNVTWFHAIHVSGTNGTKRFDNPVLPFNDGVYFASTEKSNIIRGWI FGTTLDSKTQSLLIVNNATNVVIKVCEFQFCNDPFLGVYYHKNNKSWMESEF RVYSSANNCTFEYVSQPFLMDLEGKQGNFKNLREFVFKNIDGYFKIYSKHTP INLVRDLPQGFSALEPLVDLPIGINITRFQTLLALHRSYLTPGDSSSGWTAGAA AYYVGYLQPRTFLLKYNENGTITDAVDCALDPLSETKCTLKSFTVEKGIYQT SNFRVQPTESIVRFPNITNLCPFGEVFNATRFASVYAWNRKRISNCVADYSVL YNSASFSTFKCYGVSPTKLNDLCFTNVYADSFVIRGDEVRQIAPGQTGKIAD YNYKLPDDFTGCVIAWNSNNLDSKVGGNYNYLYRLFRKSNLKPFERDISTEI YQAGSTPCNGVEGFNCYFPLQSYGFQPTNGVGYQPYRVVVLSFELLHAPAT VCGPKKSTNLVKNKCVNFNFNGLTGTGVLTESNKKFLPFQQFGRDIADTTD AVRDPQTLEILDITPCSFGGVSVITPGTNTSNQVAVLYQGVNCTEVPVAIHAD QLTPTWRVYSTGSNVFQTRAGCLIGAEHVNNSYECDIPIGAGICASYQTQTN SPRRARSVASQSIIAYTMSLGAENSVAYSNNSIAIPTNFTISVTTEILPVSMTKT SVDCTMYICGDSTECSNLLLQYGSFCTQLNRALTGIAVEQDKNTQEVFAQV KQIYKTPPIKDFGGFNFSQILPDPSKPSKRSFIEDLLFNKVTLADAGFIKQYGD CLGDIAARDLICAQKFNGLTVLPPLLTDEMIAQYTSALLAGTITSGWTFGAG AALQIPFAMQMAYRFNGIGVTQNVLYENQKLIANQFNSAIGKIQDSLSSTAS ALGKLQDVVNQNAQALNTLVKQLSSNFGAISSVLNDILSRLDKVEAEVQIDR LITGRLQSLQTYVTQQLIRAAEIRASANLAATKMSECVLGQSKRVDFCGKGY

	HLMSFPQSAPHGVVFLHVTYVPAQEKNFTTAPAICHDGKAHFPREGVFVSN<br>GTHWFVTQRNFYEPQIITTDNTFVSGNCDVVIGIVNNTVYDPLQPELDSFKEE<br>LDKYFKNHTSPDVDLGDISGINASVVNIQKEIDRLNEVAKNLNESLIDLQELG<br>KYEQYIKWPWYIWLGFIAGLIAIVMVTIMLCCMTSCCSCLKGCCSCGSCCKF<br>DEDDSEPVLKGVKLHYT
SARS-CoV	MFIFLLFLTLTSGSDLDRCTTFDDVQAPNYTQHTSSMRGVYYPDEIFRSDTLY<br>LTQDLFLPFYSNVTGFHTINHTFGNPVIPFKDGIYFAATEKSNVVRGWVFGST<br>MNNKSQSVIIINNSTNVVIRACNFELCDNPFFAVSKPMGTQTHTMIFDNAFNC<br>TFEYISDAFSLDVSEKSGNFKHLREFVFKNKDGFLYVYKGYQPIDVVRDLPS<br>GFNTLKPIFKLPLGINITNFRAILTAFSPAQDIWGTSAAAYFVGYLKPTTFMLK<br>YDENGTITDAVDCSQNPLAELKCSVKSFEIDKGIYQTSNFRVVPSGDVVRFPN<br>ITNLCPFGEVFNATKFPSVYAWERKKISNCVADYSVLYNSTFFSTFKCYGVS<br>ATKLNDLCFSNVYADSFVVKGDDVRQIAPGQTGVIADYNYKLPDDFMGCV<br>LAWNTRNIDATSTGNYNYKYRYLRHGKLRPFERDISNVPFSPDGKPCTPPAL<br>NCYWPLNDYGFYTTTGIGYQPYRVVVLSFELLNAPATVCGPKLSTDLIKNQC<br>VNFNFNGLTGTGVLTPSSKRFQPFQQFGRDVSDFTDSVRDPKTSEILDISPCSF<br>GGVSVITPGTNASSEVAVLYQDVNCTDVSTAIHADQLTPAWRIYSTGNNVFQ<br>TQAGCLIGAEHVDTSYECDIPIGAGICASYHTVSLLRSTSQKSIVAYTMSLGA<br>DSSIAYSNNTIAIPTNFSISITTEVMPVSMAKTSVDCNMYICGDSTECANLLLQ<br>YGSFCTQLNRALSGIAAEQDRNTREVFAQVKQMYKTPTLKYFGGFNFSQILP<br>DPLKPTKRSFIEDLLFNKVTLADAGFMKQYGECLGDINARDLICAQKFNGLT<br>VLPPLLTDDMIAAYTAALVSGTATAGWTFGAGAALQIPFAMQMAYRFNGIG<br>VTQNVLYENQKQIANQFNKAISQIQESLTTTSTALGKLQDVVNQNAQALNTL<br>VKQLSSNFGAISSVLNDILSRLDKVEAEVQIDRLITGRLQSLQTYVTQQLIRA<br>AEIRASANLAATKMSECVLGQSKRVDFCGKGYHLMSFPQAAPHGVVFLHVT<br>YVPSQERNFTTAPAICHEGKAYFPREGVFVFNGTSWFITQRNFFSPQIITTDNT<br>FVSGNCDVVIGIINNTVYDPLQPELDSFKEELDKYFKNHTSPDVDLGDISGIN<br>ASVVNIQKEIDRLNEVAKNLNESLIDLQELGKYEQYIKWPWYVWLGFIAGLI<br>AIVMVTILLCCMTSCCSCLKGACSCGSCCKFDEDDSEPVLKGVKLHYT
MERS-CoV	MIHSVFLLMFLLTPTESYVDVGPDSVKSACIEVDIQQTFFDKTWPRPIDVSKA<br>DGIIYPQGRTYSNITITYQGLFPYQGDHGDMYVYSAGHATGTTPQKLFVANY<br>SQDVKQFANGFVVRIGAAANSTGTVIISPSTSATIRKIYPAFMLGSSVGNFSD<br>GKMGRFFNHTLVLLPDGCGTLLRAFYCILEPRSGNHCPAGNSYTSFATYHTP<br>ATDCSDGNYNRNASLNSFKEYFNLRNCTFMYTYNITEDEILEWFGITQTAQG<br>VHLFSSRYVDLYGGNMFQFATLPVYDTIKYYSIIPHSIRSIQSDRKAWAAFYV<br>YKLQPLTFLLDFSVDGYIRRAIDCGFNDLSQLHCSYESFDVESGVYSVSSFEA<br>KPSGSVVEQAEGVECDFSPLLSGTPPQVYNFKRLVFTNCNYNLTKLLSLFSV<br>NDFTCSQISPAAIASNCYSSLILDYFSYPLSMKSDLSVSSAGPISQFNYKQSFSN<br>PTCLILATVPHNLTTITKPLKYSYINKCSRLLSDDRTEVPQLVNANQYSPCVSI<br>VPSTVWEDGDYYRKQLSPLEGGGWLVASGSTVAMTEQLQMGFGITVQYGT

	DTNSVCPKLEFANDTKIASQLGNCVEYSLYGVSGRGVFQNCTAVGVRQQRF VYDAYQNLVGYYSDDGNYYCLRACVSVPVSVIYDKETKTHATLFGSVACE HISSTMSQYSRSTRSMLKRRDSTYGPLQTPVGCVLGLVNSSLFVEDCKLPLG QSLCALPDTPSTLTPRSVRSVPGEMRLASIAFNHPIQVDQLNSSYFKLSIPTNF SFGVTQEYIQTTIQKVTVDCKQYVCNGFQKCEQLLREYGQFCSKINQALHGA NLRQDDSVRNLFASVKSSQSSPIIPGFGGDFNLTLLEPVSISTGSRSARSAIEDL LFDKVTIADPGYMQGYDDCMQQGPASARDLICAQYVAGYKVLPPLMDVN MEAAYTSSLLGSIAGVGWTAGLSSFAAIPFAQSIFYRLNGVGITQQVLSENQK LIANKFNQALGAMQTGFTTTNEAFRKVQDAVNNNAQALSKLASELSNTFGA ISASIGDIIQRLDVLEQDAQIDRLINGRLTTLNAFVAQQLVRSESAALSAQLAK DKVNECVKAQSKRSGFCGQGTHIVSFVVNAPNGLYFMHVGYYPSNHIEVVS AYGLCDAANPTNCIAPVNGYFIKTNNTRIVDEWSYTGSSFYAPEPITSLNTKY VAPQVTYQNISTNLPPPLLGNSTGIDFQDELDEFFKNVSTSIPNFGSLTQINTTL LDLTYEMLSLQQVVKALNESYIDLKELGNYTYYNKWPWYIWLGFIAGLVAL ALCVFFILCCTGCGTNCMGKLKCNRCCDRYEEYDLEPHKVHVH
HCoV-NL63	MKLFLILLVLPLASCFFTCNSNANLSMLQLGVPDNSSTIVTGLLPTHWICANQ STSVYSANGFFYIDVGNHRSAFALHTGYYDVNQYYIYVTNEIGLNASVTLKI CKFGINTTFDFLSNSSSSFDCIVNLLFTEQLGAPLGITISGETVRLHLYNVTRTF YVPAAYKLTKLSVKCYFNYSCVFSVVNATVTVNVTTHNGRVVNYTVCDDC NGYTDNIFSVQQDGRIPNGFPFNNWFLLTNGSTLVDGVSRLYQPLRLTCLWP VPGLKSSTGFVYFNATGSDVNCNGYQHNSVADVMRYNLNFSANSVDNLKS GVIVFKTLQYDVLFYCSNSSSGVLDTTIPFGPSSQPYYCFINSTINTTHVSTFV GVLPPTVREIVVARTGQFYINGFKYFDLGFIEAVNFNVTTASATDFWTVAFA TFVDVLVNVSATKIQNLLYCDSPFEKLQCEHLQFGLQDGFYSANFLDDNVLP ETYVALPIYYQHTDINFTATASFGGSCYVCKPHQVNISLNGNTSVCVRTSHFS IRYIYNRVKSGSPGDSSWHIYLKSGTCPFSFSKLNNFQKFKTICFSTVAVPGSC NFPLEATWHYTSYTIVGALYVTWSEGNSITGVPYPVSGIREFSNLVLNNCTK YNIYDYVGTGIIRSSNQSLAGGITYVSNSGNLLGFKNVSTGNIFIVTPCNQPDQ VAVYQQSIIGAMTAVNESRYGLQNLLQLPNFYYVSNGGNNCTTAVMTYSNF GICADGSLIPVRPRNSSDNGISAIITANLSIPSNWTTSVQVEYLQITSTPIVVDC ATYVCNGNPRCKNLLKQYTSACKTIEDALRLSAHLETNDVSSMLTFDSNAFS LANVTSFGDYNLSSVLPQRNIHSSRIAGRSALEDLLFSKVVTSGLGTVDVDY KSCTKGLSIADLACAQYYNGIMVLPGVADAERMAMYTGSLIGGMVLGGLT SAAAIPFSLALQARLNYVALQTDVLQENQKILAASFNKAINNIVASFSSVNDA ITQTAEAIHTVTIALNKIQDVVNQQGSALNHLTSQLRHNFQAISNSIQAIYDRL DSIQADQQVDRLITGRLAALNAFVSQVLNKYTEVRSSRRLAQQKINECVKSQ SNRYGFCGNGTHIFSIVNSAPDGLLFLHTVLLPTDYKNVKAWSGICVDGIYG YVLRQPNLVLYSDNGVFRVTSRVMFQPRLPVLSDFVQIYNCNVTFVNISRVE LHTVIPDYVDVNKTLQEFAQNLPKYVKPNFDLTPFNLTYLNLSSELKQLEAK TASLFQTTVELQGLIDQINSTYVDLKLLNRFENYIKWPWWVWLIISVVFVVL LSLLVFCCLSTGCCGCCNCLTSSMRGCCDCGSTKLPYYEFEKVHVQ

HCoV-229E	MFVLLVAYALLHIAGCQTTNGTNTSHSVCNGCVGHSENVFAVESGGYIPSNF<br>AFNNWFLLTNTSSVVDGVVRSFQPLLLNCLWSVSGSRFTTGFVYFNGTGRG<br>DCKGFYSNASSDVIRYNINFEENLRRGTILFKTSYGAVVFYCTNNTLVSGDA<br>HIPSGTVLGNFYCFVNTTIGNETTSAFVGALPKTVREFVISRTGHFYINGYRYF<br>SLGNVEAVNFNVTNAATTVCTVALASYADVLVNVSQTAIANIIYCNSVINRL<br>RCDQLSFDVPDGFYSTSPIQSVELPVSIVSLPVYHKHTFIVLYVNFELRRGPGR<br>CYNCRPAVVNITLANFNETKGPLCVDTSHFTTQFVGVKFDRWSASINTGNCP<br>FSFGKVNNFVKFGSVCFSLKDIPGGCAMPIMANLVNHKSHNIGSLYVSWSDG<br>DVITGVPKPVEGVSSFMNVTLNKCTKYNIYDVSGVGVIRISNDTFLNGITYTS<br>TSGNLLGFKDVTNGTIYSITPCNPPDQLVVYQQAVVGAMLSENFTSYGFSNV<br>VEMPKFFYASNGTYNCTDAVLTYSSFGVCADGSIIAVQPRNVSYDSVSAIVT<br>ANLSIPSNWTTSVQVEYLQITSTPIVVDCSTYVCNGNVRCVELLKQYTSACK<br>TIEDALRNSAMLESADVSEMLTFDKKAFTLANVSSFGDYNLSSVIPSLPRSGS<br>RVAGRSAIEDILFSKLVTSGLGTVDADYKKCTKGLSIADLACAQYYNGIMVL<br>PGVADAERMAMYTGSLIGGIALGGLTSAASIPFSLAIQSRLNYVALQTDVLQ<br>ENQKILAASFNKAMTNIVDAFTGVNDAITQTSQALQTVATALNKIQDVVNQ<br>QGNSLNHLTSQLRQNFQAISSSIQAIYDRLDIIQADQQVDRLITGRLAALNVF<br>VSHTLTKYTEVRASRQLAQQKVNECVKSQSKRYGFCGNGTHIFSLVNAAPE<br>GLVFLHTVLLPTQYKDVEAWSGLCVDGINGYVLRQPNLALYKEGNYYRITS<br>RIMFEPRIPTIADFVQIENCNVTFVNISRSELQTIVPEYIDVNKTLQELSYKLPN<br>YTVPDLVVEQYNQTILNLTSEISTLENKSAELNYTVQKLQTLIDNINSTLVDL<br>KWLNRVETYIKWPWWVWLCISVVLIFVVSMLLLCCCSTGCCGFFSCFASSIR<br>GCCESTKLPYYDVEKIHIQ
HCoV-OC43	MFLILLISLPTAFAVIGDLNCPLDPRLKGSFNNRDTGPPSISTDTVDVTNGLGT<br>YYVLDRVYLNTTLFLNGYYPTSGSTYRNMALKGTDLLSTLWFKPPFLSDFIN<br>GIFAKVKNTKVFKDGVMYSEFPAITIGSTFVNTSYSVVVQPRTINSTQDGVN<br>KLQGLLEVSVCQYNMCEYPHTICHPNLGNHFKELWHLDTGVVSCLYKRNFT<br>YDVNATYLYFHFYQEGGTFYAYFTDTGFVTKFLFNVYLGMALSHYYVMPL<br>TCISRRDIGFTLEYWVTPLTPRQYLLAFNQDGIIFNAVDCMSDFMSEIKCKTQ<br>SIAPPTGVYELNGYTVQPIADVYRRKPDLPNCNIEAWLNDKSVPSPLNWERK<br>TFSNCNFNMSSLMSFIQADSFTCNNIDAAKIYGMCFSSITIDKFAIPNRRKVDL<br>QLGNLGYLQSSNYRIDTTATSCQLYYNLPAANVSVSRFNPSTWNKRFGFIED<br>SVFVPQPTGVFTNHSVVYAQHCFKAPKNFCPCSSCPGKNNGIGTCPAGTNYL<br>TCDNLCTLDPITFKAPDTYKCPQTKSLVGIGEHCSGLAVKSDYCGNNSCTCQ<br>PQAFLGWSADSCLQGDKCNIFANFILHDVNNGLTCSTDLQKANTEIELGVCV<br>NYDLYGISGQGIFVEVNATYYNSWQNLLYDSNGNLYGFRDYITNRTFMIHSC<br>YSGRVSAAYHANSSEPALLFRNIKCNYVFNNSLTRQLQPINYSFDSYLGCVV<br>NAYNSTAISVQTCDLTVGSGYCVDYSKNRRSRRAITTGYRFTNFEPFTVNSV<br>NDSLEPVGGLYEIQIPSEFTIGNMEEFIQTSSPKVTIDCAAFVCGDYAACKLQL<br>VEYGSFCDNINAILTEVNELLDTTQLQVANSLMNGVTLSTKLKDGVNFNVD

	DINFSPVLGCLGSECSKASSRSAIEDLLFDKVKLSDVGFVEAYNNCTGGAEIR<br>DLICVQSYKGIKVLPPLLSENQISGYTLAATSASLFPPWTAAAGVPFYLNVQY<br>RINGLGVTMDVLSQNQKLIANAFNNALHAIQQGFDATNSALVKIQAVVNAN<br>AEALNNLLQQLSNRFGAISASLQEILSRLDALEAEAQIDRLINGRLTALNAYV<br>SQQLSDSTLVKFSAAQAMEKVNECVKSQSSRINFCGNGNHIISLVQNAPYGL<br>YFIHFNYVPTKYVTAKVSPGLCIAGNRGIAPKSGYFVNVNNTWMYTGSGYY<br>YPEPITENNVVVMSTCAVNYTKAPYVMLNTSIPNLPDFKEELDQWFKNQTS<br>VAPDLSLDYINVTFLDLQVEMNRLQEAIKVLNHSYINLKDIGTYEYYVKWP<br>WYVWLLICLAGVAMLVLLFFICCCTGCGTSCFKKCGGCCDDYTGYQELVIK<br>TSHDD
HCoV-HKU1	MLLIIFILPTTLAVIGDFNCTNFAINDKNTTVPRISEYVVDVSYGLGTYYILDR<br>VYLNTTILFTGYFPKSGANFRDLSLKGTTYLSTLWYQKPFLSDFNNGIFSRVK<br>NTKLYVNKTLYSEFSTIVIGSVFINNSYTIVVQPHNGVLEITACQYTMCEYPH<br>TICKSKGSSRNESWHFDKSEPLCLFKKNFTYNVSTDWLYFHFYQERGTFYAY<br>YADSGMPTTFLFSLYLGTLLSHYYVLPLTCNAISSNTDNETLQYWVTPLSKR<br>QYLLKFDNRGVITNAVDCSSSFFSEIQCKTKSLLPNTGVYDLSGFTVKPVATV<br>HRRIPDLPDCDIDKWLNNFNVPSPLNWERKIFSNCNFNLSTLLRLVHTDSFSC<br>NNFDESKIYGSCFKSIVLDKFAIPNSRRSDLQLGSSGFLQSSNYKIDTTSSSCQ<br>LYYSLPAINVTINNYNPSSWNRRYGFNNFNLSSHSVVYSRYCFSVNNTFCPC<br>AKPSFASSCKSHKPPSASCPIGTNYRSCESTTVLDHTDWCRCSCLPDPITAYD<br>PRSCSQKKSLVGVGEHCAGFGVDEEKCGVLDGSYNVSCLCSTDAFLGWSYD<br>TCVSNNRCNIFSNFILNGINSGTTCSNDLLQPNTEVFTDVCVDYDLYGITGQG<br>IFKEVSAVYYNSWQNLLYDSNGNIIGFKDFVTNKTYNIFPCYAGRVSAAFHQ<br>NASSLALLYRNLKCSYVLNNISLATQPYFDSYLGCVFNADNLTDYSVSSCAL<br>RMGSGFCVDYNSPSSSSSRRKRRSISASYRFVTFEPFNVSFVNDSIESVGGLYE<br>IKIPTNFTIVGQEEFIQTNSPKVTIDCSLFVCSNYAACHDLLSEYGTFCDNINSI<br>LDEVNGLLDTTQLHVADTLMQGVTLSSNLNTNLHFDVDNINFKSLVGCLGP<br>HCGSSSRSFFEDLLFDKVKLSDVGFVEAYNNCTGGSEIRDLLCVQSFNGIKVL<br>PPILSESQISGYTTAATVAAMFPPWSAAAGIPFSLNVQYRINGLGVTMDVLN<br>KNQKLIATAFNNALLSIQNGFSATNSALAKIQSVVNSNAQALNSLLQQLFNK<br>FGAISSSLQEILSRLDALEAQVQIDRLINGRLTALNAYVSQQLSDISLVKFGAA<br>LAMEKVNECVKSQSPRINFCGNGNHILSLVQNAPYGLLFMHFSYKPISFKTV<br>LVSPGLCISGDVGIAPKQGYFIKHNDHWMFTGSSYYYPEPISDKNVVFMNTC<br>SVNFTKAPLVYLNHSVPKLSDFESELSHWFKNQTSIAPNLTLNLHTINATFLD<br>LYYEMNLIQESIKSLNNSYINLKDIGTYEMYVKWPWYVWLLISFSFIIFLVLLF<br>FICCCTGCGSACFSKCHNCCDEYGGHHDFVIKTSHDD

Supplementary Table S2: List of all epitopes predicted with CTLpred. Consensus sequences of Envelope, Membrane, Nucleocapsid, and Spike proteins from SARS-CoV-2, SARS-CoV, MERS-CoV, HCoV-NL63, HCoV-229E, HCoV-OC43, and HCoV-HKU1 were fed into the CTLpred (https://webs.iiitd.edu.in/raghava/ctlpred/) with ANN/SVM combined approach with default settings. Shortlisted epitopes used for the vaccine construct are highlighted in bold letters.

**SARS-CoV-2**				
**Viral protein name**	**Rank**	**Start position**	**Sequence**	**Score (ANN/SVM)**
**Envelope**	1	13	IVNSVLLFL	0.89/1.3323721
	2	41	AYCCNIVNV	0.50/1.7081903
	3	51	LVKPSFYVY	0.70/0.7574663
	4	16	SVLLFLAFV	0.03/1.2319935
	5	57	YVYSRVKNL	0.43/0.65777634
	6	35	TALRLCAYC	0.80/0.27596158
	7	25	VFLLVTLAI	0.99/0.065623615
	**8**	**50**	**SLVKPSFYV**	**0.00/0.99144443**
	9	38	RLCAYCCNI	0.27/0.70361798
	10	20	FLAFVVFLL	0.02/0.94021965
	11	17	VLLFLAFVV	0.00/0.86079609
	12	23	FVVFLLVTL	0.08/0.76653538
	13	26	FLLVTLAIL	0.10/0.72134398
	14	11	TLIVNSVLL	0.08/0.73778864
	15	24	VVFLLVTLA	0.45/0.36571657
	16	34	LTALRLCAY	0.74/0.0034344671
	17	19	LFLAFVVFL	0.31/0.42809059
	18	53	KPSFYVYSR	0.02/0.66563238
	19	66	NSSRVPDLL	/0.67715744
	20	43	CCNIVNVSL	0.02/0.64476164
	21	44	CNIVNVSLV	0.10/0.55377124
	22	5	VSEETGTLI	0.77/-0.12141075
	23	12	LIVNSVLLF	0.64/-0.029654824
	24	18	LLFLAFVVF	0.01/0.57949258
	25	52	VKPSFYVYS	0.99/-0.45153825

	26	56	FYVYSRVKN	0.94/-0.42336705
	27	42	YCCNIVNVS	0.90/-0.39556418
	**28**	**32**	**AILTALRLC**	**0.61/-0.15241169**
	29	36	ALRLCAYCC	0.01/0.40105212
	30	37	LRLCAYCCN	0.97/-0.57229533
	31	30	TLAILTALR	0.00/0.36066322
	32	60	SRVKNLNSS	0.76/-0.51255186
	33	27	LLVTLAILT	0.95/-1.0998769
	34	2	YSFVSEETG	0.63/-0.89302618
**SARS-CoV**				
**Viral protein name**	**Rank**	**Start position**	**Sequence**	**Score (ANN/SVM)**
**Envelope**	1	13	IVNSVLLFL	0.89/1.3323721
	2	41	AYCCNIVNV	0.50/1.7081903
	**3**	**50**	**SLVKPTVYV**	**0.87/1.1235574**
	4	16	SVLLFLAFV	0.03/1.2319935
	5	51	LVKPTVYVY	0.45/0.78461679
	6	57	YVYSRVKNL	0.43/0.65777634
	7	35	TALRLCAYC	0.80/0.27596158
	8	25	VFLLVTLAI	0.99/0.065623615
	9	38	RLCAYCCNI	0.27/0.70361798
	10	20	FLAFVVFLL	0.02/0.94021965
	11	17	VLLFLAFVV	0.00/0.86079609
	12	23	FVVFLLVTL	0.08/0.76653538
	13	26	FLLVTLAIL	0.10/0.72134398
	14	11	TLIVNSVLL	0.08/0.73778864
	15	24	VVFLLVTLA	0.45/0.36571657
	16	34	LTALRLCAY	0.74/0.0034344671
	17	19	LFLAFVVFL	0.31/0.42809059
	18	43	CCNIVNVSL	0.02/0.64476164
	19	44	CNIVNVSLV	0.10/0.55377124
	20	5	VSEETGTLI	0.77/-0.12141075
	21	12	LIVNSVLLF	0.64/-0.029654824
	22	18	LLFLAFVVF	0.01/0.57949258
	23	66	NSSEGVPDL	/0.58120937
	24	67	SSEGVPDLL	/0.57470504
	25	42	YCCNIVNVS	0.90/-0.39556418
	26	56	VYVYSRVKN	0.94/-0.46663835
	**27**	**32**	**AILTALRLC**	**0.61/-0.15241169**

	28	36	ALRLCAYCC	0.01/0.40105212
	29	48	NVSLVKPTV	0.00/0.40380231
	30	37	LRLCAYCCN	0.97/-0.57229533
	31	30	TLAILTALR	0.00/0.36066322
	32	52	VKPTVYVYS	0.97/-0.9738047
	33	27	LLVTLAILT	0.95/-1.0998769
	34	2	YSFVSEETG	0.63/-0.89302618
	35	47	VNVSLVKPT	0.69/-1.5062236
	36	65	LNSSEGVPD	0.90/-1.8194142
**MERS-CoV**				
**Viral protein name**	**Rank**	**Start position**	**Sequence**	**Score (ANN/SVM)**
**Envelope**	1	50	LLVQPALYL	0.79/0.84185633
	2	1	MLPFVQERI	0.94/0.49325186
	3	25	ITLLVCMAF	0.96/0.38790733
	4	24	AITLLVCMA	0.64/0.66448331
	5	9	IGLFIVNFF	0.44/0.78198539
	6	44	MTGFNTLLV	0.31/0.71660277
	7	8	RIGLFIVNF	0.05/0.97063083
	8	13	IVNFFIFTV	0.00/1.0043246
	9	60	NTGRSVYYK	0.31/0.65772969
	10	26	TLLVCMAFL	0.08/0.85346175
	11	57	YLYNTGRSV	0.00/0.89588326
	12	12	FIVNFFIFT	0.82/0.061943241
	13	42	QCMTGFNTL	0.91/-0.050979385
	14	36	ATRLCVQCM	0.00/0.84974322
	15	19	FTVVCAITL	0.04/0.80762511
	16	17	FIFTVVCAI	0.17/0.66048223
	17	16	FFIFTVVCA	0.25/0.56456289
	18	53	QPALYLYNT	0.52/0.26424758
	19	10	GLFIVNFFI	0.11/0.54082069
	20	3	PFVQERIGL	0.75/-0.14228684
	21	11	LFIVNFFIF	0.03/0.55199648
	22	47	FNTLLVQPA	0.85/-0.28167078
	23	58	LYNTGRSVY	0.05/0.51129716
	24	31	MAFLTATRL	0.13/0.40104314
	25	59	YNTGRSVYV	0.16/0.36364365
	26	33	FLTATRLCV	0.12/0.40129342
	27	55	ALYLYNTGR	0.99/-0.47537099

	28	20	TVVCAITLL	0.02/0.49219949
	29	6	QERIGLFIV	0.00/0.50545109
	**30**	**32**	**AFLTATRLC**	**0.59/-0.093565614**
	31	52	VQPALYLYN	0.96/-0.54955225
	32	30	CMAFLTATR	0.00/0.37374539
	33	27	LLVCMAFLT	0.67/-0.8292502
	34	41	VQCMTGFNT	0.66/-0.85129807
	35	45	TGFNTLLVQ	0.90/-1.1351984
	36	34	LTATRLCVQ	0.68/-1.0467289
	37	70	QDSKPPLPP	0.99/-1.5643758
	38	67	VKFQDSKPP	0.79/-1.6396241
**HCoV-NL63**				
**Viral protein name**	**Rank**	**Start position**	**Sequence**	**Score (ANN/SVM)**
**Envelope**	1	30	MTFIKLIQL	0.85/1.1147816
	2	21	VMIFFFVLA	0.97/0.84537219
	3	12	VLNSILWLL	0.52/1.0318757
	4	5	LIDDNGIVL	0.89/0.42154725
	5	55	KIFLAYQDY	0.91/0.35324098
	6	19	LLVMIFFFV	0.48/0.70758604
	7	16	ILWLLVMIF	0.69/0.416904
	8	65	QIAPVPAEV	0.15/0.93282364
	9	37	QLCFTCHYF	0.57/0.49749676
	10	8	DNGIVLNSI	0.33/0.71998752
	11	24	FFFVLAMTF	0.70/0.34339497
	12	22	MIFFFVLAM	0.01/1.0168252
	13	48	RTLQPVYKI	0.09/0.9221873
	14	25	FFVLAMTFI	0.92/0.076178019
	15	4	RLIDDNGIV	0.06/0.88782061
	16	59	AYQDYMQIA	0.27/0.52539488
	17	43	HYFFSRTLQ	0.84/-0.04528681
	18	50	LQPVYKIFL	0.67/0.12251192
	19	28	LAMTFIKLI	0.97/-0.22110032
	20	27	VLAMTFIKL	0.01/0.7382466
	21	17	LWLLVMIFF	0.53/0.20745591
	22	66	IAPVPAEVL	/0.71392143
	23	49	TLQPVYKIF	0.01/0.60842696
	24	20	LVMIFFFVL	0.00/0.60981957
	25	13	LNSILWLLV	0.98/-0.45138175

	26	68	PVPAEVLNV	/0.52237808
	27	47	SRTLQPVYK	0.05/0.41973046
	28	34	KLIQLCFTC	0.02/0.37888815
	29	44	YFFSRTLQP	0.77/-0.47763152
	30	62	DYMQIAPVP	0.83/-0.76907218
**HCoV-229E**				
**Viral protein name**	**Rank**	**Start position**	**Sequence**	**Score (ANN/SVM)**
**Envelope**	1	16	LLWCVVLIV	0.97/1.4253784
	2	30	ITIIKLIKL	0.94/1.2826698
	3	7	DDHALVVNV	0.79/0.97984782
	4	66	HIDPFPKRV	0.37/1.176099
	5	12	VVNVLLWCV	0.95/0.50794883
	6	37	KLCFTCHMF	0.89/0.43601752
	7	14	NVLLWCVVL	0.94/0.24113564
	8	19	CVVLIVILL	0.26/0.76056346
	9	45	FCNRTVYGP	0.93/0.041517814
	10	15	VLLWCVVLI	0.18/0.79078298
	11	27	LVCITIIKL	0.04/0.88417935
	12	20	VVLIVILLV	0.08/0.8400338
	13	4	KLVDDHALV	0.46/0.45693719
	14	57	VYHIYQSYM	0.01/0.89704074
	15	41	TCHMFCNRT	0.78/0.038413593
	16	24	VILLVCITI	0.31/0.49818172
	17	59	HIYQSYMHI	0.13/0.64915513
	18	13	VNVLLWCVV	0.67/0.00080872534
	19	49	TVYGPIKNV	0.20/0.46726731
	20	56	NVYHIYQSY	0.13/0.45685054
	21	8	DHALVVNVL	0.01/0.53900496
	22	40	FTCHMFCNR	0.03/0.48649853
	23	52	GPIKNVYHI	0.00/0.51594748
	24	34	KLIKLCFTC	0.04/0.46911404
	25	18	WCVVLIVIL	0.03/0.44295609
	26	60	IYQSYMHID	0.95/-0.53151165
	27	55	KNVYHIYQS	0.93/-0.51524004
	28	25	ILLVCITII	0.00/0.3944171
	29	58	YHIYQSYMH	0.95/-0.59901297
	30	48	RTVYGPIKN	0.92/-0.58362683

	31	28	VCITIIKLI	0.51/-0.30207982
	32	61	YQSYMHIDP	0.83/-0.93658136
	33	33	IKLIKLCFT	0.56/-0.99293995
**HCoV-OC43**				
**Viral protein name**	**Rank**	**Start position**	**Sequence**	**Score (ANN/SVM)**
**Envelope**	1	7	YLADTVWYV	0.31/2.3652348
	2	55	SPSIYVFNR	0.84/1.0516232
	3	27	LVTIVVVAF	0.80/1.0279233
	4	23	AICLLVTIV	0.74/0.93439759
	5	52	LVLSPSIYV	0.99/0.66790173
	6	66	QFYEFYNDI	0.71/0.79282771
	7	24	ICLLVTIVV	0.86/0.62629457
	8	13	WYVGQIIFI	0.54/0.92308938
	9	32	VVAFLATFK	0.95/0.49439065
	10	73	DIKPPVLDV	0.75/0.67326274
	11	19	IFIVAICLL	0.82/0.53378928
	12	31	VVVAFLATF	0.84/0.36277806
	13	6	AYLADTVWY	0.86/0.32145213
	14	22	VAICLLVTI	0.92/0.21636364
	15	18	IIFIVAICL	0.01/1.1021567
	16	63	RGRQFYEFY	0.93/0.089834301
	17	59	YVFNRGRQF	0.61/0.37921737
	18	29	TIVVVAFLA	0.95/0.0087496811
	19	40	KLCIQLCGM	0.50/0.45029766
	20	46	CGMCNTLVL	0.93/0.0047116177
	21	37	ATFKLCIQL	0.04/0.8126547
	22	25	CLLVTIVVV	0.02/0.79108189
	23	14	YVGQIIFIV	0.00/0.75333868
	24	70	FYNDIKPPV	0.59/0.16051728
	25	58	IYVFNRGRQ	0.89/-0.15046971
	26	17	QIIFIVAIC	0.28/0.44484173
	27	38	TFKLCIQLC	0.66/0.063805722
	28	28	VTIVVVAFL	0.02/0.63313612
	29	67	FYEFYNDIK	0.64/-0.024399741
	30	16	GQIIFIVAI	0.00/0.61229884
	31	12	VWYVGQIIF	0.63/-0.022659664
	32	15	VGQIIFIVA	0.62/-0.017997626
	33	62	NRGRQFYEF	0.01/0.55313485

	34	60	VFNRGRQFY	0.00/0.53977627
	35	53	VLSPSIYVF	0.01/0.52058673
	36	11	TVWYVGQII	0.00/0.51642822
	37	43	IQLCGMCNT	0.72/-0.2621705
	38	35	FLATFKLCI	0.00/0.41596129
	39	33	VAFLATFKL	0.61/-0.22762835
	40	45	LCGMCNTLV	0.58/-0.52434988
	41	54	LSPSIYVFN	0.85/-1.000583
	42	72	NDIKPPVLD	0.65/-0.93329455
**HCoV-HKU1**				
**Viral protein name**	**Rank**	**Start position**	**Sequence**	**Score (ANN/SVM)**
**Envelope**	1	5	FFTDTAWYI	0.97/0.81010327
	2	25	IFLIFVVAL	0.68/1.0860147
	3	19	LVLSCVIFL	0.81/0.66262758
	4	12	YIGQIFFLV	0.89/0.40362005
	5	21	LSCVIFLIF	0.81/0.46884947
	6	11	WYIGQIFFL	0.18/1.0211905
	7	50	FIISPSAYV	0.69/0.48887938
	8	24	VIFLIFVVA	0.74/0.4330104
	9	22	SCVIFLIFV	0.01/1.1354362
	10	43	ICGFCNIFI	0.87/0.22423108
	11	41	IQICGFCNI	0.37/0.63052644
	12	26	FLIFVVALL	0.03/0.89957281
	13	59	YNRGRQLYK	0.00/0.90229452
	14	10	AWYIGQIFF	0.47/0.42991415
	15	53	SPSAYVVYNR	0.03/0.86586641
	16	14	GQIFFLVLS	0.97/-0.086289852
	17	52	ISPSAYVVYN	0.96/-0.1175764
	18	20	VLSCVIFLI	0.05/0.78040404
	19	44	CGFCNIFII	0.19/0.62693374
	20	27	LIFVVALLA	0.98/-0.16718043
	21	58	VYNRGRQLY	0.04/0.75198527
	22	70	SEHVIPSTL	0.29/0.38945586
	23	8	DTAWYIGQI	0.02/0.65013492
	24	35	ATIKLCIQI	0.00/0.66442936
	25	55	SAYVVYNRGR	0.98/-0.34168303
	26	1	MVDVFFTDT	0.55/0.064258598
	27	60	NRGRQLYKS	0.58/0.015687379

	28	48	NIFIISPSA	0.74/-0.15448485
	29	18	FLVLSCVIF	0.01/0.50903
	30	13	IGQIFFLVL	0.13/0.38814528
	31	3	DVFFTDTAW	0.01/0.46213887
	32	57	YVYNRGRQL	0.01/0.42747013
	33	73	VIPSTLDDL	/0.38726384
	34	7	TDTAWYIGQ	0.80/-0.75896706
	35	67	KSYSEHVIP	0.71/-0.69228345
**SARS-CoV-2**				
**Viral protein name**	**Rank**	**Start position**	**Sequence**	**Score (ANN/SVM)**
**Membrane**	1	38	AYANRNRFL	0.94/1.534005
	2	41	NRNRFLYII	0.96/1.2950944
	3	43	NRFLYIIKL	0.79/1.3086849
	4	15	KLLEQWNLV	0.54/1.1477859
	5	104	ARTRSMWSF	0.87/0.79456645
	6	21	NLVIGFLFL	0.30/1.3302649
	7	83	AMACLVGLM	0.96/0.62580832
	8	130	TRPLLESEL	0.01/1.5277131
	9	46	LYIIKLIFL	0.00/1.4599298
	10	39	YANRNRFLY	0.70/0.75749834
	11	37	FAYANRNRF	0.71/0.72206684
	12	99	SFRLFARTR	0.98/0.43485337
	13	136	SELVIGAVI	0.92/0.48139255
	14	50	KLIFLWLLW	0.97/0.41640893
	15	85	ACLVGLMWL	0.85/0.52376312
	16	172	TSRTLSYYK	0.68/0.67723726
	17	193	FAAYSRYRI	0.84/0.50777994
	18	112	FNPETNILL	0.41/0.92818671
	19	45	FLYIIKLIF	0.83/0.5058283
	20	61	TLACFVLAA	0.43/0.89317424
	21	196	YSRYRIGNY	0.91/0.39062916
	22	40	ANRNRFLYI	0.05/1.2284371
	23	34	LLQFAYANR	0.94/0.28716981
	24	198	RYRIGNYKL	0.05/1.1734506
	25	173	SRTLSYYKL	0.40/0.79618648
	26	92	WLSYFIASF	0.34/0.84325684
	27	96	FIASFRLFA	0.13/1.0490459
	28	49	IKLIFLWLL	0.53/0.6187025

	29	212	SSSDNIALL	/1.1372182
	30	5	NGTITVEEL	0.99/0.1463184
	31	192	GFAAYSRYR	0.80/0.32961293
	32	65	FVLAAVYRI	0.00/1.1177805
	33	168	ITVATSRTL	0.99/0.09512733
	34	55	WLLWPVTLA	0.65/0.40702879
	**35**	**94**	**SYFIASFRL**	**0.33/0.72395431**
	36	73	INWITGGIA	0.90/0.14883025
	37	52	IFLWLLWPV	0.91/0.13060966
	**38**	**111**	**SFNPETNIL**	**0.18/0.83545969**
	39	153	GHHLGRCDI	0.80/0.20706352
	40	42	RNRFLYIIK	0.00/1.0055272
	41	197	SRYRIGNYK	0.00/1.0036465
	42	142	AVILRGHLR	0.31/0.6842731
	43	48	IIKLIFLWL	0.25/0.73918596
	44	26	FLFLTWICL	0.28/0.70891806
	45	195	AYSRYRIGN	0.79/0.18502933
	46	74	NWITGGIAI	0.93/0.039965501
	47	171	ATSRTLSYY	0.00/0.94722714
	48	141	GAVILRGHL	0.84/0.095706564
	49	72	RINWITGGI	0.91/0.022493064
	50	68	AAVYRINWI	0.00/0.91891809
	51	27	LFLTWICLL	0.89/-0.0071357757
	52	98	ASFRLFART	0.41/0.46729682
	53	75	WITGGIAIA	0.77/0.098162084
	54	2	ADSNGTITV	0.68/0.1653799
	55	137	ELVIGAVIL	0.01/0.83354512
	56	138	LVIGAVILR	0.76/0.075370132
	57	148	HLRIAGHHL	0.10/0.73433113
	58	30	TWICLLQFA	0.94/-0.13203349
	59	116	TNILLNVPL	0.59/0.2051763
	60	56	LLWPVTLAC	0.62/0.17025753
	61	58	WPVTLACFV	0.27/0.51367294
	62	166	KEITVATSR	0.80/-0.040709
	63	101	RLFARTRSM	0.04/0.71755901
	64	211	SSSSDNIAL	/0.74666418
	65	88	VGLMWLSYF	0.75/-0.032093333
	66	24	IGFLFLTWI	0.93/-0.21772675

	67	8	ITVEELKKL	0.80/-0.095865837
	68	19	QWNLVIGFL	0.89/-0.18860719
	69	144	ILRGHLRIA	0.00/0.69453508
	70	178	YYKLGASQR	0.02/0.66064316
	71	79	GIAIAMACL	0.20/0.45983994
	72	126	GTILTRPLL	0.00/0.65466291
	73	194	AAYSRYRIG	0.08/0.55879723
	74	103	FARTRSMWS	1.00/-0.39487449
	75	36	QFAYANRNR	0.00/0.59525233
	76	102	LFARTRSMW	0.09/0.50026909
	77	186	RVAGDSGFA	0.97/-0.39718197
	**78**	**162**	**KDLPKEITV**	**0.12/0.44772671**
	79	76	ITGGIAIAM	0.03/0.52725066
	80	97	IASFRLFAR	0.01/0.52982343
	81	44	RFLYIIKLI	0.00/0.51353245
	82	134	LESELVIGA	0.00/0.51224851
	83	70	VYRINWITG	0.00/0.50995909
	84	64	CFVLAAVYR	0.01/0.49918463
	**85**	**188**	**AGDSGFAAY**	**0.06/0.43852094**
	86	154	HHLGRCDIK	0.62/-0.14339661
	87	119	LLNVPLHGT	0.99/-0.51433219
	88	69	AVYRINWIT	0.04/0.43528962
	89	67	LAAVYRINW	0.01/0.45284658
	90	176	LSYYKLGAS	0.99/-0.53726447
	91	201	IGNYKLNTD	0.70/-0.29728934
	92	113	NPETNILLN	0.97/-0.62630241
	93	179	YKLGASQRV	0.55/-0.22732784
	94	87	LVGLMWLSY	0.62/-0.37756975
	95	35	LQFAYANRN	0.98/-0.73842493
	96	7	TITVEELKK	0.79/-0.58360765
	97	81	AIAMACLVG	0.64/-0.44634314
	98	203	NYKLNTDHS	0.54/-0.35367928
	99	127	TILTRPLLE	0.60/-0.41466275
	100	108	SMWSFNPET	0.60/-0.45458388
	101	152	AGHHLGRCD	0.83/-0.69033051
	102	164	LPKEITVAT	0.55/-0.57245695
	103	118	ILLNVPLHG	0.57/-0.6114191
	104	156	LGRCDIKDL	0.80/-0.87765572
	105	165	PKEITVATS	0.74/-1.0843754

	106	133	LLESELVIG	0.71/-1.2327109
**SARS-CoV**				
**Viral protein name**	**Rank**	**Start position**	**Sequence**	**Score (ANN/SVM)**
**Membrane**	1	95	FVASFRLFA	0.68/1.2821694
	2	41	RNRFLYIIK	0.93/1.0055272
	3	25	FLFLAWIML	0.94/0.87487652
	4	196	NRYRIGNYK	0.92/0.87277329
	5	125	GTIVTRPLM	0.88/0.9095859
	6	64	FVLAAVYRI	0.64/1.1177805
	7	40	NRNRFLYII	0.40/1.2950944
	8	14	QLLEQWNLV	0.82/0.68995958
	9	100	RLFARTRSM	0.79/0.71755901
	10	37	AYSNRNRFL	0.09/1.3992408
	11	122	PLRGTIVTR	0.92/0.52673674
	12	1	MADNGTITV	0.69/0.72183355
	13	97	ASFRLFART	0.94/0.46729682
	14	129	TRPLMESEL	0.29/1.0952626
	15	20	NLVIGFLFL	0.01/1.3302649
	16	38	YSNRNRFLY	0.33/0.99584681
	17	42	NRFLYIIKL	0.01/1.3086849
	18	197	RYRIGNYKL	0.13/1.1734506
	19	61	LACFVLAAV	0.95/0.3289888
	20	88	GLMWLSYFV	0.77/0.50853016
	21	172	SRTLSYYKL	0.48/0.79618648
	22	211	GSNDNIALL	/1.2503836
	23	74	WVTGGIAIA	0.94/0.29104438
	24	177	YYKLGASQR	0.51/0.66064316
	25	193	AAYNRYRIG	0.92/0.22922506
	26	202	NYKLNTDHA	0.91/0.21813252
	27	143	IIRGHLRMA	0.87/0.19978509
	28	5	GTITVEELK	0.97/0.09780617
	29	36	FAYSNRNRF	0.03/1.0373734
	30	21	LVIGFLFLA	0.76/0.28726772
	31	8	TVEELKQLL	0.54/0.49652662
	32	39	SNRNRFLYI	0.10/0.92906309
	33	92	LSYFVASFR	0.80/0.2121516
	**34**	**187**	**GTDSGFAAY**	**0.24/0.75167131**
	35	138	VIGAVIIRG	0.96/0.027776638

	36	170	ATSRTLSYY	0.02/0.94722714
	37	84	ACIVGLMWL	0.00/0.95329943
	38	91	WLSYFVASF	0.23/0.72279954
	39	165	KEITVATSR	0.99/-0.040709
	40	111	FNPETNILL	0.02/0.92818671
	41	136	ELVIGAVII	0.67/0.26083068
	42	45	LYIIKLVFL	0.08/0.84998052
	**43**	**161**	**KDLPKEITV**	**0.48/0.44772671**
	44	159	DIKDLPKEI	0.99/-0.064095269
	45	133	MESELVIGA	0.07/0.85535516
	46	60	TLACFVLAA	0.01/0.89317424
	47	54	WLLWPVTLA	0.48/0.40702879
	**48**	**110**	**SFNPETNIL**	**0.05/0.83545969**
	49	192	FAAYNRYRI	0.01/0.86589126
	50	48	IKLVFLWLL	0.11/0.76135453
	51	195	YNRYRIGNY	0.57/0.26868248
	52	47	IIKLVFLWL	0.05/0.76608405
	53	103	ARTRSMWSF	0.01/0.79456645
	54	75	VTGGIAIAM	0.22/0.58328221
	55	19	WNLVIGFLF	0.74/0.058311061
	56	141	AVIIRGHLR	0.02/0.76535018
	57	119	LNVPLRGTI	0.92/-0.14422858
	58	189	DSGFAAYNR	0.22/0.53397851
	59	116	NILLNVPLR	0.11/0.61971381
	60	194	AYNRYRIGN	0.53/0.19031694
	61	101	LFARTRSMW	0.22/0.50026909
	62	120	NVPLRGTIV	0.05/0.66206936
	63	171	TSRTLSYYK	0.03/0.67723726
	64	13	KQLLEQWNL	0.03/0.66756564
	65	86	IVGLMWLSY	0.85/-0.16115119
	66	67	AAVYRINWV	0.11/0.4817265
	67	49	KLVFLWLLW	0.08/0.49584252
	68	178	YKLGASQRV	0.77/-0.22732784
	69	28	LAWIMLLQF	0.55/-0.016850555
	70	63	CFVLAAVYR	0.03/0.49918463
	71	154	SLGRCDIKD	0.97/-0.44256032
	72	57	WPVTLACFV	0.01/0.51367294
	**73**	**93**	**SYFVASFRL**	**0.01/0.49716475**
	74	135	SELVIGAVI	0.02/0.48139255

	75	69	VYRINWVTG	0.00/0.48186639
	76	145	RGHLRMAGH	0.12/0.36179993
	77	76	TGGIAIAMA	0.84/-0.36138282
	78	99	FRLFARTRS	0.66/-0.18296228
	79	82	AMACIVGLM	0.00/0.4659084
	80	98	SFRLFARTR	0.03/0.43485337
	81	44	FLYIIKLVF	0.01/0.45288171
	82	66	LAAVYRINW	0.00/0.45284658
	83	53	LWLLWPVTL	0.98/-0.52996139
	84	2	ADNGTITVE	0.99/-0.5458222
	85	78	GIAIAMACI	0.01/0.41953955
	86	96	VASFRLFAR	0.02/0.39749627
	87	140	GAVIIRGHL	0.00/0.41030383
	88	32	MLLQFAYSN	0.75/-0.34359439
	89	149	RMAGHSLGR	0.52/-0.1219183
	90	58	PVTLACFVL	0.71/-0.34015016
	91	89	LMWLSYFVA	0.73/-0.36412405
	92	200	IGNYKLNTD	0.63/-0.29728934
	93	72	INWVTGGIA	0.67/-0.38085986
	94	112	NPETNILLN	0.89/-0.62630241
	95	16	LEQWNLVIG	0.72/-0.49429055
	96	3	DNGTITVEE	0.97/-0.74767274
	97	15	LLEQWNLVI	0.83/-0.62161766
	98	139	IGAVIIRGH	0.84/-0.70317598
	99	157	RCDIKDLPK	0.95/-0.82975361
	100	155	LGRCDIKDL	0.99/-0.87765572
	101	77	GGIAIAMAC	0.64/-0.5324313
	102	121	VPLRGTIVT	0.53/-0.42807568
	103	107	SMWSFNPET	0.55/-0.45458388
	104	50	LVFLWLLWP	0.86/-0.88953572
	105	203	YKLNTDHAG	0.59/-0.69082897
	106	90	MWLSYFVAS	0.64/-0.81459416
	107	201	GNYKLNTDH	0.99/-1.1881523
	108	148	LRMAGHSLG	0.57/-0.78018021
	109	144	IRGHLRMAG	0.52/-0.76672312
	110	146	GHLRMAGHS	0.97/-1.2795936
	111	176	SYYKLGASQ	0.54/-0.90215334
**MERS-CoV**				

Viral protein name	Rank	Start position	Sequence	Score (ANN/SVM)
**Membrane**	**1**	**161**	**DRLPNEVTV**	**0.90/1.3325897**
	2	170	AKPNVLIAL	0.99/1.112663
	3	92	ISYFVQSIR	0.84/0.9756516
	4	138	SVTAVVTNG	0.96/0.50240003
	5	20	NFAWSLIFL	0.02/1.3750567
	6	15	IIKDWNFAW	0.76/0.63209891
	**7**	**187**	**GTNSGVAIY**	**0.29/1.043693**
	8	22	AWSLIFLLI	0.80/0.4916732
	9	38	YPSRSMTVY	0.93/0.30431911
	10	44	TVYVFKMFV	0.29/0.9305296
	11	133	VEDSTSVTA	0.74/0.47422488
	12	61	MALSIFSAV	0.66/0.5519665
	13	91	WISYFVQSI	0.62/0.58502457
	14	171	KPNVLIALK	0.67/0.48465964
	15	163	LPNEVTVAK	0.63/0.51851449
	**16**	**93**	**SYFVQSIRL**	**0.01/1.1261978**
	17	124	GGTTVVRPL	0.67/0.39598193
	18	189	NSGVAIYHR	0.33/0.73296251
	19	175	LIALKMVKR	0.92/0.11586787
	20	191	GVAIYHRYK	0.02/1.0027127
	21	24	SLIFLLITI	0.06/0.9604213
	22	64	SIFSAVYPI	0.76/0.25040794
	23	76	SQIISGIVA	0.75/0.2054042
	24	95	FVQSIRLFM	0.29/0.64863165
	25	41	RSMTVYVFK	0.64/0.29572551
	26	19	WNFAWSLIF	0.99/-0.059008351
	27	188	TNSGVAIYH	0.15/0.7426577
	28	7	LTEAQIIAI	0.96/-0.075540835
	29	155	FGACDYDRL	0.99/-0.12423854
	30	159	DYDRLPNEV	0.14/0.69761321
	31	45	VYVFKMFVL	0.02/0.803342
	32	26	IFLLITIVL	0.02/0.7820036
	33	47	VFKMFVLWL	0.36/0.42789595
	34	200	AGNYRSPPI	0.84/-0.068816791
	35	209	TADIELALL	/0.76173539
	36	111	FNPETNCLL	0.20/0.55943845
	37	86	VSAMMWISY	0.72/0.037550757
	38	21	FAWSLIFLL	0.11/0.61443046

	39	37	GYPSRSMTV	0.05/0.65965946
	40	5	TQLTEAQII	0.88/-0.17703249
	41	8	TEAQIIAII	0.04/0.63874655
	42	120	NVPFGGTTV	0.01/0.66645184
	43	66	FSAVYPIDL	0.01/0.6514081
	44	193	AIYHRYKAG	0.70/-0.038623293
	45	154	HFGACDYDR	0.92/-0.26473748
	**46**	**110**	**SFNPETNCL**	**0.05/0.6027168**
	47	55	LLWPSSMAL	0.10/0.54332774
	48	167	VTVAKPNVL	0.53/0.099630592
	49	4	MTQLTEAQI	0.58/0.04746237
	50	88	AMMWISYFV	0.25/0.37056163
	51	196	HRYKAGNYR	0.15/0.46866787
	52	202	NYRSPPITA	/0.60523431
	53	184	QSYGTNSGV	0.82/-0.21985573
	54	122	PFGGTTVVR	0.78/-0.19356706
	55	152	GMHFGACDY	0.59/-0.010658696
	56	116	NCLLNVPFG	0.53/0.024311399
	57	57	WPSSMALSI	0.04/0.51276495
	58	147	HLKMAGMHF	0.10/0.44982024
	59	204	RSPPITADI	/0.53618799
	60	121	VPFGGTTVV	0.06/0.46007594
	61	168	TVAKPNVLI	0.01/0.49683601
	62	62	ALSIFSAVY	0.00/0.50497952
	63	208	ITADIELAL	/0.50382266
	64	50	MFVLWLLWP	1.00/-0.49992406
	65	13	IAIIKDWNF	0.03/0.46314214
	66	141	AVVTNGHLK	0.00/0.48952038
	67	149	KMAGMHFGA	0.68/-0.20106563
	68	14	AIIKDWNFA	0.69/-0.21587273
	69	169	VAKPNVLIA	0.06/0.4127837
	70	42	SMTVYVFKM	0.00/0.47270089
	71	17	KDWNFAWSL	0.00/0.41420002
	72	143	VTNGHLKMA	0.68/-0.3130819
	73	74	LASQIISGI	0.72/-0.39172178
	74	99	IRLFMRTGS	0.85/-0.61346618
	75	23	WSLIFLLIT	0.78/-0.55058213
	76	54	WLLWPSSMA	0.77/-0.6485796
	77	119	LNVPFGGTT	0.98/-0.88103088

	78	32	IVLQYGYPS	0.95/-0.85753772
	79	127	TVVRPLVED	0.66/-0.58089465
	80	90	MWISYFVQS	0.81/-0.79029278
	81	112	NPETNCLLN	0.83/-0.81810302
	82	153	MHFGACDYD	0.53/-0.57221429
	83	157	ACDYDRLPN	0.93/-1.234207
	84	146	GHLKMAGMH	0.91/-1.2450366
	85	176	IALKMVKRQ	0.52/-0.98755935
	86	165	NEVTVAKPN	0.58/-1.1145372
	87	56	LWPSSMALS	0.75/-1.2934473
**HCoV-NL63**				
**Viral protein name**	**Rank**	**Start position**	**Sequence**	**Score (ANN/SVM)**
**Membrane**	1	61	ALSIFDCFV	0.42/1.3269396
	2	27	TVFIVVLQY	0.96/0.75983923
	3	39	KYSRLLYGL	0.09/1.6269526
	4	194	GWAFYVRAK	0.61/1.1032724
	5	143	TLLSGVLLV	0.54/1.0866306
	6	72	NVDWVFFGF	0.93/0.6902797
	7	68	FVNFNVDWV	0.17/1.3164491
	8	103	WRRVKTFWA	0.99/0.41672656
	9	24	LILTVFIVV	0.74/0.62932871
	10	112	FNPETNAII	0.99/0.36572179
	11	23	NLILTVFIV	0.05/1.2803939
	12	163	GQLPKYVIV	0.28/1.0441602
	13	217	SEREKLLHL	/1.2478922
	14	134	MAAPTGVTL	0.01/1.2020559
	15	54	CLWPLVLAL	0.00/1.2025543
	16	15	LRNWNFSWN	0.91/0.29116747
	17	193	TGWAFYVRA	0.76/0.43350684
	18	43	LLYGLKMSV	0.99/0.19587974
	19	92	WVMYFVNSF	0.20/0.97214623
	20	50	SVLWCLWPL	0.66/0.50112474
	21	46	GLKMSVLWC	0.81/0.32713326
	22	41	SRLLYGLKM	0.58/0.55680081
	23	208	GVASQEGVL	0.88/0.23328951
	24	117	NAIISLQVY	0.79/0.32261677
	25	19	NFSWNLILT	0.95/0.13441361
	26	88	TLCLWVMYF	0.46/0.60965105

	27	96	FVNSFRLWR	0.03/1.0157158
	28	25	ILTVFIVVL	0.08/0.95244546
	29	31	VVLQYGHYK	0.74/0.28403801
	30	104	RRVKTFWAF	0.47/0.5453457
	31	21	SWNLILTVF	0.76/0.21671489
	32	191	SQTGWAFYV	0.00/0.95134123
	33	93	VMYFVNSFR	0.79/0.12575145
	34	76	VFFGFSILM	0.32/0.58884796
	35	60	LALSIFDCF	0.70/0.20465324
	36	101	RLWRRVKTF	0.01/0.89108233
	37	47	LKMSVLWCL	1.00/-0.1038001
	38	192	QTGWAFYVR	0.30/0.58530988
	39	5	SVPLSEVYV	0.00/0.88332248
	40	35	YGHYKYSRL	0.47/0.40854334
	41	98	NSFRLWRRV	0.18/0.69216339
	42	152	DGHKIATRV	0.93/-0.058796914
	43	190	TSQTGWAFY	0.16/0.71048752
	44	57	PLVLALSIF	0.96/-0.090172178
	45	22	WNLILTVFI	0.60/0.26016217
	46	97	VNSFRLWRR	0.19/0.66920512
	47	51	VLWCLWPLV	0.00/0.85091173
	48	81	SILMSIITL	0.06/0.78407937
	49	7	PLSEVYVHL	0.33/0.50358441
	50	10	EVYVHLRNW	0.76/0.06737552
	**51**	**94**	**MYFVNSFRL**	**0.00/0.81397405**
	52	16	RNWNFSWNL	0.00/0.78986837
	53	12	YVHLRNWNF	0.02/0.75910697
	54	95	YFVNSFRLW	0.93/-0.16742967
	55	99	SFRLWRRVK	0.03/0.73100482
	56	155	KIATRVQVG	0.79/-0.041900185
	57	70	NFNVDWVFF	0.01/0.7324852
	58	136	APTGVTLTL	0.13/0.61061023
	59	4	SSVPLSEVY	0.96/-0.22283825
	60	172	ATPSTTIVC	0.15/0.57706808
	61	6	VPLSEVYVH	0.77/-0.04931276
	62	128	NYYLPVMAA	0.30/0.41639443
	63	89	LCLWVMYFV	0.01/0.69801811
	64	175	STTIVCDRV	0.16/0.5326677
	65	201	AKHGDFSGV	0.58/0.087165888

	66	189	ETSQTGWAF	0.22/0.44474166
	67	63	SIFDCFVNF	0.10/0.54780813
	68	75	WVFFGFSIL	0.12/0.52189011
	69	176	TTIVCDRVG	0.91/-0.27882271
	70	67	CFVNFNVDW	0.66/-0.042369151
	71	182	RVGRSVNET	0.64/-0.022524297
	72	129	YYLPVMAAP	0.18/0.42472047
	73	111	AFNPETNAI	0.01/0.57068859
	74	73	VDWVFFGFS	0.95/-0.38416166
	75	102	LWRRVKTFW	0.88/-0.33399253
	76	79	GFSILMSII	0.14/0.40177837
	77	44	LYGLKMSVL	0.05/0.48935835
	78	36	GHYKYSRLL	0.60/-0.063998079
	79	157	ATRVQVGQL	0.04/0.4959503
	80	119	IISLQVYGH	0.78/-0.25341414
	81	38	YKYSRLLYG	0.54/-0.038425177
	82	20	FSWNLILTV	0.55/-0.055016499
	83	87	ITLCLWVMY	0.00/0.49009803
	84	71	FNVDWVFFG	0.00/0.47647061
	85	123	QVYGHNYYL	0.02/0.43768443
	86	32	VLQYGHYKY	0.76/-0.34582979
	87	13	VHLRNWNFS	0.92/-0.5134316
	88	211	SQEGVLSER	/0.40520158
	89	82	ILMSIITLC	0.68/-0.33428279
	90	108	TFWAFNPET	0.93/-0.58450234
	91	174	PSTTIVCDR	0.64/-0.40569235
	92	207	SGVASQEGV	0.87/-0.68502309
	93	33	LQYGHYKYS	0.82/-0.70260336
	94	114	PETNAIISL	0.57/-0.51338289
	95	109	FWAFNPETN	0.52/-0.52429078
	96	185	RSVNETSQT	0.87/-0.90286
	97	11	VYVHLRNWN	0.51/-0.54535812
	98	181	DRVGRSVNE	0.63/-0.71633178
	99	184	GRSVNETSQ	0.83/-0.95171498
	100	45	YGLKMSVLW	0.70/-0.84469286
	101	133	VMAAPTGVT	0.69/-1.0697739
	102	106	VKTFWAFNP	0.53/-1.0908489
	103	138	TGVTLTLLS	0.78/-1.4291627
**HCoV-229E**				

Viral protein name	Rank	Start position	Sequence	Score (ANN/SVM)
**Membrane**	1	53	LLWPLVLAL	0.90/1.0172013
	2	207	AVSSPMSNM	0.65/1.1315979
	3	100	RLFRRARTF	0.69/1.0834146
	4	43	FYGLKMLVL	0.73/1.0416194
	5	62	SIFDTWANW	0.86/0.86647004
	6	69	NWDSNWAFV	0.77/0.91060155
	7	45	GLKMLVLWL	0.85/0.80022528
	8	38	KYSRLFYGL	0.07/1.5660843
	9	153	HRLASGVQV	0.98/0.59855888
	10	50	VLWLLWPLV	0.98/0.58891615
	11	24	ILTIFIVIL	0.06/1.4954772
	12	22	NVILTIFIV	0.74/0.8008092
	13	162	HNLPEYMTV	0.72/0.81596502
	14	178	IYSRVGRSV	0.57/0.93221588
	15	23	VILTIFIVI	0.98/0.49982791
	16	41	RLFYGLKML	0.93/0.5402551
	17	171	AVPSTTIIY	0.48/0.93651537
	18	199	RVKHGDFSA	0.90/0.50488593
	19	40	SRLFYGLKM	0.69/0.7118252
	20	16	NWNFGWNVI	0.70/0.69004979
	21	142	TLLSGVLYV	0.03/1.3396451
	22	200	VKHGDFSAV	0.99/0.36784466
	23	103	RRARTFWAW	0.19/1.1631307
	24	71	DSNWAFVAF	0.02/1.1416897
	25	55	WPLVLALSI	0.98/0.15944072
	26	154	RLASGVQVH	0.67/0.4606884
	27	21	WNVILTIFI	0.82/0.30367738
	28	181	RVGRSVNSQ	0.98/0.14283932
	29	52	WLLWPLVLA	0.82/0.294418
	30	189	QNSTGWVFY	0.76/0.32083315
	31	192	TGWVFYVRV	0.31/0.76078895
	32	7	TGDIVTHLK	0.82/0.24707595
	33	92	VMYFANSFR	0.97/0.072860827
	34	133	QQAPTGITV	0.14/0.86735107
	35	98	SFRLFRRAR	0.12/0.87329753
	36	84	AVSTLVMWV	0.00/0.97860228
	37	177	IIYSRVGRS	0.98/-0.0067330453

	38	216	TENERLLHF	/0.95511253
	39	6	CTGDIVTHL	0.42/0.53178046
	40	76	FVAFSFLMA	0.16/0.78893424
	41	91	WVMYFANSF	0.19/0.73912126
	**42**	**93**	**MYFANSFRL**	**0.23/0.69655934**
	43	29	IVILQFGHY	0.95/-0.026529684
	44	135	APTGITVTL	0.00/0.90960118
	45	97	NSFRLFRRA	0.06/0.8416029
	46	96	ANSFRLFRR	0.01/0.88298762
	47	47	KMLVLWLLW	0.87/0.021222742
	48	121	TTVLGQTYY	0.80/0.079188078
	49	156	ASGVQVHNL	0.25/0.62625185
	50	26	TIFIVILQF	0.07/0.80272298
	51	151	DGHRLASGV	0.90/-0.029760481
	52	122	TVLGQTYYQ	0.87/-0.0033528359
	53	112	NPEVNAITV	0.01/0.85169738
	54	107	TFWAWNPEV	0.75/0.099752668
	55	159	VQVHNLPEY	0.92/-0.091567921
	56	95	FANSFRLFR	0.07/0.75279398
	57	115	VNAITVTTV	0.03/0.78849761
	58	176	TIIYSRVGR	0.74/0.075212733
	59	15	KNWNFGWNV	0.17/0.63687585
	60	110	AWNPEVNAI	0.00/0.76448507
	61	102	FRRARTFWA	0.00/0.73206915
	62	214	NMTENERLL	/0.7228851
	63	60	ALSIFDTWA	0.02/0.70106824
	64	74	WAFVAFSFL	0.04/0.67644523
	65	81	FLMAVSTLV	0.29/0.38369605
	66	147	VLYVDGHRL	0.56/0.087799519
	67	99	FRLFRRART	0.25/0.394557
	68	191	STGWVFYVR	0.19/0.44185124
	69	73	NWAFVAFSF	0.12/0.50684428
	70	193	GWVFYVRVK	0.01/0.61394467
	71	30	VILQFGHYK	0.18/0.38876175
	72	169	TVAVPSTTI	0.16/0.38178865
	73	58	VLALSIFDT	0.60/-0.083623343
	74	163	NLPEYMTVA	0.77/-0.27216171
	75	188	SQNSTGWVF	0.04/0.45088961

	76	11	VTHLKNWNF	0.00/0.49021607
	77	68	ANWDSNWAF	0.00/0.48682911
	78	126	QTYYQPIQQ	0.77/-0.2954456
	79	87	TLVMWVMYF	0.01/0.46406701
	80	143	LLSGVLYVD	0.70/-0.25662713
	81	168	MTVAVPSTT	1.00/-0.56747993
	82	34	FGHYKYSRL	0.00/0.42878079
	83	139	ITVTLLSGV	0.71/-0.30838919
	84	184	RSVNSQNST	0.99/-0.60372976
	85	120	VTTVLGQTY	0.97/-0.6509589
	86	203	GDFSAVSSP	0.98/-0.70057856
	87	10	IVTHLKNWN	0.88/-0.60266118
	88	119	TVTTVLGQT	0.97/-0.75396264
	89	187	NSQNSTGWV	0.69/-0.52883165
	90	186	VNSQNSTGW	0.74/-0.5944038
	91	183	GRSVNSQNS	0.55/-0.43389174
	92	82	LMAVSTLVM	0.55/-0.44613325
	93	165	PEYMTVAVP	0.95/-0.91303044
	94	175	TTIIYSRVG	0.92/-0.91126139
	95	123	VLGQTYYQP	0.70/-0.69320026
	96	5	NCTGDIVTH	0.61/-0.68737295
	97	57	LVLALSIFD	0.80/-0.9154223
	98	118	ITVTTVLGQ	0.51/-0.81357846
	99	152	GHRLASGVQ	0.69/-1.0067898
	100	205	FSAVSSPMS	0.82/-1.1698711
	101	131	PIQQAPTGI	0.64/-1.2193975
	102	144	LSGVLYVDG	0.77/-1.4398635
**HCoV-OC43**				
**Viral protein name**	**Rank**	**Start position**	**Sequence**	**Score (ANN/SVM)**
**Membrane**	1	204	KSKVGNYRL	0.87/1.0800295
	2	153	HLYIQGIKL	0.91/0.99884889
	3	101	FVNSIRLFI	0.72/1.0052845
	4	98	IVYFVNSIR	0.72/0.97294785
	5	88	VFTIVAIIM	0.89/0.77527236
	6	202	YVKSKVGNY	0.01/1.5368575
	7	32	ILLFITIIL	0.82/0.67852814
	8	84	GLSIVFTIV	0.00/1.4977617
	9	195	DTSGFAVYV	0.01/1.4556836

	10	196	TSGFAVYVK	0.82/0.64395629
	11	117	FNPETNNLM	0.76/0.65010619
	12	75	VYALNNVYL	0.08/1.3192874
	**13**	**99**	**VYFVNSIRL**	**0.19/1.1841596**
	14	90	TIVAIIMWI	0.67/0.687161
	15	47	RSMFVYYVIK	0.45/0.89755685
	16	13	WTADEAIKF	0.43/0.91282428
	17	168	ADLPAYMTV	0.65/0.68062847
	18	26	NFSLGIILL	0.52/0.75929494
	19	76	YALNNVYLG	0.99/0.28536806
	20	106	RLFIRTGSF	0.86/0.38885643
	21	57	IILWLMWPL	0.78/0.45035621
	22	51	VYVIKMIIL	0.00/1.2206353
	23	53	VIKMIILWL	0.91/0.31035067
	24	5	TTPAPVYIW	0.65/0.56357336
	25	166	SLADLPAYM	0.25/0.93801696
	26	28	SLGIILLFI	0.32/0.86143284
	27	185	YKRGFLDRI	0.87/0.30619036
	28	139	IEDYHTLTV	0.83/0.3012014
	29	109	IRTGSFWSF	0.58/0.54674803
	30	121	TNNLMCIDM	0.54/0.5838765
	31	91	IVAIIMWIV	0.00/1.1008378
	32	87	IVFTIVAII	0.22/0.86440487
	33	97	WIVYFVNSI	0.31/0.73599333
	34	45	TSRSMFVYV	0.67/0.375493
	35	41	QFGYTSRSM	0.85/0.16598633
	36	61	LMWPLTIIL	0.30/0.68843628
	37	107	LFIRTGSFW	0.97/0.0054854584
	38	4	KTTPAPVYI	0.49/0.45991837
	39	46	SRSMFVYVI	0.33/0.61400396
	40	116	SFNPETNNL	0.07/0.8711914
	41	73	NCVYALNNV	0.19/0.73995041
	42	48	SMFVYVIKM	0.18/0.7496604
	43	147	VTIIRGHLY	0.63/0.29023927
	44	141	DYHTLTVTI	0.14/0.76679025
	45	44	YTSRSMFVY	0.52/0.38320157
	46	50	FVYVIKMII	0.55/0.34820031
	47	148	TIIRGHLYI	0.62/0.27756513
	48	70	TIFNCVYAL	0.03/0.86500586

	49	43	GYTSRSMFV	0.23/0.62312688
	50	60	WLMWPLTII	0.26/0.5798484
	51	86	SIVFTIVAI	0.33/0.50061561
	52	128	DMKGTMYVR	0.52/0.31026034
	53	81	VYLGLSIVF	0.70/0.12251951
	54	63	WPLTIILTI	0.00/0.81833073
	55	14	TADEAIKFL	0.23/0.58809773
	56	77	ALNNVYLGL	0.01/0.80614231
	57	80	NVYLGLSIV	0.02/0.78781589
	58	222	DTALLRNNI	/0.74298675
	59	131	GTMYVRPII	0.06/0.68201434
	60	6	TPAPVYIWT	0.01/0.69695802
	61	71	IFNCVYALN	0.91/-0.21001346
	62	27	FSLGIILLF	0.16/0.47991135
	63	162	GTGYSLADL	0.00/0.62454384
	64	171	PAYMTVAKV	0.59/0.029421572
	65	89	FTIVAIIMW	0.01/0.59649437
	66	102	VNSIRLFIR	0.00/0.60407438
	67	16	DEAIKFLKE	0.60/-0.0035159999
	68	83	LGLSIVFTI	0.08/0.51618789
	69	156	IQGIKLGTG	0.92/-0.32930791
	70	67	IILTIFNCV	0.20/0.38686145
	71	23	KEWNFSLGI	0.11/0.46300213
	72	52	YVIKMIILW	0.59/-0.02569042
	73	33	LLFITIILQ	0.96/-0.39815528
	74	198	GFAVYVKSK	0.01/0.54861816
	75	30	GIILLFITI	0.00/0.55003067
	76	170	LPAYMTVAK	0.90/-0.36265672
	77	64	PLTIILTIF	0.74/-0.20273025
	78	2	SSKTTPAPV	0.10/0.43423301
	79	79	NNVYLGLSI	0.90/-0.38064983
	80	125	MCIDMKGTM	0.92/-0.40415557
	81	165	YSLADLPAY	0.80/-0.28568776
	82	113	SFWSFNPET	0.96/-0.44918176
	83	93	AIIMWIVYF	0.01/0.49439466
	84	39	ILQFGYTSR	0.51/-0.0063597186
	85	94	IIMWIVYFV	0.00/0.49695658

	86	118	NPETNNLMC	0.99/-0.52216283
	87	82	YLGLSIVFT	0.99/-0.52451338
	88	25	WNFSLGIIL	0.03/0.43323177
	89	21	FLKEWNFSL	0.05/0.38922126
	90	194	SDTSGFAVY	0.70/-0.26941699
	91	184	TYKRGFLDR	0.03/0.38589956
	92	186	KRGFLDRIS	0.97/-0.56233208
	93	209	NYRLPSTQK	0.00/0.39768487
	94	143	HTLTVTIIR	0.02/0.37679545
	95	167	LADLPAYMT	0.98/-0.58678549
	96	31	IILLFITII	0.01/0.38045256
	97	178	KVTHLCTYK	0.01/0.37415561
	98	103	NSIRLFIRT	0.00/0.37253474
	99	142	YHTLTVTII	0.60/-0.23463653
	100	172	AYMTVAKVT	0.76/-0.40286962
	101	12	IWTADEAIK	0.65/-0.41066048
	102	182	LCTYKRGFL	0.85/-0.6361775
	103	133	MYVRPIIED	0.61/-0.40004934
	104	37	TIILQFGYT	0.86/-0.67178448
	105	108	FIRTGSFWS	0.88/-0.72882034
	106	200	AVYYVKSKVG	0.92/-0.82483856
	107	65	LTIILTIFN	0.98/-1.0027422
	108	138	IIEDYHTLT	0.95/-1.0002105
	109	150	IRGHLYIQG	0.65/-0.71544181
	110	145	LTVTIIRGH	0.84/-1.0417056
	111	7	PAPVYIWTA	0.53/-0.81669081
	112	197	SGFAVYVKS	0.62/-0.95789293
	113	40	LQFGYTSRS	0.54/-1.0393013
	114	124	LMCIDMKGT	0.63/-1.2106251
	115	29	LGIILLFIT	0.60/-1.3935892
**HCoV-HKU1**				
**Viral protein name**	**Rank**	**Start position**	**Sequence**	**Score (ANN/SVM)**
**Membrane**	1	161	TLSDLPVYV	0.82/1.6426512
	2	96	FVNSIRLFI	0.86/1.0052845
	3	75	NAFLAFSIV	0.96/0.88310432
	4	25	GVILLFITI	0.89/0.83050669
	5	111	SFNPETNNL	0.80/0.8711914
	6	163	SDLPVYVTV	0.59/1.0252376

	7	112	FNPETNNLM	0.96/0.65010619
	8	38	GYTSRSMFV	0.98/0.62312688
	9	79	AFSIVFTII	0.73/0.86282911
	10	136	DYHTLTATV	0.70/0.82891884
	11	86	IISIVIWIL	0.06/1.4653727
	12	1	MNKSFFPQF	0.91/0.54186681
	13	72	ALNNAFLAF	0.88/0.56512118
	14	41	SRSMFVYLI	0.81/0.61535157
	15	14	ATFLKEWNF	0.16/1.2182794
	16	21	NFSLGVILL	0.75/0.62512415
	17	63	TLTIFNCFY	0.99/0.37497442
	18	62	ITLTIFNCF	0.78/0.57908849
	19	84	FTIISIVIW	0.60/0.74460738
	20	104	IRTGSWWSF	0.87/0.41438112
	21	70	FYALNNAFL	0.01/1.241901
	22	43	SMFVYLIKM	0.68/0.57181132
	23	197	FVKSKVGNY	0.00/1.21182
	24	179	TYKRAFLDK	0.94/0.27057003
	25	94	LYFVNSIRL	0.55/0.61934416
	26	141	TATVIRGHL	0.85/0.31455757
	27	99	SIRLFIRTG	0.99/0.16760057
	28	74	NNAFLAFSI	0.36/0.78130362
	29	189	DVNSGFAVF	0.03/1.1105628
	30	148	HLYIQGVKL	0.24/0.89431328
	31	85	TIISIVIWI	0.21/0.92006925
	32	98	NSIRLFIRT	0.75/0.37253474
	33	123	DMKGKMFVR	0.08/1.0423894
	34	199	KSKVGNYRL	0.04/1.0800295
	35	81	SIVFTIISI	0.18/0.86866348
	36	65	TIFNCFYAL	0.00/0.99832674
	37	190	VNSGFAVFV	0.03/0.91457699
	38	46	VYLIKMIIL	0.00/0.92964441
	39	69	CFYALNNAF	1.00/-0.071757212
	40	83	VFTIISIVI	0.63/0.27647494
	41	157	GTGYTLSDL	0.01/0.89257713
	42	12	DQATFLKEW	0.84/0.055260038
	43	23	SLGVILLFI	0.04/0.82424265
	44	50	KMIILWLMW	0.93/-0.074516348
	45	211	KPSGMDTAL	/0.8355206

	46	102	LFIRTGSWW	0.69/0.13616864
	47	82	IVFTIISIV	0.20/0.60767042
	48	61	TITLTIFNC	0.55/0.2542018
	49	8	QFTSDQATF	0.78/0.01411364
	50	67	FNCFYALNN	0.92/-0.13202853
	51	193	GFAVFVKSK	0.00/0.78456363
	52	45	FVYLIKMII	0.37/0.40929747
	53	66	IFNCFYALN	0.82/-0.074265626
	54	39	YTSRSMFVY	0.36/0.38320157
	55	88	SIVIWILYF	0.09/0.65029146
	56	154	VKLGTGYTL	0.92/-0.18144495
	57	164	DLPVYVTVA	0.99/-0.25351136
	58	92	WILYFVNSI	0.07/0.65480057
	59	56	LMWPLTITL	0.07/0.65168812
	60	89	IVIWILYFV	0.00/0.71849902
	61	76	AFLAFSIVF	0.51/0.20705242
	62	27	ILLFITIIL	0.01/0.67852814
	63	9	FTSDQATFL	0.63/0.032709597
	64	52	IILWLMWPL	0.20/0.45035621
	**65**	**95**	**YFVNSIRLF**	**0.77/-0.13241026**
	66	40	TSRSMFVYL	0.08/0.55538402
	67	93	ILYFVNSIR	0.02/0.59878913
	68	97	VNSIRLFIR	0.00/0.60407438
	69	116	TNNLMCIDM	0.02/0.5838765
	70	78	LAFSIVFTI	0.02/0.57733795
	71	87	ISIVIWILY	0.21/0.38150171
	72	22	FSLGVILLF	0.08/0.44594864
	73	16	FLKEWNFSL	0.11/0.38922126
	74	101	RLFIRTGSW	0.11/0.38242376
	75	73	LNNAFLAFS	0.92/-0.42902267
	76	149	LYIQGVKLG	0.67/-0.22461178
	77	28	LLFITIILQ	0.80/-0.39815528
	78	42	RSMFVYLIK	0.00/0.39421498
	79	166	PVYVTVAKV	0.00/0.36871985
	80	109	WWSFNPETN	0.77/-0.40375519
	81	188	LDVNSGFAV	0.96/-0.61386228
	82	127	KMFVRPVIE	0.64/-0.33198466
	83	144	VIRGHLYIQ	0.81/-0.50687677
	84	103	FIRTGSWWS	0.75/-0.45677222

	85	15	TFLKEWNFS	0.75/-0.47347113
	86	30	FITIILQFG	0.73/-0.46932787
	87	130	VRPVIEDYH	0.64/-0.38282906
	88	106	TGSWWSFNP	0.87/-0.64308731
	89	140	LTATVIRGH	0.94/-0.76560859
	90	182	RAFLDKLDV	0.89/-0.75765784
	91	131	RPVIEDYHT	0.68/-0.67948815
	92	108	SWWSFNPET	0.71/-0.72766196
	93	35	LQFGYTSRS	0.91/-1.0393013
	94	181	KRAFLDKLD	0.79/-0.93814167
	95	201	KVGNYRLPS	0.69/-0.89105159
	96	133	VIEDYHTLT	0.84/-1.2414406
	97	137	YHTLTATVI	0.56/-1.0434784
	98	158	TGYTLSDLP	0.55/-1.1778233
	99	156	LGTGYTLSD	0.58/-1.8489281
**SARS-CoV-2**				
**Viral protein name**	**Rank**	**Start position**	**Sequence**	**Score ANN/SVM**
**Nucleocapsid**	1	262	RTATKAYNV	0.89/0.93090592
	**2**	**222**	**LLLDRLNQL**	**1.00/0.77917492**
	**3**	**85**	**GYYRRATRR**	**0.96/0.64584436**
	**4**	**105**	**SPRWYFYYL**	**0.09/1.2901952**
	5	165	TTLPKGFYA	0.84/0.47343878
	6	269	NVTQAFGRR	0.99/0.29166166
	**7**	**150**	**NPANNAAIV**	**0.84/0.43927091**
	8	266	KAYNVTQAF	0.83/0.43425876
	9	78	SSPDDQIGY	0.84/0.40764252
	10	215	GDAALALLL	0.90/0.34336961
	11	211	AGNGGDAAL	0.87/0.36326844
	12	87	YRRATRRIR	0.36/0.86386896
	13	216	DAALALLLL	0.19/1.026872
	14	153	NNAAIVLQL	0.70/0.43817133
	15	201	SSRGTSPAR	0.62/0.51098887
	16	131	IWVATEGAL	0.97/0.12227819
	17	226	RLNQLESKM	0.55/0.5287504
	18	138	ALNTPKDHI	0.49/0.54832454
	19	202	SRGTSPARM	0.19/0.84455909
	20	345	NFKDQVILL	0.04/0.93817097
	21	355	KHIDAYKTF	0.59/0.38618339

	22	37	SKQRRPQGL	0.82/0.15310833
	23	47	NNTASWFTA	0.99/-0.023318872
	24	312	SAFFGMSRI	0.00/0.93379976
	25	146	IGTRNPANN	0.95/-0.020760697
	26	258	PRQKRTATK	0.93/-0.0085201677
	27	81	DDQIGYYRR	0.60/0.31359257
	28	86	YYRRATRRI	0.03/0.87920722
	29	188	SRSRNSSRN	0.74/0.15874619
	30	45	LPNNTASWF	0.87/0.026589893
	31	323	EVTPSGTWL	0.50/0.38083972
	32	186	SSSRSRNSS	0.95/-0.077380571
	**33**	**125**	**ANKDGIIWV**	**0.02/0.85111885**
	34	166	TLPKGFYAE	0.89/-0.029340939
	35	240	QQQGQTVTK	0.98/-0.12221078
	36	124	GANKDGIIW	0.55/0.29607236
	37	9	QRNAPRITF	0.15/0.69591987
	38	30	GERSGARSK	0.12/0.72256846
	**39**	**311**	**ASAFFGMSR**	**0.02/0.78351091**
	40	299	KHWPQIAQF	0.69/0.1045106
	41	102	KDLSPRWYF	0.11/0.68097968
	42	89	RATRRIRGG	0.85/-0.095326758
	43	93	RIRGGDGKM	0.98/-0.24295114
	**44**	**181**	**QASSRSSSR**	**0.32/0.40488402**
	45	122	PYGANKDGI	0.96/-0.23589992
	46	141	TPKDHIGTR	0.79/-0.070754546
	47	96	GGDGKMKDL	0.05/0.65008429
	48	68	RGQGVPINT	0.83/-0.13269028
	49	84	IGYYRRATR	0.01/0.66944734
	50	338	KLDDKDPNF	0.01/0.66715268
	51	2	SDNGPQNQR	0.64/0.036463719
	52	53	FTALTQHGK	0.08/0.59448868
	53	329	TWLTYTGAI	0.99/-0.31632651
	54	213	NGGDAALAL	0.67/0.0028763949
	55	48	NTASWFTAL	0.71/-0.042997835
	56	173	AEGSRGGSQ	0.98/-0.34218131
	57	7	QNQRNAPRI	0.14/0.48978888
	58	306	QFAPSASAF	0.00/0.6216748
	59	218	ALALLLLDR	0.19/0.42058446

	60	210	MAGNGGDAA	0.82/-0.21169807
	61	119	AGLPYGANK	0.21/0.38539776
	62	148	TRNPANNAA	0.04/0.54723853
	63	362	TFPPTDFSK	/0.55405184
	64	175	GSRGGSQAS	0.96/-0.41600347
	65	117	PEAGLPYGA	0.62/-0.093465736
	66	88	RRATRRIRG	0.01/0.51061059
	67	314	FFGMSRIGM	0.04/0.44248594
	68	368	FSKQLQQSM	/0.48014359
	69	343	DPNFKDQVI	0.00/0.46907794
	70	103	DLSPRWYFY	0.00/0.45513017
	71	289	QELIRQGTD	0.96/-0.50787165
	72	309	PSASAFFGM	0.53/-0.087196442
	73	229	QLESKMSGK	0.79/-0.36665693
	74	4	NGPQNQRNA	0.02/0.397257
	75	270	VTQAFGRRG	0.82/-0.4093242
	76	66	FPRGQGVPI	0.05/0.36004115
	77	26	SNQNGERSG	0.91/-0.50282377
	78	156	AIVLQLPQG	0.54/-0.14856364
	79	50	ASWFTALTQ	0.71/-0.31887616
	80	298	YKHWPQIAQ	0.94/-0.55888964
	81	143	KDHIGTRNP	0.74/-0.35924171
	82	205	TSPARMAGN	0.80/-0.46091766
	83	349	QVILLNKHI	0.54/-0.23291994
	84	253	EASKKPRQK	0.57/-0.2697544
	85	246	VTKKSAAEA	0.88/-0.58975552
	86	115	TGPEAGLPY	1.00/-0.72582022
	87	256	KKPRQKRTA	0.55/-0.31949939
	88	339	LDDKDPNFK	1.00/-0.79829376
	89	217	AALALLLLD	0.82/-0.66387806
	90	12	APRITFGGP	0.64/-0.51055721
	91	320	IGMEVTPSG	0.93/-0.80199944
	92	268	YNVTQAFGR	0.66/-0.54408881
	93	233	KMSGKGQQQ	0.66/-0.5681714
	94	301	WPQIAQFAP	0.86/-0.7731242
	95	260	QKRTATKAY	0.82/-0.73406697
	96	305	AQFAPSASA	0.68/-0.63194405
	97	244	QTVTKKSAA	0.71/-0.6637588
	98	22	DSTGSNQNG	0.97/-0.92411209

	99	3	DNGPQNQRN	0.97/-0.92525482
	100	231	ESKMSGKGQ	0.85/-0.84680824
	101	107	RWYFYYLGT	0.57/-0.57892917
	102	60	GKEDLKFPR	0.83/-0.85866559
	103	332	TYTGAIKLD	0.96/-1.0332853
	104	139	LNTPKDHIG	0.68/-0.76483333
	105	197	STPGSSRGT	0.96/-1.0609933
	106	278	GPEQTQGNF	0.56/-0.74934502
	107	17	FGGPSDSTG	0.85/-1.0477316
	108	137	GALNTPKDH	0.94/-1.1694863
	109	157	IVLQLPQGT	0.65/-0.94266092
	110	97	GDGKMKDLS	0.79/-1.096834
	111	279	PEQTQGNFG	0.91/-1.2228358
	112	198	TPGSSRGTS	0.65/-0.9769109
	113	16	TFGGPSDST	0.88/-1.2239998
	114	239	QQQQGQTVT	0.59/-0.95994625
	115	340	DDKDPNFKD	0.52/-0.90141856
	116	158	VLQLPQGTT	0.91/-1.2940397
	117	333	YTGAIKLDD	0.67/-1.0851174
	118	247	TKKSAAEAS	0.78/-1.2140661
	**119**	**72**	**VPINTNSSP**	**0.55/-1.0398301**
	120	294	QGTDYKHWP	0.73/-1.2211872
	121	335	GAIKLDDKD	0.94/-1.4612102
	122	302	PQIAQFAPS	0.61/-1.2992434
	123	168	PKGFYAEGS	0.80/-1.7322271
**SARS-CoV**				
**Viral protein name**	**Rank**	**Start position**	**Sequence**	**Score ANN/SVM**
**Nucleocapsid**	1	87	YYRRATRRV	0.96/1.0113912
	2	212	ASGGGETAL	0.91/0.90898954
	**3**	**106**	**SPRWYFYYL**	**0.44/1.2901952**
	4	103	KELSPRWYF	0.56/1.1574925
	5	203	SRGNSPARM	0.96/0.68383146
	6	85	IGYYRRATR	0.94/0.66944734
	**7**	**151**	**NPNNNAATV**	**0.90/0.64775325**
	8	346	QFKDNVILL	0.20/1.2729819
	9	166	TTLPKGFYA	0.90/0.47343878
	10	315	FFGMSRIGM	0.91/0.44248594
	**11**	**312**	**ASAFFGMSR**	**0.55/0.78351091**

	12	255	ASKKPRQKR	0.98/0.35330623
	13	104	ELSPRWYFY	0.70/0.63184645
	**14**	**182**	**QASSRSSSR**	**0.79/0.40488402**
	15	313	SAFFGMSRI	0.23/0.93379976
	16	149	TRNPNNNAA	0.06/1.0992146
	17	381	QPLPQRQKK	0.81/0.331546
	18	348	KDNVILLNK	0.73/0.4042565
	19	217	ETALALLLL	0.00/1.1212241
	20	242	QQGQTVTKK	0.95/0.15472685
	21	344	DPQFKDNVI	0.93/0.15510863
	22	97	GGDGKMKEL	0.04/1.0139312
	23	216	GETALALLL	0.79/0.25443743
	24	332	LTYHGAIKL	0.63/0.40861273
	25	308	FAPSASAFF	0.68/0.3213651
	26	101	KMKELSPRW	1.00/-0.021813224
	27	7	QSNQRSAPR	0.35/0.62090318
	28	329	GTWLTYHGA	0.80/0.15791305
	29	62	KEELRFPRG	0.96/-0.045242636
	30	120	ASLPYGANK	0.08/0.82674523
	31	183	ASSRSSSRS	1.00/-0.10019775
	32	65	LRFPRGQGV	0.66/0.23783152
	33	353	LLNKHIDAY	0.85/0.021288078
	34	80	GPDDQIGYY	0.84/0.025720532
	35	227	RLNQLESKV	0.00/0.86225984
	**36**	**223**	**LLLDRLNQL**	**0.07/0.77917492**
	37	362	KTFPPTEPK	0.60/0.24733102
	38	128	KEGIVWVAT	0.59/0.24617659
	39	263	RTATKQYNV	0.00/0.82832214
	40	10	QRSAPRITF	0.09/0.73651318
	41	307	QFAPSASAF	0.19/0.6216748
	42	380	AQPLPQRQK	0.23/0.5753709
	43	350	NVILLNKHI	0.98/-0.19546084
	44	267	KQYNVTQAF	0.18/0.58266537
	45	142	TPKDHIGTR	0.83/-0.070754546
	46	202	SSRGNSPAR	0.09/0.66714286
	**47**	**126**	**ANKEGIVWV**	**0.20/0.55428711**
	48	160	LQLPQGTTL	0.98/-0.23496764
	49	205	GNSPARMAS	0.99/-0.24525216
	50	152	PNNNAATVL	0.97/-0.25317817

	51	26	DNNQNGGRN	0.97/-0.27184383
	**52**	**86**	**GYYRRATRR**	**0.05/0.64584436**
	53	400	DMDDFSRQL	/0.6943173
	54	33	RNGARPKQR	0.97/-0.28343228
	55	88	YRRATRRVR	0.00/0.68621587
	56	272	TQAFGRRGP	0.89/-0.20410199
	57	260	RQKRTATKQ	0.98/-0.3004523
	58	249	KKSAAEASK	0.93/-0.27044377
	59	139	ALNTPKDHI	0.11/0.54832454
	60	154	NNAATVLQL	0.06/0.5857138
	61	8	SNQRSAPRI	0.16/0.4740182
	62	360	AYKTFPPTE	0.52/0.11141851
	63	35	GARPKQRRP	0.83/-0.20998679
	64	29	QNGGRNGAR	0.96/-0.36319524
	65	54	FTALTQHGK	0.00/0.59448868
	66	9	NQRSAPRIT	0.75/-0.16130801
	67	292	LIRQGTDYK	0.51/0.072620614
	68	42	RPQGLPNNT	0.81/-0.24416525
	69	339	KLDDKDPQF	0.10/0.45884604
	70	136	TEGALNTPK	0.72/-0.16914112
	71	57	LTQHGKEEL	0.58/-0.042653612
	72	186	RSSSRSRGN	0.90/-0.37107981
	73	90	RATRRVRGG	0.10/0.42004926
	74	207	SPARMASGG	0.94/-0.42016665
	75	286	NFGDQDLIR	0.60/-0.083027619
	76	201	GSSRGNSPA	0.64/-0.1233026
	77	220	LALLLLDRL	0.62/-0.11574675
	78	56	ALTQHGKEE	0.94/-0.44868483
	79	84	QIGYYRRAT	0.91/-0.42881926
	80	363	TFPPTEPKK	0.03/0.4507155
	81	112	YYLGTGPEA	0.55/-0.077572032
	82	294	RQGTDYKHW	0.95/-0.48354644
	83	324	EVTPSGTWL	0.08/0.38083972
	84	296	GTDYKHWPQ	0.85/-0.38989884
	85	146	HIGTRNPNN	0.99/-0.54574633
	86	176	GSRGGSQAS	0.86/-0.41600347
	87	404	FSRQLQNSM	/0.43846516
	88	219	ALALLLLDR	0.01/0.42058446
	89	214	GGGETALAL	0.02/0.40188538

	90	11	RSAPRITFG	0.02/0.39113805
	91	2	SDNGPQSNQ	0.99/-0.58342809
	92	356	KHIDAYKTF	0.00/0.38618339
	93	3	DNGPQSNQR	0.89/-0.50840327
	94	67	FPRGQGVPI	0.02/0.36004115
	95	63	EELRFPRGQ	1.00/-0.63332203
	96	52	SWFTALTQH	0.99/-0.62969617
	97	293	IRQGTDYKH	0.87/-0.5127469
	98	175	EGSRGGSQA	0.55/-0.19442815
	99	210	RMASGGGET	0.88/-0.53626227
	100	316	FGMSRIGME	0.76/-0.43050044
	101	121	SLPYGANKE	0.59/-0.28571486
	102	92	TRRVRGGDG	0.57/-0.27342213
	103	129	EGIVWVATE	0.63/-0.34245656
	104	165	GTTLPKGFY	0.96/-0.68259645
	105	269	YNVTQAFGR	0.82/-0.54408881
	106	274	AFGRRGPEQ	0.96/-0.69079218
	107	235	VSGKGQQQQ	0.93/-0.6682894
	108	24	STDNNQNGG	0.66/-0.40356691
	109	190	RSRGNSRNS	0.52/-0.28449689
	110	291	DLIRQGTDY	0.66/-0.42705044
	111	199	TPGSSRGNS	0.87/-0.69928417
	112	351	VILLNKHID	0.90/-0.76166859
	113	20	GPTDSTDNN	0.75/-0.62044357
	114	91	ATRRVRGGD	0.55/-0.4368316
	115	247	VTKKSAAEA	0.70/-0.58975552
	116	40	QRRPQGLPN	0.96/-0.85617741
	117	390	QPTVTLLPA	0.58/-0.50072892
	118	114	LGTGPEASL	0.87/-0.80322609
	119	43	PQGLPNNTA	0.97/-0.93230078
	120	22	TDSTDNNQN	0.84/-0.81465668
	121	252	AAEASKKPR	0.76/-0.74082171
	122	229	NQLESKVSG	0.91/-0.90777161
	123	158	TVLQLPQGT	0.91/-0.9130794
	124	302	WPQIAQFAP	0.77/-0.7731242
	125	240	QQQQGQTVT	0.93/-0.95994625
	126	78	NSGPDDQIG	0.52/-0.57410796
	127	47	PNNTASWFT	0.83/-0.8916636
	128	66	RFPRGQGVP	0.85/-0.92044246

	129	232	ESKVSGKGQ	0.68/-0.80324094
	130	233	SKVSGKGQQ	0.98/-1.1173681
	131	283	TQGNFGDQD	0.64/-0.8435021
	132	342	DKDPQFKDN	0.69/-0.90895165
	133	277	RRGPEQTQG	0.99/-1.216101
	134	295	QGTDYKHWP	0.96/-1.2211872
	135	382	PLPQRQKKQ	0.94/-1.2017533
	136	200	PGSSRGNSP	0.93/-1.1938617
	137	282	QTQGNFGDQ	0.99/-1.2588899
	138	320	RIGMEVTPS	0.99/-1.2663348
	139	377	TDEAQPLPQ	0.97/-1.3052823
	140	141	NTPKDHIGT	0.56/-0.95695803
	141	244	GQTVTKKSA	0.66/-1.143769
	142	238	KGQQQQGQT	0.95/-1.4372311
	143	322	GMEVTPSGT	0.71/-1.2057264
	**144**	**73**	**VPINTNSGP**	**0.67/-1.1760564**
	145	180	GSQASSRSS	0.75/-1.2942226
	146	6	PQSNQRSAP	0.87/-1.4717376
	147	198	STPGSSRGN	0.77/-1.3867119
	148	60	HGKEELRFP	0.59/-1.2712352
	149	74	PINTNSGPD	0.69/-1.3928335
	150	143	PKDHIGTRN	0.98/-1.6828756
	151	395	LLPAADMDD	0.60/-1.3249575
	152	173	YAEGSRGGS	0.98/-1.804953
	153	163	PQGTTLPKG	0.75/-1.7411809
**MERS-CoV**				
**Viral protein name**	**Rank**	**Start position**	**Sequence**	**Score ANN/SVM**
**Nucleocapsid**	1	326	VYFLRYSGA	0.82/0.85173683
	2	288	TEDPRWPQI	0.60/1.020664
	3	329	LRYSGAIKL	0.36/1.2090184
	**4**	**214**	**LYLDLLNRL**	**0.61/0.93922265**
	5	342	PNYNKWLEL	1.00/0.47691486
	**6**	**170**	**QSSSRASSV**	**0.92/0.48670336**
	7	92	KQLAPRWYF	0.55/0.78160836
	8	15	NNDITNTNL	0.87/0.4309186
	**9**	**115**	**AVKDGIVWV**	**0.01/1.2876187**
	10	347	WLELLEQNI	0.97/0.30634976
	11	35	AAPNNTVSW	0.96/0.26512505

	12	343	NYNKWLELL	0.57/0.63619978
	13	28	GRNPKPRAA	0.00/1.1641891
	14	211	GDLLYLDLL	0.90/0.25642104
	15	353	QNIDAYKTF	0.98/0.16272627
	16	155	KLPKNFHIE	0.82/0.30335762
	17	233	SQPKVITKK	0.79/0.32852037
	18	245	AAKNKMRHK	0.51/0.60005073
	19	109	AALPFRAVK	0.29/0.79870845
	20	82	DRKINTGNG	0.78/0.29116261
	21	327	YFLRYSGAI	0.70/0.37093526
	22	72	AQNAGYWRR	0.39/0.67261704
	23	130	DAPSTFGTR	0.51/0.53477132
	24	173	SRASSVSRN	0.99/0.023642934
	25	210	GGDLLYLDL	0.94/0.065766746
	26	90	GIKQLAPRW	0.96/0.017562599
	27	208	AVGGDLLYL	0.05/0.92649247
	**28**	**95**	**APRWYFYYT**	**0.60/0.37406648**
	29	246	AKNKMRHKR	0.99/-0.027664866
	30	143	DSAIVTQFA	0.49/0.46452033
	31	198	GTSPGPSGI	0.98/-0.034719191
	32	380	SEPPKEQRV	0.18/0.7439029
	33	356	DAYKTFPKK	0.84/0.082428678
	34	305	AFMGMSQFK	0.69/0.22322831
	35	250	MRHKRTSTK	0.00/0.90434598
	36	149	QFAPGTKLP	0.98/-0.084491668
	37	150	FAPGTKLPK	0.97/-0.084591696
	**38**	**139**	**NPNNDSAIV**	**0.69/0.19349234**
	39	18	ITNTNLSRG	0.96/-0.10557402
	40	221	RLQALESGK	0.97/-0.15275972
	41	322	HGNPVYFLR	0.33/0.47551321
	42	350	LLEQNIDAY	0.98/-0.17770976
	43	217	DLLNRLQAL	0.00/0.7886719
	44	154	TKLPKNFHI	0.33/0.43450264
	**45**	**303**	**ASAFMGMSQ**	**0.95/-0.1961539**
	46	183	SRSSSQGSR	0.74/-0.017063041
	47	328	FLRYSGAIK	0.00/0.71581396
	48	33	PRAAPNNTV	0.91/-0.19875788
	49	200	SPGPSGIGA	0.30/0.39825848
	50	176	SSVSRNSSR	0.31/0.38038282

	51	148	TQFAPGTKL	0.00/0.68511766
	52	330	RYSGAIKLD	1.00/-0.32297433
	53	291	PRWPQIAEL	0.16/0.51109243
	54	36	APNNTVSWY	0.00/0.66729812
	55	318	NNDDHGNPV	0.53/0.13346147
	56	80	RQDRKINTG	0.59/0.053859252
	57	57	FPPGQGVPL	0.14/0.45757756
	58	2	ASPAAPRAV	0.02/0.57689358
	59	39	NTVSWYTGL	0.83/-0.2415175
	60	24	SRGRGRNPK	0.06/0.5133712
	61	1	MASPAAPRA	0.52/0.042181518
	62	249	KMRHKRTST	0.97/-0.40798952
	63	259	SFNMVQAFG	0.70/-0.1449044
	64	384	KEQRVQGSI	0.00/0.55401551
	65	306	FMGMSQFKL	0.87/-0.3252675
	66	295	QIAELAPTA	0.59/-0.053972092
	67	394	QRTRTRPSV	/0.52141346
	68	60	GQGVPLNAN	0.74/-0.23063099
	**69**	**76**	**GYWRRQDRK**	**0.00/0.50733206**
	70	52	KVPLTFPPG	0.88/-0.37641247
	71	107	PEAALPFRA	0.93/-0.43063521
	72	77	YWRRQDRKI	0.72/-0.22493933
	73	43	WYTGLTQHG	0.62/-0.12625376
	74	340	KNPNYNKWL	0.67/-0.17807239
	75	338	DPKNPNYNK	0.00/0.48988479
	76	137	TRNPNNDSA	0.09/0.38771361
	77	213	LLYLDLLNR	0.02/0.43876131
	78	349	ELLEQNIDA	0.92/-0.4646237
	79	320	DDHGNPVYF	0.08/0.37157273
	80	304	SAFMGMSQF	0.03/0.40607493
	81	27	RGRNPKPRA	0.84/-0.41637797
	82	84	KINTGNGIK	0.00/0.41259831
	83	402	VQPGPMIDV	/0.41215303
	84	242	DAAAAKNKM	0.73/-0.32337359
	85	75	AGYWRRQDR	0.01/0.39004073
	86	106	GPEAALPFR	0.93/-0.53419678
	87	113	FRAVKDGIV	0.00/0.39224958
	88	292	RWPQIAELA	0.97/-0.60231607
	89	232	QSQPKVITK	0.00/0.36478831

	90	23	LSRGRGRNP	0.75/-0.40775681
	91	360	TFPKKEKKQ	0.92/-0.59649026
	92	83	RKINTGNGI	0.95/-0.62975784
	93	372	KEESTDQMS	0.82/-0.50634084
	94	201	PGPSGIGAV	0.59/-0.28998395
	95	252	HKRTSTKSF	0.97/-0.67059799
	96	117	KDGIVWVHE	0.51/-0.2218636
	97	332	SGAIKLDPK	0.95/-0.69689508
	98	185	SSSQGSRSG	0.56/-0.31672703
	99	247	KNKMRHKRT	0.52/-0.28027281
	100	37	PNNTVSWYT	0.88/-0.65141131
	101	42	SWYTGLTQH	0.82/-0.62385673
	102	122	WVHEDGATD	0.72/-0.52644832
	103	319	NDDHGNPVY	0.61/-0.42396956
	104	263	VQAFGLRGP	0.74/-0.61483983
	105	123	VHEDGATDA	0.80/-0.67587854
	106	227	SGKVKQSQP	0.99/-0.91028653
	107	194	NSTRGTSPG	0.96/-0.88518292
	108	98	WYFYYTGTG	0.92/-0.88490721
	109	224	ALESGKVKQ	1.00/-0.97315219
	110	223	QALESGKVK	0.79/-0.78521188
	111	310	SQFKLTHQN	0.81/-0.82783131
	112	317	QNNDDHGNP	0.77/-0.79513552
	113	192	SGNSTRGTS	0.96/-1.0468348
	114	203	PSGIGAVGG	0.74/-0.82836864
	115	212	DLLYLDLLN	0.55/-0.65856866
	116	13	ADNNDITNT	0.54/-0.6704073
	117	280	DLQLNKLGT	0.77/-0.94074599
	118	231	KQSQPKVIT	0.51/-0.68078812
	119	40	TVSWYTGLT	0.84/-1.0114911
	120	168	NSQSSSRAS	0.67/-0.88895741
	121	269	RGPGDLQGN	0.92/-1.1405124
	122	335	IKLDPKNPN	0.85/-1.0728028
	123	365	EKKQKAPKE	0.79/-1.014063
	124	309	MSQFKLTHQ	0.62/-0.85540747
	125	16	NDITNTNLS	0.97/-1.2251622
	126	222	LQALESGKV	0.78/-1.082446
	127	46	GLTQHGKVP	0.95/-1.2622989
	128	331	YSGAIKLDP	0.94/-1.2708713

	129	119	GIVWVHEDG	0.60/-0.98102059
	130	363	KKEKKQKAP	0.93/-1.3267215
	131	7	PRAVSFADN	0.65/-1.0473359
	132	271	PGDLQGNFG	0.62/-1.0298644
	133	333	GAIKLDPKN	0.85/-1.2743699
	134	237	VITKKDAAA	0.89/-1.3561122
	135	196	TRGTSPGPS	0.60/-1.0713156
	136	65	LNANSTPAQ	0.66/-1.1731177
	137	375	STDQMSEPP	0.86/-1.5075913
	138	204	SGIGAVGGD	0.93/-1.5873422
	139	121	VWVHEDGAT	0.60/-1.2582851
	140	366	KKQKAPKEE	0.53/-1.2082515
	**141**	**63**	**VPLNANSTP**	**0.70/-1.4579462**
**HCoV-NL63**				
**Viral protein name**	**Rank**	**Start position**	**Sequence**	**Score ANN/SVM**
**Nucleocapsid**	1	93	QRSDGVVWV	0.83/1.0503585
	2	27	VSSDKAPYR	0.89/0.85441578
	3	153	RSSTRNNSR	0.98/0.53378298
	4	297	EVGDNVQIT	0.81/0.65935549
	5	64	MRRGQRVDL	0.64/0.82563036
	6	38	PRNLVPIGK	0.99/0.3316211
	7	251	GPRDFNHNM	0.07/1.2149976
	8	7	ADDRAARKK	0.95/0.33291945
	9	167	TSRQQSRTR	0.59/0.6626187
	10	33	PYRVIPRNL	0.89/0.34361091
	11	32	APYRVIPRN	0.90/0.31070761
	12	181	SSSDLVAAV	0.22/0.98207037
	13	127	FSIALPPEL	0.96/0.22155007
	14	166	STSRQQSRT	0.91/0.26624856
	15	272	KGFPQLAEL	0.63/0.48881282
	16	61	RWRMRRGQR	0.88/0.237016
	17	53	IGYWNVQER	0.31/0.788897
	18	279	ELIPNQAAL	0.00/1.0575762
	19	296	DEVGDNVQI	0.88/0.17056271
	20	278	AELIPNQAA	0.79/0.25853901
	21	94	RSDGVVWVA	0.16/0.88619516
	22	6	WADDRAARK	0.00/1.0073102
	23	238	RVPTREENV	0.00/0.99866868

	24	327	SAFTKPSSI	0.70/0.26711755
	25	247	IQCFGPRDF	0.43/0.52897559
	26	321	KFIEQISAF	0.25/0.70443494
	27	47	GNKDEQIGY	0.57/0.37815546
	28	230	QLKKPRWKR	0.21/0.73360993
	29	62	WRMRRGQRV	0.00/0.91669726
	30	3	SVNWADDRA	0.63/0.28523363
	31	50	DEQIGYWNV	0.00/0.89996169
	32	29	SDKAPYRVI	0.67/0.22325403
	33	281	IPNQAALFF	0.02/0.87146481
	34	188	AVTLALKNL	0.04/0.84887309
	35	195	NLGFDNQSK	0.90/-0.022352029
	36	95	SDGVVWVAK	0.52/0.33841478
	37	318	NLPKFIEQI	0.04/0.79643602
	38	243	EENVIQCFG	0.96/-0.12436364
	39	70	VDLPPKVHF	0.89/-0.066727275
	40	367	AIIEIVNEV	/0.82051619
	41	245	NVIQCFGPR	0.59/0.2096198
	42	68	QRVDLPPKV	0.72/0.078024545
	43	264	LVQNGVDAK	0.76/0.028387027
	44	294	STDEVGDNV	0.00/0.78810974
	45	58	VQERWRMRR	0.60/0.17273593
	46	28	SSDKAPYRV	0.08/0.6794857
	47	57	NVQERWRMR	0.02/0.7372599
	48	224	RADKPSQLK	0.20/0.55370903
	49	330	TKPSSIKEM	0.86/-0.10663569
	50	146	NSSRASSRS	0.74/0.0073732169
	51	36	VIPRNLVPI	0.94/-0.19830235
	52	129	IALPPELSV	0.64/0.094165838
	53	145	NNSSRASSR	0.00/0.72792832
	54	149	RASSRSSTR	0.01/0.71577376
	55	314	KDNKNLPKF	0.75/-0.029560779
	56	183	SDLVAAVTL	0.95/-0.23214497
	57	269	VDAKGFPQL	0.67/0.047815037
	58	123	LEPKFSIAL	0.22/0.49253334
	59	302	VQITYTYKM	0.98/-0.27260668
	60	121	KPLEPKFSI	0.29/0.40952758
	61	165	RSTSRQQSR	0.01/0.65243185
	62	56	WNVQERWRM	0.03/0.60837944

	63	265	VQNGVDAKG	1.00/-0.3761421
	64	105	GAKTVNTSL	0.03/0.58121056
	65	180	QSSSDLVAA	0.77/-0.17987904
	66	157	RNNSRDSSR	0.07/0.51667575
	67	177	DSNQSSSDL	0.14/0.43242551
	68	159	NSRDSSRST	0.56/-0.0050559884
	69	108	TVNTSLGNR	0.01/0.54170818
	70	133	PELSVVEFE	0.93/-0.39332569
	71	334	SIKEMQSQS	0.98/-0.44833146
	72	48	NKDEQIGYW	0.58/-0.059508967
	73	79	YYLGTGPHK	0.02/0.49258046
	74	15	KFPPPSFYM	0.06/0.44951581
	75	342	SSHVVQNTV	0.04/0.45734456
	76	114	GNRKRNQKP	0.96/-0.46925538
	77	112	SLGNRKRNQ	0.93/-0.4486876
	78	301	NVQITYTYK	0.00/0.47327988
	79	241	TREENVIQC	0.00/0.46984244
	80	63	RMRRGQRVD	0.94/-0.47648794
	81	368	IIEIVNEVL	/0.45993555
	82	347	QNTVLNASI	0.98/-0.52255007
	83	252	PRDFNHNMG	0.72/-0.27256097
	84	323	IEQISAFTK	0.07/0.37570906
	85	115	NRKRNQKPL	0.00/0.40656426
	86	242	REENVIQCF	0.01/0.3949619
	**87**	**73**	**PPKVHFYYL**	**0.03/0.36963986**
	88	59	QERWRMRRG	0.01/0.38920753
	89	162	DSSRSTSRQ	0.52/-0.15910388
	90	324	EQISAFTKP	0.94/-0.58828055
	91	336	KEMQSQSSH	0.59/-0.2934749
	92	109	VNTSLGNRK	0.75/-0.45733365
	93	80	YLGTGPHKD	0.98/-0.70409094
	94	174	TRSDSNQSS	0.69/-0.42839095
	95	348	NTVLNASIP	0.67/-0.41687283
	96	307	TYKMLVAKD	0.90/-0.65446786
	97	26	LVSSDKAPY	0.89/-0.65067805
	98	256	NHNMGDSDL	0.52/-0.29159951
	99	136	SVVEFEDRS	0.59/-0.36166442
	100	309	KMLVAKDNK	0.92/-0.69753357

	101	92	RQRSDGVVW	0.55/-0.33061775
	102	134	ELSVVEFED	0.70/-0.4834477
	103	350	VLNASIPES	0.62/-0.41936453
	104	190	TLALKNLGF	0.76/-0.57306937
	105	122	PLEPKFSIA	0.83/-0.64468962
	106	103	KEGAKTVNT	0.72/-0.54231507
	107	72	LPPKVHFYY	0.69/-0.52054071
	108	144	SNNSSRASS	0.61/-0.46571218
	109	333	SSIKEMQSQ	0.94/-0.82165389
	110	99	VWVAKEGAK	0.75/-0.64096712
	111	311	LVAKDNKNL	0.65/-0.56632874
	112	312	VAKDNKNLP	0.59/-0.50910295
	113	106	AKTVNTSLG	0.65/-0.58682727
	114	193	LKNLGFDNQ	0.62/-0.5680716
	115	258	NMGDSDLVQ	0.84/-0.78932
	116	204	SPSSSGTST	0.57/-0.55455441
	117	178	SNQSSSDLV	0.72/-0.72102631
	118	89	LKFRQRSDG	0.94/-0.97484454
	119	274	FPQLAELIP	0.89/-0.93373417
	120	280	LIPNQAALF	0.55/-0.60569392
	121	332	PSSIKEMQS	0.92/-1.0260017
	122	40	NLVPIGKGN	0.99/-1.1594635
	123	19	PSFYMPLLV	0.53/-0.7327578
	124	352	NASIPESKP	0.53/-0.77936507
	125	196	LGFDNQSKS	0.93/-1.1827807
	126	49	KDEQIGYWN	0.93/-1.1991049
	127	207	SSGTSTPKK	0.56/-0.83177295
	128	155	STRNNSRDS	0.64/-1.0013112
	129	213	PKKPNKPLS	0.94/-1.3310662
	130	10	RAARKKFPP	0.81/-1.2379601
	131	197	GFDNQSKSP	0.62/-1.0696074
	132	313	AKDNKNLPK	0.67/-1.1337899
	133	52	QIGYWNVQE	0.81/-1.2762632
	134	217	NKPLSQPRA	0.62/-1.1000601
	135	77	HFYYLGTGP	0.92/-1.4174491
	136	232	KKPRWKRVP	0.74/-1.3135443
	137	222	QPRADKPSQ	0.62/-1.2730379
	138	176	SDSNQSSSD	0.87/-1.5739187
	139	308	YKMLVAKDN	0.81/-1.5219762

	140	275	PQLAELIPN	0.75/-1.5298008
	141	102	AKEGAKTVN	0.73/-1.5281956
	142	208	SGTSTPKKP	0.61/-1.4323565
	143	335	IKEMQSQSS	0.72/-1.6078258
	144	24	PLLVSSDKA	0.63/-1.8420451
	145	262	SDLVQNGVD	0.67/-1.9171155
**HCoV-229E**				
**Viral protein name**	**Rank**	**Start position**	**Sequence**	**Score ANN/SVM**
**Nucleocapsid**	1	96	RVEGVVWVA	0.99/0.91622308
	2	333	HLGKFLEEL	0.77/1.0331021
	3	16	RQGRIPYSL	0.95/0.69755833
	4	131	QKLPNGVTV	0.93/0.65267196
	5	340	ELNAFTREM	0.90/0.61756135
	6	146	RAPSRSQSR	0.60/0.90146006
	7	142	EPDSRAPSR	0.65/0.82474115
	8	40	PRNLVPINK	0.88/0.54735297
	9	60	VQKRFRTRK	0.91/0.49418632
	**10**	**75**	**SPKLHFYYL**	**0.15/1.2036463**
	11	125	EIPHFNQKL	1.00/0.34615981
	12	55	IGYWNVQKR	0.95/0.3801915
	13	49	KDKNKLIGY	0.59/0.64992196
	14	97	VEGVVWVAV	0.14/1.0911548
	15	301	AMLFDSHIV	0.90/0.32027775
	16	178	SQDDIMKAV	0.18/1.0281547
	17	316	TVVLTFTTR	0.55/0.63669774
	18	262	TQCFGPRDL	0.04/1.1314081
	19	182	IMKAVAAAL	0.70/0.46065499
	20	295	LVPSTAAML	0.91/0.24603278
	21	66	TRKGKRVDL	0.00/1.0673474
	22	70	KRVDLSPKL	0.98/0.086905042
	23	12	PQRGRQGRI	0.93/0.11614714
	24	288	GYPQFAELV	0.38/0.66351077
	25	84	GTGPHKDAK	0.91/0.042910867
	26	311	KESGNTVVL	0.10/0.83520062
	27	73	DLSPKLHFY	0.36/0.57360838
	28	57	YWNVQKRFR	0.97/-0.040738699
	29	242	HEMQKPRWK	0.92/0.0075692109
	30	30	VDSEQPWKV	0.87/0.047847944

	31	276	SAGVVANGV	0.88/0.033896012
	32	62	KRFRTRKGK	0.01/0.87190794
	33	266	GPRDLDHNF	0.85/0.018575467
	34	85	TGPHKDAKF	0.95/-0.094427052
	35	88	HKDAKFRER	0.66/0.17563285
	36	269	DLDHNFGSA	0.95/-0.12192039
	37	287	KGYPQFAEL	0.01/0.81610108
	38	351	QPLLNPSAL	0.44/0.37436007
	39	95	ERVEGVVWV	0.02/0.77795782
	40	153	SRSQSRSRG	0.87/-0.072772925
	41	160	RGESKSQSR	0.94/-0.14344557
	42	258	TSNVTQCFG	0.95/-0.17865933
	43	336	KFLEELNAF	0.17/0.58639788
	44	8	DASEPQRGR	0.82/-0.065278576
	45	147	APSRSQSRS	0.59/0.15949376
	46	212	KPSRNQSPA	0.01/0.72358528
	47	313	SGNTVVLTF	0.94/-0.21218693
	48	312	ESGNTVVLT	0.68/-0.00053717926
	49	132	KLPNGVTVV	0.04/0.63929904
	50	189	ALKSLGFDK	0.22/0.45660609
	51	207	KTGTPKPSR	0.64/0.0092939722
	52	94	RERVEGVVW	0.84/-0.19877636
	53	156	QSRSRGESK	0.72/-0.094187755
	54	81	YYLGTGPHK	0.13/0.49258046
	55	92	KFRERVEGV	0.23/0.3870249
	56	294	ELVPSTAAM	0.07/0.54195853
	57	219	PASSQSAAK	1.00/-0.39375896
	58	331	HPHLGKFLE	0.93/-0.32704756
	59	166	QSRNPSSDR	0.82/-0.22002781
	60	246	KPRWKRQPN	0.67/-0.073549592
	61	204	KSAKTGTPK	0.85/-0.25520001
	62	41	RNLVPINKK	0.11/0.47153736
	63	174	RNHNSQDDI	0.71/-0.14818412
	64	59	NVQKRFRTR	0.10/0.46031065
	65	61	QKRFRTRKG	0.95/-0.41058221
	66	366	TSPATVEPV	/0.53685795
	67	253	PNDDVTSNV	0.01/0.52487438
	68	52	NKLIGYWNV	0.04/0.4910114

	69	317	VVLTFTTRV	0.00/0.52411543
	70	303	LFDSHIVSK	0.91/-0.38718112
	71	236	ETKEQKHEM	0.00/0.52163142
	72	335	GKFLEELNA	0.88/-0.3764547
	73	155	SQSRSRGES	0.98/-0.48969212
	74	210	TPKPSRNQS	0.90/-0.41812113
	75	118	RRKNSEPEI	0.06/0.41790866
	76	370	TVEPVRDEV	/0.46443112
	77	279	VVANGVKAK	0.02/0.44383583
	78	305	DSHIVSKES	0.97/-0.51205745
	79	309	VSKESGNTV	0.01/0.44037585
	80	223	QSAAKILAR	0.00/0.43185349
	81	128	HFNQKLPNG	0.60/-0.17257577
	82	230	ARSQSSETK	0.02/0.40465747
	83	176	HNSQDDIMK	0.57/-0.15094078
	84	7	ADASEPQRG	0.92/-0.50517523
	85	46	INKKDKNKL	0.01/0.39938551
	86	151	SQSRSQSRS	0.65/-0.25747986
	87	233	QSSETKEQK	0.79/-0.39868484
	88	374	VRDEVSIET	/0.37206472
	89	193	LGFDKPQEK	0.67/-0.29807002
	90	76	PKLHFYYLG	0.97/-0.5998392
	91	36	WKVIPRNLV	0.64/-0.271922
	92	264	CFGPRDLDH	0.96/-0.59305311
	93	222	SQSAAKILA	0.98/-0.61830664
	94	169	NPSSDRNHN	0.62/-0.27202627
	95	343	AFTREMQQQ	0.99/-0.64317551
	96	310	SKESGNTVV	0.92/-0.58600038
	97	183	MKAVAAALK	0.77/-0.43764113
	98	162	ESKSQSRNP	0.71/-0.4052145
	99	144	DSRAPSRSQ	0.97/-0.67182608
	100	58	WNVQKRFRT	0.83/-0.53626171
	101	120	KNSEPEIPH	0.75/-0.46313565
	102	32	SEQPWKVIP	0.63/-0.37191504
	103	77	KLHFYYLGT	0.51/-0.26014265
	104	214	SRNQSPASS	0.61/-0.37235207
	105	255	DDVTSNVTQ	0.94/-0.70386792
	106	249	WKRQPNDDV	0.68/-0.53077535
	107	80	FYYLGTGPH	0.54/-0.39331188

	108	197	KPQEKDKKS	0.83/-0.71545221
	109	299	TAAMLFDSH	0.54/-0.42606284
	110	126	IPHFNQKLP	0.78/-0.68352791
	111	123	EPEIPHFNQ	0.75/-0.65410199
	112	209	GTPKPSRNQ	0.90/-0.82823563
	113	19	RIPYSLYSP	0.87/-0.81368293
	114	195	FDKPQEKDK	0.58/-0.53498434
	115	99	GVVWVAVDG	0.64/-0.60336552
	116	320	TFTTRVTVP	0.68/-0.65033092
	117	42	NLVPINKKD	0.86/-0.8308423
	118	239	EQKHEMQKP	0.86/-0.84002514
	119	328	PKDHPHLGK	0.97/-0.9627777
	120	263	QCFGPRDLD	0.93/-0.93474488
	121	339	EELNAFTRE	0.56/-0.56800581
	122	360	EFNPSQTSP	0.68/-0.69223836
	123	307	HIVSKESGN	0.56/-0.58140183
	124	281	ANGVKAKGY	0.52/-0.55856737
	125	215	RNQSPASSQ	0.51/-0.5617737
	126	304	FDSHIVSKE	0.75/-0.88250981
	127	179	QDDIMKAVA	0.51/-0.65401886
	128	2	ATVKWADAS	0.94/-1.1097636
	129	184	KAVAAALKS	0.76/-0.95242894
	130	136	GVTVVEEPD	0.55/-0.80294364
	131	251	RQPNDDVTS	0.75/-1.0132369
	132	332	PHLGKFLEE	0.56/-0.82481605
	133	188	AALKSLGFD	0.93/-1.214132
	134	363	PSQTSPATV	0.51/-0.8004713
	135	148	PSRSQSRSQ	0.97/-1.2882981
	136	78	LHFYYLGTG	0.99/-1.3365084
	137	213	PSRNQSPAS	0.99/-1.352974
	138	5	KWADASEPQ	0.72/-1.1760276
	139	190	LKSLGFDKP	0.93/-1.4108754
	140	356	PSALEFNPS	0.62/-1.1466631
	141	163	SKSQSRNPS	0.67/-1.2017927
	142	237	TKEQKHEMQ	0.91/-1.5050854
	143	354	LNPSALEFN	0.97/-1.6062851
	144	334	LGKFLEELN	0.75/-1.5378383
	145	4	VKWADASEP	0.60/-1.4799163
	146	198	PQEKDKKSA	0.54/-1.7502832

	147	290	PQFAELVPS	0.56/-1.8487869
	148	27	PLLVDSEQP	0.77/-2.2873173
**HCoV-OC43**				
**Viral protein name**	**Rank**	**Start position**	**Sequence**	**Score ANN/SVM**
**Nucleocapsid**	1	372	VLSENLNAY	0.98/1.1589641
	2	111	TADGNQRQL	0.86/1.0763098
	3	195	APNSRSTSR	0.99/0.72578835
	4	305	TSDPQFPIL	0.53/1.0784335
	5	355	GAIRFDSTL	1.00/0.59758371
	6	101	WYRHNRRSF	0.60/0.88504665
	7	320	AGAFFFGSR	0.98/0.49611093
	8	95	TEAKGYWYR	0.69/0.7854361
	9	348	VYELRYNGA	0.96/0.51262726
	10	214	RSRANSGNR	0.91/0.5574033
	11	174	TRFPPGTVL	0.33/1.1317061
	12	70	TQFQKGKEF	0.95/0.49619917
	13	127	YLGTGPHAK	0.92/0.50505149
	14	410	SVAVPKSRV	0.82/0.55273831
	15	102	YRHNRRSFK	0.56/0.78670916
	**16**	**120**	**LPRWYFYYL**	**0.00/1.3445772**
	17	33	FRNVQTRGR	0.65/0.69112238
	18	140	TDIDGVYWV	0.60/0.66153271
	19	350	ELRYNGAIR	0.66/0.57692708
	20	389	MSPKPQRQR	0.82/0.40238414
	21	212	GSRSRANSG	0.99/0.19801971
	22	180	TVLPQGYYI	0.11/1.071776
	23	292	NQNFGGGEM	0.86/0.24436914
	24	28	DQSDQFRNV	0.16/0.9288344
	25	362	TLSGFETIM	0.11/0.95739281
	26	248	ATKPQQVTK	0.02/1.0266931
	27	261	EVRQKILNK	0.85/0.18807761
	28	26	WADQSDQFR	0.94/0.067667846
	29	317	APTAGAFFF	0.00/0.99957346
	30	378	NAYQQQDGM	0.93/0.063430407
	31	38	TRGRRAQPK	0.63/0.36004039
	32	235	QIASLVLAK	0.37/0.60747635
	33	104	HNRRSFKTA	0.58/0.38779782
	34	374	SENLNAYQQ	0.54/0.415616

	35	118	QLLPRWYFY	0.98/-0.029293394
	36	35	NVQTRGRRA	0.95/-0.020171979
	37	407	DNISVAVPK	0.99/-0.071861209
	38	232	MADQIASLV	0.90/-0.0013435781
	39	57	GNVVPYYSW	0.83/0.065642286
	40	87	PIAPGVPAT	0.70/0.19052465
	41	405	ENDNISVAV	0.95/-0.066271
	42	236	IASLVLAKL	0.03/0.83832741
	43	72	FQKGKEFEF	0.15/0.71231577
	44	15	SGNRSGNGI	0.98/-0.12225342
	45	98	KGYWYRHNR	0.37/0.46659975
	46	281	TVQQCFGKR	0.76/0.075971959
	47	52	QQPSGGNVV	0.43/0.40136015
	48	117	RQLLPRWYF	0.12/0.69126931
	49	119	LLPRWYFYY	0.66/0.14958306
	50	144	GVYWVASNQ	0.74/0.064004786
	51	61	PYYSWFSGI	0.33/0.42787822
	52	203	RTSSRASSA	0.01/0.73618624
	53	298	GEMLKLGTS	0.91/-0.16625057
	54	112	ADGNQRQLL	0.65/0.092373213
	55	358	RFDSTLSGF	0.64/0.10180045
	56	170	EAIPTRFPP	0.96/-0.21946597
	57	76	KEFEFAEGQ	0.85/-0.11156586
	58	404	GENDNISVA	0.15/0.58622941
	59	18	RSGNGILKW	0.36/0.37499581
	60	200	STSRTSSRA	0.57/0.16279576
	61	81	AEGQGVPIA	0.00/0.73152769
	62	415	KSRVQQNKS	0.88/-0.16747608
	63	414	PKSRVQQNK	0.88/-0.17052663
	64	329	LELAKVQNL	0.15/0.54371414
	65	110	KTADGNQRQ	0.88/-0.18966282
	66	51	SQQPSGGNV	0.02/0.67014926
	67	323	FFFGSRLEL	0.30/0.38427807
	68	139	GTDIDGVYW	0.52/0.16174201
	69	280	CTVQQCFGK	0.89/-0.21336165
	70	258	TAKEVRQKI	0.03/0.64565508
	71	351	LRYNGAIRF	0.01/0.66297897
	72	204	TSSRASSAG	0.97/-0.29827064

	73	306	SDPQFPILA	0.80/-0.12835707
	74	326	GSRLELAKV	0.56/0.10387762
	75	17	NRSGNGILK	0.00/0.66350694
	76	154	DVNTPADIV	0.00/0.66288667
	77	322	AFFFGSRLE	0.80/-0.14400224
	78	308	PQFPILAEL	0.66/-0.015817526
	79	99	GYWYRHNRR	0.16/0.47660875
	80	418	VQQNKSIEL	0.19/0.43088901
	81	369	IMKVLSENL	0.11/0.5046064
	82	265	KILNKPRQK	0.67/-0.05586895
	83	115	NQRQLLPRW	0.86/-0.24916488
	84	65	WFSGITQFQ	0.98/-0.38070944
	85	66	FSGITQFQK	0.75/-0.15600666
	86	94	ATEAKGYWY	0.04/0.54584638
	87	16	GNRSGNGIL	0.98/-0.40156265
	88	365	GFETIMKVL	0.12/0.45554363
	89	188	IEGSGRSAP	0.83/-0.26854815
	90	349	YELRYNGAI	0.00/0.55373259
	91	392	KPQRQRGHK	0.08/0.46484825
	92	316	LAPTAGAFF	0.89/-0.3492627
	93	371	KVLSENLNA	0.02/0.51461096
	94	234	DQIASLVLA	0.87/-0.33606481
	95	229	TPDMADQIA	0.93/-0.39632129
	96	8	QSSSRASSG	0.68/-0.14745512
	97	233	ADQIASLVL	0.85/-0.32030753
	98	116	QRQLLPRWY	0.98/-0.45161339
	99	321	GAFFFGSRL	0.00/0.52367268
	100	31	DQFRNVQTR	0.01/0.47579119
	101	147	WVASNQADV	0.00/0.47524332
	102	156	NTPADIVDR	0.85/-0.37584138
	103	381	QQQDGMMNM	0.00/0.47333015
	104	210	SAGSRSRAN	0.92/-0.44776397
	105	145	VYWVASNQA	0.91/-0.45971292
	106	86	VPIAPGVPA	0.52/-0.081605456
	107	208	ASSAGSRSR	0.00/0.42280948
	108	344	PQKDVYELR	0.94/-0.51876437
	109	266	ILNKPRQKR	0.01/0.40169297
	110	48	TATSQQPSG	0.87/-0.46990769
	111	88	IAPGVPATE	0.66/-0.266765

	112	103	RHNRRSFKT	0.94/-0.55384533
	113	202	SRTSSRASS	0.67/-0.28627804
	114	263	RQKILNKPR	0.98/-0.59824813
	115	409	ISVAVPKSR	0.67/-0.29111441
	116	78	FEFAEGQGV	0.74/-0.41562362
	117	227	GVTPDMADQ	0.99/-0.67107157
	118	105	NRRSFKTAD	0.88/-0.56190498
	119	39	RGRRAQPKQ	0.68/-0.37835187
	120	217	ANSGNRTPT	0.99/-0.69366685
	121	380	YQQQDGMMN	0.92/-0.64246371
	122	260	KEVRQKILN	0.95/-0.68944282
	123	198	SRSTSRTSS	1.00/-0.78806077
	124	176	FPPGTVLPQ	0.92/-0.70942411
	125	223	TPTSGVTPD	0.66/-0.47617744
	126	417	RVQQNKSIE	0.92/-0.74387469
	127	332	AKVQNLSGN	0.90/-0.74889762
	128	272	QKRSPNKQC	0.84/-0.6929533
	129	262	VRQKILNKP	0.85/-0.71258341
	130	346	KDVYELRYN	0.84/-0.71158156
	131	143	DGVYWVASN	0.96/-0.85322507
	132	9	SSSRASSGN	0.83/-0.73755023
	133	160	DIVDRDPSS	0.93/-0.84494347
	134	269	KPRQKRSPN	0.77/-0.70191533
	135	131	GPHAKDQYG	0.51/-0.4490066
	136	382	QQDGMMNMS	0.61/-0.57713077
	137	368	TIMKVLSEN	0.61/-0.5775436
	138	275	SPNKQCTVQ	0.88/-0.86039888
	139	186	YYIEGSGRS	0.89/-0.8913775
	140	100	YWYRHNRRS	0.74/-0.80281384
	141	218	NSGNRTPTS	0.73/-0.8219241
	142	196	PNSRSTSRT	0.72/-0.82214697
	143	113	DGNQRQLLP	0.77/-0.87846238
	144	370	MKVLSENLN	0.99/-1.1110516
	145	408	NISVAVPKS	0.60/-0.73476361
	146	59	VVPYYSWFS	0.75/-0.88913655
	147	393	PQRQRGHKN	0.62/-0.76562887
	148	264	QKILNKPRQ	0.95/-1.1080764
	149	20	GNGILKWAD	0.78/-0.94466833
	150	395	RQRGHKNGQ	0.53/-0.72963819

	151	333	KVQNLSGNP	0.86/-1.0843094
	152	221	NRTPTSGVT	0.59/-0.81525772
	153	6	GKQSSSRAS	0.94/-1.1654096
	154	159	ADIVDRDPS	0.97/-1.2393525
	155	166	PSSDEAIPT	0.83/-1.1086528
	156	285	CFGKRGPNQ	0.67/-0.94935807
	157	135	KDQYGTDID	0.91/-1.1913038
	158	69	ITQFQKGKE	0.57/-0.87735713
	159	253	QVTKHTAKE	0.59/-0.90283739
	160	42	RAQPKQTAT	0.52/-0.83385458
	161	327	SRLELAKVQ	0.75/-1.0871909
	162	330	ELAKVQNLS	0.55/-0.91879418
	163	283	QQCFGKRGP	0.52/-0.90087492
	164	162	VDRDPSSDE	0.96/-1.3437042
	165	375	ENLNAYQQQ	0.71/-1.0946865
	166	396	QRGHKNGQG	0.79/-1.1780586
	167	226	SGVTPDMAD	0.66/-1.1779906
	168	341	PDEPQKDVY	0.85/-1.4332236
	169	245	GKDATKPQQ	0.58/-1.1995382
	170	247	DATKPQQVT	0.51/-1.1432332
	171	290	GPNQNFGGG	0.56/-1.2629712
	172	331	LAKVQNLSG	0.75/-1.4736735
	173	385	GMMNMSPKP	0.85/-1.6132945
	174	178	PGTVLPQGY	0.65/-1.5461551
	175	383	QDGMMNMSP	0.61/-1.5386503
	176	337	LSGNPDEPQ	0.95/-1.9712696
	177	297	GGEMLKLGT	0.91/-1.9332697
	178	45	PKQTATSQQ	0.84/-2.0144397
**HCoV-HKU1**				
**Viral protein name**	**Rank**	**Start position**	**Sequence**	**Score ANN/SVM**
**Nucleocapsid**	1	314	APTPGAFFF	1.00/1.4961973
	2	355	STLPGFETI	1.00/0.97182876
	3	32	ERSHQTYNR	0.82/0.86769761
	4	100	WYKHNRRSF	0.70/0.97369498
	5	345	LRYSGSIRF	0.96/0.71316192
	6	194	ASNSRPGSR	0.88/0.72719808
	7	16	GNRSGILKK	0.90/0.66710451
	8	69	TQFQKGRDF	0.98/0.4921251

	9	395	GVKQSPESF	0.90/0.55619454
	10	51	STQPQGNTI	0.88/0.489655
	**11**	**119**	**LPRWYFYYL**	**0.00/1.3445772**
	12	166	TIQEAIPTR	0.94/0.38718563
	13	337	SPSKDTFEL	0.78/0.51268009
	14	141	HEGIFWVAS	0.97/0.31072606
	15	162	ARDPTIQEA	0.09/1.1728461
	16	205	SRGPNNRSL	0.01/1.178805
	17	13	SSSGNRSGI	0.71/0.47744339
	18	275	NKFCNVQQC	0.88/0.29567564
	19	366	VLKENLDAY	0.30/0.85327468
	20	88	AYGIPPSEA	0.47/0.66066661
	21	94	SEAKGYWYK	0.34/0.77827761
	22	110	TADGQQKQL	0.88/0.23068034
	23	116	KQLLPRWYF	0.38/0.72259239
	24	356	TLPGFETIM	0.00/1.0953365
	25	385	SLSPKPQRK	0.38/0.70228852
	26	407	NLSADTQHI	0.86/0.21778056
	27	246	DSKPQQVTK	0.16/0.8846158
	28	125	YYLGTGPYA	0.44/0.60022141
	29	264	ILMKPRQKR	0.96/0.059271792
	30	276	KFCNVQQCF	0.70/0.30659755
	31	269	RQKRTPNKF	0.41/0.59205225
	32	235	IASLVLAKL	0.15/0.83832741
	33	363	IMKVLKENL	0.16/0.76137225
	34	139	ESHEGIFWV	0.08/0.83099216
	35	230	DMADEIASL	0.87/0.03858306
	36	231	MADEIASLV	0.64/0.26806972
	37	138	GESHEGIFW	0.81/0.094269955
	38	290	LQNFGNEML	0.81/0.091112397
	39	349	GSIRFDSTL	0.02/0.87707935
	40	272	RTPNKFCNV	0.04/0.85664392
	41	173	TRFSPGTIL	0.00/0.89520939
	42	163	RDPTIQEAI	0.98/-0.091534088
	43	136	SYGESHEGI	0.98/-0.09514377
	44	198	RPGSRSQSR	0.23/0.6542641
	45	344	ELRYSGSIR	0.82/0.052614872
	46	126	YLGTGPYAN	0.93/-0.062409977
	47	179	TILPQGYYV	0.01/0.85514416

	48	170	AIPTRFSPG	0.87/-0.0074589008
	49	39	NRGRKPQPK	0.79/0.059153307
	50	15	SGNRSGILK	0.31/0.53667082
	51	299	KLGTNDPQF	0.94/-0.093433378
	52	374	YVNSNQNTV	0.03/0.80286352
	53	373	AYVNSNQNT	0.72/0.10713683
	54	292	NFGNEMLKL	0.82/0.0061638397
	55	35	HQTYNRGRK	0.63/0.19607071
	56	302	TNDPQFPIL	0.00/0.82315451
	57	263	KILMKPRQK	0.68/0.112952
	58	343	FELRYSGSI	0.36/0.40478423
	59	42	RKPQPKFTV	0.56/0.1987804
	60	289	PLQNFGNEM	0.97/-0.23728854
	61	24	KTSWVDQSE	0.93/-0.21097773
	62	285	GKRGPLQNF	0.54/0.17107091
	63	2	SYTPGHHAG	0.28/0.41662649
	64	48	FTVSTQPQG	0.91/-0.21785461
	65	98	GYWYKHNRR	0.04/0.64327472
	66	203	SQSRGPNNR	0.28/0.39298196
	67	359	GFETIMKVL	0.21/0.45554363
	68	227	VKPDMADEI	0.88/-0.240664
	69	256	NAKEIRHKI	0.00/0.63518759
	70	321	FFGSKLELF	0.70/-0.082833362
	71	214	SRSNSNFRH	0.54/0.077132849
	72	60	PHYSWFSGI	0.96/-0.36043775
	73	252	VTKQNAKEI	0.53/0.067310078
	74	306	QFPILAELA	0.78/-0.1828691
	75	164	DPTIQEAIP	0.98/-0.38787822
	76	422	EDHSLLATL	/0.57708186
	77	142	EGIFWVASH	0.83/-0.2631943
	78	370	NLDAYVNSN	0.77/-0.20748117
	79	174	RFSPGTILP	0.84/-0.28600685
	80	124	FYYLGTGPY	0.68/-0.12632572
	81	319	AFFFGSKLE	0.96/-0.40723022
	82	117	QLLPRWYFY	0.57/-0.029293394
	83	381	TVSGSLSPK	0.16/0.37354508
	84	301	GTNDPQFPI	0.09/0.44294619
	85	234	EIASLVLAK	0.14/0.37554337

	86	57	NTIPHYSWF	0.00/0.48730721
	87	192	RSASNSRPG	0.94/-0.45394261
	88	190	SGRSASNSR	0.88/-0.39746696
	89	391	QRKRGVKQS	0.72/-0.24787469
	90	130	GPYANASYG	0.92/-0.45072802
	91	259	EIRHKILMK	0.06/0.3990221
	92	291	QNFGNEMLK	0.00/0.45211586
	93	405	SLNLSADTQ	0.83/-0.38015142
	94	122	WYFYYLGTG	1.00/-0.5715081
	95	206	RGPNNRSLS	0.98/-0.56476422
	96	212	SLSRSNSNF	0.04/0.37027036
	97	143	GIFWVASHQ	0.63/-0.23044717
	98	103	HNRRSFKTA	0.00/0.38779782
	99	204	QSRGPNNRS	0.91/-0.52645022
	100	43	KPQPKFTVS	0.67/-0.31219191
	101	62	YSWFSGITQ	0.77/-0.41601612
	102	59	IPHYSWFSG	0.98/-0.63268884
	103	376	NSNQNTVSG	0.86/-0.52848642
	104	372	DAYVNSNQN	0.92/-0.60470553
	105	128	GTGPYANAS	0.69/-0.38814964
	106	410	ADTQHISND	0.96/-0.66010948
	107	330	KRDSDADSP	0.87/-0.57057488
	108	255	QNAKEIRHK	0.53/-0.25034625
	109	278	CNVQQCFGK	0.56/-0.30105439
	110	30	QSERSHQTY	0.53/-0.2890537
	111	408	LSADTQHIS	0.56/-0.36127016
	112	186	YVEGSGRSA	0.98/-0.79492794
	113	347	YSGSIRFDS	0.98/-0.79775431
	114	409	SADTQHISN	0.55/-0.37989788
	115	351	IRFDSTLPG	0.93/-0.76138085
	116	80	PDGQGVPIA	0.61/-0.45241142
	117	176	SPGTILPQG	0.98/-0.82963646
	118	87	IAYGIPPSE	0.80/-0.65853321
	119	188	EGSGRSASN	0.80/-0.67107022
	120	397	KQSPESFDS	0.96/-0.90646967
	121	78	KFPDGQGVP	0.87/-0.87581571
	122	350	SIRFDSTLP	0.95/-0.97054987
	123	85	VPIAYGIPP	0.94/-0.97212355
	124	96	AKGYWYKHN	0.65/-0.711482

	125	399	SPESFDSLN	0.56/-0.62619157
	126	325	KLELFKRDS	0.69/-0.78342076
	127	280	VQQCFGKRG	0.86/-0.96094544
	128	38	YNRGRKPQP	0.77/-0.9033711
	129	340	KDTFELRYS	0.57/-0.7253276
	130	131	PYANASYGE	0.99/-1.1726272
	131	293	FGNEMLKLG	0.63/-0.81381149
	132	6	GHHAGSRSS	0.81/-1.0943574
	133	283	CFGKRGPLQ	0.94/-1.2487202
	134	284	FGKRGPLQN	0.78/-1.0955521
	135	383	SGSLSPKPQ	0.91/-1.260197
	136	247	SKPQQVTKQ	0.83/-1.1942034
	137	329	FKRDSDADS	0.90/-1.2730956
	138	262	HKILMKPRQ	0.98/-1.3900266
	139	382	VSGSLSPKP	0.76/-1.3047531
	140	28	VDQSERSHQ	0.57/-1.1424233
	141	404	DSLNLSADT	0.69/-1.2804554
	142	123	YFYYLGTGP	0.90/-1.5129985
	143	225	SIVKPDMAD	0.53/-1.1861916
	144	5	PGHHAGSRS	0.73/-1.389111
	145	308	PILAELAPT	0.78/-1.5495611
	146	375	VNSNQNTVS	0.65/-1.4984255
	147	245	KDSKPQQVT	0.52/-1.3880026
	148	22	LKKTSWVDQ	0.57/-1.4740862
	149	72	QKGRDFKFP	0.66/-1.651665
**SARS-COV-2**				
**Viral protein name**	**Rank**	**Start position**	**Sequence**	**Score ANN/SVM**
**Spike**	**1**	**1192**	**NLNESLIDL**	**0.92/1.5879094**
	2	236	TRFQTLLAL	0.99/1.4877641
	3	983	RLDKVEAEV	0.71/1.6892505
	4	679	NSPRRARSV	0.87/1.0916413
	5	292	ALDPLSETK	0.92/1.0117189
	6	204	YSKHTPINL	0.47/1.4169218
	**7**	**820**	**DLLFNKVTL**	**0.73/1.1189541**
	8	972	AISSVLNDI	0.99/0.84037452
	9	1125	NCDVVIGIV	0.93/0.83215588
	10	97	KSNIIRGWI	0.89/0.86720593
	**11**	**846**	**ARDLICAQK**	**0.82/0.93408287**

	12	618	TEVPVAIHA	0.98/0.76924266
	13	852	AQKFNGLTV	0.98/0.73319244
	14	115	QSLLIVNNA	0.95/0.76004022
	15	123	ATNVVIKVC	0.84/0.8570027
	16	612	YQGVNCTEV	0.69/0.96038564
	17	535	KNKCVNFNF	0.97/0.64771057
	18	54	LFLPFFSNV	0.83/0.76135348
	19	464	FERDISTEI	0.67/0.92124621
	20	735	SVDCTMYIC	0.99/0.56080212
	21	706	AYSNNSIAI	0.71/0.83826283
	22	814	KRSFIEDLL	0.84/0.70530797
	23	958	ALNTLVKQL	0.14/1.3939207
	24	1185	RLNEVAKNL	0.23/1.2586948
	25	345	TRFASVYAW	0.37/1.1119585
	26	28	YTNSFTRGV	0.99/0.4496507
	27	350	VYAWNRKRI	0.93/0.49736819
	28	171	VSQPFLMDL	0.64/0.78489273
	29	233	INITRFQTL	0.88/0.51843698
	30	119	IVNNATNVV	0.62/0.75028555
	**31**	**1060**	**VVFLHVTYV**	**0.59/0.77948535**
	32	333	TNLCPFGEV	0.87/0.49897438
	33	163	ANNCTFEYV	0.67/0.68976923
	34	336	CPFGEVFNA	0.86/0.49893355
	35	976	VLNDILSRL	0.19/1.1679864
	36	634	RVYSTGSNV	0.61/0.74770151
	37	725	EILPVSMTK	0.85/0.5027134
	38	810	SKPSKRSFI	0.98/0.37244882
	39	448	NYNYLYRLF	1.00/0.34772566
	40	502	GVGYQPYRV	0.86/0.48701691
	41	833	FIKQYGDCL	0.76/0.57526737
	42	880	GTITSGWTF	0.75/0.57483752
	43	319	RVQPTESIV	0.38/0.94410253
	44	433	VIAWNSNNL	0.82/0.4956948
	45	456	FRKSNLKPF	0.96/0.34990342
	46	836	QYGDCLGDI	0.68/0.61787268
	47	1006	TYVTQQLIR	0.99/0.29995915
	48	797	FGGFNFSQI	0.45/0.83156397
	49	280	NENGTITDA	0.99/0.2889406
	50	59	FSNVTWFHA	0.53/0.74877447

	51	399	SFVIRGDEV	0.97/0.30742774
	52	395	VYADSFVIR	0.01/1.2592861
	53	780	EVFAQVKQI	0.79/0.4771388
	54	416	GKIADYNYK	0.99/0.27547906
	55	62	VTWFHAIHV	0.41/0.85297789
	56	827	TLADAGFIK	0.26/0.99225063
	57	298	ETKCTLKSF	0.90/0.34635795
	58	318	FRVQPTESI	1.00/0.24226172
	59	925	NQFNSAIGK	0.76/0.48116482
	60	609	AVLYQGVNC	0.96/0.27961479
	61	892	AALQIPFAM	0.30/0.936978
	62	1000	RLQSLQTYV	0.12/1.1162891
	63	516	ELLHAPATV	0.39/0.84172953
	64	1010	QQLIRAAEI	0.91/0.3197616
	**65**	**903**	**AYRFNGIGV**	**0.19/1.0390298**
	66	1171	GINASVVNI	0.00/1.2240296
	67	1127	DVVIGIVNN	0.95/0.2685717
	68	677	QTNSPRRAR	0.66/0.55678436
	69	764	NRALTGIAV	0.34/0.86148546
	70	1026	ATKMSECVL	0.97/0.2116657
	**71**	**1054**	**QSAPHGVVF**	**0.44/0.73864024**
	72	460	NLKPFERDI	0.41/0.75730112
	73	973	ISSVLNDIL	0.95/0.20493016
	74	1124	GNCDVVIGI	0.04/1.1035891
	75	1055	SAPHGVVFL	0.35/0.78352262
	76	504	GYQPYRVVV	0.23/0.90122156
	77	1099	GTHWFVTQR	0.27/0.85459551
	78	382	VSPTKLNDL	0.82/0.30409279
	79	121	NNATNVVIK	0.71/0.41011789
	80	759	FCTQLNRAL	0.85/0.26316347
	81	122	NATNVVIKV	0.69/0.41830926
	82	98	SNIIRGWIF	0.09/1.0182657
	83	954	QNAQALNTL	0.70/0.40665469
	84	926	QFNSAIGKI	0.58/0.5145498
	85	386	KLNDLCFTN	0.42/0.67438404
	86	327	VRFPNITNL	0.04/1.0522648
	87	444	KVGGNYNYL	0.01/1.0792264
	88	458	KSNLKPFER	0.01/1.0757571
	89	258	WTAGAAAYY	0.54/0.5440791

	90	1164	VDLGDISGI	0.95/0.12980383
	91	757	GSFCTQLNR	0.83/0.23591759
	92	1095	FVSNGTHWF	0.61/0.45070133
	93	396	YADSFVIRG	0.00/1.0606061
	94	478	TPCNGVEGF	0.84/0.22053613
	95	392	FTNVYADSF	0.86/0.18825795
	96	1062	FLHVTYVPA	0.95/0.094842726
	97	781	VFAQVKQIY	0.71/0.33134329
	98	361	CVADYSVLY	0.83/0.20890758
	99	798	GGFNFSQIL	0.82/0.21884931
	100	1144	ELDSFKEEL	0.27/0.76364207
	101	529	KSTNLVKNK	0.99/0.032044011
	102	1163	DVDLGDISG	0.89/0.13059576
	103	324	ESIVRFPNI	0.18/0.84007409
	104	1086	KAHFPREGV	0.94/0.076210395
	105	923	IANQFNSAI	0.93/0.085106375
	106	989	AEVQIDRLI	0.86/0.1550506
	107	102	RGWIFGTTL	0.80/0.21354425
	108	685	RSVASQSII	0.57/0.43730301
	109	449	YNYLYRLFR	0.84/0.16648622
	110	1190	AKNLNESLI	0.04/0.96099358
	111	584	ILDITPCSF	0.69/0.31041717
	112	15	CVNLTTRTQ	0.98/0.019925455
	113	285	ITDAVDCAL	0.75/0.23848008
	114	89	GVYFASTEK	0.00/0.98684427
	115	55	FLPFFSNVT	0.72/0.26607576
	116	538	CVNFNFNGL	0.00/0.9787292
	117	93	ASTEKSNII	0.49/0.48581774
	118	487	NCYFPLQSY	0.74/0.22240704
	119	494	SYGFQPTNG	0.98/-0.022026824
	120	138	DPFLGVYYH	0.41/0.54564184
	121	1181	KEIDRLNEV	0.00/0.95542235
	122	208	TPINLVRDL	0.01/0.93604158
	**123**	**951**	**VVNQNAQAL**	**0.16/0.78381232**
	124	135	FCNDPFLGV	0.00/0.94369976
	125	252	GDSSSGWTA	0.79/0.15356838
	126	153	MESEFRVYS	0.90/0.042492164
	127	387	LNDLCFTNV	0.31/0.63039809
	128	268	GYLQPRTFL	0.25/0.68904137

	129	13	SQCVNLTTR	0.71/0.22569446
	130	255	SSGWTAGAA	0.82/0.10844838
	131	198	DGYFKIYSK	0.75/0.17701473
	132	847	RDLICAQKF	0.93/-0.0040749685
	133	342	FNATRFASV	0.18/0.74271004
	134	1067	YVPAQEKNF	0.94/-0.021668534
	135	509	RVVVLSFEL	0.54/0.37790287
	136	869	MIAQYTSAL	0.41/0.50570354
	137	413	GQTGKIADY	0.95/-0.036742046
	138	474	QAGSTPCNG	0.73/0.17553918
	139	530	STNLVKNKC	0.77/0.13357426
	140	295	PLSETKCTL	0.88/0.023422332
	141	139	PFLGVYYHK	0.56/0.3361685
	142	155	SEFRVYSSA	0.44/0.44339405
	143	987	VEAEVQIDR	0.34/0.53761645
	144	1076	TTAPAICHD	0.46/0.41720267
	145	675	QTQTNSPRR	0.90/-0.025268287
	146	554	ESNKKFLPF	0.99/-0.11633723
	147	787	QIYKTPPIK	0.71/0.16240686
	148	525	CGPKKSTNL	0.81/0.06208729
	149	536	NKCVNFNFN	0.99/-0.11902214
	150	576	VRDPQTLEI	0.06/0.81001705
	151	940	STASALGKL	0.98/-0.11279199
	152	559	FLPFQQFGR	0.80/0.066311857
	153	400	FVIRGDEVR	0.89/-0.02750081
	154	238	FQTLLALHR	0.85/0.0028903032
	155	30	NSFTRGVYY	0.02/0.83054514
	156	1007	YVTQQLIRA	0.67/0.18003803
	157	533	LVKNKCVNF	0.00/0.85001899
	158	1118	DNTFVSGNC	0.95/-0.1022437
	159	409	QIAPGQTGK	0.00/0.83303707
	**160**	**901**	**QMAYRFNGI**	**0.13/0.69832921**
	**161**	**1208**	**QYIKWPWYI**	**/0.82786365**
	162	313	YQTSNFRVQ	0.99/-0.16260307
	163	1123	SGNCDVVIG	0.87/-0.044511101
	164	912	TQNVLYENQ	0.95/-0.12643186
	165	1115	ITTDNTFVS	0.79/0.031855701
	166	378	KCYGVSPTK	0.99/-0.17129001

	167	532	NLVKNKCVN	0.85/-0.03143559
	168	189	LREFVFKNI	0.13/0.68287982
	169	348	ASVYAWNRK	0.41/0.4028606
	170	568	DIADTTDAV	0.01/0.79165536
	171	1016	AEIRASANL	0.78/0.017047345
	172	379	CYGVSPTKL	0.00/0.79227611
	173	270	LQPRTFLLK	0.00/0.78938338
	174	777	NTQEVFAQV	0.10/0.68271776
	175	77	KRFDNPVLP	0.72/0.060755904
	176	965	QLSSNFGAI	0.56/0.21791063
	177	84	LPFNDGVYF	0.80/-0.022575978
	178	353	WNRKRISNC	0.62/0.14927305
	179	360	NCVADYSVL	0.94/-0.17191863
	180	1096	VSNGTHWFV	0.32/0.44642728
	181	687	VASQSIIAY	0.52/0.24420376
	182	883	TSGWTFGAG	0.67/0.087730892
	183	975	SVLNDILSR	0.90/-0.14367289
	184	755	QYGSFCTQL	0.02/0.73586609
	**185**	**1041**	**DFCGKGYHL**	**0.02/0.73481455**
	186	1121	FVSGNCDVV	0.18/0.57456887
	187	1051	SFPQSAPHG	0.80/-0.045461664
	188	206	KHTPINLVR	0.94/-0.18910122
	189	676	TQTNSPRRA	0.90/-0.15761293
	190	417	KIADYNYKL	0.00/0.74005063
	191	697	MSLGAENSV	0.91/-0.17159345
	192	447	GNYNYLYRL	0.01/0.72750718
	193	782	FAQVKQIYK	0.22/0.51645801
	194	23	QLPPAYTNS	0.91/-0.1743011
	195	999	GRLQSLQTY	0.56/0.17547758
	196	1137	VYDPLQPEL	0.14/0.59484561
	197	1226	AIVMVTIML	/0.73431223
	198	169	EYVSQPFLM	0.00/0.7324723
	199	264	AYYVGYLQP	0.95/-0.21896143
	200	630	TPTWRVYST	0.64/0.090783366
	201	614	GVNCTEVPV	0.00/0.72983584
	202	227	VDLPIGINI	0.13/0.59776359
	203	1150	EELDKYFKN	0.91/-0.18583258
	**204**	**1056**	**APHGVVFLH**	**0.14/0.57504297**
	205	592	FGGVSVITP	0.97/-0.25743026

	206	894	LQIPFAMQM	0.74/-0.037819834
	207	234	NITRFQTLL	0.57/0.13034199
	208	664	IPIGAGICA	0.78/-0.081495116
	209	321	QPTESIVRF	0.14/0.55765263
	210	253	DSSSGWTAG	0.72/-0.023444227
	211	27	AYTNSFTRG	0.56/0.13432448
	212	550	GVLTESNKK	0.67/0.022718272
	213	193	VFKNIDGYF	0.84/-0.14767335
	214	660	YECDIPIGA	0.07/0.62181879
	215	288	AVDCALDPL	0.53/0.15420622
	**216**	**1209**	**YIKWPWYIW**	**/0.68245283**
	217	1204	GKYEQYIKW	0.01/0.6658816
	218	1085	GKAHFPREG	0.94/-0.27137105
	219	734	TSVDCTMYI	0.07/0.59295884
	220	312	IYQTSNFRV	0.27/0.39102148
	221	46	SVLHSTQDL	0.59/0.070628293
	222	389	DLCFTNVYA	0.06/0.59541601
	223	501	NGVGYQPYR	0.63/0.025251303
	**224**	**919**	**NQKLIANQF**	**0.00/0.65422365**
	225	269	YLQPRTFLL	0.01/0.64342282
	226	354	NRKRISNCV	0.01/0.63952346
	227	627	DQLTPTWRV	0.00/0.6487372
	228	446	GGNYNYLYR	0.00/0.64830171
	229	394	NVYADSFVI	0.76/-0.11532989
	230	32	FTRGVYYPD	0.91/-0.26674456
	231	370	NSASFSTFK	0.01/0.63262149
	232	832	GFIKQYGDC	0.85/-0.20761876
	233	1196	SLIDLQELG	0.75/-0.11355354
	234	1210	IKWPWYIWL	/0.63616187
	235	907	NGIGVTQNV	0.03/0.60456213
	236	956	AQALNTLVK	0.00/0.62945518
	237	712	IAIPTNFTI	0.01/0.6169805
	238	450	NYLYRLFRK	0.00/0.62171566
	239	1262	EPVLKGVKL	/0.61158557
	240	1165	DLGDISGIN	0.84/-0.23244334
	241	505	YQPYRVVVL	0.01/0.5965429
	242	688	ASQSIIAYT	0.99/-0.3882286
	243	1183	IDRLNEVAK	0.01/0.59035876
	244	1122	VSGNCDVVI	0.68/-0.07996517

	245	149	NKSWMESEF	0.77/-0.1756724
	246	626	ADQLTPTWR	0.93/-0.33882418
	247	1194	NESLIDLQE	0.77/-0.17943301
	248	334	NLCPFGEVF	0.00/0.58858915
	249	95	TEKSNIIRG	0.07/0.51324547
	250	86	FNDGVYFAS	0.84/-0.2570983
	251	136	CNDPFLGVY	0.00/0.58175107
	252	221	SALEPLVDL	0.10/0.47975488
	253	686	SVASQSIIA	0.12/0.45958209
	254	997	ITGRLQSLQ	0.82/-0.24138811
	255	38	YPDKVFRSS	0.90/-0.32638397
	256	1169	ISGINASVV	0.00/0.56782952
	257	186	FKNLREFVF	0.19/0.37421466
	258	1044	GKGYHLMSF	0.80/-0.23905486
	259	402	IRGDEVRQI	0.02/0.53867845
	260	410	IAPGQTGKI	0.12/0.43746731
	261	3	VFLVLLPLV	0.95/-0.39272787
	262	993	IDRLITGRL	0.96/-0.40288235
	263	477	STPCNGVEG	0.68/-0.12441887
	264	1221	IAGLIAIVM	/0.5530776
	265	83	VLPFNDGVY	0.90/-0.34931234
	266	1133	VNNTVYDPL	0.91/-0.36386937
	267	1175	SVVNIQKEI	0.92/-0.37518574
	268	1195	ESLIDLQEL	0.04/0.50136368
	269	112	SKTQSLLIV	0.65/-0.11336405
	270	1173	NASVVNIQK	0.04/0.48923419
	271	42	VFRSSVLHS	0.78/-0.25143064
	272	60	SNVTWFHAI	0.00/0.52175752
	273	1028	KMSECVLGQ	0.86/-0.33830367
	274	691	SIIAYTMSL	0.08/0.4387551
	275	125	NVVIKVCEF	0.08/0.43765697
	276	916	LYENQKLIA	0.98/-0.46793709
	277	250	TPGDSSSGW	0.72/-0.20818618
	278	187	KNLREFVFK	0.03/0.48025447
	279	551	VLTESNKKF	0.91/-0.40127646
	280	754	LQYGSFCTQ	0.73/-0.22195096
	281	708	SNNSIAIPT	0.97/-0.46238529
	282	50	STQDLFLPF	0.96/-0.45381589
	283	563	QQFGRDIAD	0.97/-0.46484265

	284	75	GTKRFDNPV	0.02/0.48077201
	285	813	SKRSFIEDL	0.55/-0.054529485
	286	644	QTRAGCLIG	0.94/-0.44643291
	287	748	ECSNLLLQY	0.86/-0.36907603
	288	701	AENSVAYSN	0.06/0.42092325
	289	710	NSIAIPTNF	0.00/0.48057058
	290	215	DLPQGFSAL	0.00/0.47967982
	291	195	KNIDGYFKI	0.04/0.43907249
	292	465	ERDISTEIY	0.04/0.43514758
	293	1224	LIAIVMVTI	/0.47352128
	294	1081	ICHDGKAHF	0.00/0.47336462
	295	678	TNSPRRARS	0.00/0.46942667
	296	962	LVKQLSSNF	0.62/-0.15274303
	297	986	KVEAEVQID	0.99/-0.52449453
	298	85	PFNDGVYFA	0.02/0.44528067
	299	1106	QRNFYEPQI	0.00/0.46521579
	300	1129	VIGIVNNTV	0.00/0.45624515
	301	655	HVNNSYECD	0.98/-0.52703499
	302	898	FAMQMAYRF	0.59/-0.13988547
	303	783	AQVKQIYKT	1.00/-0.55131466
	304	1214	WYIWLGFIA	/0.44831422
	305	645	TRAGCLIGA	0.01/0.4360915
	306	88	DGVYFASTE	0.88/-0.43436613
	307	1102	WFVTQRNFY	0.01/0.43437486
	308	403	RGDEVRQIA	0.01/0.41897524
	309	63	TWFHAIHVS	0.93/-0.51246264
	310	1078	APAICHDGK	0.65/-0.23312101
	311	239	QTLLALHRS	0.89/-0.47398884
	312	182	KQGNFKNLR	0.03/0.38099958
	313	2	FVFLVLLPL	0.00/0.40759495
	314	704	SVAYSNNSI	0.03/0.37331941
	315	937	SLSSTASAL	0.00/0.40309069
	316	260	AGAAAYYVG	0.91/-0.51126797
	317	499	PTNGVGYQP	0.91/-0.51404835
	**318**	**1219**	**GFIAGLIAI**	**/0.38830748**
	319	235	ITRFQTLLA	0.00/0.38611895
	320	1264	VLKGVKLHY	/0.38501663
	321	1119	NTFVSGNCD	0.97/-0.59392325
	322	596	SVITPGTNT	0.58/-0.22309952

	323	1167	GDISGINAS	0.90/-0.55720895
	324	854	KFNGLTVLP	0.53/-0.18989855
	325	776	KNTQEVFAQ	0.92/-0.58719275
	326	917	YENQKLIAN	0.54/-0.20739899
	327	263	AAYYVGYLQ	0.90/-0.5712202
	328	214	RDLPQGFSA	0.84/-0.5154096
	329	1203	LGKYEQYIK	0.86/-0.5409742
	330	473	YQAGSTPCN	0.95/-0.63128422
	331	913	QNVLYENQK	0.52/-0.20458317
	332	423	YKLPDDFTG	0.81/-0.49564959
	333	950	DVVNQNAQA	0.68/-0.36599599
	334	484	EGFNCYFPL	0.68/-0.36825199
	335	768	TGIAVEQDK	0.55/-0.24021556
	336	896	IPFAMQMAY	0.97/-0.66172733
	337	352	AWNRKRISN	0.63/-0.32440239
	338	226	LVDLPIGIN	0.70/-0.39579694
	339	517	LLHAPATVC	0.85/-0.54749701
	340	1161	SPDVDLGDI	0.59/-0.2922528
	341	81	NPVLPFNDG	0.63/-0.348098
	342	904	YRFNGIGVT	0.85/-0.56977742
	343	763	LNRALTGIA	0.95/-0.67111733
	344	190	REFVFKNID	0.72/-0.44130943
	345	695	YTMSLGAEN	0.98/-0.70186504
	346	70	VSGTNGTKR	0.63/-0.35311067
	347	693	IAYTMSLGA	0.82/-0.54699421
	348	161	SSANNCTFE	0.63/-0.36027531
	349	1075	FTTAPAICH	0.92/-0.65180223
	350	796	DFGGFNFSQ	0.68/-0.41568299
	351	340	EVFNATRFA	0.75/-0.49056096
	352	909	IGVTQNVLY	0.93/-0.67251748
	353	146	HKNNKSWME	0.98/-0.72398028
	354	673	SYQTQTNSP	0.99/-0.7400553
	355	337	PFGEVFNAT	0.63/-0.38873589
	356	835	KQYGDCLGD	0.98/-0.7445586
	357	571	DTTDAVRDP	0.68/-0.44612401
	358	66	HAIHVSGTN	0.93/-0.70085024
	359	362	VADYSVLYN	0.67/-0.44691471
	360	212	LVRDLPQGF	0.90/-0.68007362
	361	1023	NLAATKMSE	0.53/-0.31242396

	362	598	ITPGTNTSN	0.93/-0.73041048
	363	586	DITPCSFGG	0.97/-0.77631283
	364	126	VVIKVCEFQ	0.61/-0.42185546
	365	930	AIGKIQDSL	0.58/-0.3943784
	366	430	TGCVIAWNS	0.76/-0.5860783
	367	1029	MSECVLGQS	0.93/-0.75851111
	368	581	TLEILDITP	0.63/-0.46402363
	369	597	VITPGTNTS	0.75/-0.5957304
	370	113	KTQSLLIVN	0.53/-0.38088275
	371	955	NAQALNTLV	0.73/-0.58185384
	372	1097	SNGTHWFVT	0.80/-0.65699727
	373	893	ALQIPFAMQ	0.65/-0.5129913
	374	64	WFHAIHVSG	0.57/-0.43580992
	375	1017	EIRASANLA	0.83/-0.69786445
	376	1112	PQIITTDNT	0.93/-0.79950416
	377	110	LDSKTQSLL	0.77/-0.64316734
	378	247	SYLTPGDSS	0.95/-0.82460435
	379	611	LYQGVNCTE	0.98/-0.86124867
	380	48	LHSTQDLFL	0.80/-0.70004985
	381	1117	TDNTFVSGN	0.55/-0.45005215
	382	713	AIPTNFTIS	0.81/-0.71497488
	383	885	GWTFGAGAA	0.58/-0.48541337
	384	621	PVAIHADQL	0.68/-0.59137254
	385	1187	NEVAKNLNE	0.62/-0.53369454
	386	426	PDDFTGCVI	0.65/-0.5747398
	387	291	CALDPLSET	0.56/-0.48548595
	388	741	YICGDSTEC	0.59/-0.51750479
	389	231	IGINITRFQ	0.87/-0.80177053
	390	726	ILPVSMTKT	0.63/-0.5635257
	391	633	WRVYSTGSN	0.80/-0.74120623
	392	276	LLKYNENGT	0.98/-0.9406483
	393	657	NNSYECDIP	0.99/-0.962859
	394	74	NGTKRFDNP	0.59/-0.56674644
	395	5	LVLLPLVSS	0.97/-0.947971
	396	1158	NHTSPDVDL	0.86/-0.84283319
	397	225	PLVDLPIGI	0.54/-0.52759395
	398	573	TDAVRDPQT	0.70/-0.70467958
	399	1025	AATKMSECV	0.70/-0.70789169
	400	566	GRDIADTTD	0.68/-0.70104485

	401	1159	HTSPDVDLG	0.57/-0.59425387
	402	580	QTLEILDIT	0.99/-1.0148329
	403	730	SMTKTSVDC	0.93/-0.96031133
	404	547	TGTGVLTES	0.63/-0.66250487
	405	245	HRSYLTPGD	0.94/-0.97707267
	406	391	CFTNVYADS	0.61/-0.64858188
	407	911	VTQNVLYEN	0.89/-0.93287129
	408	863	PLLTDEMIA	0.88/-0.93527199
	409	522	ATVCGPKKS	0.93/-0.99606588
	410	498	QPTNGVGYQ	0.60/-0.66984026
	411	6	VLLPLVSSQ	0.69/-0.76803716
	412	750	SNLLLQYGS	0.51/-0.61301734
	413	756	YGSFCTQLN	0.87/-0.97427523
	414	665	PIGAGICAS	0.85/-0.95602426
	415	49	HSTQDLFLP	0.91/-1.0195034
	416	646	RAGCLIGAE	0.68/-0.80187557
	417	770	IAVEQDKNT	0.94/-1.0740015
	418	981	LSRLDKVEA	0.60/-0.74646517
	419	132	EFQFCNDPF	0.51/-0.67539709
	420	80	DNPVLPFND	0.77/-0.93836403
	421	539	VNFNFNGLT	0.85/-1.0241041
	422	52	QDLFLPFFS	0.69/-0.88010274
	423	4	FLVLLPLVS	0.69/-0.88344636
	424	19	TTRTQLPPA	0.56/-0.75895321
	425	463	PFERDISTE	0.85/-1.0494737
	426	471	EIYQAGSTP	0.56/-0.80393159
	427	578	DPQTLEILD	0.77/-1.0158216
	428	938	LSSTASALG	0.83/-1.0760172
	429	440	NLDSKVGGN	0.96/-1.2071085
	430	789	YKTPPIKDF	0.51/-0.76410238
	431	1047	YHLMSFPQS	0.97/-1.2266547
	432	124	TNVVIKVCE	0.89/-1.1756177
	433	873	YTSALLAGT	0.79/-1.0772384
	434	545	GLTGTGVLT	0.68/-0.97105971
	435	952	VNQNAQALN	0.87/-1.1959752
	436	108	TTLDSKTQS	0.55/-0.8761585
	437	492	LQSYGFQPT	0.95/-1.2840322
	438	7	LLPLVSSQC	0.91/-1.2472776
	439	224	EPLVDLPIG	0.88/-1.2198089

	440	375	STFKCYGVS	0.87/-1.2291059
	441	438	SNNLDSKVG	0.74/-1.1128457
	442	858	LTVLPPLLT	0.61/-0.98417306
	443	173	QPFLMDLEG	0.65/-1.0346008
	444	931	IGKIQDSLS	0.54/-0.93081472
	445	544	NGLTGTGVL	0.80/-1.2226609
	446	414	QTGKIADYN	0.68/-1.1328443
	447	1148	FKEELDKYF	0.55/-1.0106407
	448	385	TKLNDLCFT	0.74/-1.2190256
	449	541	FNFNGLTGT	0.59/-1.0862641
	450	1069	PAQEKNFTT	0.57/-1.0798732
	451	1186	LNEVAKNLN	0.99/-1.5031759
	452	932	GKIQDSLSS	0.91/-1.4344021
	453	282	NGTITDAVD	0.81/-1.3404228
	454	792	PPIKDFGGF	0.64/-1.1739676
	455	632	TWRVYSTGS	0.97/-1.5043721
	456	650	LIGAEHVNN	0.63/-1.1938114
	457	216	LPQGFSALE	0.58/-1.1812157
	458	244	LHRSYLTPG	0.68/-1.3189648
	459	25	PPAYTNSFT	0.60/-1.269398
	460	309	EKGIYQTSN	0.66/-1.3328727
	461	963	VKQLSSNFG	0.76/-1.4346284
	462	1138	YDPLQPELD	0.96/-1.7567044
	463	727	LPVSMTKTS	0.94/-1.8342409
	464	1162	PDVDLGDIS	0.75/-1.685451
	465	1145	LDSFKEELD	0.75/-1.6857213
	466	1142	QPELDSFKE	0.82/-1.796165
	467	491	PLQSYGFQP	0.55/-1.5478997
	468	802	FSQILPDPS	0.60/-1.6421824
	469	945	LGKLQDVVN	0.60/-1.7300742
	470	613	QGVNCTEVP	0.59/-1.7221469
**SARS-CoV**				
**Viral protein name**	**Rank**	**Start position**	**Sequence**	**Score ANN/SVM**
**Spike**	1	1153	GINASVVNI	0.97/1.2240296
	2	982	RLQSLQTYV	0.84/1.1162891
	3	188	KNKDGFLYV	0.74/1.2048106
	4	447	KLRPFERDI	0.87/1.0022281
	5	162	EYISDAFSL	0.79/1.0588161

	6	1071	FPREGVFVF	0.83/1.013369
	7	250	AAYFVGYLK	0.99/0.80755975
	**8**	**1042**	**VVFLHVTYV**	**0.99/0.77948535**
	9	382	VYADSFVVK	0.76/0.94660302
	10	965	RLDKVEAEV	0.00/1.6892505
	**11**	**883**	**QMAYRFNGI**	**0.98/0.69832921**
	**12**	**1023**	**DFCGKGYHL**	**0.94/0.73481455**
	13	834	AQKFNGLTV	0.93/0.73319244
	**14**	**1174**	**NLNESLIDL**	**0.06/1.5879094**
	15	403	GVIADYNYK	0.90/0.72832925
	16	1084	WFITQRNFF	0.89/0.73497574
	17	366	CYGVSATKL	0.92/0.68821391
	18	1186	GKYEQYIKW	0.94/0.6658816
	19	439	KYRYLRHGK	0.98/0.60308771
	20	958	VLNDILSRL	0.41/1.1679864
	21	818	QYGECLGDI	0.96/0.54398657
	22	502	ELLNAPATV	0.77/0.7335387
	23	809	TLADAGFMK	0.80/0.7032719
	24	115	IIINNSTNV	0.95/0.54319162
	25	1063	ICHEGKAYF	0.98/0.49852803
	26	311	GDVVRFPNI	0.87/0.60715172
	27	314	VRFPNITNL	0.42/1.0522648
	28	320	TNLCPFGEV	0.97/0.49897438
	29	156	AFNCTFEYI	0.55/0.88025204
	30	356	YNSTFFSTF	0.86/0.56550332
	31	175	KSGNFKHLR	0.80/0.62538011
	32	796	KRSFIEDLL	0.72/0.70530797
	33	415	DFMGCVLAW	0.99/0.43251469
	34	940	ALNTLVKQL	0.02/1.3939207
	35	717	SVDCNMYIC	0.58/0.83015798
	36	726	GDSTECANL	0.97/0.43466714
	37	236	TAFSPAQDI	0.98/0.40751572
	38	562	VRDPKTSEI	0.26/1.1009364
	39	119	NSTNVVIRA	0.85/0.50140518
	40	954	AISSVLNDI	0.50/0.84037452
	41	1088	QRNFFSPQI	0.41/0.92732642
	42	1054	ERNFTTAPA	0.98/0.34762297
	43	390	KGDDVRQIA	0.90/0.42722844
	44	171	DVSEKSGNF	0.99/0.33539521

	45	755	EQDRNTREV	0.69/0.63416129
	46	404	VIADYNYKL	1.00/0.31666138
	47	55	TQDLFLPFY	0.84/0.43933781
	48	1167	RLNEVAKNL	0.02/1.2586948
	49	267	DENGTITDA	0.88/0.38987142
	50	1070	YFPREGVFV	0.98/0.28571274
	51	201	QPIDVVRDL	0.09/1.1565693
	52	604	TDVSTAIHA	0.84/0.40572419
	53	435	NYNYKYRYL	0.03/1.2104636
	54	321	NLCPFGEVF	0.65/0.58858915
	55	440	YRYLRHGKL	0.95/0.28440489
	56	282	PLAELKCSV	0.99/0.23875545
	57	919	SLTTTSTAL	0.71/0.50881171
	58	108	NNKSQSVII	0.94/0.27236091
	59	947	QLSSNFGAI	0.99/0.21791063
	60	874	AALQIPFAM	0.25/0.936978
	61	369	VSATKLNDL	0.79/0.39639835
	62	223	PLGINITNF	0.98/0.20545682
	63	120	STNVVIRAC	0.38/0.80215202
	64	374	LNDLCFSNV	0.45/0.73166267
	65	671	QKSIVAYTM	0.90/0.27893316
	66	418	GCVLAWNTR	0.97/0.2030597
	67	616	TPAWRIYST	0.74/0.42093465
	68	590	ASSEVAVLY	0.34/0.81690548
	69	2	FIFLLFLTL	0.59/0.56257776
	70	1017	GQSKRVDFC	0.99/0.15997079
	**71**	**802**	**DLLFNKVTL**	**0.01/1.1189541**
	72	226	INITNFRAI	0.81/0.31891554
	73	658	ASYHTVSLL	0.01/1.1156833
	74	554	DVSDFTDSV	0.11/1.0096042
	75	667	RSTSQKSIV	0.98/0.13520779
	**76**	**828**	**ARDLICAQK**	**0.18/0.93408287**
	77	1106	GNCDVVIGI	0.01/1.1035891
	78	764	FAQVKQMYK	0.80/0.30544036
	79	1177	ESLIDLQEL	0.60/0.50136368
	80	431	TSTGNYNYK	0.48/0.61544296
	81	1069	AYFPREGVF	0.44/0.64684427
	82	981	GRLQSLQTY	0.91/0.17547758
	83	1073	REGVFVFNG	0.79/0.29511842

	84	323	CPFGEVFNA	0.58/0.49893355
	85	1002	ASANLAATK	0.81/0.26402875
	86	953	GAISSVLND	1.00/0.06918826
	87	122	NVVIRACNF	0.26/0.80007167
	88	40	VYYPDEIFR	0.76/0.29961556
	89	759	NTREVFAQV	0.06/0.9893323
	90	420	VLAWNTRNI	0.23/0.81608829
	91	490	GYQPYRVVV	0.14/0.90122156
	92	763	VFAQVKQMY	0.99/0.049718937
	93	389	VKGDDVRQI	0.78/0.25960269
	94	938	AQALNTLVK	0.41/0.62945518
	**95**	**885**	**AYRFNGIGV**	**0.00/1.0390298**
	96	125	IRACNFELC	0.80/0.23153875
	97	68	GFHTINHTF	0.47/0.55733551
	98	1119	VYDPLQPEL	0.43/0.59484561
	99	753	AAEQDRNTR	0.46/0.55838722
	100	760	TREVFAQVK	0.60/0.41458855
	101	998	AEIRASANL	0.99/0.017047345
	102	733	NLLLQYGSF	0.93/0.063908892
	103	289	SVKSFEIDK	0.17/0.82158984
	104	524	CVNFNFNGL	0.01/0.9787292
	105	1163	KEIDRLNEV	0.03/0.95542235
	106	1103	FVSGNCDVV	0.41/0.57456887
	107	306	RVVPSGDVV	0.71/0.26777445
	**108**	**1190**	**QYIKWPWYV**	**/0.975564**
	109	332	TKFPSVYAW	0.09/0.88229049
	110	646	YECDIPIGA	0.35/0.62181879
	111	255	GYLKPTTFM	0.70/0.27103346
	112	1083	SWFITQRNF	0.99/-0.020811536
	113	832	ICAQKFNGL	1.00/-0.033742263
	114	829	RDLICAQKF	0.97/-0.0040749685
	115	897	VLYENQKQI	0.78/0.18363948
	116	1172	AKNLNESLI	0.00/0.96099358
	117	799	FIEDLLFNK	0.95/0.0075979248
	118	1033	SFPQAAPHG	0.86/0.095792957
	119	101	WVFGSTMNN	0.81/0.14346986
	120	109	NKSQSVIII	0.55/0.39016662
	121	688	AYSNNTIAI	0.19/0.74549251

	122	82	PFKDGIYFA	0.90/0.029161163
	123	780	GGFNFSQIL	0.71/0.21884931
	124	707	EVMPVSMAK	0.22/0.70548911
	**125**	**933**	**VVNQNAQAL**	**0.14/0.78381232**
	126	1020	KRVDFCGKG	0.98/-0.059610779
	127	437	NYKYRYLRH	0.27/0.64150725
	128	595	AVLYQDVNC	0.87/0.038606135
	129	229	TNFRAILTA	0.44/0.46188632
	130	132	LCDNPFFAV	0.44/0.46102711
	131	76	FGNPVIPFK	0.78/0.12097698
	132	1039	PHGVVFLHV	0.93/-0.030721353
	133	737	QYGSFCTQL	0.16/0.73586609
	134	174	EKSGNFKHL	0.00/0.89543681
	135	908	QFNKAISQI	0.47/0.4236368
	136	1165	IDRLNEVAK	0.30/0.59035876
	137	436	YNYKYRYLR	0.88/0.0067398286
	138	457	NVPFSPDGK	0.80/0.08543008
	139	971	AEVQIDRLI	0.73/0.1550506
	140	371	ATKLNDLCF	0.57/0.31481201
	141	300	YQTSNFRVV	0.06/0.8233986
	142	716	TSVDCNMYI	0.23/0.65244227
	143	555	VSDFTDSVR	0.87/0.0086210083
	144	543	KRFQPFQQF	0.35/0.52788471
	145	308	VPSGDVVRF	0.35/0.52506127
	146	1089	RNFFSPQII	0.41/0.46333694
	147	884	MAYRFNGIG	0.88/-0.0076877628
	148	1166	DRLNEVAKN	0.98/-0.11756115
	149	996	RAAEIRASA	0.72/0.13858567
	150	703	SITTEVMPV	0.57/0.28665971
	151	693	TIAIPTNFS	0.93/-0.07363762
	**152**	**1036**	**QAAPHGVVF**	**0.18/0.67508934**
	153	52	LYLTQDLFL	0.70/0.14707418
	154	1154	INASVVNIQ	0.79/0.056617765
	155	117	INNSTNVVI	0.65/0.1916925
	156	519	LIKNQCVNF	0.55/0.28881045
	157	353	SVLYNSTFF	0.88/-0.044011066
	158	807	KVTLADAGF	0.99/-0.15789446
	159	779	FGGFNFSQI	0.00/0.83156397

	160	862	GTATAGWTF	0.39/0.44146214
	161	727	DSTECANLL	0.25/0.58096964
	162	969	VEAEVQIDR	0.29/0.53761645
	163	944	LVKQLSSNF	0.97/-0.15274303
	164	44	DEIFRSDTL	0.28/0.53527504
	165	589	NASSEVAVL	0.31/0.50025079
	166	625	GNNVFQTQA	0.94/-0.13078131
	167	686	SIAYSNNTI	0.77/0.038400848
	168	279	SQNPLAELK	0.00/0.80511986
	169	58	LFLPFYSNV	0.29/0.51471164
	170	182	LREFVFKNK	0.28/0.52209339
	171	1176	NESLIDLQE	0.98/-0.17943301
	172	297	KGIYQTSNF	0.66/0.13239
	173	256	YLKPTTFML	0.00/0.78015806
	174	889	NGIGVTQNV	0.17/0.60456213
	175	668	STSQKSIVA	0.94/-0.16673334
	176	208	DLPSGFNTL	0.05/0.72145971
	177	94	KSNVVRGWV	0.09/0.68135937
	178	1150	DISGINASV	0.80/-0.028901047
	179	433	TGNYNYKYR	0.02/0.74372612
	180	1126	ELDSFKEEL	0.00/0.76364207
	181	1026	GKGYHLMSF	1.00/-0.23905486
	182	769	QMYKTPTLK	0.73/0.028274204
	183	1145	DVDLGDISG	0.62/0.13059576
	184	358	STFFSTFKC	0.81/-0.061051474
	185	230	NFRAILTAF	0.18/0.56811097
	186	443	LRHGKLRPF	0.22/0.51348983
	187	1037	AAPHGVVFL	0.00/0.73228057
	188	620	RIYSTGNNV	0.00/0.73177713
	189	1024	FCGKGYHLM	0.70/0.029800619
	190	95	SNVVRGWVF	0.57/0.15189833
	191	791	PLKPTKRSF	0.93/-0.21777967
	192	1151	ISGINASVV	0.14/0.56782952
	193	1159	VNIQKEIDR	0.97/-0.26534154
	194	880	FAMQMAYRF	0.84/-0.13988547
	195	501	FELLNAPAT	0.51/0.1899194
	196	785	SQILPDPLK	0.97/-0.27104059
	197	494	YRVVVLSFE	0.87/-0.17477017
	198	621	IYSTGNNVF	0.21/0.48460579

	199	1056	NFTTAPAIC	0.99/-0.29928407
	200	131	ELCDNPFFA	0.02/0.66894397
	201	761	REVFAQVKQ	0.80/-0.11119255
	202	149	HTMIFDNAF	0.02/0.66053968
	203	935	NQNAQALNT	0.99/-0.31166887
	204	377	LCFSNVYAD	0.97/-0.29241058
	205	407	DYNYKLPDD	0.96/-0.28377689
	206	921	TTTSTALGK	0.60/0.073904519
	207	839	GLTVLPPLL	0.67/0.0030849041
	208	294	EIDKGIYQT	0.99/-0.32034458
	209	86	GIYFAATEK	0.00/0.66826612
	210	47	FRSDTLYLT	0.94/-0.27964059
	211	503	LLNAPATVC	0.93/-0.2705496
	212	118	NNSTNVVIR	0.00/0.6543242
	213	441	RYLRHGKLR	0.68/-0.033318088
	214	475	YWPLNDYGF	0.99/-0.34362042
	215	34	TSSMRGVYY	0.00/0.64601526
	216	1081	GTSWFITQR	0.01/0.63397739
	217	694	IAIPTNFSI	0.00/0.64266886
	218	153	FDNAFNCTF	0.71/-0.0684593
	219	805	FNKVTLADA	0.95/-0.30930826
	220	903	KQIANQFNK	0.06/0.58043944
	221	498	VLSFELLNA	0.02/0.61958251
	222	17	DRCTTFDDV	0.22/0.41115954
	223	278	CSQNPLAEL	0.00/0.62904085
	224	1049	YVPSQERNF	0.00/0.62810603
	225	577	SFGGVSVIT	0.72/-0.092513133
	226	1107	NCDVVIGII	0.04/0.58602062
	227	331	ATKFPSVYA	0.03/0.59209716
	228	315	RFPNITNLC	0.61/0.010634901
	229	1109	DVVIGIINN	0.25/0.3701283
	230	1100	DNTFVSGNC	0.72/-0.1022437
	231	491	YQPYRVVVL	0.02/0.5965429
	232	1164	EIDRLNEVA	0.97/-0.35660241
	233	1244	EPVLKGVKL	/0.61158557
	234	729	TECANLLLQ	0.88/-0.27126781
	235	473	NCYWPLNDY	0.17/0.43703774
	236	31	TQHTSSMRG	0.85/-0.24366779
	237	858	ALVSGTATA	0.06/0.54390801

	238	373	KLNDLCFSN	0.00/0.59853344
	239	746	NRALSGIAA	0.04/0.5554449
	240	464	GKPCTPPAL	0.02/0.56591254
	241	516	STDLIKNQC	0.08/0.50549987
	**242**	**1038**	**APHGVVFLH**	**0.01/0.57504297**
	243	613	DQLTPAWRI	0.15/0.43459649
	244	220	FKLPLGINI	0.00/0.57760919
	245	106	TMNNKSQSV	0.83/-0.25421526
	246	705	TTEVMPVSM	0.62/-0.045282362
	247	815	FMKQYGECL	0.03/0.54054092
	248	598	YQDVNCTDV	0.00/0.56479598
	249	1067	GKAYFPREG	0.93/-0.36672025
	250	154	DNAFNCTFE	0.98/-0.41906956
	251	887	RFNGIGVTQ	0.76/-0.2020951
	252	1115	INNTVYDPL	0.73/-0.17409328
	253	285	ELKCSVKSF	0.00/0.55424931
	254	1203	IAGLIAIVM	/0.5530776
	255	584	ITPGTNASS	0.99/-0.44178811
	256	573	ISPCSFGGV	0.74/-0.19370372
	257	412	LPDDFMGCV	0.11/0.43347787
	258	397	IAPGQTGVI	0.03/0.5118819
	259	522	NQCVNFNFN	0.52/0.021830229
	260	396	QIAPGQTGV	0.00/0.54060927
	261	657	CASYHTVSL	0.57/-0.03478087
	262	352	YSVLYNSTF	0.86/-0.32544177
	263	383	YADSFVVKG	0.08/0.4540279
	264	1000	IRASANLAA	0.60/-0.067413399
	265	1018	QSKRVDFCG	0.99/-0.45968019
	266	699	NFSISITTE	0.77/-0.24155566
	267	1095	QIITTDNTF	0.83/-0.30217935
	268	363	TFKCYGVSA	0.66/-0.13507416
	269	398	APGQTGVIA	0.02/0.50481136
	270	489	IGYQPYRVV	0.02/0.4999939
	271	176	SGNFKHLRE	0.94/-0.42435894
	272	1208	AIVMVTILL	/0.51235881
	273	357	NSTFFSTFK	0.05/0.46101123
	274	376	DLCFSNVYA	0.10/0.40826713
	275	495	RVVVLSFEL	0.13/0.37790287
	276	134	DNPFFAVSK	0.00/0.50568662

	277	523	QCVNFNFNG	0.88/-0.3771993
	**278**	**1191**	**YIKWPWYVW**	**/0.50188623**
	279	808	VTLADAGFM	0.79/-0.28951613
	280	1078	VFNGTSWFI	0.01/0.48775526
	281	1197	YVWLGFIAG	/0.49768537
	282	778	YFGGFNFSQ	0.82/-0.32690109
	283	172	VSEKSGNFK	0.80/-0.30778547
	284	704	ITTEVMPVS	0.86/-0.36799302
	285	1155	NASVVNIQK	0.00/0.48923419
	286	732	ANLLLQYGS	0.73/-0.24101658
	287	736	LQYGSFCTQ	0.71/-0.22195096
	288	505	NAPATVCGP	0.77/-0.28444478
	289	324	PFGEVFNAT	0.87/-0.38873589
	290	875	ALQIPFAMQ	0.99/-0.5129913
	291	1206	LIAIVMVTI	/0.47352128
	292	92	TEKSNVVRG	0.02/0.44950188
	293	849	DDMIAAYTA	0.54/-0.071829492
	294	32	QHTSSMRGV	0.86/-0.39185733
	295	214	NTLKPIFKL	0.05/0.4134994
	296	430	ATSTGNYNY	0.00/0.46341048
	297	1207	IAIVMVTIL	/0.45549214
	**298**	**901**	**NQKQIANQF**	**0.05/0.40289272**
	299	860	VSGTATAGW	0.88/-0.43015748
	300	13	GSDLDRCTT	0.95/-0.50143335
	301	852	IAAYTAALV	0.60/-0.16000632
	302	302	TSNFRVVPS	0.59/-0.15349161
	303	895	QNVLYENQK	0.64/-0.20458317
	304	820	GECLGDINA	0.00/0.42754106
	305	497	VVLSFELLN	0.76/-0.33640163
	306	334	FPSVYAWER	0.02/0.40036043
	307	936	QNAQALNTL	0.01/0.40665469
	308	520	IKNQCVNFN	0.81/-0.39497434
	309	626	NNVFQTQAG	0.97/-0.55874967
	310	455	ISNVPFSPD	0.99/-0.57920428
	311	152	IFDNAFNCT	0.65/-0.24184003
	312	676	AYTMSLGAD	0.96/-0.55566783
	313	846	LLTDDMIAA	0.54/-0.14057689
	314	649	DIPIGAGIC	0.80/-0.405829
	315	299	IYQTSNFRV	0.00/0.39102148

	316	1032	MSFPQAAPH	0.83/-0.43993695
	**317**	**1201**	**GFIAGLIAI**	**/0.38830748**
	318	673	SIVAYTMSL	0.01/0.37599299
	319	1246	VLKGVKLHY	/0.38501663
	320	200	YQPIDVVRD	0.92/-0.54851618
	321	212	GFNTLKPIF	0.59/-0.22225368
	322	15	DLDRCTTFD	0.99/-0.62317732
	323	957	SVLNDILSR	0.51/-0.14367289
	324	838	NGLTVLPPL	0.73/-0.36548633
	325	1019	SKRVDFCGK	0.56/-0.19627116
	326	178	NFKHLREFV	0.00/0.36348113
	327	251	AYFVGYLKP	0.79/-0.43021312
	328	979	ITGRLQSLQ	0.60/-0.24138811
	329	446	GKLRPFERD	0.97/-0.62008704
	330	1096	IITTDNTFV	0.58/-0.23155762
	331	892	GVTQNVLYE	0.66/-0.31273301
	332	183	REFVFKNKD	0.97/-0.62576758
	333	163	YISDAFSLD	0.69/-0.34672464
	334	803	LLFNKVTLA	0.64/-0.29799872
	335	601	VNCTDVSTA	0.85/-0.50938353
	336	653	GAGICASYH	0.64/-0.30590714
	337	781	GFNFSQILP	0.78/-0.45454639
	338	193	FLYVYKGYQ	0.97/-0.64488319
	339	343	KKISNCVAD	0.81/-0.48534288
	340	186	VFKNKDGFL	0.74/-0.42051083
	341	215	TLKPIFKLP	0.87/-0.5524945
	342	3	IFLLFLTLT	0.99/-0.6749784
	343	257	LKPTTFMLK	0.52/-0.21193809
	344	587	GTNASSEVA	0.89/-0.58466495
	345	59	FLPFYSNVT	0.56/-0.25567868
	346	962	ILSRLDKVE	0.98/-0.67803869
	347	662	TVSLLRSTS	0.61/-0.31640394
	348	123	VVIRACNFE	0.92/-0.62889917
	349	1113	GIINNTVYD	0.79/-0.50753657
	350	731	CANLLLQYG	0.63/-0.35212059
	351	812	DAGFMKQYG	0.91/-0.63706258
	352	721	NMYICGDST	0.94/-0.66739372
	353	976	DRLITGRLQ	0.95/-0.68422966
	354	270	GTITDAVDC	0.90/-0.63452242

	355	652	IGAGICASY	0.96/-0.69611784
	356	1182	LQELGKYEQ	0.91/-0.64746161
	357	881	AMQMAYRFN	0.89/-0.62842562
	358	1074	EGVFVFNGT	0.81/-0.55295901
	359	712	SMAKTSVDC	0.97/-0.73165598
	360	750	SGIAAEQDR	0.97/-0.73414792
	361	641	HVDTSYECD	0.79/-0.55453632
	362	470	PALNCYWPL	0.98/-0.74879714
	363	772	KTPTLKYFG	0.87/-0.63912731
	364	169	SLDVSEKSG	0.80/-0.56984781
	365	1048	TYVPSQERN	0.60/-0.37130228
	366	140	VSKPMGTQT	0.59/-0.36264949
	367	844	PPLLTDDMI	0.93/-0.7035536
	368	968	KVEAEVQID	0.72/-0.52449453
	369	85	DGIYFAATE	0.81/-0.61792411
	370	1011	MSECVLGQS	0.95/-0.75851111
	371	480	DYGFYTTTG	0.65/-0.45976869
	372	571	LDISPCSFG	0.90/-0.71313511
	373	605	DVSTAIHAD	0.73/-0.55196068
	374	61	PFYSNVTGF	0.58/-0.40289454
	375	428	IDATSTGNY	0.73/-0.55787431
	376	878	IPFAMQMAY	0.83/-0.66172733
	377	137	FFAVSKPMG	0.98/-0.8140195
	378	856	TAALVSGTA	0.62/-0.4559563
	379	542	SKRFQPFQQ	0.77/-0.60706626
	380	196	VYKGYQPID	0.87/-0.71193255
	381	1064	CHEGKAYFP	0.98/-0.82245574
	382	394	VRQIAPGQT	0.97/-0.83689108
	383	675	VAYTMSLGA	0.70/-0.57061653
	384	546	QPFQQFGRD	0.97/-0.84322976
	385	456	SNVPFSPDG	0.69/-0.56400599
	386	709	MPVSMAKTS	0.93/-0.81254283
	387	752	IAAEQDRNT	0.63/-0.51852776
	388	633	AGCLIGAEH	0.63/-0.52021926
	389	775	TLKYFGGFN	0.80/-0.70419167
	390	816	MKQYGECLG	0.91/-0.81836769
	391	88	YFAATEKSN	0.86/-0.77045812
	392	191	DGFLYVYKG	0.54/-0.45379858
	393	166	DAFSLDVSE	0.98/-0.89696565

	394	1161	IQKEIDRLN	0.73/-0.6602502
	395	654	AGICASYHT	0.78/-0.72217748
	396	114	VIIINNSTN	0.75/-0.69732738
	397	931	QDVVNQNAQ	0.68/-0.62903621
	398	264	LKYDENGTI	0.94/-0.89676427
	399	585	TPGTNASSE	0.79/-0.74784998
	400	661	HTVSLLRST	0.73/-0.69219799
	401	317	PNITNLCPF	0.75/-0.71932082
	402	484	YTTTGIGYQ	0.93/-0.90376755
	403	408	YNYKLPDDF	0.85/-0.83580025
	404	143	PMGTQTHTM	0.99/-0.99044343
	405	630	QTQAGCLIG	0.76/-0.76196468
	406	204	DVVRDLPSG	0.85/-0.85876101
	407	23	DDVQAPNYT	0.78/-0.78924934
	408	271	TITDAVDCS	0.64/-0.65928597
	409	783	NFSQILPDP	0.95/-0.97250767
	410	855	YTAALVSGT	0.54/-0.56296968
	411	643	DTSYECDIP	0.95/-0.97409968
	412	385	DSFVVKGDD	0.97/-1.0069969
	413	5	LLFLTLTSG	0.63/-0.67296897
	414	720	CNMYICGDS	0.83/-0.88885802
	415	26	QAPNYTQHT	0.74/-0.80155471
	416	24	DVQAPNYTQ	0.60/-0.68396787
	417	135	NPFFAVSKP	0.60/-0.6988653
	418	532	LTGTGVLTP	0.65/-0.77063935
	419	392	DDVRQIAPG	0.62/-0.75249678
	420	339	AWERKKISN	0.93/-1.0690283
	421	69	FHTINHTFG	0.67/-0.81235071
	422	948	LSSNFGAIS	0.66/-0.81528605
	423	549	QQFGRDVSD	0.93/-1.0948516
	424	680	SLGADSSIA	0.96/-1.1350953
	425	786	QILPDPLKP	0.73/-0.90576926
	426	527	FNFNGLTGT	0.91/-1.0862641
	427	1094	PQIITTDNT	0.59/-0.79950416
	428	813	AGFMKQYGE	0.64/-0.85601039
	429	485	TTTGIGYQP	0.73/-0.97038176
	430	128	CNFELCDNP	0.68/-0.92957098
	431	1051	PSQERNFTT	0.56/-0.81950656
	432	564	DPKTSEILD	0.55/-0.8202666

	433	893	VTQNVLYEN	0.66/-0.93287129
	434	1045	LHVTYVPSQ	0.99/-1.2654502
	435	1140	NHTSPDVDL	0.55/-0.84283319
	436	87	IYFAATEKS	0.54/-0.84065819
	437	1173	KNLNESLID	0.77/-1.0715629
	438	290	VKSFEIDKG	0.76/-1.0690506
	439	103	FGSTMNNKS	0.96/-1.2927739
	440	327	EVFNATKFP	0.66/-0.99992216
	441	259	PTTFMLKYD	0.90/-1.241965
	442	782	FNFSQILPD	0.62/-0.9620474
	443	273	TDAVDCSQN	0.60/-0.9602398
	444	788	LPDPLKPTK	0.66/-1.0655041
	445	1004	ANLAATKMS	0.87/-1.288379
	446	606	VSTAIHADQ	0.79/-1.2439519
	447	9	TLTSGSDLD	0.52/-0.97728736
	448	170	LDVSEKSGN	0.85/-1.3174187
	449	318	NITNLCPFG	0.94/-1.4393988
	450	945	VKQLSSNFG	0.93/-1.4346284
	451	203	IDVVRDLPS	0.96/-1.4851088
	452	184	EFVFKNKDG	0.68/-1.2268172
	453	599	QDVNCTDVS	0.97/-1.5251249
	454	458	VPFSPDGKP	0.94/-1.4954966
	455	1028	GYHLMSFPQ	0.61/-1.2157184
	456	7	FLTLTSGSD	0.87/-1.481056
	457	580	GVSVITPGT	0.69/-1.3218354
	458	1122	PLQPELDSF	0.55/-1.1856682
	459	6	LFLTLTSGS	0.94/-1.5786607
	460	1116	NNTVYDPLQ	0.85/-1.492597
	461	513	PKLSTDLIK	0.60/-1.2689348
	462	636	LIGAEHVDT	0.86/-1.5903752
	463	916	IQESLTTTS	0.80/-1.5617904
	464	367	YGVSATKLN	0.96/-1.7341912
	465	663	VSLLRSTSQ	0.53/-1.364465
	466	966	LDKVEAEVQ	0.67/-1.5533956
	467	767	VKQMYKTPT	0.96/-2.0682375
	468	1120	YDPLQPELD	0.59/-1.7567044
	469	28	PNYTQHTSS	0.66/-2.1108841
**MERS-CoV**				

Viral protein name	Rank	Start position	Sequence	Score ANN/SVM
**Spike**	1	751	RSVPGEMRL	0.95/1.0687206
	2	336	AIDCGFNDL	0.98/0.90784894
	3	1	MIHSVFLLM	0.97/0.91473499
	4	1032	ALSKLASEL	0.92/0.95686386
	5	1278	ESYIDLKEL	0.91/0.94986528
	6	630	FVYDAYQNL	0.84/0.98684097
	7	498	SYINKCSRL	0.47/1.3067989
	8	270	YVDLYGGNM	0.95/0.79768027
	9	112	FANGFVVRI	0.45/1.2939448
	10	1244	TSIPNFGSL	0.92/0.77792369
	11	845	SVRNLFASV	0.97/0.70106372
	12	772	QLNSSYFKL	0.97/0.67687168
	13	823	EYGQFCSKI	0.93/0.69441063
	14	473	FSNPTCLIL	0.87/0.74637331
	15	307	RKAWAAFYV	0.84/0.7381001
	16	292	YYSIIPHSI	0.79/0.77662825
	17	282	ATLPVYDTI	0.07/1.4867292
	18	1065	AQIDRLING	0.77/0.72729886
	19	1070	LINGRLTTL	0.72/0.7584431
	20	1074	RLTTLNAFV	0.20/1.2783667
	21	1201	NTKYVAPQV	0.91/0.55793045
	22	97	PQKLFVANY	0.70/0.7543466
	23	1254	QINTTLLDL	0.81/0.63927539
	**24**	**975**	**SIFYRLNGV**	**0.98/0.45706474**
	25	1166	APVNGYFIK	0.99/0.43439606
	**26**	**1025**	**AVNNNAQAL**	**0.33/1.0919338**
	27	595	KIASQLGNC	0.94/0.48078322
	28	317	KLQPLTFLL	0.21/1.1941265
	29	434	ASNCYSSLI	0.79/0.61355361
	30	1197	ITSLNTKYV	0.92/0.48237559
	31	31	IEVDIQQTF	0.96/0.44123869
	32	346	QLHCSYESF	0.98/0.41824276
	33	278	MFQFATLPV	0.94/0.45179384
	34	303	IQSDRKAWA	1.00/0.3750027
	35	226	NSFKEYFNL	0.55/0.81163751
	36	737	ALPDTPSTL	0.06/1.2937612
	**37**	**1128**	**VNAPNGLYF**	**0.60/0.74121655**

	38	1141	YYPSNHIEV	0.12/1.2183088
	**39**	**1275**	**ALNESYIDL**	**0.00/1.3214488**
	40	3	HSVFLLMFL	0.33/0.97938501
	41	242	TYNITEDEI	0.52/0.78788058
	42	462	GPISQFNYK	0.73/0.57779357
	43	624	GVRQQRFVY	0.96/0.33001584
	44	1073	GRLTTLNAF	0.82/0.46949067
	45	972	FAQSIFYRL	0.31/0.97721311
	46	1002	NQALGAMQT	0.86/0.4220723
	47	42	KTWPRPIDV	0.30/0.97309465
	48	419	SVNDFTCSQ	0.64/0.62272262
	49	1215	STNLPPPLL	0.72/0.53785544
	50	156	FSDGKMGRF	0.88/0.37258124
	51	703	TYGPLQTPV	0.73/0.51648442
	52	631	VYDAYQNLV	0.75/0.46216708
	53	787	SFGVTQEYI	0.83/0.36450572
	54	1258	TLLDLTYEM	0.13/1.0607423
	55	141	RKIYPAFML	0.93/0.26058818
	56	433	IASNCYSSL	0.99/0.18701988
	57	900	IADPGYMQG	0.90/0.27478176
	58	164	FFNHTLVLL	0.00/1.1687357
	59	221	RNASLNSFK	0.88/0.28872588
	60	881	TGSRSARSA	0.96/0.20003002
	61	94	GTTPQKLFV	0.85/0.30586214
	62	328	SVDGYIRRA	0.62/0.52897679
	63	576	QYGTDTNSV	0.99/0.15513332
	64	395	QVYNFKRLV	0.65/0.49190588
	65	25	SVKSACIEV	0.14/1.0016961
	66	826	QFCSKINQA	0.94/0.19846214
	67	641	YYSDDGNYY	0.00/1.1359411
	68	918	ASARDLICA	0.77/0.36455438
	69	1049	ASIGDIIQR	0.92/0.21269236
	70	699	RRDSTYGPL	0.86/0.26840132
	71	730	PLGQSLCAL	0.82/0.30213022
	72	841	RQDDSVRNL	0.06/1.0596324
	73	570	GFGITVQYG	0.70/0.41848594
	74	220	NRNASLNSF	0.38/0.73628472
	**75**	**920**	**ARDLICAQY**	**0.14/0.97234577**
	76	92	ATGTTPQKL	0.67/0.43882611

	77	655	VSVPVSVIY	0.50/0.6044505
	78	1000	KFNQALGAM	0.23/0.86456217
	79	1268	SLQQVVKAL	0.01/1.0736436
	80	266	FSSRYVDLY	0.67/0.40983872
	81	1309	ALALCVFFI	/1.0797517
	82	1050	SIGDIIQRL	0.72/0.35948984
	83	1116	FCGQGTHIV	0.85/0.22624257
	84	331	GYIRRAIDC	0.45/0.62562951
	85	136	TSATIRKIY	0.85/0.22399459
	86	997	IANKFNQAL	0.55/0.5224597
	87	710	PVGCVLGLV	0.78/0.29204099
	88	480	ILATVPHNL	0.32/0.7473596
	89	580	DTNSVCPKL	0.53/0.53162014
	90	78	QGDHGDMYV	0.64/0.41941369
	**91**	**892**	**DLLFDKVTI**	**0.56/0.49242704**
	92	933	KVLPPLMDV	0.69/0.35876353
	93	535	WEDGDYYRK	0.81/0.22653775
	94	627	QQRFVYDAY	0.84/0.19222925
	95	163	RFFNHTLVL	0.91/0.1208001
	96	404	FTNCNYNLT	0.98/0.046119296
	97	232	FNLRNCTFM	0.05/0.97291655
	98	11	LLTPTESYV	0.71/0.3081859
	99	263	VHLFSSRYV	0.90/0.10626798
	100	310	WAAFYVYKL	0.13/0.87561177
	101	698	KRRDSTYGP	0.97/0.031699774
	102	949	SSLLGSIAG	0.97/0.023813555
	**103**	**1291**	**YYNKWPWYI**	**/0.98943415**
	104	827	FCSKINQAL	0.82/0.1685718
	105	969	AIPFAQSIF	0.72/0.26687558
	106	262	GVHLFSSRY	0.43/0.55464322
	107	786	FSFGVTQEY	0.97/0.0096269755
	108	968	AAIPFAQSI	0.10/0.8750469
	109	483	TVPHNLTTI	0.22/0.75473192
	110	866	GGDFNLTLL	0.31/0.66065832
	111	108	DVKQFANGF	0.00/0.96649715
	112	459	SSAGPISQF	0.65/0.31636956
	113	444	DYFSYPLSM	0.18/0.78615375
	114	1122	HIVSFVVNA	0.15/0.81293353
	115	50	VSKADGIIY	0.03/0.9329199

	116	1163	NCIAPVNGY	0.73/0.23244441
	117	453	KSDLSVSSA	0.84/0.12022072
	118	522	QYSPCVSIV	0.01/0.94906953
	119	1047	ISASIGDII	0.99/-0.036092964
	120	179	LLRAFYCIL	0.01/0.94087367
	121	1185	SYTGSSFYA	0.87/0.080057043
	122	391	GTPPQVYNF	0.05/0.8975718
	123	59	PQGRTYSNI	0.91/0.035446458
	124	1171	YFIKTNNTR	0.95/-0.0050865908
	125	887	RSAIEDLLF	0.90/0.04378359
	126	355	DVESGVYSV	0.03/0.90428769
	127	716	GLVNSSLFV	0.52/0.40571098
	128	1230	DFQDELDEF	0.98/-0.059229733
	129	1284	KELGNYTYY	/0.92016098
	130	526	CVSIVPSTV	0.00/0.91874491
	131	213	DCSDGNYNR	0.57/0.34873258
	132	1179	RIVDEWSYT	0.76/0.15226026
	133	1234	ELDEFFKNV	0.02/0.8921197
	134	752	SVPGEMRLA	0.97/-0.063279431
	135	674	FGSVACEHI	0.98/-0.081708659
	136	982	GVGITQQVL	0.39/0.50294203
	137	1332	KCNRCCDRY	/0.89207171
	138	304	QSDRKAWAA	0.50/0.38817415
	139	813	GFQKCEQLL	0.56/0.32803925
	140	4	SVFLLMFLL	0.01/0.87744856
	**141**	**1115**	**GFCGQGTHI**	**0.97/-0.086697044**
	**142**	**1302**	**GFIAGLVAL**	**/0.88200538**
	143	857	QSSPIIPGF	0.98/-0.10086419
	144	178	TLLRAFYCI	0.01/0.86649455
	145	1012	FTTTNEAFR	0.85/0.02424523
	146	582	NSVCPKLEF	0.65/0.22269833
	147	1241	NVSTSIPNF	0.47/0.40157321
	148	548	LEGGGWLVA	0.37/0.49860677
	149	1035	KLASELSNT	0.96/-0.092672325
	150	1120	GTHIVSFVV	0.70/0.16135788
	151	56	IIYPQGRTY	0.86/0.00072868393
	152	506	LLSDDRTEV	0.00/0.85876251

	153	617	FQNCTAVGV	0.32/0.53059444
	**154**	**1134**	**LYFMHVGYY**	**0.99/-0.14427586**
	155	1126	FVVNAPNGL	0.90/-0.06198942
	156	113	ANGFVVRIG	0.02/0.81652064
	157	114	NGFVVRIGA	0.62/0.21548528
	158	534	VWEDGDYYR	0.77/0.061463248
	159	926	AQYVAGYKV	0.68/0.14881084
	160	648	YYCLRACVS	0.99/-0.16373158
	161	276	GNMFQFATL	0.17/0.65487679
	162	18	YVDVGPDSV	0.51/0.31472512
	163	540	YYRKQLSPL	0.45/0.36831902
	164	1038	SELSNTFGA	0.44/0.3777823
	165	499	YINKCSRLL	0.83/-0.012687027
	166	313	FYVYKLQPL	0.08/0.73686339
	167	686	MSQYSRSTR	0.37/0.44470934
	168	1165	IAPVNGYFI	0.01/0.80325124
	169	49	DVSKADGII	0.83/-0.020093876
	170	825	GQFCSKINQ	0.99/-0.18087676
	171	563	MTEQLQMGF	0.91/-0.10586779
	172	653	ACVSVPVSV	0.34/0.45748738
	**173**	**977**	**FYRLNGVGI**	**0.00/0.79279512**
	174	296	IPHSIRSIQ	0.90/-0.10977856
	175	1147	IEVVSAYGL	0.05/0.73879841
	176	690	SRSTRSMLK	0.51/0.27723927
	177	1053	DIIQRLDVL	0.10/0.6839985
	178	229	KEYFNLRNC	0.03/0.75375487
	179	954	SIAGVGWTA	0.67/0.10986263
	180	1227	TGIDFQDEL	0.07/0.70964031
	181	692	STRSMLKRR	0.80/-0.021814712
	182	1014	TTNEAFRKV	0.00/0.77732566
	183	671	ATLFGSVAC	0.17/0.59396847
	184	1236	DEFFKNVST	0.99/-0.22718534
	185	327	FSVDGYIRR	0.00/0.76216554
	186	491	ITKPLKYSY	0.52/0.24070756
	187	1112	KRSGFCGQG	0.92/-0.1603229
	188	442	ILDYFSYPL	0.88/-0.12223742
	189	366	FEAKPSGSV	0.67/0.074974423
	190	176	CGTLLRAFY	0.95/-0.2078755
	191	231	YFNLRNCTF	0.12/0.62145635

	192	295	IIPHSIRSI	0.53/0.21101408
	193	706	PLQTPVGCV	1.00/-0.26141429
	194	392	TPPQVYNFK	0.03/0.70677111
	195	890	IEDLLFDKV	0.84/-0.10481566
	196	1310	LALCVFFIL	/0.73482458
	197	567	LQMGFGITV	0.71/0.02301018
	198	1206	APQVTYQNI	0.03/0.70174242
	199	1160	NPTNCIAPV	0.71/0.020766975
	200	893	LLFDKVTIA	0.35/0.37940735
	201	721	SLFVEDCKL	0.05/0.67843327
	202	882	GSRSARSAI	0.29/0.43802917
	203	626	RQQRFVYDA	0.09/0.63256432
	204	970	IPFAQSIFY	0.79/-0.068162709
	205	1247	PNFGSLTQI	0.88/-0.16010176
	206	922	DLICAQYVA	0.97/-0.27332673
	**207**	**1130**	**APNGLYFMH**	**0.30/0.39359148**
	208	299	SIRSIQSDR	0.82/-0.12991588
	209	1293	NKWPWYIWL	/0.68175091
	210	600	LGNCVEYSL	0.66/0.019552802
	211	668	KTHATLFGS	0.88/-0.20124114
	212	921	RDLICAQYV	0.01/0.66830014
	213	1344	DLEPHKVHV	/0.67725876
	214	647	NYYCLRACV	0.29/0.38221191
	**215**	**993**	**NQKLIANKF**	**0.02/0.65017867**
	216	245	ITEDEILEW	0.58/0.082462128
	217	553	WLVASGSTV	0.01/0.64985351
	218	1145	NHIEVVSAY	0.13/0.52891408
	219	1158	AANPTNCIA	0.02/0.63833265
	220	642	YSDDGNYYC	0.18/0.47774429
	221	1249	FGSLTQINT	0.80/-0.14549239
	222	844	DSVRNLFAS	0.60/0.05024281
	223	766	HPIQVDQLN	0.96/-0.31041729
	224	782	IPTNFSFGV	0.65/-0.0019764399
	225	177	GTLLRAFYC	0.02/0.6217205
	226	305	SDRKAWAAF	0.75/-0.112341
	227	38	TFFDKTWPR	0.05/0.58761966
	228	1016	NEAFRKVQD	0.83/-0.19275131
	229	562	AMTEQLQMG	0.80/-0.16549054

	230	720	SSLFVEDCK	0.55/0.083770077
	231	678	ACEHISSTM	0.09/0.54096983
	232	865	FGGDFNLTL	0.72/-0.092457905
	233	915	QGPASARDL	0.72/-0.092768774
	234	294	SIIPHSIRS	0.57/0.057063443
	235	77	YQGDHGDMY	0.84/-0.21452308
	236	85	YVYSAGHAT	0.98/-0.3570909
	237	57	IYPQGRTYS	0.89/-0.26710895
	238	780	LSIPTNFSF	0.64/-0.017340059
	239	801	KVTVDCKQY	0.98/-0.35759757
	240	861	IIPGFGGDF	0.56/0.061012449
	241	533	TVWEDGDYY	0.66/-0.045226686
	**242**	**1292**	**YNKWPWYIW**	**/0.60806956**
	243	985	ITQQVLSEN	0.97/-0.36321819
	244	140	IRKIYPAFM	0.04/0.5665451
	245	556	ASGSTVAMT	0.17/0.4348019
	246	615	GVFQNCTAV	0.04/0.56373656
	247	962	AGLSSFAAI	0.08/0.51323156
	248	253	WFGITQTAQ	0.85/-0.25945579
	249	513	EVPQLVNAN	0.51/0.072816261
	250	846	VRNLFASVK	0.09/0.49084835
	251	1046	AISASIGDI	0.04/0.53748172
	252	1078	LNAFVAQQL	0.70/-0.12423014
	253	234	LRNCTFMYT	0.00/0.57528912
	254	115	GFVVRIGAA	0.91/-0.33511276
	255	707	LQTPVGCVL	0.75/-0.17520072
	256	1266	MLSLQQVVK	0.08/0.49193623
	257	1152	AYGLCDAAN	0.54/0.025994238
	258	1335	RCCDRYEEY	/0.56468276
	259	1132	NGLYFMHVG	0.91/-0.34904069
	260	643	SDDGNYYCL	0.02/0.53931673
	261	863	PGFGGDFNL	0.82/-0.26686725
	262	1246	IPNFGSLTQ	0.87/-0.32157967
	263	309	AWAAFYVYK	0.00/0.54566796
	264	246	TEDEILEWF	0.05/0.4922968
	265	376	EQAEGVECD	0.97/-0.42865706
	266	676	SVACEHISS	0.91/-0.37144384
	267	1303	FIAGLVALA	/0.53558793
	268	1216	TNLPPPLLG	0.87/-0.33564407

	269	1125	SFVVNAPNG	0.85/-0.31576137
	270	70	TYQGLFPYQ	0.64/-0.10616507
	271	330	DGYIRRAID	0.99/-0.45820288
	272	105	YSQDVKQFA	0.58/-0.052124143
	273	10	FLLTPTESY	0.99/-0.46327628
	274	396	VYNFKRLVF	0.13/0.39443369
	275	878	SISTGSRSA	0.93/-0.40687571
	276	406	NCNYNLTKL	0.07/0.45311362
	277	390	SGTPPQVYN	0.99/-0.46850976
	278	621	TAVGVRQQR	0.02/0.4994245
	279	271	VDLYGGNMF	0.86/-0.34166406
	280	1164	CIAPVNGYF	0.00/0.51460498
	281	1069	RLINGRLTT	0.68/-0.16814007
	282	1193	APEPITSLN	0.63/-0.12071683
	283	794	YIQTTIQKV	0.63/-0.1275968
	284	258	QTAQGVHLF	0.02/0.48126671
	285	614	RGVFQNCTA	0.96/-0.45959761
	286	1024	DAVNNNAQA	0.66/-0.16084347
	287	913	MQQGPASAR	0.55/-0.056978444
	288	1154	GLCDAANPT	0.93/-0.43989785
	289	135	STSATIRKI	0.01/0.47961503
	290	622	AVGVRQQRF	0.01/0.47770348
	291	708	QTPVGCVLG	0.98/-0.49560177
	292	822	REYGQFCSK	0.05/0.43280774
	293	122	AAANSTGTV	0.73/-0.26108703
	294	1342	EYDLEPHKV	/0.46745524
	295	663	YDKETKTHA	0.98/-0.51502941
	296	1204	YVAPQVTYQ	0.00/0.46243144
	297	300	IRSIQSDRK	0.03/0.4298579
	298	660	SVIYDKETK	0.67/-0.21073164
	299	1311	ALCVFFILC	/0.45721592
	300	943	MEAAYTSSL	0.01/0.44420198
	301	749	SVRSVPGEM	0.01/0.44105273
	302	1297	WYIWLGFIA	/0.44831422
	303	217	GNYNRNASL	0.01/0.43741602
	304	197	AGNSYTSFA	0.74/-0.29348105
	305	953	GSIAGVGWT	0.86/-0.41798702
	306	998	ANKFNQALG	0.03/0.41171683
	307	95	TTPQKLFVA	0.99/-0.55128102

	308	955	IAGVGWTAG	0.58/-0.14139627
	309	565	EQLQMGFGI	0.89/-0.45194789
	310	509	DDRTEVPQL	0.51/-0.07652848
	311	802	VTVDCKQYV	0.01/0.42310575
	312	628	QRFVYDAYQ	0.82/-0.38776991
	313	764	FNHPIQVDQ	0.93/-0.50039409
	314	161	MGRFFNHTL	0.00/0.42042943
	315	665	KETKTHATL	0.04/0.37890249
	316	501	NKCSRLLSD	0.97/-0.55271012
	317	22	GPDSVKSAC	0.61/-0.19345945
	318	512	TEVPQLVNA	0.03/0.38606041
	319	1007	AMQTGFTTT	0.90/-0.48650257
	320	1245	SIPNFGSLT	0.72/-0.30830238
	321	1041	SNTFGAISA	0.05/0.36159342
	322	65	SNITITYQG	0.87/-0.46133046
	323	555	VASGSTVAM	0.00/0.40780761
	324	1330	KLKCNRCCD	/0.4029762
	325	224	SLNSFKEYF	0.00/0.3972556
	326	432	AIASNCYSS	0.55/-0.15764841
	327	39	FFDKTWPRP	0.96/-0.56834753
	328	416	SLFSVNDFT	0.66/-0.26923111
	329	1129	NAPNGLYFM	0.00/0.3903336
	330	645	DGNYYCLRA	0.58/-0.19163244
	331	1173	IKTNNTRIV	0.01/0.37834217
	332	964	LSSFAAIPF	0.83/-0.44408461
	333	935	LPPLMDVNM	0.64/-0.25524073
	334	1251	SLTQINTTL	0.00/0.38367782
	335	1059	DVLEQDAQI	0.00/0.38097157
	336	696	MLKRRDSTY	0.00/0.38077499
	337	649	YCLRACVSV	0.01/0.37036168
	338	495	LKYSYINKC	0.54/-0.1608164
	339	485	PHNLTTITK	0.75/-0.37712782
	340	188	EPRSGNHCP	0.92/-0.54962202
	341	872	TLLEPVSIS	0.77/-0.41449786
	342	1159	ANPTNCIAP	0.55/-0.19517041
	343	1044	FGAISASIG	0.94/-0.60011359
	344	165	FNHTLVLLP	0.93/-0.59351837
	345	461	AGPISQFNY	0.53/-0.200378
	346	465	SQFNYKQSF	0.90/-0.57048416

	347	239	FMYTYNITE	0.67/-0.34847386
	348	502	KCSRLLSDD	0.95/-0.63288187
	349	334	RRAIDCGFN	0.91/-0.60643944
	350	959	GWTAGLSSF	0.77/-0.46872835
	351	603	CVEYSLYGV	0.72/-0.42265218
	352	52	KADGIIYPQ	0.71/-0.41343896
	353	662	IYDKETKTH	0.96/-0.66858006
	354	564	TEQLQMGFG	0.79/-0.50194902
	355	588	LEFANDTKI	0.94/-0.65250566
	356	399	FKRLVFTNC	0.62/-0.33996511
	357	1022	VQDAVNNNA	0.56/-0.28132524
	358	858	SSPIIPGFG	1.00/-0.72965374
	359	518	VNANQYSPC	0.99/-0.71992597
	360	869	FNLTLLEPV	0.91/-0.64120938
	361	289	TIKYYSIIP	0.71/-0.44173822
	362	1086	LVRSESAAL	0.55/-0.28200055
	363	1089	SESAALSAQ	0.68/-0.42090549
	364	904	GYMQGYDDC	0.64/-0.38370308
	365	1198	TSLNTKYVA	0.61/-0.35433589
	366	974	QSIFYRLNG	0.53/-0.27617988
	367	507	LSDDRTEVP	0.98/-0.72719503
	368	1168	VNGYFIKTN	0.62/-0.36813429
	369	439	SSLILDYFS	0.97/-0.72620275
	370	281	FATLPVYDT	0.78/-0.54298364
	371	209	TPATDCSDG	0.96/-0.728034
	372	1279	SYIDLKELG	0.77/-0.53928964
	373	940	DVNMEAAYT	0.54/-0.3102788
	374	279	FQFATLPVY	0.94/-0.7221058
	375	372	GSVVEQAEG	0.95/-0.73672015
	376	597	ASQLGNCVE	0.81/-0.59946363
	377	1256	NTTLLDLTY	0.99/-0.78138739
	378	40	FDKTWPRPI	0.58/-0.37239834
	379	369	KPSGSVVEQ	0.68/-0.50061853
	380	208	HTPATDCSD	0.94/-0.76122135
	381	742	PSTLTPRSV	0.96/-0.78323864
	382	436	NCYSSLILD	0.94/-0.77100125
	383	496	KYSYINKCS	0.80/-0.65796787
	384	45	PRPIDVSKA	0.83/-0.6902416
	385	186	ILEPRSGNH	0.52/-0.38503946

	386	670	HATLFGSVA	0.56/-0.43188367
	387	746	TPRSVRSVP	0.60/-0.47314795
	388	650	CLRACVSVP	0.73/-0.60447888
	389	1243	STSIPNFGS	0.85/-0.72722733
	390	130	VIISPSTSA	0.69/-0.57102461
	391	72	QGLFPYQGD	0.82/-0.71369352
	392	592	NDTKIASQL	0.66/-0.55518204
	393	5	VFLLMFLLT	0.96/-0.85793243
	394	260	AQGVHLFSS	0.84/-0.74222638
	395	348	HCSYESFDV	0.72/-0.63106176
	396	249	EILEWFGIT	0.93/-0.85377882
	397	17	SYVDVGPDS	0.70/-0.63134303
	398	322	TFLLDFSVD	0.84/-0.77261535
	399	1023	QDAVNNNAQ	0.94/-0.87554894
	400	1153	YGLCDAANP	0.98/-0.91728324
	401	575	VQYGTDTNS	0.73/-0.66954552
	402	873	LLEPVSIST	0.92/-0.86139275
	403	256	ITQTAQGVH	0.97/-0.91150271
	404	1087	VRSESAALS	0.71/-0.65321967
	405	353	SFDVESGVY	0.66/-0.61260149
	406	1257	TTLLDLTYE	0.81/-0.76817195
	407	897	KVTIADPGY	0.53/-0.4890011
	408	1133	GLYFMHVGY	0.64/-0.60141762
	409	447	SYPLSMKSD	0.68/-0.64937752
	410	1013	TTTNEAFRK	0.55/-0.51966562
	411	1202	TKYVAPQVT	0.90/-0.86994577
	412	365	SFEAKPSGS	0.83/-0.81594474
	413	103	ANYSQDVKQ	0.76/-0.75077778
	414	190	RSGNHCPAG	0.66/-0.65395516
	415	1143	PSNHIEVVS	0.95/-0.94428207
	416	212	TDCSDGNYN	0.94/-0.93562508
	417	134	PSTSATIRK	0.80/-0.80034738
	418	1169	NGYFIKTNN	1.00/-1.0070648
	419	290	IKYYSIIPH	0.55/-0.560356
	420	727	CKLPLGQSL	0.51/-0.52707663
	421	520	ANQYSPCVS	0.95/-0.97060464
	422	928	YVAGYKVLP	0.72/-0.76315835
	423	820	LLREYGQFC	0.78/-0.83582724
	424	55	GIIYPQGRT	0.95/-1.0115031

	425	244	NITEDEILE	0.91/-0.97500076
	426	952	LGSIAGVGW	0.76/-0.82524351
	427	1228	GIDFQDELD	0.97/-1.0387297
	428	992	ENQKLIANK	0.72/-0.79125318
	429	849	LFASVKSSQ	0.58/-0.65550029
	430	741	TPSTLTPRS	0.86/-0.93821969
	431	695	SMLKRRDST	0.74/-0.83179842
	432	1055	IQRLDVLEQ	0.68/-0.77302756
	433	1226	STGIDFQDE	0.72/-0.82938184
	434	312	AFYVYKLQP	0.88/-0.98950297
	435	789	GVTQEYIQT	0.53/-0.64904728
	436	729	LPLGQSLCA	0.73/-0.85747062
	437	1177	NTRIVDEWS	0.61/-0.75893742
	438	1103	VNECVKAQS	0.74/-0.90425647
	439	109	VKQFANGFV	0.51/-0.6836031
	440	864	GFGGDFNLT	0.55/-0.72887075
	441	1232	QDELDEFFK	0.72/-0.90314068
	442	652	RACVSVPVS	0.58/-0.76755349
	443	571	FGITVQYGT	0.60/-0.80617775
	444	474	SNPTCLILA	0.52/-0.73311341
	445	76	PYQGDHGDM	0.87/-1.0831585
	446	452	MKSDLSVSS	0.95/-1.1763039
	447	538	GDYYRKQLS	0.58/-0.80837331
	448	1220	PPLLGNSTG	0.76/-0.99012338
	449	1031	QALSKLASE	0.53/-0.76319456
	450	1271	QVVKALNES	0.82/-1.0623915
	451	604	VEYSLYGVS	0.53/-0.77657278
	452	14	PTESYVDVG	0.59/-0.85025294
	453	74	LFPYQGDHG	0.55/-0.81621351
	454	1253	TQINTTLLD	0.56/-0.83021605
	455	166	NHTLVLLPD	0.87/-1.1479338
	456	835	LHGANLRQD	0.88/-1.1623996
	457	517	LVNANQYSP	0.55/-0.8345149
	458	144	YPAFMLGSS	0.54/-0.83135736
	459	566	QLQMGFGIT	0.65/-0.9534416
	460	810	VCNGFQKCE	0.52/-0.83107607
	461	228	FKEYFNLRN	0.72/-1.0312241
	462	73	GLFPYQGDH	0.71/-1.024888
	463	875	EPVSISTGS	0.82/-1.1351898

	464	932	YKVLPPLMD	0.86/-1.1806539
	465	733	QSLCALPDT	0.74/-1.0695994
	466	147	FMLGSSVGN	0.70/-1.0410759
	467	664	DKETKTHAT	0.93/-1.2779392
	468	797	TTIQKVTVD	0.95/-1.3074728
	469	379	EGVECDFSP	0.69/-1.0566543
	470	1096	AQLAKDKVN	0.65/-1.0263296
	471	119	RIGAAANST	0.57/-0.9525637
	472	88	SAGHATGTT	0.78/-1.2030449
	473	463	PISQFNYKQ	0.62/-1.0638477
	474	545	LSPLEGGGW	0.86/-1.3108022
	475	1196	PITSLNTKY	0.98/-1.4437418
	476	455	DLSVSSAGP	0.91/-1.3920107
	477	374	VVEQAEGVE	0.94/-1.4272455
	478	195	CPAGNSYTS	0.85/-1.3433177
	479	909	YDDCMQQGP	0.69/-1.2028043
	480	490	TITKPLKYS	0.65/-1.1637635
	481	37	QTFFDKTWP	0.77/-1.2887773
	482	210	PATDCSDGN	0.80/-1.3294758
	483	658	PVSVIYDKE	0.70/-1.2543317
	484	895	FDKVTIADP	0.67/-1.2474127
	485	731	LGQSLCALP	0.80/-1.3968119
	486	120	IGAAANSTG	0.51/-1.1441705
	487	581	TNSVCPKLE	0.58/-1.2526687
	488	1033	LSKLASELS	0.82/-1.5518681
	489	725	EDCKLPLGQ	0.94/-1.7100772
	490	836	HGANLRQDD	0.63/-1.41272
	491	363	VSSFEAKPS	0.71/-1.51183
	492	638	LVGYYSDDG	0.74/-1.6071945
	493	573	ITVQYGTDT	0.61/-1.567401
	494	735	LCALPDTPS	0.78/-1.8348734
	495	739	PDTPSTLTP	0.85/-1.9485422
**HCoV-NL63**				
**Viral protein name**	**Rank**	**Start position**	**Sequence**	**Score ANN/SVM**
**Spike**	1	647	SLAGGITYV	0.50/1.7485472
	2	1121	SAPDGLLFL	0.71/1.4701125
	3	1220	TLQEFAQNL	0.78/1.270963
	4	1165	YSDNGVFRV	0.98/1.0340139

	5	1024	ALNHLTSQL	0.99/0.96059106
	6	1154	GYVLRQPNL	0.99/0.94512873
	7	1169	GVFRVTSRV	0.92/0.97829107
	8	590	YTIVGALYV	0.90/0.96558479
	9	178	NYSCVFSVV	0.55/1.3061226
	10	1175	SRVMFQPRL	0.90/0.94576564
	11	1066	RLAALNAFV	0.73/1.1011045
	12	372	TVREIVVAR	0.98/0.83200893
	13	1280	YVDLKLLNR	0.71/1.0833337
	14	953	ALQARLNYV	0.28/1.4734208
	15	1185	VLSDFVQIY	0.22/1.5251199
	16	522	FSIRYIYNR	0.97/0.74513173
	17	1001	AEAIHTVTI	0.76/0.93207468
	18	150	RLHLYNVTR	0.94/0.74561912
	19	785	IVVDCATYV	0.61/1.0353148
	20	1155	YVLRQPNLV	0.96/0.66182292
	21	1069	ALNAFVSQV	0.23/1.3344931
	22	565	TICFSTVAV	0.94/0.61771094
	23	588	TSYTIVGAL	0.92/0.62464212
	24	666	NVSTGNIFI	0.94/0.60457205
	25	825	ETNDVSSML	0.93/0.60718551
	26	89	IYVTNEIGL	0.16/1.357696
	27	709	LQLPNFYYV	0.02/1.4941587
	28	1076	QVLNKYTEV	0.76/0.73136175
	29	519	TSHFSIRYI	0.53/0.95241857
	30	491	ASFGGSCYV	0.96/0.51462509
	31	415	VAFATFVDV	0.68/0.78532103
	32	2	KLFLILLVL	0.09/1.3728741
	33	1230	KYVKPNFDL	0.58/0.8803956
	34	988	ASFSSVNDA	0.79/0.64883649
	35	196	THNGRVVNY	0.88/0.5477679
	36	20	NSNANLSML	0.28/1.1461754
	37	501	KPHQVNISL	0.01/1.414479
	38	399	AVNFNVTTA	0.00/1.4215403
	39	345	SSQPYYCFI	0.94/0.48121853
	40	878	FSKVVTSGL	0.91/0.50924148
	41	212	GYTDNIFSV	0.85/0.56238908
	42	174	KCYFNYSCV	0.71/0.67844509
	43	872	ALEDLLFSK	0.84/0.53717421

	44	83	DVNQYYIYV	0.01/1.3630435
	45	152	HLYNVTRTF	0.86/0.5089841
	46	60	ANGFFYIDV	0.67/0.69532891
	47	955	QARLNYVAL	0.16/1.2027791
	48	419	TFVDVLVNV	0.00/1.3436307
	49	468	VLPETYVAL	0.01/1.3326933
	50	143	TISGETVRL	0.93/0.41008745
	51	144	ISGETVRLH	0.88/0.44742436
	52	1098	CVKSQSNRY	0.71/0.60557634
	53	1213	DYVDVNKTL	0.65/0.66393519
	54	69	GNHRSAFAL	0.61/0.70391144
	55	795	NGNPRCKNL	0.58/0.72936105
	56	969	QENQKILAA	0.95/0.35162883
	57	1182	RLPVLSDFV	0.69/0.60633387
	58	690	SIIGAMTAV	0.49/0.80587575
	59	952	LALQARLNY	0.91/0.38221057
	60	1156	VLRQPNLVL	0.99/0.28647426
	61	899	GLSIADLAC	0.98/0.29253223
	62	890	DVDYKSCTK	0.86/0.41155337
	63	1192	IYNCNVTFV	0.24/1.0262973
	64	197	HNGRVVNYT	0.94/0.32625754
	65	1300	WLIISVVFV	/1.2571117
	66	1228	LPKYVKPNF	0.94/0.31373832
	67	308	DNLKSGVIV	0.94/0.30886148
	68	122	SSFDCIVNL	0.39/0.8545901
	69	625	NNCTKYNIY	0.77/0.46053662
	70	646	QSLAGGITY	0.99/0.23858537
	71	194	VTTHNGRVV	0.94/0.28631213
	72	31	GVPDNSSTI	0.07/1.1530067
	**73**	**1120**	**NSAPDGLLF**	**0.51/0.71103764**
	74	909	QYYNGIMVL	0.39/0.82550391
	75	1261	SLFQTTVEL	0.00/1.2047668
	76	796	GNPRCKNLL	0.90/0.30205663
	77	1087	SRRLAQQKI	0.72/0.4655759
	78	87	YYIYVTNEI	0.41/0.77168294
	79	1002	EAIHTVTIA	0.86/0.31598044
	80	902	IADLACAQY	0.96/0.21199025
	81	686	VYQQSIIGA	0.79/0.38133482
	82	1244	TYLNLSSEL	0.43/0.73997232

	83	1287	NRFENYIKW	/1.1620609
	84	667	VSTGNIFIV	0.49/0.65592697
	85	1168	NGVFRVTSR	0.78/0.3656689
	86	946	AAIPFSLAL	0.45/0.69063352
	87	437	YCDSPFEKL	0.82/0.31664561
	88	697	AVNESRYGL	0.44/0.67509782
	89	624	LNNCTKYNI	0.72/0.39111634
	90	701	SRYGLQNLL	0.00/1.1082027
	91	1042	SIQAIYDRL	1.00/0.1074006
	92	1144	WSGICVDGI	0.52/0.58640681
	93	413	WTVAFATFV	0.63/0.47556945
	94	689	QSIIGAMTA	0.68/0.42525788
	95	320	LQYDVLFYC	0.71/0.39428404
	96	361	HVSTFVGVL	0.46/0.63670723
	97	368	VLPPTVREI	0.34/0.75536966
	98	96	GLNASVTLK	0.00/1.0926166
	99	186	VNATVTVNV	0.18/0.91153623
	100	228	NGFPFNNWF	0.72/0.36993696
	101	93	NEIGLNASV	0.98/0.10922593
	102	517	VRTSHFSIR	0.84/0.24562668
	103	383	QFYINGFKY	0.79/0.28272963
	104	1303	ISVVFVVLL	/1.0702129
	105	392	FDLGFIEAV	0.72/0.3446838
	106	1073	FVSQVLNKY	0.57/0.48234715
	107	409	ATDFWTVAF	0.29/0.76233728
	108	1082	TEVRSSRRL	0.53/0.52013817
	109	80	GYYDVNQYY	0.02/1.0291098
	110	299	NLNFSANSV	0.06/0.98802186
	111	1003	AIHTVTIAL	0.22/0.82698101
	112	834	TFDSNAFSL	0.33/0.71582162
	113	806	QYTSACKTI	0.99/0.055726595
	114	67	DVGNHRSAF	0.98/0.060473689
	115	611	YPVSGIREF	0.02/1.0179848
	116	665	KNVSTGNIF	0.78/0.24818534
	117	1265	TTVELQGLI	0.69/0.32803909
	118	518	RTSHFSIRY	0.00/1.0179404
	119	58	YSANGFFYI	0.01/1.0064755
	120	1080	KYTEVRSSR	0.02/0.99452973
	121	123	SFDCIVNLL	0.18/0.83170893

	122	1216	DVNKTLQEF	0.94/0.069375114
	123	309	NLKSGVIVF	0.03/0.97633635
	124	7	LLVLPLASC	0.84/0.16380939
	125	420	FVDVLVNVS	0.90/0.099772074
	126	892	DYKSCTKGL	0.53/0.46727909
	127	156	VTRTFYVPA	0.89/0.10463975
	128	793	VCNGNPRCK	0.70/0.2923813
	129	168	LTKLSVKCY	0.98/0.011790597
	130	50	CANQSTSVY	0.83/0.1571116
	131	883	TSGLGTVDV	0.65/0.33360293
	132	482	HTDINFTAT	0.98/0.00059218188
	133	523	SIRYIYNRV	0.35/0.62967485
	134	552	FSFSKLNNF	0.45/0.52867297
	135	36	SSTIVTGLL	0.93/0.040560127
	136	1112	GTHIFSIVN	0.86/0.11035307
	137	749	NSSDNGISA	0.25/0.71807843
	138	1035	NFQAISNSI	0.68/0.28221563
	139	944	SAAAIPFSL	0.87/0.091521307
	140	81	YYDVNQYYI	0.01/0.95071361
	141	716	YVSNGGNNC	0.54/0.41685514
	142	499	VCKPHQVNI	0.67/0.28679491
	143	1108	FCGNGTHIF	0.97/-0.014700882
	144	229	GFPFNNWFL	0.03/0.92362977
	145	959	NYVALQTDV	0.66/0.29095699
	146	812	KTIEDALRL	0.44/0.50292724
	147	410	TDFWTVAFA	0.74/0.20196062
	148	141	GITISGETV	0.93/0.011474211
	149	1172	RVTSRVMFQ	0.25/0.68868661
	150	160	FYVPAAYKL	0.01/0.92154374
	151	1100	KSQSNRYGF	0.11/0.81717377
	152	770	TTSVQVEYL	0.49/0.43589624
	153	243	TLVDGVSRL	0.13/0.79565075
	154	1045	AIYDRLDSI	0.00/0.92458091
	155	1194	NCNVTFVNI	0.10/0.82143975
	156	64	FYIDVGNHR	0.00/0.91957366
	157	1028	LTSQLRHNF	0.99/-0.070453131
	158	475	ALPIYYQHT	0.88/0.036254557
	159	236	FLLTNGSTL	0.54/0.37110512

	160	700	ESRYGLQNL	0.00/0.90894505
	161	224	GRIPNGFPF	0.46/0.44783211
	162	230	FPFNNWFLL	0.24/0.66438825
	163	609	VPYPVSGIR	0.14/0.75986932
	164	960	YVALQTDVL	0.58/0.3188048
	165	1017	VVNQQGSAL	0.81/0.088394453
	166	595	ALYVTWSEG	1.00/-0.11064179
	167	120	SSSSFDCIV	0.39/0.49728833
	168	109	GINTTFDFL	0.00/0.88301886
	169	76	ALHTGYYDV	0.11/0.7710844
	170	68	VGNHRSAFA	0.85/0.030503775
	171	791	TYVCNGNPR	0.41/0.47005482
	172	1199	FVNISRVEL	0.01/0.86752135
	173	703	YGLQNLLQL	0.73/0.14329516
	174	267	KSSTGFVYF	0.22/0.65221262
	175	715	YYVSNGGNN	0.97/-0.099784989
	176	57	VYSANGFFY	0.04/0.82896505
	177	456	DGFYSANFL	0.94/-0.07281743
	178	172	SVKCYFNYS	0.92/-0.058566303
	179	1031	QLRHNFQAI	0.12/0.73975815
	180	395	GFIEAVNFN	0.93/-0.077976637
	181	310	LKSGVIVFK	0.95/-0.10067565
	182	1273	IDQINSTYV	0.52/0.32710996
	183	177	FNYSCVFSV	0.08/0.76687706
	184	105	ICKFGINTT	0.96/-0.11791621
	185	287	YQHNSVADV	0.22/0.62057052
	186	1064	TGRLAALNA	0.88/-0.040067279
	187	982	AINNIVASF	0.33/0.50952376
	188	389	FKYFDLGFI	0.89/-0.050648976
	189	185	VVNATVTVN	0.98/-0.14390927
	190	1197	VTFVNISRV	0.17/0.66507703
	191	165	AYKLTKLSV	0.25/0.57898383
	192	148	TVRLHLYNV	0.05/0.77594486
	193	136	LGAPLGITI	0.80/0.024552374
	194	1291	NYIKWPWWV	/0.81982151
	195	316	VFKTLQYDV	0.95/-0.133608
	196	97	LNASVTLKI	0.70/0.11555113
	197	865	SRIAGRSAL	0.05/0.7630997
	198	996	AITQTAEAI	0.91/-0.099155362

	199	416	AFATFVDVL	0.11/0.70016861
	200	740	GSLIPVRPR	0.00/0.80968904
	201	369	LPPTVREIV	0.59/0.21811733
	202	1298	WVWLIISVV	/0.80704834
	203	373	VREIVVART	0.93/-0.1252681
	204	827	NDVSSMLTF	0.98/-0.17792716
	205	74	AFALHTGYY	0.68/0.12115824
	206	832	MLTFDSNAF	0.74/0.060872202
	207	412	FWTVAFATF	0.90/-0.099847397
	208	967	VLQENQKIL	0.76/0.036650192
	209	921	ADAERMAMY	0.24/0.55454404
	210	1302	IISVVFVVL	/0.79428752
	211	497	CYVCKPHQV	0.04/0.75423386
	212	294	DVMRYNLNF	0.40/0.38656581
	213	901	SIADLACAQ	0.95/-0.16644765
	214	357	INTTHVSTF	0.89/-0.1069322
	215	1147	ICVDGIYGY	0.96/-0.17819768
	216	352	FINSTINTT	0.74/0.041253138
	217	247	GVSRLYQPL	0.57/0.20914439
	218	1131	TVLLPTDYK	0.16/0.6128775
	219	1170	VFRVTSRVM	0.81/-0.03960289
	220	209	DCNGYTDNI	0.26/0.50820824
	221	1285	LLNRFENYI	/0.76809085
	222	391	YFDLGFIEA	0.19/0.57516701
	223	158	RTFYVPAAY	0.53/0.23405711
	224	232	FNNWFLLTN	0.83/-0.071336717
	225	465	DDNVLPETY	0.95/-0.19146001
	226	1342	STKLPYYEF	/0.75702633
	227	184	SVVNATVTV	0.35/0.40585993
	228	558	NNFQKFKTI	0.04/0.71521064
	229	940	GGLTSAAAI	0.82/-0.065385197
	230	226	IPNGFPFNN	0.00/0.75326527
	231	653	TYVSNSGNL	0.13/0.62016707
	232	22	NANLSMLQL	0.07/0.67580278
	233	1225	AQNLPKYVK	0.02/0.72072651
	234	349	YYCFINSTI	0.02/0.71576597
	235	365	FVGVLPPTV	0.69/0.045548071
	236	808	TSACKTIED	0.89/-0.16008049
	237	384	FYINGFKYF	0.11/0.61735784

	238	159	TFYVPAAYK	0.15/0.57711365
	239	1196	NVTFVNISR	0.14/0.58609672
	240	614	SGIREFSNL	0.01/0.7142468
	241	274	YFNATGSDV	0.31/0.4094302
	242	854	SSVLPQRNI	0.22/0.49273507
	243	1086	SSRRLAQQK	0.05/0.66261708
	244	934	IGGMVLGGL	0.92/-0.21023028
	245	627	CTKYNIYDY	0.04/0.6686317
	246	354	NSTINTTHV	0.89/-0.18209401
	247	1118	IVNSAPDGL	0.63/0.067213964
	248	332	SSGVLDTTI	0.95/-0.25758336
	249	750	SSDNGISAI	0.00/0.68918248
	250	999	QTAEAIHTV	0.04/0.64864443
	251	950	FSLALQARL	0.00/0.68863499
	**252**	**1292**	**YIKWPWWVW**	**/0.68663607**
	253	1048	DRLDSIQAD	0.92/-0.23370948
	254	297	RYNLNFSAN	0.13/0.55394579
	255	279	GSDVNCNGY	0.70/-0.021040362
	256	508	SLNGNTSVC	0.31/0.36716509
	257	205	TVCDDCNGY	0.24/0.43709719
	258	985	NIVASFSSV	0.00/0.67709251
	259	1235	NFDLTPFNL	0.01/0.66392842
	260	548	GTCPFSFSK	0.01/0.66124711
	261	908	AQYYNGIMV	0.00/0.67011179
	262	313	GVIVFKTLQ	0.90/-0.23226724
	263	535	SPGDSSWHI	0.00/0.66516522
	264	978	SFNKAINNI	0.17/0.49166828
	265	466	DNVLPETYV	0.18/0.48133486
	266	1190	VQIYNCNVT	0.93/-0.26963089
	267	442	FEKLQCEHL	0.11/0.54846185
	268	556	KLNNFQKFK	0.00/0.65676664
	269	608	GVPYPVSGI	0.05/0.60612364
	270	629	KYNIYDYVG	0.95/-0.29640713
	271	730	TYSNFGICA	0.12/0.53332877
	272	422	DVLVNVSAT	0.65/0.0032103136
	273	937	MVLGGLTSA	0.95/-0.29802898
	274	526	YIYNRVKSG	0.97/-0.3220123
	275	153	LYNVTRTFY	0.53/0.11368219
	276	975	LAASFNKAI	1.00/-0.3567849

	277	1033	RHNFQAISN	0.99/-0.34988227
	278	1094	KINECVKSQ	0.73/-0.090891295
	279	258	TCLWPVPGL	0.00/0.63689405
	280	516	CVRTSHFSI	0.98/-0.34396749
	281	929	YTGSLIGGM	0.99/-0.35574078
	282	326	FYCSNSSSG	0.95/-0.32770028
	283	726	TAVMTYSNF	0.00/0.62184941
	284	1084	VRSSRRLAQ	0.99/-0.36831842
	285	544	YLKSGTCPF	0.11/0.50779975
	286	407	ASATDFWTV	0.00/0.61754287
	287	1072	AFVSQVLNK	0.01/0.60477023
	288	295	VMRYNLNFS	0.96/-0.3457583
	289	905	LACAQYYNG	0.66/-0.046858515
	290	915	MVLPGVADA	0.04/0.57104125
	291	836	DSNAFSLAN	0.98/-0.37150207
	292	898	KGLSIADLA	0.57/0.038495119
	293	562	KFKTICFST	0.88/-0.27337283
	294	741	SLIPVRPRN	0.10/0.50452706
	295	9	VLPLASCFF	0.92/-0.31727456
	296	175	CYFNYSCVF	0.95/-0.34774304
	297	1077	VLNKYTEVR	0.66/-0.059900419
	298	755	ISAIITANL	0.02/0.5761487
	299	1202	ISRVELHTV	0.53/0.062218
	300	549	TCPFSFSKL	0.18/0.41131222
	301	641	IRSSNQSLA	0.00/0.58966843
	302	920	VADAERMAM	0.59/-0.0010617828
	303	810	ACKTIEDAL	0.15/0.42832587
	304	852	NLSSVLPQR	0.05/0.52769584
	305	374	REIVVARTG	0.64/-0.062689593
	306	555	SKLNNFQKF	0.64/-0.065544177
	307	521	HFSIRYIYN	0.03/0.54244334
	308	1195	CNVTFVNIS	0.84/-0.26922709
	309	1189	FVQIYNCNV	0.00/0.56962022
	310	1219	KTLQEFAQN	0.78/-0.21340889
	311	503	HQVNISLNG	0.96/-0.39355616
	312	455	QDGFYSANF	0.94/-0.37839705
	313	406	TASATDFWT	0.88/-0.32314226
	314	334	GVLDTTIPF	0.02/0.53645384

	315	619	FSNLVLNNC	0.01/0.54575229
	316	82	YDVNQYYIY	0.81/-0.25705896
	317	571	VAVPGSCNF	0.11/0.44260339
	318	1163	VLYSDNGVF	0.91/-0.35876137
	319	479	YYQHTDINF	0.13/0.41587506
	320	1253	KQLEAKTAS	0.95/-0.40503389
	321	180	SCVFSVVNA	0.11/0.43204577
	322	78	HTGYYDVNQ	0.94/-0.40009667
	323	125	DCIVNLLFT	0.98/-0.44244182
	324	1301	LIISVVFVV	/0.53657419
	325	885	GLGTVDVDY	0.95/-0.4163575
	326	1275	QINSTYVDL	0.01/0.51961701
	327	649	AGGITYVSN	0.98/-0.45085953
	328	1037	QAISNSIQA	0.01/0.51902963
	329	838	NAFSLANVT	0.81/-0.283673
	330	225	RIPNGFPFN	0.99/-0.46715179
	331	621	NLVLNNCTK	0.00/0.52102561
	332	536	PGDSSWHIY	0.82/-0.2989773
	333	672	IFIVTPCNQ	0.96/-0.44288359
	334	919	GVADAERMA	0.04/0.4766117
	335	424	LVNVSATKI	0.76/-0.24394201
	336	154	YNVTRTFYV	0.01/0.50194357
	337	1101	SQSNRYGFC	0.85/-0.3474219
	338	600	WSEGNSITG	0.75/-0.2532342
	339	1134	LPTDYKNVK	0.69/-0.19471857
	340	504	QVNISLNGN	0.89/-0.39688785
	341	721	GNNCTTAVM	0.01/0.48010274
	342	869	GRSALEDLL	0.06/0.4283327
	343	157	TRTFYVPAA	0.01/0.47811724
	344	1184	PVLSDFVQI	0.54/-0.058458381
	345	5	LILLVLPLA	0.97/-0.48936344
	346	1062	LITGRLAAL	0.00/0.47893931
	347	490	TASFGGSCY	0.05/0.42709924
	348	1295	WPWWVWLII	/0.4757909
	349	1305	VVFVVLLSL	/0.47473929
	350	1226	QNLPKYVKP	0.92/-0.45107071
	351	1164	LYSDNGVFR	0.01/0.45767625
	352	181	CVFSVVNAT	0.01/0.45508949
	353	506	NISLNGNTS	0.87/-0.40562111

	354	302	FSANSVDNL	0.04/0.42407014
	355	495	GSCYVCKPH	0.97/-0.50770133
	356	1055	ADQQVDRLI	0.68/-0.21796388
	357	1132	VLLPTDYKN	0.90/-0.43882807
	358	767	SNWTTSVQV	0.00/0.46070684
	359	973	KILAASFNK	0.61/-0.15005263
	360	781	TSTPIVVDC	0.04/0.41970129
	361	483	TDINFTATA	0.52/-0.06251456
	362	472	TYVALPIYY	0.00/0.45712689
	363	821	SAHLETNDV	0.53/-0.074047723
	364	524	IRYIYNRVK	0.00/0.45478019
	365	725	TTAVMTYSN	0.64/-0.18585488
	366	239	TNGSTLVDG	0.89/-0.43667911
	367	837	SNAFSLANV	0.00/0.45020023
	368	32	VPDNSSTIV	0.03/0.41572927
	369	206	VCDDCNGYT	0.77/-0.32461314
	370	1139	KNVKAWSGI	0.68/-0.23558417
	371	385	YINGFKYFD	0.81/-0.36698015
	372	66	IDVGNHRSA	0.94/-0.49907895
	373	291	SVADVMRYN	0.00/0.44077119
	374	1251	ELKQLEAKT	0.97/-0.53238036
	375	1152	IYGYVLRQP	0.81/-0.37764397
	376	398	EAVNFNVTT	0.84/-0.40778528
	377	1267	VELQGLIDQ	0.91/-0.47956997
	378	538	DSSWHIYLK	0.02/0.40856322
	379	1114	HIFSIVNSA	0.00/0.42137042
	380	1148	CVDGIYGYV	0.00/0.42057261
	381	92	TNEIGLNAS	0.59/-0.17816339
	382	866	RIAGRSALE	0.77/-0.35818712
	383	1209	TVIPDYVDV	0.03/0.38178166
	384	843	ANVTSFGDY	0.72/-0.30949526
	385	912	NGIMVLPGV	0.52/-0.11400895
	386	786	VVDCATYVC	0.01/0.39261562
	387	380	RTGQFYING	0.00/0.39763001
	388	344	PSSQPYYCF	0.59/-0.19545169
	389	845	VTSFGDYNL	0.00/0.39244636
	390	116	FLSNSSSSF	0.79/-0.39952442
	391	292	VADVMRYNL	0.02/0.36732007
	392	1041	NSIQAIYDR	0.00/0.38661732

	393	762	NLSIPSNWT	0.71/-0.32634225
	394	635	YVGTGIIRS	0.61/-0.2314161
	395	1344	KLPYYEFEK	/0.37793187
	396	427	VSATKIQNL	0.01/0.36410775
	397	803	LLKQYTSAC	0.89/-0.51625964
	398	486	NFTATASFG	0.97/-0.59844676
	399	580	PLEATWHYT	0.99/-0.6187496
	400	1294	KWPWWVWLI	/0.36874339
	401	695	MTAVNESRY	0.00/0.36783213
	402	856	VLPQRNIHS	0.66/-0.29358821
	403	1181	PRLPVLSDF	0.54/-0.17438213
	404	250	RLYQPLRLT	0.88/-0.51563493
	405	307	VDNLKSGVI	0.62/-0.25756919
	406	1063	ITGRLAALN	0.89/-0.53545237
	407	170	KLSVKCYFN	0.64/-0.28554086
	408	494	GGSCYVCKP	0.90/-0.54914357
	409	1201	NISRVELHT	0.65/-0.30017658
	410	670	GNIFIVTPC	0.54/-0.19053574
	411	207	CDDCNGYTD	0.83/-0.48688557
	412	270	TGFVYFNAT	0.62/-0.28113561
	413	1115	IFSIVNSAP	0.99/-0.65832253
	414	1223	EFAQNLPKY	0.91/-0.58286731
	415	359	TTHVSTFVG	0.67/-0.34596887
	416	1158	RQPNLVLYS	0.91/-0.58726713
	417	776	EYLQITSTP	0.97/-0.65306558
	418	1254	QLEAKTASL	0.85/-0.53660417
	419	509	LNGNTSVCV	0.80/-0.48935029
	420	322	YDVLFYCSN	0.89/-0.58518661
	421	28	LQLGVPDNS	0.79/-0.49172582
	422	1203	SRVELHTVI	0.57/-0.27584068
	423	19	CNSNANLSM	0.72/-0.43013285
	424	747	PRNSSDNGI	0.79/-0.50811028
	425	330	NSSSGVLDT	0.83/-0.56128745
	426	760	TANLSIPSN	0.96/-0.69625857
	427	742	LIPVRPRNS	0.90/-0.64085629
	428	233	NNWFLLTNG	0.97/-0.71680874
	429	597	YVTWSEGNS	0.95/-0.69910405
	430	610	PYPVSGIRE	0.96/-0.70919913
	431	835	FDSNAFSLA	0.64/-0.39265703

	432	1103	SNRYGFCGN	0.67/-0.42632403
	433	718	SNGGNNCTT	0.61/-0.38121932
	434	293	ADVMRYNLN	0.75/-0.5249897
	435	314	VIVFKTLQY	0.52/-0.29505227
	436	874	EDLLFSKVV	0.99/-0.77758162
	437	14	SCFFTCNSN	0.65/-0.44179279
	438	1095	INECVKSQS	0.85/-0.64417014
	439	1143	AWSGICVDG	0.94/-0.73505255
	440	327	YCSNSSSGV	0.95/-0.74568568
	441	463	FLDDNVLPE	0.81/-0.61323046
	442	61	NGFFYIDVG	0.95/-0.75551754
	443	337	DTTIPFGPS	0.97/-0.77914943
	444	820	LSAHLETND	0.91/-0.73122578
	445	48	WICANQSTS	0.86/-0.69190279
	446	688	QQSIIGAMT	0.87/-0.71448635
	447	850	DYNLSSVLP	0.63/-0.50259028
	448	364	TFVGVLPPT	0.78/-0.65289224
	449	200	RVVNYTVCD	0.86/-0.7376643
	450	355	STINTTHVS	0.87/-0.74978859
	451	542	HIYLKSGTC	0.69/-0.57703562
	452	217	IFSVQQDGR	0.52/-0.40725701
	453	301	NFSANSVDN	0.51/-0.39917181
	454	1109	CGNGTHIFS	0.69/-0.58205052
	455	1236	FDLTPFNLT	0.97/-0.86773462
	456	1096	NECVKSQSN	0.99/-0.8964071
	457	10	LPLASCFFT	0.80/-0.70812199
	458	543	IYLKSGTCP	0.79/-0.70586974
	459	418	ATFVDVLVN	0.63/-0.54928233
	460	1043	IQAIYDRLD	0.76/-0.68606589
	461	440	SPFEKLQCE	0.87/-0.80543131
	462	179	YSCVFSVVN	0.64/-0.57767518
	463	127	IVNLLFTEQ	0.61/-0.55888341
	464	1018	VNQQGSALN	0.98/-0.92953148
	465	39	IVTGLLPTH	0.86/-0.81432357
	466	722	NNCTTAVMT	0.74/-0.70055333
	467	692	IGAMTAVNE	0.60/-0.56386442
	468	963	LQTDVLQEN	0.98/-0.94548141
	469	604	NSITGVPYP	0.52/-0.48684409
	470	452	FGLQDGFYS	0.86/-0.82733097

	471	459	YSANFLDDN	0.78/-0.7535541
	472	315	IVFKTLQYD	0.78/-0.75511057
	473	939	LGGLTSAAA	0.82/-0.79697613
	474	245	VDGVSRLYQ	0.91/-0.89247564
	475	45	PTHWICANQ	0.69/-0.6774849
	476	790	ATYVCNGNP	0.81/-0.80119268
	477	739	DGSLIPVRP	0.94/-0.93604532
	478	763	LSIPSNWTT	0.90/-0.89723455
	479	350	YCFINSTIN	0.60/-0.59842528
	480	319	TLQYDVLFY	0.61/-0.60901685
	481	652	ITYVSNSGN	0.97/-0.96911796
	482	450	LQFGLQDGF	0.92/-0.91916096
	483	568	FSTVAVPGS	0.70/-0.70282629
	484	1015	QDVVNQQGS	0.98/-0.98322824
	485	462	NFLDDNVLP	0.80/-0.80737677
	486	183	FSVVNATVT	0.66/-0.6792846
	487	421	VDVLVNVSA	0.65/-0.67314091
	488	732	SNFGICADG	0.90/-0.93035591
	489	644	SNQSLAGGI	0.70/-0.73116338
	490	990	FSSVNDAIT	0.51/-0.54952064
	491	12	LASCFFTCN	0.56/-0.60483011
	492	626	NCTKYNIYD	0.82/-0.8731964
	493	1241	FNLTYLNLS	0.97/-1.0258481
	494	744	PVRPRNSSD	0.84/-0.9073214
	495	403	NVTTASATD	0.72/-0.79478634
	496	794	CNGNPRCKN	0.75/-0.83111047
	497	766	PSNWTTSVQ	0.76/-0.85006615
	498	235	WFLLTNGST	0.79/-0.88042498
	499	37	STIVTGLLP	0.88/-0.97046583
	500	895	SCTKGLSIA	0.85/-0.94052322
	501	660	NLLGFKNVS	0.72/-0.81066823
	502	918	PGVADAERM	0.76/-0.85941545
	503	561	QKFKTICFS	0.87/-0.98208006
	504	1257	AKTASLFQT	0.85/-0.97013702
	505	775	VEYLQITST	0.59/-0.71757214
	506	498	YVCKPHQVN	0.69/-0.82879719
	507	1021	QGSALNHLT	0.90/-1.0565682
	508	246	DGVSRLYQP	0.61/-0.77281509
	509	62	GFFYIDVGN	0.94/-1.124061

	510	822	AHLETNDVS	0.92/-1.1094466
	511	557	LNNFQKFKT	0.67/-0.86273161
	512	1269	LQGLIDQIN	0.87/-1.073234
	513	1026	NHLTSQLRH	0.58/-0.78799615
	514	103	LKICKFGIN	0.55/-0.76576516
	515	620	SNLVLNNCT	0.78/-1.0111585
	516	818	LRLSAHLET	0.51/-0.74520102
	517	46	THWICANQS	0.58/-0.82444777
	518	734	FGICADGSL	0.55/-0.80259024
	519	21	SNANLSMLQ	0.62/-0.87332856
	520	651	GITYVSNSG	0.71/-0.96740514
	521	1117	SIVNSAPDG	0.80/-1.0609345
	522	375	EIVVARTGQ	0.95/-1.2191328
	523	257	LTCLWPVPG	0.69/-0.96791555
	524	704	GLQNLLQLP	0.71/-0.99244215
	525	26	SMLQLGVPD	0.72/-1.0130671
	526	593	VGALYVTWS	0.85/-1.1463538
	527	161	YVPAAYKLT	0.95/-1.2536919
	528	851	YNLSSVLPQ	0.63/-0.94170498
	529	114	FDFLSNSSS	0.81/-1.1220574
	530	691	IIGAMTAVN	0.74/-1.0588107
	531	529	NRVKSGSPG	0.57/-0.89537498
	532	496	SCYVCKPHQ	0.72/-1.0461098
	533	540	SWHIYLKSG	0.69/-1.0174474
	534	961	VALQTDVLQ	0.59/-0.9450207
	535	675	VTPCNQPDQ	0.51/-0.86667655
	536	219	SVQQDGRIP	0.63/-1.0192834
	537	882	VTSGLGTVD	0.54/-0.93667796
	538	578	NFPLEATWH	0.51/-0.9163556
	539	487	FTATASFGG	0.57/-0.99628377
	540	1056	DQQVDRLIT	0.95/-1.3798289
	541	457	GFYSANFLD	0.52/-0.95852554
	542	1123	PDGLLFLHT	0.87/-1.3505087
	543	3	LFLILLVLP	0.95/-1.4529949
	544	958	LNYVALQTD	0.73/-1.2332524
	545	596	LYVTWSEGN	0.92/-1.4279859
	546	329	SNSSSGVLD	0.74/-1.2559305
	547	280	SDVNCNGYQ	0.96/-1.5016393
	548	1252	LKQLEAKTA	0.81/-1.3727952

	549	1183	LPVLSDFVQ	0.57/-1.1331272
	550	893	YKSCTKGLS	0.89/-1.4584225
	551	241	GSTLVDGVS	0.63/-1.2090417
	552	341	PFGPSSQPY	0.80/-1.3816066
	553	551	PFSFSKLNN	0.56/-1.167181
	554	325	LFYCSNSSS	0.52/-1.1365713
	555	25	LSMLQLGVP	0.64/-1.2976153
	556	139	PLGITISGE	0.87/-1.5836941
	557	1262	LFQTTVELQ	0.55/-1.3558081
	558	435	LLYCDSPFE	0.66/-1.4793635
	559	935	GGMVLGGLT	0.58/-1.4473077
	560	336	LDTTIPFGP	0.68/-1.572947
	561	853	LSSVLPQRN	0.79/-1.7629417
	562	1160	PNLVLYSDN	0.62/-1.7062135
	563	1129	LHTVLLPTD	0.96/-2.1223174
**HCoV-229E**				
**Viral protein name**	**Rank**	**Start position**	**Sequence**	**Score ANN/SVM**
**Spike**	1	235	SYADVLVNV	0.79/1.8674984
	2	1056	QYNQTILNL	0.82/1.3531035
	3	483	VTNGTIYSI	0.90/1.0448798
	4	1101	WLNRVETYI	0.98/0.95296113
	5	295	YHKHTFIVL	0.81/1.1011519
	6	320	RPAVVNITL	0.71/1.1982227
	7	892	HTLTKYTEV	0.81/1.0859292
	8	46	GYIPSNFAF	0.99/0.87869412
	9	769	AIQSRLNYV	0.90/0.95884354
	10	607	TYVCNGNVR	0.99/0.803791
	11	1010	NCNVTFVNI	0.95/0.82143975
	12	427	KPVEGVSSF	0.82/0.95057829
	13	1077	ELNYTVQKL	0.96/0.78826668
	14	1036	TLQELSYKL	0.93/0.77813212
	15	216	AVNFNVTNA	0.24/1.4507931
	16	277	TSPIQSVEL	0.87/0.81769459
	17	896	KYTEVRASR	0.84/0.83155092
	18	406	SHNIGSLYV	0.92/0.73114542
	19	201	FYINGYRYF	0.42/1.1868815
	20	558	IIAVQPRNV	1.00/0.60525918
	21	49	PSNFAFNNW	0.78/0.80918015

	22	368	FSFGKVNNF	0.86/0.7209464
	23	516	FTSYGFSNV	0.99/0.57238523
	24	52	FAFNNWFLL	0.93/0.62976246
	25	289	IVSLPVYHK	0.62/0.93506074
	26	985	NYYRITSRI	0.59/0.95204458
	27	525	VEMPKFFYA	0.88/0.64569606
	28	685	GRSAIEDIL	0.99/0.52356122
	29	937	AAPEGLVFL	0.80/0.70872321
	30	323	VVNITLANF	0.71/0.78885227
	31	731	MVLPGVADA	0.92/0.57104125
	32	89	RFTTGFVYF	0.48/1.0076679
	33	916	KSQSKRYGF	0.72/0.76151462
	34	162	VLGNFYCFV	0.29/1.1825707
	35	1025	TIVPEYIDV	0.70/0.76114642
	36	1029	EYIDVNKTL	0.61/0.84239534
	37	232	ALASYADVL	0.85/0.59711401
	38	417	SDGDVITGV	0.98/0.4623578
	39	206	YRYFSLGNV	0.82/0.61256483
	40	249	ANIIYCNSV	0.99/0.43179498
	41	583	SNWTTSVQV	0.93/0.46070684
	42	451	VSGVGVIRI	0.50/0.8906717
	43	294	VYHKHTFIV	0.67/0.71297662
	44	613	NVRCVELLK	0.92/0.45735117
	45	111	NASSDVIRY	0.54/0.81078762
	46	1118	CISVVLIFV	/1.340019
	47	567	SYDSVSAIV	0.03/1.2935833
	48	785	QENQKILAA	0.96/0.35162883
	49	882	RLAALNVFV	0.02/1.2836399
	50	346	TTQFVGVKF	0.74/0.54546205
	51	991	SRIMFEPRI	0.69/0.59218388
	52	394	AMPIMANLV	0.77/0.50575436
	53	760	SAASIPFSL	0.99/0.28566631
	54	115	DVIRYNINF	0.67/0.60410097
	55	661	VSSFGDYNL	0.62/0.6461
	56	135	KTSYGAVVF	1.00/0.25763561
	57	694	FSKLVTSGL	0.91/0.34420326
	58	369	SFGKVNNFV	0.98/0.26941735
	59	178	TTSAFVGAL	0.56/0.6823268
	60	307	FELRRGPGR	0.88/0.3609089

	61	847	QLRQNFQAI	0.67/0.56653056
	62	689	IEDILFSKL	0.59/0.64639245
	63	393	CAMPIMANL	0.39/0.84452801
	64	678	RSGSRVAGR	0.28/0.9520858
	65	302	VLYVNFELR	0.85/0.38186099
	66	903	SRQLAQQKV	0.88/0.34964962
	67	771	QSRLNYVAL	0.09/1.1311114
	68	189	TVREFVISR	0.15/1.0555086
	69	252	IYCNSVINR	0.48/0.71575058
	70	1103	NRVETYIKW	0.30/0.89489893
	71	374	NNFVKFGSV	0.86/0.33249972
	72	127	LRRGTILFK	0.64/0.54941191
	73	376	FVKFGSVCF	0.58/0.60784418
	74	735	GVADAERMA	0.71/0.4766117
	75	316	CYNCRPAVV	0.28/0.90276199
	76	766	FSLAIQSRL	0.95/0.21696195
	77	601	IVVDCSTYV	0.25/0.91605442
	78	166	FYCFVNTTI	0.72/0.44509881
	79	1087	TLIDNINST	0.80/0.36106377
	80	3	VLLVAYALL	0.96/0.19537306
	81	747	GSLIGGIAL	0.09/1.0482509
	82	718	IADLACAQY	0.92/0.21199025
	83	66	VVDGVVRSF	0.58/0.54851216
	84	342	TSHFTTQFV	0.47/0.65637686
	85	930	HIFSLVNAA	0.75/0.37491738
	86	124	EENLRRGTI	0.06/1.0626125
	87	401	LVNHKSHNI	0.61/0.50902711
	88	914	CVKSQSKRY	0.86/0.25632977
	89	885	ALNVFVSHT	0.94/0.17364435
	90	397	IMANLVNHK	0.85/0.25302582
	91	990	TSRIMFEPR	0.98/0.11959916
	92	513	SENFTSYGF	0.75/0.34772899
	93	443	CTKYNIYDV	0.51/0.58622773
	94	725	QYYNGIMVL	0.27/0.82550391
	95	537	TYNCTDAVL	0.00/1.0931999
	96	73	SFQPLLLNC	0.98/0.10832622
	97	301	IVLYVNFEL	0.84/0.24266543
	98	972	VLRQPNLAL	0.64/0.43922754
	99	297	KHTFIVLYV	0.71/0.36403013

	100	112	ASSDVIRYN	0.70/0.3726522
	101	877	RLITGRLAA	0.90/0.1717323
	102	1041	SYKLPNYTV	0.61/0.44278541
	103	1094	STLVDLKWL	0.85/0.19147491
	104	1	MFVLLVAYA	0.53/0.50741409
	105	209	FSLGNVEAV	0.01/1.0208665
	106	1084	KLQTLIDNI	0.73/0.2981202
	107	1121	VVLIFVVSM	/1.0266072
	108	450	DVSGVGVIR	0.14/0.88629946
	109	318	NCRPAVVNI	0.15/0.86753865
	110	203	INGYRYFSL	0.83/0.1868726
	111	970	GYVLRQPNL	0.07/0.94512873
	112	492	TPCNPPDQL	0.83/0.18329478
	113	640	LESADVSEM	0.92/0.091135185
	114	94	FVYFNGTGR	0.58/0.43057183
	115	70	VVRSFQPLL	0.94/0.065829315
	116	1096	LVDLKWLNR	0.02/0.98490518
	117	282	SVELPVSIV	0.02/0.98416244
	118	299	TFIVLYVNF	0.18/0.82148897
	119	462	DTFLNGITY	0.05/0.95137086
	120	553	CADGSIIAV	0.49/0.50823926
	121	1008	IENCNVTFV	0.14/0.84796279
	122	1032	DVNKTLQEL	0.22/0.76726182
	123	345	FTTQFVGVK	0.49/0.49421911
	124	675	SLPRSGSRV	0.00/0.97343014
	125	840	SLNHLTSQL	0.02/0.95158788
	126	870	QADQQVDRL	0.81/0.16036435
	127	947	TVLLPTQYK	0.10/0.86981699
	128	996	EPRIPTIAD	0.96/0.0095154556
	129	864	DRLDIIQAD	0.99/-0.028714089
	130	1005	FVQIENCNV	0.56/0.38784067
	131	1102	LNRVETYIK	0.98/-0.039698991
	132	994	MFEPRIPTI	0.10/0.82822264
	133	782	DVLQENQKI	0.94/-0.018259514
	134	861	AIYDRLDII	0.00/0.91534075
	135	5	LVAYALLHI	0.74/0.17394567
	136	643	ADVSEMLTF	0.99/-0.077213855
	137	119	YNINFEENL	0.05/0.85968561
	138	364	GNCPFSFGK	0.42/0.48731197

	139	703	GTVDADYKK	0.99/-0.10022226
	140	185	ALPKTVREF	0.17/0.71973496
	141	874	QVDRLITGR	0.66/0.22945282
	142	646	SEMLTFDKK	0.94/-0.053857407
	143	36	SENVFAVES	0.98/-0.10024764
	144	472	STSGNLLGF	0.80/0.078257942
	145	802	IVDAFTGVN	0.42/0.45761822
	146	104	DCKGFYSNA	0.15/0.72682753
	147	681	SRVAGRSAI	0.26/0.61071974
	148	405	KSHNIGSLY	0.27/0.60015863
	149	722	ACAQYYNGI	0.74/0.12818655
	150	1001	TIADFVQIE	0.29/0.57621536
	151	75	QPLLLNCLW	0.98/-0.11717826
	152	26	HSVCNGCVG	1.00/-0.14273432
	153	608	YVCNGNVRC	0.65/0.20553062
	154	200	HFYINGYRY	0.10/0.75470995
	155	974	RQPNLALYK	0.41/0.44467609
	156	1080	YTVQKLQTL	0.74/0.11050933
	157	532	YASNGTYNC	0.59/0.25669996
	158	223	NAATTVCTV	0.43/0.41024426
	159	876	DRLITGRLA	0.79/0.045018785
	160	812	AITQTSQAL	0.42/0.41468226
	161	281	QSVELPVSI	0.96/-0.12663657
	162	348	QFVGVKFDR	0.93/-0.1090081
	163	453	GVGVIRISN	0.83/-0.025506206
	164	697	LVTSGLGTV	0.97/-0.16600661
	165	311	RGPGRCYNC	0.02/0.7799757
	166	611	NGNVRCVEL	0.01/0.78743139
	167	858	SIQAIYDRL	0.69/0.1074006
	168	434	SFMNVTLNK	0.88/-0.083120717
	169	854	AISSSIQAI	0.56/0.23386998
	170	1035	KTLQELSYK	0.06/0.72787522
	171	34	GHSENVFAV	0.14/0.64571548
	172	762	ASIPFSLAI	0.26/0.52224994
	173	801	NIVDAFTGV	0.09/0.68411889
	174	1114	WVWLCISVV	/0.77228223
	175	319	CRPAVVNIT	0.61/0.16148364
	176	1107	TYIKWPWWV	0.00/0.77137517
	177	789	KILAASFNK	0.92/-0.15005263

	178	378	KFGSVCFSL	0.06/0.70669931
	179	980	LYKEGNYYR	0.24/0.52449807
	180	566	VSYDSVSAI	0.10/0.66293112
	181	149	TLVSGDAHI	0.14/0.60636099
	182	573	AIVTANLSI	0.88/-0.13653229
	183	940	EGLVFLHTV	0.89/-0.14936414
	184	2	FVLLVAYAL	0.00/0.73925828
	185	169	FVNTTIGNE	0.95/-0.21603387
	186	446	YNIYDVSGV	0.90/-0.16686651
	187	724	AQYYNGIMV	0.06/0.67011179
	188	234	ASYADVLVN	0.96/-0.23019959
	189	656	FTLANVSSF	0.66/0.064119553
	190	986	YYRITSRIM	0.01/0.70525022
	191	1100	KWLNRVETY	0.12/0.59135612
	192	910	KVNECVKSQ	0.54/0.16973449
	193	353	KFDRWSASI	0.08/0.62899224
	194	448	IYDVSGVGV	0.24/0.46726532
	195	196	SRTGHFYIN	0.99/-0.28548587
	196	143	FYCTNNTLV	0.02/0.67709199
	197	907	AQQKVNECV	0.92/-0.22454834
	198	137	SYGAVVFYC	0.00/0.69264725
	199	981	YKEGNYYRI	0.00/0.69189128
	200	341	DTSHFTTQF	0.29/0.39790601
	**201**	**1108**	**YIKWPWWVW**	**/0.68663607**
	202	668	NLSSVIPSL	0.04/0.6464634
	203	1086	QTLIDNINS	0.99/-0.30454623
	204	688	AIEDILFSK	0.29/0.38699398
	205	765	PFSLAIQSR	0.89/-0.21756993
	206	929	THIFSLVNA	0.69/-0.018795332
	207	524	VVEMPKFFY	0.98/-0.30957933
	208	1019	SRSELQTIV	0.16/0.50968915
	209	387	KDIPGGCAM	0.11/0.55922917
	210	851	NFQAISSSI	0.67/-0.0030036502
	211	218	NFNVTNAAT	0.76/-0.094476459
	212	356	RWSASINTG	0.90/-0.23586429
	213	154	DAHIPSGTV	0.20/0.46166747
	214	198	TGHFYINGY	0.76/-0.099687321
	215	241	VNVSQTAIA	0.84/-0.17998223

	216	521	FSNVVEMPK	0.81/-0.15167492
	217	253	YCNSVINRL	0.00/0.6486805
	218	560	AVQPRNVSY	0.01/0.63705648
	219	536	GTYNCTDAV	0.62/0.020826311
	220	982	KEGNYYRIT	0.99/-0.35082543
	221	1092	INSTLVDLK	0.97/-0.33162479
	**222**	**936**	**NAAPEGLVF**	**0.04/0.59776222**
	223	571	VSAIVTANL	0.03/0.59471685
	224	717	SIADLACAQ	0.79/-0.16644765
	225	136	TSYGAVVFY	0.17/0.4473033
	226	542	DAVLTYSSF	0.01/0.60335384
	227	113	SSDVIRYNI	0.01/0.60002135
	228	1012	NVTFVNISR	0.02/0.58609672
	229	1089	IDNINSTLV	0.51/0.094785561
	230	312	GPGRCYNCR	0.61/-0.0062245289
	231	1070	TLENKSAEL	0.08/0.51648165
	232	818	QALQTVATA	0.00/0.59629874
	233	165	NFYCFVNTT	0.53/0.065864476
	234	792	AASFNKAMT	0.65/-0.057975537
	235	415	SWSDGDVIT	0.99/-0.39962906
	236	609	VCNGNVRCV	0.14/0.45011757
	237	902	ASRQLAQQK	0.04/0.54710832
	238	246	TAIANIIYC	0.00/0.58374967
	239	578	NLSIPSNWT	0.91/-0.32634225
	240	732	VLPGVADAE	0.98/-0.39679437
	241	1049	VPDLVVEQY	0.02/0.55750201
	242	1015	FVNISRSEL	0.04/0.53516845
	243	737	ADAERMAMY	0.02/0.55454404
	244	886	LNVFVSHTL	0.96/-0.38847497
	245	24	TSHSVCNGC	0.13/0.44104516
	246	671	SVIPSLPRS	0.60/-0.029356677
	247	74	FQPLLLNCL	0.01/0.55990136
	248	1045	PNYTVPDLV	0.91/-0.3424814
	249	1022	ELQTIVPEY	0.81/-0.24544621
	250	829	KIQDVVNQQ	0.97/-0.40984551
	251	464	FLNGITYTS	0.66/-0.10644623
	252	835	NQQGNSLNH	0.98/-0.43019055

	253	511	MLSENFTSY	0.55/-0.0020421399
	254	197	RTGHFYING	0.00/0.54784924
	255	546	TYSSFGVCA	0.00/0.54592906
	256	519	YGFSNVVEM	0.07/0.47486415
	257	1091	NINSTLVDL	0.00/0.5390921
	258	1156	STKLPYYDV	/0.53783653
	259	1116	WLCISVVLI	/0.53187977
	260	439	TLNKCTKYN	0.90/-0.37017565
	261	225	ATTVCTVAL	0.14/0.38865657
	262	963	LCVDGINGY	0.86/-0.33411288
	263	867	DIIQADQQV	0.57/-0.046201068
	264	351	GVKFDRWSA	0.00/0.52357451
	265	905	QLAQQKVNE	0.97/-0.44737215
	266	641	ESADVSEML	0.00/0.51824056
	267	825	TALNKIQDV	0.79/-0.2730957
	268	523	NVVEMPKFF	0.85/-0.33492762
	269	445	KYNIYDVSG	0.86/-0.35250506
	270	865	RLDIIQADQ	0.81/-0.30272767
	271	917	SQSKRYGFC	0.88/-0.37717579
	272	77	LLLNCLWSV	0.14/0.36169184
	273	973	LRQPNLALY	0.01/0.49040965
	274	161	TVLGNFYCF	0.01/0.48814844
	275	310	RRGPGRCYN	0.00/0.49270729
	276	51	NFAFNNWFL	0.00/0.48776454
	277	744	MYTGSLIGG	0.85/-0.36553556
	278	69	GVVRSFQPL	0.06/0.42212265
	279	878	LITGRLAAL	0.00/0.47893931
	280	701	GLGTVDADY	0.95/-0.4719181
	281	612	GNVRCVELL	0.00/0.47415948
	282	952	TQYKDVEAW	0.67/-0.19763242
	283	262	RCDQLSFDV	0.55/-0.080345705
	284	898	TEVRASRQL	0.00/0.46636497
	285	908	QQKVNECVK	0.58/-0.11443267
	286	708	DYKKCTKGL	0.10/0.36496436
	287	37	ENVFAVESG	0.90/-0.4378157
	288	602	VVDCSTYVC	0.00/0.46013971
	289	327	TLANFNETK	0.00/0.45979244
	290	597	TSTPIVVDC	0.04/0.41970129

	291	88	SRFTTGFVY	0.52/-0.061584829
	292	586	TTSVQVEYL	0.02/0.43589624
	293	255	NSVINRLRC	0.03/0.42406909
	294	372	KVNNFVKFG	0.00/0.45339313
	295	626	ACKTIEDAL	0.02/0.42832587
	296	1039	ELSYKLPNY	0.00/0.44555067
	297	819	ALQTVATAL	0.00/0.44195641
	298	308	ELRRGPGRC	0.58/-0.14132533
	299	155	AHIPSGTVL	0.00/0.43257788
	300	322	AVVNITLAN	0.80/-0.37110654
	301	650	TFDKKAFTL	0.00/0.42624886
	302	245	QTAIANIIY	0.01/0.41188351
	303	846	SQLRQNFQA	0.66/-0.24359989
	304	628	KTIEDALRN	0.77/-0.35564078
	305	38	NVFAVESGG	0.73/-0.32178622
	306	315	RCYNCRPAV	0.01/0.39397177
	307	889	FVSHTLTKY	0.63/-0.22663645
	308	633	ALRNSAMLE	0.53/-0.12694683
	309	358	SASINTGNC	0.01/0.39131931
	310	396	PIMANLVNH	0.84/-0.4402206
	311	606	STYVCNGNV	0.03/0.36592377
	312	1142	FFSCFASSI	/0.39565224
	313	887	NVFVSHTLT	0.96/-0.56489893
	314	942	LVFLHTVLL	0.73/-0.33703446
	315	857	SSIQAIYDR	0.02/0.37213588
	316	517	TSYGFSNVV	0.00/0.38948488
	317	508	VGAMLSENF	0.74/-0.35252647
	318	62	NTSSVVDGV	0.01/0.37533965
	319	632	DALRNSAML	0.01/0.36842498
	320	452	SGVGVIRIS	0.85/-0.47314963
	321	1113	WWVWLCISV	/0.3685549
	322	1138	GCCGFFSCF	/0.36600621
	323	1117	LCISVVLIF	/0.36038831
	324	590	QVEYLQITS	0.99/-0.6333917
	325	156	HIPSGTVLG	0.77/-0.41714308
	326	1088	LIDNINSTL	0.97/-0.62490563
	327	179	TSAFVGALP	0.76/-0.41598162
	328	470	YTSTSGNLL	0.96/-0.62167019
	329	912	NECVKSQSK	0.51/-0.17224333

	330	367	PFSFGKVNN	0.96/-0.63819861
	331	236	YADVLVNVS	0.62/-0.30480027
	332	605	CSTYVCNGN	0.84/-0.52834667
	333	716	LSIADLACA	0.96/-0.65097904
	334	803	VDAFTGVND	0.55/-0.25543738
	335	1081	TVQKLQTLI	0.53/-0.24006154
	336	745	YTGSLIGGI	0.54/-0.25176861
	337	1026	IVPEYIDVN	0.90/-0.6121174
	338	592	EYLQITSTP	0.94/-0.65306558
	339	790	ILAASFNKA	0.86/-0.57923679
	340	738	DAERMAMYT	0.70/-0.42524277
	341	843	HLTSQLRQN	0.90/-0.62566165
	342	1011	CNVTFVNIS	0.54/-0.26922709
	343	78	LLNCLWSVS	0.69/-0.42631526
	344	402	VNHKSHNIG	0.88/-0.62056885
	345	772	SRLNYVALQ	0.60/-0.34824453
	346	273	GFYSTSPIQ	0.99/-0.73964479
	347	993	IMFEPRIPT	0.55/-0.30814447
	348	625	SACKTIEDA	0.97/-0.73039223
	349	883	LAALNVFVS	0.98/-0.74296752
	350	1034	NKTLQELSY	0.99/-0.75423339
	351	1061	ILNLTSEIS	0.97/-0.73497931
	352	1054	VEQYNQTIL	0.52/-0.28622372
	353	687	SAIEDILFS	0.81/-0.57788787
	354	557	SIIAVQPRN	0.81/-0.57822068
	355	292	LPVYHKHTF	0.51/-0.30073016
	356	1013	VTFVNISRS	0.57/-0.370745
	357	192	EFVISRTGH	0.67/-0.47310592
	358	568	YDSVSAIVT	0.75/-0.55609741
	359	455	GVIRISNDT	0.72/-0.53619664
	360	475	GNLLGFKDV	0.61/-0.42674079
	361	97	FNGTGRGDC	0.74/-0.5576958
	362	925	CGNGTHIFS	0.76/-0.58205052
	363	272	DGFYSTSPI	0.75/-0.57516659
	364	649	LTFDKKAFT	0.81/-0.6382898
	365	509	GAMLSENFT	0.87/-0.69861697
	366	237	ADVLVNVSQ	0.98/-0.83313
	367	181	AFVGALPKT	0.81/-0.67288943
	368	96	YFNGTGRGD	0.98/-0.84910663

	369	19	TNGTNTSHS	0.95/-0.81915444
	370	361	INTGNCPFS	0.57/-0.44375531
	371	168	CFVNTTIGN	0.92/-0.8073517
	372	204	NGYRYFSLG	0.61/-0.49905751
	373	152	SGDAHIPSG	0.70/-0.59317155
	374	850	QNFQAISSS	0.91/-0.8067722
	375	1021	SELQTIVPE	0.65/-0.5495362
	376	13	IAGCQTTNG	0.72/-0.62907467
	377	1073	NKSAELNYT	0.75/-0.6682695
	378	274	FYSTSPIQS	0.66/-0.58502157
	379	43	ESGGYIPSN	0.86/-0.78829773
	380	333	ETKGPLCVD	0.60/-0.53306782
	381	12	HIAGCQTTN	0.88/-0.82711406
	382	207	RYFSLGNVE	0.57/-0.51871077
	383	400	NLVNHKSHN	0.91/-0.85980582
	384	106	KGFYSNASS	0.91/-0.86585437
	385	712	CTKGLSIAD	0.76/-0.72347179
	386	331	FNETKGPLC	0.89/-0.85467211
	387	411	SLYVSWSDG	0.65/-0.61546482
	388	989	ITSRIMFEP	0.57/-0.54334517
	389	879	ITGRLAALN	0.56/-0.53545237
	390	268	FDVPDGFYS	0.77/-0.76135237
	391	663	SFGDYNLSS	0.90/-0.92363067
	392	831	QDVVNQQGN	0.96/-0.98436847
	393	698	VTSGLGTVD	0.91/-0.93667796
	394	939	PEGLVFLHT	0.81/-0.84117946
	395	17	QTTNGTNTS	0.97/-1.0132252
	396	564	RNVSYDSVS	1.00/-1.0474162
	397	305	VNFELRRGP	0.97/-1.0341897
	398	468	ITYTSTSGN	0.71/-0.77757767
	399	968	INGYVLRQP	0.56/-0.6406024
	400	335	KGPLCVDTS	0.72/-0.80694304
	401	157	IPSGTVLGN	0.81/-0.89919577
	402	80	NCLWSVSGS	0.66/-0.75737334
	403	399	ANLVNHKSH	0.93/-1.0292441
	404	418	DGDVITGVP	0.78/-0.88356208
	405	1082	VQKLQTLID	0.78/-0.89669535
	406	285	LPVSIVSLP	0.52/-0.65196154
	407	644	DVSEMLTFD	0.70/-0.84566535

	408	555	DGSIIAVQP	0.90/-1.0553293
	409	734	PGVADAERM	0.70/-0.85941545
	410	535	NGTYNCTDA	0.55/-0.72438715
	411	813	ITQTSQALQ	0.79/-0.98447689
	412	328	LANFNETKG	0.55/-0.74500319
	413	693	LFSKLVTSG	0.98/-1.1754057
	414	593	YLQITSTPI	0.79/-0.99429385
	415	875	VDRLITGRL	0.68/-0.88516301
	416	76	PLLLNCLWS	0.82/-1.0499466
	417	589	VQVEYLQIT	0.89/-1.1386482
	418	654	KAFTLANVS	0.97/-1.2275132
	419	172	TTIGNETTS	0.91/-1.1715195
	420	962	GLCVDGING	0.73/-0.99284111
	421	128	RRGTILFKT	0.70/-0.96898117
	422	265	QLSFDVPDG	0.89/-1.1631618
	423	528	PKFFYASNG	0.75/-1.0233617
	424	505	QAVVGAMLS	0.56/-0.85011124
	425	29	CNGCVGHSE	0.61/-0.90378002
	426	515	NFTSYGFSN	0.77/-1.0820661
	427	705	VDADYKKCT	0.93/-1.2732464
	428	1031	IDVNKTLQE	0.84/-1.1900464
	429	866	LDIIQADQQ	0.99/-1.3408012
	430	56	NWFLLTNTS	0.80/-1.1595931
	431	105	CKGFYSNAS	0.51/-0.87994452
	432	496	PPDQLVVYQ	0.66/-1.033688
	433	366	CPFSFGKVN	0.64/-1.0220149
	434	1030	YIDVNKTLQ	0.54/-0.94559946
	435	1085	LQTLIDNIN	0.53/-0.94715365
	436	67	VDGVVRSFQ	0.63/-1.0473691
	437	976	PNLALYKEG	0.99/-1.4108035
	438	788	QKILAASFN	0.83/-1.3005132
	439	757	GLTSAASIP	0.56/-1.0697704
	440	9	ALLHIAGCQ	0.55/-1.0601386
	441	436	MNVTLNKCT	0.88/-1.4132269
	442	79	LNCLWSVSG	0.93/-1.4709827
	443	827	LNKIQDVVN	0.82/-1.3779429
	444	329	ANFNETKGP	0.90/-1.4704893
	445	68	DGVVRSFQP	0.78/-1.3561402
	446	868	IIQADQQVD	0.84/-1.4393487

	447	1067	EISTLENKS	0.78/-1.3808414
	448	1068	ISTLENKSA	0.76/-1.3634631
	449	22	TNTSHSVCN	0.59/-1.2011995
	450	30	NGCVGHSEN	0.65/-1.2884225
	451	467	GITYTSTSG	0.54/-1.3242179
	452	695	SKLVTSGLG	0.60/-1.4684949
	453	749	LIGGIALGG	0.52/-1.765249
**HCoV-OC43**				
**Viral protein name**	**Rank**	**Start position**	**Sequence**	**Score ANN/SVM**
**Spike**	1	1047	ALNNLLQQL	0.95/1.4112217
	2	698	FRNIKCNYV	0.95/1.1709293
	3	987	FYLNVQYRI	0.98/1.090522
	4	1273	NRLQEAIKV	0.94/1.1290627
	5	881	GVNFNVDDI	0.62/1.3894502
	6	1085	LINGRLTAL	0.91/1.0044934
	7	1089	RLTALNAYV	0.68/1.0179382
	8	1227	MLNTSIPNL	0.93/0.76203536
	9	456	FNPSTWNKR	0.97/0.72124296
	10	1303	WYVWLLICL	/1.6803307
	11	1	MFLILLISL	0.95/0.72279412
	12	620	IELGVCVNY	0.92/0.74972224
	13	732	AYNSTAISV	0.21/1.4529222
	14	209	TYDVNATYL	0.19/1.46477
	15	646	YYNSWQNLL	0.99/0.6301557
	16	1068	EILSRLDAL	0.99/0.61409618
	17	438	TSCQLYYNL	0.23/1.3659761
	18	240	KFLFNVYLG	0.88/0.68603567
	19	841	SFCDNINAI	0.31/1.2510239
	20	445	NLPAANVSV	0.86/0.69940644
	21	455	RFNPSTWNK	0.68/0.85397591
	22	1050	NLLQQLSNR	0.93/0.59233873
	23	724	SYLGCVVNA	0.99/0.53149928
	24	1274	RLQEAIKVL	0.84/0.67054773
	25	1123	KSQSSRINF	0.96/0.5384127
	26	1012	IANAFNNAL	0.99/0.49337339
	27	707	FNNSLTRQL	0.86/0.60523812
	28	49	TNGLGTYYV	0.53/0.91235578
	29	1276	QEAIKVLNH	0.99/0.45116678

	30	61	VYLNTTLFL	0.83/0.59556931
	31	78	STYRNMALK	0.18/1.2296772
	32	73	YPTSGSTYR	0.71/0.68884631
	33	1281	VLNHSYINL	0.35/1.0482089
	34	59	DRVYLNTTL	0.91/0.48591595
	35	425	YLQSSNYRI	0.96/0.41883581
	36	1033	ALVKIQAVV	1.00/0.37157227
	37	1065	SLQEILSRL	0.31/1.0593795
	38	1234	NLPDFKEEL	0.04/1.3193041
	39	645	TYYNSWQNL	0.19/1.1651561
	40	44	DTVDVTNGL	0.93/0.41931319
	41	180	ICHPNLGNH	0.92/0.4269388
	42	900	SECSKASSR	0.94/0.39299948
	43	55	YYVLDRVYL	0.71/0.62291031
	44	1193	MYTGSGYYY	0.97/0.36147644
	45	848	AILTEVNEL	0.14/1.1749989
	46	774	FEPFTVNSV	0.01/1.3022989
	47	53	GTYYVLDRV	0.69/0.60199689
	48	886	VDDINFSPV	0.83/0.45986232
	49	883	NFNVDDINF	0.78/0.50752128
	50	573	CQPQAFLGW	0.99/0.28651332
	51	823	AFVCGDYAA	0.99/0.28228767
	52	801	FTIGNMEEF	0.98/0.29156448
	53	238	VTKFLFNVY	0.62/0.63532879
	54	934	TGGAEIRDL	0.93/0.3175666
	55	103	DFINGIFAK	0.96/0.28254372
	56	372	NMSSLMSFI	0.68/0.54977987
	57	221	FYQEGGTFY	0.81/0.41757152
	58	232	FTDTGFVTK	0.82/0.40702178
	59	314	IAPPTGVYE	0.95/0.27336411
	60	86	KGTDLLSTL	0.90/0.31778329
	61	249	MALSHYYVM	0.56/0.65690464
	62	300	MSDFMSEIK	0.98/0.23444278
	63	75	TSGSTYRNM	0.01/1.2010616
	64	553	GEHCSGLAV	0.90/0.30660072
	65	172	NMCEYPHTI	0.90/0.30410877
	66	104	FINGIFAKV	0.55/0.65009989
	67	695	ALLFRNIKC	1.00/0.190667
	68	102	SDFINGIFA	1.00/0.19053218

	69	1185	FVNVNNTWM	0.44/0.74167686
	70	93	TLWFKPPFL	0.48/0.69472086
	71	1121	CVKSQSSRI	0.64/0.5260796
	72	1029	ATNSALVKI	0.98/0.17734067
	73	353	KSVPSPLNW	0.90/0.25572466
	74	705	YVFNNSLTR	0.92/0.23398838
	75	1255	DLSLDYINV	0.30/0.84797395
	76	257	MPLTCISRR	0.95/0.19667284
	77	315	APPTGVYEL	0.02/1.1244147
	78	288	NQDGIIFNA	0.53/0.61375342
	79	7	ISLPTAFAV	0.04/1.1025963
	80	443	YYNLPAANV	0.11/1.0288998
	81	1149	LYFIHFNYV	0.03/1.1084429
	82	1043	ANAEALNNL	0.05/1.0871314
	83	312	QSIAPPTGV	0.83/0.30513685
	84	415	KVDLQLGNL	0.01/1.1246018
	85	286	AFNQDGIIF	0.37/0.76161999
	86	759	RRSRRAITT	0.58/0.54861985
	87	1184	YFVNVNNTW	1.00/0.11331374
	88	237	FVTKFLFNV	0.50/0.61279072
	89	983	AGVPFYLNV	0.24/0.86902301
	90	89	DLLSTLWFK	0.49/0.60690967
	91	838	EYGSFCDNI	0.03/1.0662257
	92	693	EPALLFRNI	0.20/0.89291034
	93	165	EVSVCQYNM	0.43/0.6575204
	94	1205	ITENNVVVM	0.61/0.47194715
	95	1297	YYVKWPWYV	/1.0811943
	96	587	LQGDKCNIF	0.79/0.29042741
	97	593	NIFANFILH	0.84/0.23956208
	98	840	GSFCDNINA	0.77/0.30676157
	99	1062	ISASLQEIL	0.54/0.53070597
	100	299	CMSDFMSEI	0.00/1.0705229
	101	1218	VNYTKAPYV	0.75/0.31957114
	102	755	YSKNRRSRR	0.09/0.97754
	103	481	FTNHSVVYA	0.03/1.0358614
	104	663	GFRDYITNR	0.73/0.33455557
	105	72	YYPTSGSTY	0.90/0.16318297
	106	1267	DLQVEMNRL	0.01/1.0440607
	107	486	VVYAQHCFK	0.81/0.23959963

	108	1264	TFLDLQVEM	0.03/1.0192909
	109	190	KELWHLDTG	0.99/0.054476873
	110	408	FAIPNRRKV	0.31/0.73333435
	111	100	FLSDFINGI	0.03/1.0039439
	112	889	INFSPVLGC	0.97/0.05905104
	113	214	ATYLYFHFY	0.50/0.52759043
	114	869	MNGVTLSTK	0.92/0.1047512
	115	387	CNNIDAAKI	0.97/0.051703862
	116	1315	AMLVLLFFI	/1.0168347
	117	1284	HSYINLKDI	0.75/0.26674026
	118	1008	NQKLIANAF	0.25/0.76483172
	119	410	IPNRRKVDL	0.00/1.0144714
	120	981	AAAGVPFYL	0.06/0.95135741
	121	175	EYPHTICHP	0.98/0.029419481
	122	26	RLKGSFNNR	0.08/0.92219217
	123	68	FLNGYYPTS	0.99/0.0075039235
	124	279	TPRQYLLAF	0.65/0.34707301
	**125**	**1298**	**YVKWPWYVW**	**/0.9882908**
	126	1220	YTKAPYVML	0.10/0.88798981
	127	152	STQDGVNKL	0.58/0.40302599
	128	831	ACKLQLVEY	0.41/0.57250738
	129	452	SVSRFNPST	0.88/0.097310705
	130	916	FDKVKLSDV	0.99/-0.013587218
	131	395	IYGMCFSSI	0.00/0.96874448
	132	660	NLYGFRDYI	0.31/0.65430472
	133	1114	AMEKVNECV	0.98/-0.027050296
	134	664	FRDYITNRT	0.91/0.041126691
	135	248	GMALSHYYV	0.41/0.53802265
	136	223	QEGGTFYAY	0.51/0.43651074
	137	336	RRKPDLPNC	0.58/0.35549882
	138	598	FILHDVNNG	0.89/0.041984006
	139	762	RRAITTGYR	0.01/0.91189376
	140	1208	NNVVVMSTC	0.97/-0.062852773
	141	971	TSASLFPPW	0.58/0.31571442
	142	1278	AIKVLNHSY	0.88/0.009819656
	143	984	GVPFYLNVQ	0.97/-0.081793283
	144	1126	SSRINFCGN	0.80/0.087054246
	145	50	NGLGTYYVL	0.49/0.39410372
	146	47	DVTNGLGTY	0.64/0.244054

	147	625	CVNYDLYGI	0.71/0.17380978
	148	355	VPSPLNWER	0.84/0.043685485
	149	1213	MSTCAVNYT	0.76/0.12337384
	150	182	HPNLGNHFK	0.23/0.64654865
	151	703	CNYVFNNSL	0.83/0.044655497
	152	1088	GRLTALNAY	0.82/0.053010075
	153	326	YTVQPIADV	0.65/0.22291911
	154	757	KNRRSRRAI	0.12/0.7488378
	155	142	SVVVQPRTI	0.99/-0.12375199
	156	519	TNYLTCDNL	0.00/0.86476576
	157	13	FAVIGDLNC	0.56/0.29687663
	158	239	TKFLFNVYL	0.24/0.61649385
	159	435	TTATSCQLY	0.98/-0.12369374
	160	795	IQIPSEFTI	0.40/0.44760283
	161	166	VSVCQYNMC	0.81/0.031736858
	162	690	NSSEPALLF	0.20/0.64170804
	163	1144	NAPYGLYFI	0.08/0.76023932
	164	460	TWNKRFGFI	0.46/0.37223574
	165	854	NELLDTTQL	0.95/-0.11790109
	166	737	AISVQTCDL	0.83/0.0016002471
	167	211	DVNATYLYF	0.04/0.79043859
	168	1194	YTGSGYYYP	0.70/0.12167664
	169	478	TGVFTNHSV	0.61/0.20905354
	170	338	KPDLPNCNI	0.04/0.77749754
	171	222	YQEGGTFYA	0.10/0.71632531
	172	491	HCFKAPKNF	0.94/-0.12924902
	173	1260	YINVTFLDL	0.07/0.73527251
	174	494	KAPKNFCPC	0.61/0.18369692
	175	64	NTTLFLNGY	0.29/0.50264266
	176	1250	TSVAPDLSL	0.71/0.08076331
	177	253	HYYVMPLTC	0.99/-0.20029993
	178	960	ENQISGYTL	0.42/0.36562494
	179	974	SLFPPWTAA	0.09/0.69513002
	180	208	FTYDVNATY	0.55/0.22922013
	181	1061	AISASLQEI	0.33/0.44906176
	182	406	DKFAIPNRR	0.85/-0.07174264
	183	276	TPLTPRQYL	0.00/0.77599705
	184	997	GLGVTMDVL	0.00/0.77527918
	185	1313	GVAMLVLLF	/0.76736083

	186	1282	LNHSYINLK	0.54/0.22670868
	187	1092	ALNAYVSQQ	0.85/-0.096656997
	188	962	QISGYTLAA	0.77/-0.020037817
	189	56	YVLDRVYLN	0.34/0.40926692
	190	1165	KVSPGLCIA	0.35/0.39918266
	191	1310	CLAGVAMLV	/0.74744067
	192	448	AANVSVSRF	0.04/0.70554959
	193	725	YLGCVVNAY	0.17/0.57212179
	194	678	CYSGRVSAA	0.03/0.7107723
	195	382	ADSFTCNNI	0.08/0.66009748
	196	1080	AQIDRLING	0.01/0.72729886
	197	973	ASLFPPWTA	0.00/0.73282348
	198	878	LKDGVNFNV	0.99/-0.25906731
	199	388	NNIDAAKIY	0.54/0.18869519
	200	787	EPVGGLYEI	0.17/0.55786069
	201	941	DLICVQSYK	0.14/0.58405131
	202	1240	EELDQWFKN	0.97/-0.25948147
	203	699	RNIKCNYVF	0.82/-0.11039526
	204	754	DYSKNRRSR	0.29/0.4176939
	205	306	EIKCKTQSI	0.52/0.18669072
	206	522	LTCDNLCTL	0.57/0.13631168
	207	1230	TSIPNLPDF	0.32/0.3855904
	208	996	NGLGVTMDV	0.62/0.079774929
	209	289	QDGIIFNAV	0.00/0.69829607
	210	778	TVNSVNDSL	0.00/0.69697261
	211	714	QLQPINYSF	0.59/0.10689114
	212	826	CGDYAACKL	0.82/-0.12324492
	213	66	TLFLNGYYP	0.70/-0.0035975393
	214	890	NFSPVLGCL	0.02/0.67492761
	215	397	GMCFSSITI	0.03/0.66154645
	216	1022	AIQQGFDAT	0.72/-0.029113002
	217	347	EAWLNDKSV	0.83/-0.14267198
	218	129	AITIGSTFV	0.12/0.55958619
	219	784	DSLEPVGGL	0.74/-0.060698413
	220	944	CVQSYKGIK	0.99/-0.31229481
	221	1098	SQQLSDSTL	0.80/-0.13068828
	222	623	GVCVNYDLY	0.05/0.61745976
	223	1158	PTKYVTAKV	0.85/-0.18294795

	224	365	TFSNCNFNM	0.19/0.47604251
	225	763	RAITTGYRF	0.00/0.66576004
	226	281	RQYLLAFNQ	0.88/-0.2160557
	227	972	SASLFPPWT	0.95/-0.28683702
	228	1293	GTYEYYVKW	/0.66268852
	229	828	DYAACKLQL	0.06/0.59971396
	230	1003	DVLSQNQKL	0.69/-0.0322844
	231	1104	STLVKFSAA	0.70/-0.044472735
	232	860	TQLQVANSL	0.01/0.6449722
	233	18	DLNCPLDPR	0.53/0.1165145
	234	158	NKLQGLLEV	0.14/0.50596177
	235	340	DLPNCNIEA	1.00/-0.35617448
	236	528	CTLDPITFK	0.07/0.57312374
	237	599	ILHDVNNGL	0.00/0.64300865
	238	668	ITNRTFMIH	0.01/0.63270915
	239	592	CNIFANFIL	0.53/0.10874037
	240	923	DVGFVEAYN	0.96/-0.32327137
	241	201	SCLYKRNFT	0.98/-0.34380037
	242	363	RKTFSNCNF	0.83/-0.19796853
	243	918	KVKLSDVGF	0.00/0.63123157
	244	771	FTNFEPFTV	0.16/0.4672843
	245	392	AAKIYGMCF	0.16/0.46608809
	246	817	VTIDCAAFV	0.14/0.48426328
	247	259	LTCISRRDI	0.90/-0.27793737
	248	766	TTGYRFTNF	0.07/0.54384314
	249	689	ANSSEPALL	0.62/-0.0077838697
	250	702	KCNYVFNNS	0.62/-0.0081875223
	251	380	IQADSFTCN	0.91/-0.29818823
	252	295	NAVDCMSDF	0.19/0.42143147
	253	291	GIIFNAVDC	0.93/-0.32119415
	254	1084	RLINGRLTA	0.14/0.46520304
	255	224	EGGTFYAYF	0.04/0.56507794
	256	802	TIGNMEEFI	0.60/0.0014550198
	257	911	IEDLLFDKV	0.70/-0.10481566
	258	1130	NFCGNGNHI	0.11/0.47477456
	259	843	CDNINAILT	0.93/-0.34822341
	260	932	NCTGGAEIR	0.88/-0.30516226

	261	87	GTDLLSTLW	0.99/-0.4160785
	**262**	**1143**	**QNAPYGLYF**	**0.00/0.57365265**
	263	1006	SQNQKLIAN	0.54/0.023150896
	264	935	GGAEIRDLI	0.19/0.37275014
	265	11	TAFAVIGDL	0.01/0.55076349
	266	1239	KEELDQWFK	0.96/-0.40040811
	267	92	STLWFKPPF	0.68/-0.12043133
	268	849	ILTEVNELL	0.03/0.52947398
	269	586	CLQGDKCNI	0.72/-0.16110256
	270	994	RINGLGVTM	0.08/0.47722697
	271	342	PNCNIEAWL	0.82/-0.26332217
	272	965	GYTLAATSA	1.00/-0.44381501
	273	866	NSLMNGVTL	0.09/0.465982
	274	260	TCISRRDIG	0.67/-0.11730873
	275	1072	RLDALEAEA	0.02/0.5316099
	276	437	ATSCQLYYN	0.87/-0.3211714
	277	424	GYLQSSNYR	0.01/0.53880786
	278	60	RVYLNTTLF	0.52/0.026021448
	279	432	RIDTTATSC	0.90/-0.35685537
	280	1272	MNRLQEAIK	0.00/0.541886
	281	612	DLQKANTEI	0.72/-0.18053657
	282	8	SLPTAFAVI	0.01/0.52927823
	283	1137	HIISLVQNA	0.10/0.43765557
	284	781	SVNDSLEPV	0.02/0.51138399
	285	671	RTFMIHSCY	0.56/-0.029309502
	286	206	RNFTYDVNA	0.72/-0.19035185
	287	864	VANSLMNGV	0.60/-0.072806232
	288	1113	QAMEKVNEC	0.63/-0.10346227
	289	261	CISRRDIGF	0.10/0.42390412
	290	20	NCPLDPRLK	1.00/-0.48350687
	291	591	KCNIFANFI	0.04/0.4709378
	292	619	EIELGVCVN	0.51/0.00065446274
	293	1058	RFGAISASL	0.77/-0.25970955
	294	1300	KWPWYVWLL	/0.50615282
	295	358	PLNWERKTF	0.89/-0.38479811
	296	979	WTAAAGVPF	0.02/0.483316
	297	329	QPIADVYRR	0.01/0.49214361
	298	1221	TKAPYVMLN	0.64/-0.13991298

	299	1004	VLSQNQKLI	0.52/-0.022422484
	300	156	GVNKLQGLL	0.01/0.48647109
	301	1271	EMNRLQEAI	0.00/0.49444084
	302	1055	LSNRFGAIS	0.84/-0.34586669
	303	412	NRRKVDLQL	0.04/0.4505505
	304	1056	SNRFGAISA	0.00/0.48756828
	305	514	TCPAGTNYL	0.00/0.48576885
	306	263	SRRDIGFTL	0.09/0.39504491
	307	351	NDKSVPSPL	0.88/-0.39511623
	308	476	QPTGVFTNH	0.04/0.44456138
	309	1151	FIHFNYVPT	0.76/-0.27616536
	310	855	ELLDTTQLQ	0.96/-0.47711623
	311	1096	YVSQQLSDS	0.98/-0.49812739
	312	931	NNCTGGAEI	0.06/0.42177788
	313	846	INAILTEVN	0.91/-0.42993354
	314	641	EVNATYYNS	0.63/-0.15279713
	315	681	GRVSAAYHA	0.00/0.46978696
	316	217	LYFHFYQEG	0.72/-0.25059039
	317	666	DYITNRTFM	0.00/0.46560219
	318	77	GSTYRNMAL	0.02/0.44499329
	319	1291	DIGTYEYYV	/0.46381712
	320	207	NFTYDVNAT	0.75/-0.28705922
	321	124	YSEFPAITI	0.03/0.43068298
	322	534	TFKAPDTYK	0.08/0.37780319
	323	1262	NVTFLDLQV	0.69/-0.23278799
	324	644	ATYYNSWQN	0.80/-0.34594176
	325	194	HLDTGVVSC	0.00/0.45387067
	326	588	QGDKCNIFA	1.00/-0.54648073
	327	674	MIHSCYSGR	0.00/0.44851207
	328	176	YPHTICHPN	0.69/-0.25099717
	329	368	NCNFNMSSL	0.00/0.43511016
	330	1017	NNALHAIQQ	0.65/-0.21524797
	331	616	ANTEIELGV	0.93/-0.50233594
	332	654	LYDSNGNLY	0.01/0.41692321
	333	202	CLYKRNFTY	0.02/0.40617812
	334	457	NPSTWNKRF	0.05/0.37604116
	335	1202	PEPITENNV	0.03/0.39578709
	336	758	NRRSRRAIT	0.03/0.39316024
	337	275	VTPLTPRQY	0.53/-0.1079905

	338	988	YLNVQYRIN	0.00/0.41799404
	339	216	YLYFHFYQE	0.56/-0.14373867
	340	244	NVYLGMALS	0.01/0.40391588
	341	1317	LVLLFFICC	/0.41201937
	342	1192	WMYTGSGYY	0.74/-0.33025172
	343	1082	IDRLINGRL	0.93/-0.52091464
	344	691	SSEPALLFR	0.02/0.38692398
	345	607	LTCSTDLQK	0.52/-0.11330045
	346	1112	AQAMEKVNE	0.93/-0.52470152
	347	453	VSRFNPSTW	0.01/0.39453873
	348	1316	MLVLLFFIC	/0.40448677
	349	712	TRQLQPINY	0.04/0.36290021
	350	196	DTGVVSCLY	0.98/-0.57715749
	351	1040	VVNANAEAL	0.01/0.39005797
	352	403	ITIDKFAIP	0.60/-0.20006665
	353	418	LQLGNLGYL	0.03/0.36814366
	354	269	FTLEYWVTP	0.84/-0.44348394
	355	605	NGLTCSTDL	0.93/-0.53401282
	356	1309	ICLAGVAML	/0.39466956
	357	48	VTNGLGTYY	0.03/0.36353205
	358	458	PSTWNKRFG	0.89/-0.497645
	359	111	KVKNTKVFK	0.01/0.38051629
	360	513	GTCPAGTNY	0.01/0.3764456
	361	992	QYRINGLGV	0.00/0.38442158
	362	1071	SRLDALEAE	0.59/-0.21367453
	363	686	AYHANSSEP	0.59/-0.21487044
	364	1101	LSDSTLVKF	0.69/-0.31845964
	365	1204	PITENNVVV	0.70/-0.33970453
	366	873	TLSTKLKDG	0.95/-0.5958323
	367	943	ICVQSYKGI	0.81/-0.46412512
	368	344	CNIEAWLND	0.58/-0.23931148
	369	1241	ELDQWFKNQ	0.98/-0.64667895
	370	1277	EAIKVLNHS	0.88/-0.55048873
	371	1134	NGNHIISLV	0.54/-0.21083952
	372	955	PPLLSENQI	0.65/-0.32494524
	373	680	SGRVSAAYH	0.55/-0.22501637
	374	852	EVNELLDTT	0.86/-0.54075608
	375	673	FMIHSCYSG	0.58/-0.26337873
	376	302	DFMSEIKCK	0.63/-0.33209923

	377	1135	GNHIISLVQ	0.80/-0.50584485
	378	413	RRKVDLQLG	0.71/-0.41620099
	379	516	PAGTNYLTC	0.92/-0.62977525
	380	304	MSEIKCKTQ	0.69/-0.40222402
	381	647	YNSWQNLLY	0.77/-0.49874867
	382	35	DTGPPSIST	0.84/-0.56895408
	383	858	DTTQLQVAN	0.96/-0.69962055
	384	825	VCGDYAACK	0.86/-0.59972232
	385	393	AKIYGMCFS	1.00/-0.75505759
	386	218	YFHFYQEGG	0.87/-0.63256838
	387	682	RVSAAYHAN	0.65/-0.41568367
	388	110	AKVKNTKVF	0.86/-0.6297661
	389	337	RKPDLPNCN	0.87/-0.64290963
	390	163	LLEVSVCQY	0.74/-0.51421165
	391	1211	VVMSTCAVN	0.57/-0.34500854
	392	597	NFILHDVNN	0.70/-0.48115938
	393	807	EEFIQTSSP	0.79/-0.57134192
	394	488	YAQHCFKAP	0.98/-0.76170797
	395	1167	SPGLCIAGN	0.89/-0.67335075
	396	909	SAIEDLLFD	0.53/-0.31535
	397	604	NNGLTCSTD	0.68/-0.47649818
	398	706	VFNNSLTRQ	0.99/-0.79064358
	399	1025	QGFDATNSA	0.97/-0.78151497
	400	1150	YFIHFNYVP	0.84/-0.65757591
	401	547	KSLVGIGEH	0.86/-0.67854085
	402	132	IGSTFVNTS	0.69/-0.51562853
	403	745	LTVGSGYCV	0.58/-0.41749306
	404	894	VLGCLGSEC	0.99/-0.82944512
	405	865	ANSLMNGVT	0.97/-0.82130289
	406	1060	GAISASLQE	0.97/-0.82240708
	407	743	CDLTVGSGY	0.64/-0.49756575
	408	19	LNCPLDPRL	0.60/-0.46049867
	409	5	LLISLPTAF	0.78/-0.64332399
	410	362	ERKTFSNCN	0.51/-0.37516632
	411	29	GSFNNRDTG	0.56/-0.43610297
	412	1176	RGIAPKSGY	0.83/-0.70812954
	413	274	WVTPLTPRQ	0.56/-0.44276648
	414	179	TICHPNLGN	0.94/-0.83615439
	415	183	PNLGNHFKE	0.82/-0.72560525

	416	1160	KYVTAKVSP	0.88/-0.78985969
	417	715	LQPINYSFD	0.70/-0.61148709
	418	768	GYRFTNFEP	0.81/-0.72203329
	419	305	SEIKCKTQS	0.81/-0.73675677
	420	343	NCNIEAWLN	0.78/-0.70741334
	421	834	LQLVEYGSF	0.59/-0.52017472
	422	603	VNNGLTCST	0.81/-0.74144409
	423	736	TAISVQTCD	0.90/-0.83421642
	424	602	DVNNGLTCS	0.65/-0.58624337
	425	1030	TNSALVKIQ	0.70/-0.6403108
	426	1232	IPNLPDFKE	0.71/-0.65284227
	427	185	LGNHFKELW	0.53/-0.49048956
	428	540	TYKCPQTKS	0.66/-0.634436
	429	999	GVTMDVLSQ	0.54/-0.51486845
	430	440	CQLYYNLPA	0.93/-0.90855461
	431	734	NSTAISVQT	0.75/-0.72984075
	432	160	LQGLLEVSV	0.54/-0.5299666
	433	370	NFNMSSLMS	0.52/-0.51276669
	434	733	YNSTAISVQ	0.83/-0.82465938
	435	303	FMSEIKCKT	0.64/-0.64391806
	436	792	LYEIQIPSE	0.92/-0.92620775
	437	936	GAEIRDLIC	0.52/-0.53052956
	438	1252	VAPDLSLDY	0.72/-0.73208717
	439	170	QYNMCEYPH	0.52/-0.53476248
	440	290	DGIIFNAVD	0.88/-0.89752012
	441	661	LYGFRDYIT	0.60/-0.63273785
	442	1102	SDSTLVKFS	0.96/-0.99682303
	443	1249	QTSVAPDLS	0.93/-0.9701338
	444	731	NAYNSTAIS	0.72/-0.76168058
	445	915	LFDKVKLSD	0.91/-0.95737381
	446	76	SGSTYRNMA	0.52/-0.56749591
	447	800	EFTIGNMEE	0.74/-0.78988985
	448	84	ALKGTDLLS	0.62/-0.67112019
	449	1237	DFKEELDQW	0.99/-1.0653404
	450	515	CPAGTNYLT	0.61/-0.68654272
	451	143	VVVQPRTIN	0.92/-1.0031782
	452	830	AACKLQLVE	0.99/-1.1013187
	453	1118	VNECVKSQS	0.74/-0.85144344
	454	1129	INFCGNGNH	0.66/-0.77528715

	455	271	LEYWVTPLT	0.95/-1.0843846
	456	626	VNYDLYGIS	0.88/-1.0144346
	457	439	SCQLYYNLP	0.96/-1.1044019
	458	126	EFPAITIGS	0.98/-1.1310392
	459	164	LEVSVCQYN	0.52/-0.68212951
	460	423	LGYLQSSNY	0.94/-1.1135489
	461	379	FIQADSFTC	0.85/-1.0255097
	462	738	ISVQTCDLT	0.69/-0.8835284
	463	655	YDSNGNLYG	0.57/-0.76773168
	464	624	VCVNYDLYG	0.90/-1.0994467
	465	90	LLSTLWFKP	0.97/-1.1744841
	466	294	FNAVDCMSD	0.63/-0.83843424
	467	1023	IQQGFDATN	0.58/-0.79554506
	468	1138	IISLVQNAP	0.64/-0.85762903
	469	899	GSECSKASS	0.70/-0.94197746
	470	414	RKVDLQLGN	0.64/-0.8833033
	471	297	VDCMSDFMS	0.95/-1.199791
	472	63	LNTTLFLNG	0.58/-0.83857498
	473	582	SADSCLQGD	0.74/-1.0009609
	474	433	IDTTATSCQ	0.97/-1.2312266
	475	629	DLYGISGQG	0.95/-1.2499934
	476	585	SCLQGDKCN	0.93/-1.2617111
	477	94	LWFKPPFLS	0.73/-1.0640988
	478	1275	LQEAIKVLN	0.85/-1.1890652
	479	687	YHANSSEPA	0.99/-1.3494791
	480	23	LDPRLKGSF	0.65/-1.0288432
	481	192	LWHLDTGVV	0.63/-1.0241095
	482	272	EYWVTPLTP	0.61/-1.0087404
	483	815	PKVTIDCAA	0.96/-1.3703482
	484	173	MCEYPHTIC	0.54/-0.97772655
	485	1283	NHSYINLKD	0.79/-1.2538952
	486	898	LGSECSKAS	0.97/-1.459627
	487	893	PVLGCLGSE	0.79/-1.2837736
	488	1016	FNNALHAIQ	0.62/-1.1205932
	489	1247	KNQTSVAPD	0.86/-1.4031885
	490	966	YTLAATSAS	0.74/-1.3078469
	491	376	LMSFIQADS	0.68/-1.2753651
	492	649	SWQNLLYDS	0.79/-1.3856862
	493	317	PTGVYELNG	0.64/-1.2363928

	494	853	VNELLDTTQ	0.91/-1.5430266
	495	794	EIQIPSEFT	0.60/-1.2380885
	496	147	PRTINSTQD	0.66/-1.3513955
	497	17	GDLNCPLDP	0.98/-1.6959279
	498	874	LSTKLKDGV	0.76/-1.4797166
	499	652	NLLYDSNGN	0.65/-1.3889788
	500	1052	LQQLSNRFG	0.57/-1.3478396
	501	1226	VMLNTSIPN	0.56/-1.3608976
	502	85	LKGTDLLST	0.98/-1.8358649
	503	46	VDVTNGLGT	0.68/-1.6244871
**HCoV-HKU1**				
**Viral protein name**	**Rank**	**Start position**	**Sequence**	**Score ANN/SVM**
**Spike**	1	700	SYVLNNISL	0.82/1.7620527
	2	1230	KLSDFESEL	0.73/1.5428952
	3	43	SYGLGTYYI	0.96/1.2092847
	4	1043	ALNSLLQQL	0.62/1.4388165
	5	1085	RLTALNAYV	1.00/1.0179382
	6	272	NRGVITNAV	0.99/1.0100408
	7	493	ASCPIGTNY	0.98/0.98864166
	8	752	SSSSRRKRR	0.99/0.93621214
	9	69	KSGANFRDL	0.29/1.6294666
	10	421	SSCQLYYSL	0.88/1.0333743
	11	1279	SLNNSYINL	0.65/1.2588282
	12	105	RVKNTKLYV	0.88/1.0142424
	13	1046	SLLQQLFNK	0.97/0.91980637
	14	1216	FTKAPLVYL	0.85/1.0336339
	15	426	YYSLPAINV	0.67/1.1909905
	16	710	TQPYFDSYL	0.97/0.88413644
	17	759	RRSISASYR	0.94/0.8998011
	18	316	TVHRRIPDL	0.97/0.82574329
	19	586	NRCNIFSNF	0.99/0.7827496
	20	1140	NAPYGLLFM	0.98/0.7818804
	21	828	ACHDLLSEY	0.78/0.968353
	22	1175	APKQGYFIK	0.93/0.81055523
	23	1119	KSQSPRINF	0.98/0.74189283
	24	993	GLGVTMDVL	0.88/0.77527918
	25	963	TTAATVAAM	0.99/0.63779509
	26	687	ASSLALLYR	0.91/0.71152114

	27	190	TYNVSTDWL	0.51/1.0546429
	28	583	VSNNRCNIF	0.90/0.65970885
	29	760	RSISASYRF	0.92/0.63541909
	30	719	GCVFNADNL	0.88/0.63192236
	31	298	LPNTGVYDL	0.56/0.92549997
	32	1258	TINATFLDL	0.35/1.1252987
	33	608	DLLQPNTEV	0.26/1.2138412
	34	1025	ATNSALAKI	0.91/0.54701037
	35	116	TLYSEFSTI	0.96/0.49510647
	36	756	RRKRRSISA	0.65/0.79842193
	37	659	GFKDFVTNK	0.03/1.414362
	38	41	DVSYGLGTY	0.86/0.5842947
	39	102	IFSRVKNTK	0.64/0.78915923
	40	20	CTNFAINDK	0.75/0.67871498
	41	494	SCPIGTNYR	0.99/0.43613207
	42	689	SLALLYRNL	0.10/1.3084838
	43	66	YFPKSGANF	0.90/0.48607673
	44	1248	IAPNLTLNL	0.87/0.49571062
	45	770	TFEPFNVSF	0.71/0.64479112
	46	1282	NSYINLKDI	0.96/0.36903748
	47	557	GVLDGSYNV	0.86/0.46236517
	48	903	SRSFFEDLL	0.44/0.86461849
	49	616	VFTDVCVDY	1.00/0.30138995
	50	984	SLNVQYRIN	0.96/0.33455348
	51	902	SSRSFFEDL	0.58/0.71380652
	52	668	TYNIFPCYA	0.15/1.1424939
	53	1076	VQIDRLING	0.98/0.30891106
	54	1277	IKSLNNSYI	0.99/0.29657575
	55	203	YQERGTFYA	0.92/0.35979012
	56	754	SSRRKRRSI	0.29/0.97791422
	57	1276	SIKSLNNSY	1.00/0.25899736
	58	716	SYLGCVFNA	0.46/0.79580845
	59	670	NIFPCYAGR	0.86/0.39556392
	60	224	FSLYLGTLL	0.90/0.34920887
	61	380	GSCFKSIVL	0.52/0.72519269
	62	698	KCSYVLNNI	0.09/1.1537617
	63	1262	TFLDLYYEM	0.08/1.1465407
	64	1029	ALAKIQSVV	0.99/0.2335248
	65	824	SNYAACHDL	0.67/0.55271113

	66	1039	SNAQALNSL	0.53/0.69256061
	67	663	FVTNKTYNI	0.92/0.29439927
	68	1213	SVNFTKAPL	0.65/0.56402523
	69	34	RISEYVVDV	0.60/0.61107435
	70	31	TVPRISEYV	0.84/0.36983368
	71	123	TIVIGSVFI	0.75/0.45831722
	72	121	FSTIVIGSV	0.89/0.31395181
	73	50	YILDRVYLN	0.88/0.32378538
	74	520	CLPDPITAY	0.94/0.24984492
	75	1068	RLDALEAQV	0.16/1.0275264
	76	1106	GAALAMEKV	0.97/0.2122842
	77	694	YRNLKCSYV	0.03/1.1489538
	78	1036	VVNSNAQAL	0.55/0.62669954
	79	44	YGLGTYYIL	0.51/0.66332393
	80	587	RCNIFSNFI	0.57/0.59998654
	81	168	SSRNESWHF	0.94/0.22365044
	82	940	CVQSFNGIK	0.97/0.18017776
	83	1310	SFIIFLVLL	/1.1462725
	84	799	TIVGQEEFI	0.99/0.14878547
	85	336	FNVPSPLNW	0.87/0.26876333
	86	684	HQNASSLAL	0.60/0.52976512
	87	1280	LNNSYINLK	0.98/0.14938808
	88	1173	GIAPKQGYF	0.89/0.23258714
	89	768	FVTFEPFNV	0.74/0.38140978
	90	667	KTYNIFPCY	0.96/0.16056305
	91	167	GSSRNESWH	0.67/0.44543268
	92	664	VTNKTYNIF	0.65/0.46142895
	93	154	TMCEYPHTI	0.88/0.22894775
	94	259	TPLSKRQYL	0.69/0.41730156
	95	969	AAMFPPWSA	0.96/0.14246828
	96	566	SCLCSTDAF	0.78/0.32080457
	97	98	FNNGIFSRV	0.50/0.59960068
	98	763	SASYRFVTF	0.42/0.66799322
	99	1081	LINGRLTAL	0.08/1.0044934
	100	216	SGMPTTFLF	0.87/0.21339683
	101	30	TTVPRISEY	0.49/0.59254183
	102	638	VSAVYYNSW	0.76/0.31132417
	103	749	SPSSSSSRR	0.98/0.086673944
	104	1113	KVNECVKSQ	0.89/0.16973449

	105	1061	SLQEILSRL	0.00/1.0593795
	106	933	SEIRDLLCV	0.97/0.088650187
	107	55	VYLNTTILF	0.94/0.11184547
	108	1008	IATAFNNAL	0.86/0.18873703
	109	1120	SQSPRINFC	0.71/0.33327033
	110	512	HTDWCRCSC	0.76/0.27946536
	111	713	YFDSYLGCV	0.99/0.047453498
	112	623	DYDLYGITG	0.93/0.10714629
	113	271	DNRGVITNA	0.38/0.6541886
	114	1309	FSFIIFLVL	/1.02016
	115	1181	FIKHNDHWM	0.34/0.67452595
	116	456	SSHSVVYSR	0.16/0.8521888
	117	438	NYNPSSWNR	0.01/1.0021574
	118	269	KFDNRGVIT	0.92/0.091904369
	119	825	NYAACHDLL	0.92/0.090832085
	120	766	YRFVTFEPF	0.65/0.35859663
	121	838	TFCDNINSI	0.01/0.99608652
	122	857	TQLHVADTL	0.14/0.85599145
	123	706	ISLATQPYF	0.51/0.48360961
	124	835	EYGTFCDNI	0.00/0.98854271
	**125**	**1296**	**YVKWPWYVW**	**/0.9882908**
	126	67	FPKSGANFR	0.00/0.98805195
	127	229	GTLLSHYYV	0.38/0.60497684
	128	282	CSSSFFSEI	0.37/0.61421477
	129	83	TYLSTLWYQ	0.92/0.057406196
	130	445	NRRYGFNNF	0.00/0.97131068
	131	758	KRRSISASY	0.46/0.50867717
	132	941	VQSFNGIKV	0.59/0.3746817
	133	99	NNGIFSRVK	0.46/0.5046264
	134	6	FILPTTLAV	0.12/0.8440887
	135	771	FEPFNVSFV	0.00/0.96360683
	136	1295	MYVKWPWYV	/0.96271032
	137	982	PFSLNVQYR	0.70/0.25872781
	138	351	NCNFNLSTL	0.08/0.87856975
	139	1220	PLVYLNHSV	0.99/-0.032345436
	140	793	KIPTNFTIV	0.23/0.72663501
	141	942	QSFNGIKVL	0.83/0.12580053
	142	927	NNCTGGSEI	0.86/0.090929945
	143	192	NVSTDWLYF	0.56/0.39015094

	144	195	TDWLYFHFY	0.98/-0.032061155
	145	790	YEIKIPTNF	0.77/0.17685198
	146	1101	SLVKFGAAL	0.04/0.90519989
	147	1084	GRLTALNAY	0.89/0.053010075
	148	218	MPTTFLFSL	0.30/0.64085994
	149	1217	TKAPLVYLN	0.90/0.039178237
	150	53	DRVYLNTTI	0.99/-0.052404722
	151	784	ESVGGLYEI	0.61/0.32512498
	152	582	CVSNNRCNI	0.01/0.92421002
	153	775	NVSFVNDSI	0.27/0.66251473
	154	896	GPHCGSSSR	0.75/0.18243433
	155	641	VYYNSWQNL	0.01/0.91599745
	156	15	IGDFNCTNF	0.57/0.35013327
	157	344	WERKIFSNC	0.81/0.10647642
	158	179	SEPLCLFKK	0.93/-0.013901221
	159	649	LLYDSNGNI	0.73/0.18122959
	160	447	RYGFNNFNL	0.02/0.89119812
	161	755	SRRKRRSIS	0.89/0.017578754
	162	76	DLSLKGTTY	0.67/0.23461964
	163	880	HFDVDNINF	0.69/0.21297986
	164	1102	LVKFGAALA	0.63/0.26450716
	165	1246	TSIAPNLTL	0.66/0.22695478
	166	839	FCDNINSIL	0.87/0.014076705
	167	1254	LNLHTINAT	0.95/-0.070832522
	168	821	FVCSNYAAC	0.00/0.87835355
	169	1223	YLNHSVPKL	0.00/0.87416547
	170	261	LSKRQYLLK	0.40/0.47387658
	171	731	SVSSCALRM	0.06/0.81062214
	172	262	SKRQYLLKF	0.97/-0.1017003
	173	311	VKPVATVHR	0.99/-0.13332111
	174	379	YGSCFKSIV	0.12/0.73650034
	175	478	KPSFASSCK	0.85/0.0063641689
	176	858	QLHVADTLM	0.67/0.18622172
	177	1023	FSATNSALA	0.92/-0.066135006
	178	470	NNTFCPCAK	0.67/0.18375089
	179	1133	HILSLVQNA	0.38/0.46644373
	180	979	AGIPFSLNV	0.59/0.25530636
	181	674	CYAGRVSAA	0.28/0.5643572
	182	792	IKIPTNFTI	0.79/0.052412036

	183	204	QERGTFYAY	0.31/0.52384156
	184	90	YQKPFLSDF	0.00/0.83050707
	185	921	GFVEAYNNC	0.72/0.11041821
	186	452	NFNLSSHSV	0.65/0.17807557
	187	82	TTYLSTLWY	0.96/-0.13333662
	188	810	NSPKVTIDC	0.92/-0.097998412
	189	32	VPRISEYVV	0.91/-0.089489515
	190	878	NLHFDVDNI	0.00/0.81835696
	191	205	ERGTFYAYY	0.19/0.62816863
	192	213	YADSGMPTT	0.79/0.026366717
	193	126	IGSVFINNS	0.99/-0.17778451
	194	1301	WYVWLLISF	/0.81108318
	195	682	AFHQNASSL	0.23/0.5762915
	196	323	DLPDCDIDK	0.55/0.24927616
	197	375	ESKIYGSCF	0.23/0.56911828
	198	442	SSWNRRYGF	0.13/0.66621604
	199	468	SVNNTFCPC	0.69/0.10515734
	200	1018	SIQNGFSAT	0.94/-0.14581676
	201	424	QLYYSLPAI	0.91/-0.11824045
	202	715	DSYLGCVFN	0.89/-0.1002855
	203	1307	ISFSFIIFL	/0.78575864
	204	870	TLSSNLNTN	0.53/0.25374782
	205	975	WSAAAGIPF	0.42/0.36285401
	206	802	GQEEFIQTN	0.95/-0.16816374
	207	1117	CVKSQSPRI	0.24/0.53208529
	208	1049	QQLFNKFGA	1.00/-0.22821043
	209	983	FSLNVQYRI	0.00/0.77109989
	210	553	EEKCGVLDG	0.94/-0.16928054
	211	842	NINSILDEV	0.76/0.0093573244
	212	355	NLSTLLRLV	0.21/0.55837403
	213	296	SLLPNTGVY	0.79/-0.022870965
	214	774	FNVSFVNDS	0.99/-0.22480162
	215	268	LKFDNRGVI	0.96/-0.19895001
	216	319	RRIPDLPDC	0.95/-0.19023402
	217	1199	EPISDKNVV	0.98/-0.22196621
	218	621	CVDYDLYGI	0.98/-0.22576904
	219	1255	NLHTINATF	0.02/0.7275797
	220	990	RINGLGVTM	0.27/0.47722697
	221	609	LLQPNTEVF	0.92/-0.17327496

	222	431	AINVTINNY	0.01/0.73662746
	223	446	RRYGFNNFN	0.95/-0.20542774
	224	764	ASYRFVTFE	0.71/0.034522759
	225	1344	GHHDFVIKT	/0.73421119
	226	914	KVKLSDVGF	0.10/0.63123157
	227	84	YLSTLWYQK	0.10/0.62805235
	228	283	SSSFFSEIQ	0.97/-0.24306024
	229	250	DNETLQYWV	0.99/-0.2639652
	230	569	CSTDAFLGW	0.36/0.36553576
	231	148	ITACQYTMC	0.66/0.062803268
	232	230	TLLSHYYVL	0.03/0.69149378
	233	367	SFSCNNFDE	0.95/-0.23018078
	234	24	AINDKNTTV	0.00/0.71801456
	235	1136	SLVQNAPYG	0.51/0.20785211
	236	968	VAAMFPPWS	0.91/-0.19743027
	237	584	SNNRCNIFS	0.70/0.010838591
	238	1153	KPISFKTVL	0.14/0.56974466
	239	220	TTFLFSLYL	0.12/0.58921455
	240	1235	ESELSHWFK	0.87/-0.16129879
	241	591	FSNFILNGI	0.03/0.67748901
	242	831	DLLSEYGTF	0.53/0.17644984
	243	1126	NFCGNGNHI	0.23/0.47477456
	244	1094	SQQLSDISL	0.34/0.36361471
	245	476	CAKPSFASS	0.93/-0.2329537
	246	912	FDKVKLSDV	0.71/-0.013587218
	247	696	NLKCSYVLN	0.78/-0.084990188
	248	378	IYGSCFKSI	0.26/0.43265229
	249	778	FVNDSIESV	0.02/0.67162651
	250	1211	TCSVNFTKA	0.95/-0.26021539
	251	303	VYDLSGFTV	0.05/0.63793452
	252	71	GANFRDLSL	0.10/0.58244558
	253	661	KDFVTNKTY	0.96/-0.27860918
	254	861	VADTLMQGV	0.89/-0.21024097
	255	1313	IFLVLLFFI	/0.67886396
	256	845	SILDEVNGL	0.01/0.66860581
	257	279	AVDCSSSFF	0.01/0.66734623
	258	393	IPNSRRSDL	0.07/0.60429302
	259	94	FLSDFNNGI	0.00/0.67069999
	260	181	PLCLFKKNF	0.85/-0.17951239

	261	109	TKLYVNKTL	0.76/-0.089771842
	262	371	NNFDESKIY	0.54/0.12834157
	263	309	FTVKPVATV	0.05/0.61695832
	264	617	FTDVCVDYD	0.95/-0.28306966
	265	736	ALRMGSGFC	0.84/-0.17342683
	266	97	DFNNGIFSR	0.23/0.42996947
	267	574	FLGWSYDTC	0.67/-0.010117306
	268	1145	LLFMHFSYK	0.14/0.51861318
	269	901	SSSRSFFED	0.84/-0.18454701
	270	436	INNYNPSSW	0.67/-0.019908136
	271	383	FKSIVLDKF	0.90/-0.25461354
	272	310	TVKPVATVH	0.62/0.025081417
	273	386	IVLDKFAIP	0.96/-0.31530854
	274	818	CSLFVCSNY	0.09/0.55363702
	275	1251	NLTLNLHTI	0.01/0.63317409
	276	265	QYLLKFDNR	0.00/0.64215101
	277	637	EVSAVYYNS	0.73/-0.090024514
	278	95	LSDFNNGIF	0.99/-0.35154806
	279	461	VYSRYCFSV	0.00/0.63844054
	280	613	NTEVFTDVC	0.93/-0.29226743
	281	345	ERKIFSNCN	0.88/-0.24336764
	282	737	LRMGSGFCV	0.85/-0.21364811
	283	1096	QLSDISLVK	0.07/0.56491084
	284	1278	KSLNNSYIN	0.93/-0.29574406
	285	1009	ATAFNNALL	0.02/0.61373012
	286	642	YYNSWQNLL	0.00/0.6301557
	287	1201	ISDKNVVFM	0.00/0.62784627
	288	244	AISSNTDNE	0.98/-0.35224845
	289	1064	EILSRLDAL	0.01/0.61409618
	290	680	SAAFHQNAS	0.92/-0.29897792
	291	1195	YYYPEPISD	1.00/-0.37990603
	292	49	YYILDRVYL	0.00/0.61815747
	293	761	SISASYRFV	0.03/0.58708429
	294	628	GITGQGIFK	0.64/-0.025004151
	295	249	TDNETLQYW	0.68/-0.06721555
	296	705	NISLATQPY	0.55/0.062014224
	297	407	GFLQSSNYK	0.03/0.57213049
	298	312	KPVATVHRR	0.02/0.57630943
	299	513	TDWCRCSCL	0.17/0.42197058

	300	1289	DIGTYEMYV	/0.59184516
	301	194	STDWLYFHF	0.01/0.58049051
	302	707	SLATQPYFD	0.92/-0.33140434
	303	612	PNTEVFTDV	0.85/-0.26779246
	**304**	**1139**	**QNAPYGLLF**	**0.10/0.48203718**
	305	22	NFAINDKNT	0.86/-0.29060754
	306	676	AGRVSAAFH	0.99/-0.42362248
	307	619	DVCVDYDLY	0.84/-0.27545188
	308	354	FNLSTLLRL	0.01/0.55416564
	309	420	SSSCQLYYS	0.91/-0.34786364
	310	428	SLPAINVTI	0.11/0.4508478
	311	846	ILDEVNGLL	0.13/0.43043316
	312	1312	IIFLVLLFF	/0.55391827
	313	237	VLPLTCNAI	0.17/0.38390309
	314	1169	SGDVGIAPK	0.58/-0.026446645
	315	212	YYADSGMPT	0.76/-0.20649614
	316	823	CSNYAACHD	0.63/-0.076609213
	317	956	ESQISGYTT	0.98/-0.43115406
	318	104	SRVKNTKLY	0.94/-0.39415576
	319	1249	APNLTLNLH	0.10/0.44537961
	320	108	NTKLYVNKT	0.76/-0.21692109
	321	92	KPFLSDFNN	0.60/-0.057320616
	322	978	AAGIPFSLN	0.70/-0.15877418
	323	1334	KCHNCCDEY	/0.53725745
	324	170	RNESWHFDK	0.87/-0.33295051
	325	1027	NSALAKIQS	0.92/-0.3902425
	326	1304	WLLISFSFI	/0.52219583
	327	1007	LIATAFNNA	0.51/0.0078548036
	328	573	AFLGWSYDT	0.83/-0.31303054
	329	285	SFFSEIQCK	0.87/-0.35358949
	330	967	TVAAMFPPW	0.53/-0.017996401
	331	656	NIIGFKDFV	0.72/-0.20812647
	332	1035	SVVNSNAQA	0.64/-0.1282722
	333	80	KGTTYLSTL	0.66/-0.14924444
	334	1109	LAMEKVNEC	0.74/-0.22937302
	335	1298	KWPWYVWLL	/0.50615282
	336	686	NASSLALLY	0.52/-0.016611144
	337	1013	NNALLSIQN	0.98/-0.47714226

	338	110	KLYVNKTLY	0.51/-0.0086607937
	339	63	FTGYFPKSG	0.87/-0.37039924
	340	929	CTGGSEIRD	0.94/-0.44560625
	341	567	CLCSTDAFL	0.00/0.48113686
	342	765	SYRFVTFEP	0.90/-0.42077695
	343	113	VNKTLYSEF	0.56/-0.084744278
	344	1080	RLINGRLTA	0.01/0.46520304
	345	1091	AYVSQQLSD	0.57/-0.096254408
	346	1306	LISFSFIIF	/0.47340461
	347	87	TLWYQKPFL	0.00/0.47070531
	348	1058	ISSSLQEIL	0.06/0.40879305
	349	208	TFYAYYADS	0.91/-0.44478649
	350	1047	LLQQLFNKF	0.56/-0.096387161
	351	729	DYSVSSCAL	0.01/0.45349998
	352	643	YNSWQNLLY	0.96/-0.49874867
	353	370	CNNFDESKI	0.06/0.39783651
	354	278	NAVDCSSSF	0.00/0.45743779
	355	1070	DALEAQVQI	0.00/0.45586622
	356	56	YLNTTILFT	0.96/-0.51013712
	357	815	TIDCSLFVC	0.84/-0.3902094
	358	919	DVGFVEAYN	0.77/-0.32327137
	359	881	FDVDNINFK	0.01/0.4343559
	360	137	IVVQPHNGV	0.01/0.43382035
	361	1160	VLVSPGLCI	0.60/-0.16010756
	362	1244	NQTSIAPNL	0.00/0.42928267
	363	369	SCNNFDESK	0.72/-0.29175091
	364	1311	FIIFLVLLF	/0.4254223
	365	234	HYYVLPLTC	0.95/-0.53184335
	366	995	GVTMDVLNK	0.05/0.36752088
	367	1315	LVLLFFICC	/0.41201937
	368	301	TGVYDLSGF	0.99/-0.58161554
	369	1011	AFNNALLSI	0.00/0.40789941
	370	136	TIVVQPHNG	0.87/-0.47087208
	371	14	VIGDFNCTN	0.86/-0.46146459
	372	358	TLLRLVHTD	0.00/0.39603874
	373	1340	DEYGGHHDF	/0.39453733
	374	646	WQNLLYDSN	0.89/-0.49568818
	375	140	QPHNGVLEI	0.03/0.36182254

	376	191	YNVSTDWLY	0.67/-0.28154727
	377	593	NFILNGINS	0.01/0.37841412
	378	988	QYRINGLGV	0.00/0.38442158
	379	1266	LYYEMNLIQ	0.99/-0.60719617
	380	1274	QESIKSLNN	0.66/-0.27918132
	381	1168	ISGDVGIAP	0.54/-0.16140511
	382	225	SLYLGTLLS	0.74/-0.36583862
	383	1291	GTYEMYVKW	/0.37300047
	384	1284	YINLKDIGT	0.81/-0.43769982
	385	117	LYSEFSTIV	0.00/0.37138757
	386	246	SSNTDNETL	0.00/0.3675537
	387	945	NGIKVLPPI	0.55/-0.1831929
	388	808	QTNSPKVTI	0.00/0.36370318
	389	1308	SFSFIIFLV	/0.36348684
	390	1203	DKNVVFMNT	0.68/-0.32249778
	391	373	FDESKIYGS	0.82/-0.46546892
	392	1104	KFGAALAME	0.98/-0.62602726
	393	757	RKRRSISAS	0.76/-0.41062382
	394	1034	QSVVNSNAQ	0.85/-0.50250075
	395	28	KNTTVPRIS	0.84/-0.49389475
	396	669	YNIFPCYAG	0.52/-0.17993155
	397	466	CFSVNNTFC	0.59/-0.25503
	398	973	PPWSAAAGI	0.86/-0.53075691
	399	253	TLQYWVTPL	0.86/-0.54234401
	400	703	LNNISLATQ	1.00/-0.69561064
	401	1261	ATFLDLYYE	0.68/-0.37705261
	402	867	QGVTLSSNL	0.52/-0.21802335
	403	396	SRRSDLQLG	0.65/-0.35071912
	404	961	GYTTAATVA	0.84/-0.54999436
	405	856	TTQLHVADT	0.87/-0.58132348
	406	500	NYRSCESTT	0.64/-0.374593
	407	36	SEYVVDVSY	0.54/-0.27510673
	408	644	NSWQNLLYD	0.61/-0.35769978
	409	175	HFDKSEPLC	0.70/-0.45435513
	410	5	IFILPTTLA	0.92/-0.67899069
	411	1033	IQSVVNSNA	0.62/-0.38184421
	412	805	EFIQTNSPK	0.90/-0.66261155
	413	164	KSKGSSRNE	0.55/-0.32326481
	414	89	WYQKPFLSD	0.66/-0.44014453

	415	955	SESQISGYT	0.92/-0.70246871
	416	96	SDFNNGIFS	0.77/-0.57073891
	417	60	TILFTGYFP	0.86/-0.66235025
	418	600	NSGTTCSND	0.54/-0.34397566
	419	177	DKSEPLCLF	0.56/-0.36458908
	420	1044	LNSLLQQLF	0.75/-0.56321456
	421	26	NDKNTTVPR	0.56/-0.38498303
	422	276	ITNAVDCSS	0.96/-0.79727375
	423	741	SGFCVDYNS	0.96/-0.80289721
	424	965	AATVAAMFP	0.96/-0.80290496
	425	403	LGSSGFLQS	0.95/-0.79772719
	426	480	SFASSCKSH	0.52/-0.37536045
	427	267	LLKFDNRGV	0.84/-0.70074456
	428	335	NFNVPSPLN	0.80/-0.66989397
	429	1031	AKIQSVVNS	0.57/-0.44119322
	430	620	VCVDYDLYG	0.92/-0.79279366
	431	742	GFCVDYNSP	0.70/-0.58838526
	432	657	IIGFKDFVT	0.86/-0.74926241
	433	746	DYNSPSSSS	0.86/-0.75441479
	434	827	AACHDLLSE	0.73/-0.62930206
	435	1221	LVYLNHSVP	0.78/-0.67958717
	436	934	EIRDLLCVQ	0.99/-0.89398253
	437	221	TFLFSLYLG	0.69/-0.60269387
	438	1193	SSYYYPEPI	0.59/-0.50616665
	439	892	VGCLGPHCG	0.72/-0.64089218
	440	397	RRSDLQLGS	0.60/-0.527383
	441	280	VDCSSSFFS	0.85/-0.78407158
	442	606	SNDLLQPNT	0.72/-0.65751758
	443	333	LNNFNVPSP	0.94/-0.89129025
	444	1132	NHILSLVQN	0.78/-0.74707211
	445	882	DVDNINFKS	0.79/-0.7575608
	446	776	VSFVNDSIE	0.85/-0.82012771
	447	392	AIPNSRRSD	0.81/-0.78256021
	448	734	SCALRMGSG	0.51/-0.49183573
	449	672	FPCYAGRVS	0.51/-0.5027555
	450	1191	TGSSYYYPE	0.72/-0.72422561
	451	517	RCSCLPDPI	0.83/-0.83569933
	452	326	DCDIDKWLN	0.74/-0.7476457
	453	789	LYEIKIPTN	0.62/-0.63294201

	454	811	SPKVTIDCS	0.88/-0.89853357
	455	62	LFTGYFPKS	0.63/-0.65634241
	456	172	ESWHFDKSE	0.83/-0.85967749
	457	647	QNLLYDSNG	0.62/-0.6549701
	458	401	LQLGSSGFL	0.99/-1.0250406
	459	539	LVGVGEHCA	0.62/-0.66037379
	460	256	YWVTPLSKR	0.54/-0.58719918
	461	1075	QVQIDRLIN	0.71/-0.75773694
	462	1090	NAYVSQQLS	0.67/-0.72905555
	463	1163	SPGLCISGD	0.83/-0.89250164
	464	1228	VPKLSDFES	0.61/-0.68824535
	465	495	CPIGTNYRS	0.56/-0.65088614
	466	1170	GDVGIAPKQ	0.90/-0.99249942
	467	258	VTPLSKRQY	0.53/-0.6315164
	468	423	CQLYYSLPA	0.85/-0.960454
	469	565	VSCLCSTDA	0.58/-0.69245581
	470	563	YNVSCLCST	0.68/-0.79259981
	471	361	RLVHTDSFS	0.53/-0.65251529
	472	381	SCFKSIVLD	0.62/-0.74942087
	473	807	IQTNSPKVT	0.99/-1.1300118
	474	683	FHQNASSLA	0.93/-1.0713918
	475	1283	SYINLKDIG	0.72/-0.86827363
	476	1086	LTALNAYVS	0.76/-0.90941691
	477	551	VDEEKCGVL	0.64/-0.81204896
	478	1260	NATFLDLYY	0.53/-0.72353102
	479	1148	MHFSYKPIS	0.90/-1.094533
	480	467	FSVNNTFCP	0.68/-0.88764661
	481	1135	LSLVQNAPY	0.87/-1.0784129
	482	434	VTINNYNPS	0.79/-1.0007724
	483	1005	QKLIATAFN	0.92/-1.1477793
	484	1257	HTINATFLD	0.70/-0.92788208
	485	491	PSASCPIGT	0.54/-0.78325459
	486	288	SEIQCKTKS	0.97/-1.2177181
	487	1016	LLSIQNGFS	0.79/-1.0416696
	488	708	LATQPYFDS	0.95/-1.2061844
	489	559	LDGSYNVSC	0.63/-0.88731451
	490	233	SHYYVLPLT	0.73/-0.98840856
	491	599	INSGTTCSN	0.51/-0.77381362
	492	1198	PEPISDKNV	0.53/-0.79441812

	493	300	NTGVYDLSG	0.81/-1.0778341
	494	1271	NLIQESIKS	0.55/-0.83062557
	495	484	SCKSHKPPS	0.94/-1.2230892
	496	155	MCEYPHTIC	0.68/-0.97772655
	497	1243	KNQTSIAPN	0.95/-1.2651821
	498	356	LSTLLRLVH	0.97/-1.2914599
	499	745	VDYNSPSSS	0.81/-1.1406725
	500	254	LQYWVTPLS	0.90/-1.2358166
	501	888	FKSLVGCLG	0.67/-1.0074895
	502	518	CSCLPDPIT	0.73/-1.0764705
	503	416	IDTTSSSCQ	0.97/-1.3411052
	504	68	PKSGANFRD	0.78/-1.1525222
	505	275	VITNAVDCS	0.92/-1.2972589
	506	704	NNISLATQP	0.61/-0.99267762
	507	1093	VSQQLSDIS	0.81/-1.1971889
	508	506	STTVLDHTD	0.93/-1.324689
	509	239	PLTCNAISS	0.52/-0.92619456
	510	895	LGPHCGSSS	0.97/-1.3881777
	511	299	PNTGVYDLS	0.84/-1.2595447
	512	492	SASCPIGTN	0.60/-1.0339423
	513	91	QKPFLSDFN	0.68/-1.124113
	514	165	SKGSSRNES	0.61/-1.0572177
	515	75	RDLSLKGTT	0.93/-1.3958825
	516	772	EPFNVSFVN	0.51/-0.98344697
	517	865	LMQGVTLSS	0.80/-1.3086614
	518	173	SWHFDKSEP	0.52/-1.0291541
	519	497	IGTNYRSCE	0.78/-1.304046
	520	228	LGTLLSHYY	0.79/-1.3209309
	521	106	VKNTKLYVN	0.66/-1.2002027
	522	437	NNYNPSSWN	0.66/-1.2428289
	523	2	LLIIFILPT	0.66/-1.2466662
	524	556	CGVLDGSYN	0.66/-1.2651568
	525	1042	QALNSLLQQ	0.60/-1.2210328
	526	88	LWYQKPFLS	0.61/-1.2685105
	527	1030	LAKIQSVVN	0.51/-1.1870013
	528	568	LCSTDAFLG	0.99/-1.6797284
	529	114	NKTLYSEFS	0.57/-1.3044412
	530	1252	LTLNLHTIN	0.95/-1.7257947
	531	952	PILSESQIS	0.67/-1.5090783

	532	1069	LDALEAQVQ	0.61/-1.5813453
	533	913	DKVKLSDVG	0.79/-1.8528379
	534	1250	PNLTLNLHT	0.52/-1.6937584
	535	847	LDEVNGLLD	0.57/-1.8290063
